# Switchable Reactivities of Metalated Phosphasilenes Regulated by Reversible 1,2‐Metal Migration

**DOI:** 10.1002/anie.202518102

**Published:** 2025-12-16

**Authors:** Huaiyuan Zhu, Shicheng Dong, Xufang Liu, Xiyuan Li, Tobias Weng, Jun Zhu, Shigeyoshi Inoue

**Affiliations:** ^1^ TUM School of Natural Sciences Department of Chemistry Institute of Silicon Chemistry and Catalysis Research Center Technische Universität München (TUM) Lichtenbergstraße 4 85748 Garching bei München Germany; ^2^ School of Chemistry and Chemical Engineering Guangxi Colleges and Universities Key Laboratory of Applied Chemistry Technology and Resource Development Guangxi University Nanning Guangxi 530004 China; ^3^ School of Science and Engineering The Chinese University of Hong Kong No.2001 Longxiang Blvd. Longgang District Shenzhen Guangdong 518172 China

**Keywords:** CO activation, Metal migration, Phosphasilene, Silyl migration, Silylene

## Abstract

Anionic reagents with silicon‐containing double bonds, M(R)Si═ER_n_ (E = main group elements), have garnered significant interest owing to their unique metal‐mediated reactivity and their potential in transferring the Si═E unit. Within this domain, the intriguing field of *Si*‐metalated phosphasilenes remains uncharted. Herein, we present a novel strategy for the efficient synthesis of *P*‐metalated phosphasilenes and their subsequent transformation into *Si*‐metalated phosphasilenes via a distinctive phosphinosilylene intermediate formed during a metal‐mediated skeletal rearrangement. While *P*‐metalated phosphasilenes exhibit a pronounced π‐bonding character in the Si═P linkage, the skeletal rearrangement attenuates both the Si═P and Si─M bonds in the resulting *Si*‐metalated phosphasilenes, rendering them effective silylene equivalents. Moreover, formal transmetallation of *Si*‐metalated phosphasilene with dimetal carbonyl complexes provides access to a broader array of *Si*‐metalated analogues. This transmetallation proceeds through the phosphinosilylene intermediate, as evidenced by the isolation of a silylene–dimanganese complex, thereby revealing an uncharted mechanistic manifold for this class of transformations.

## Graphical abstract

We develop novel strategies for the facile synthesis of anionic phosphasilenes in the forms of both *P*‐metalation and *Si*‐metalation. The *P*‐metalated phosphasilene displays a pronounced π‐bond‐driven reactivity toward robust inert molecules, whereas the *Si*‐metalated analogue behaves as a silylene equivalent via a retro‐1,2‐silver migration.

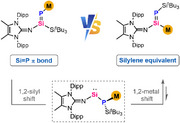

## Introduction

**Figure 1:**
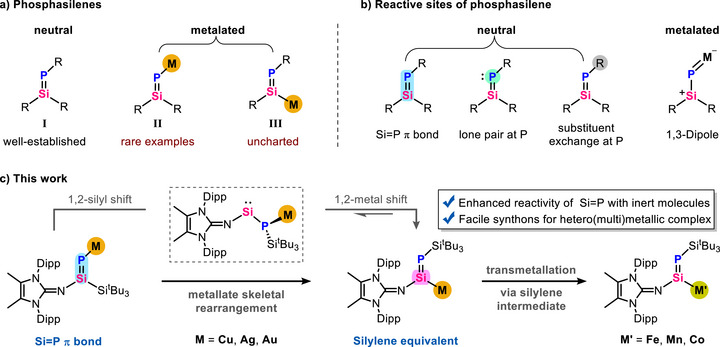
a) State‐of‐the‐art for phosphasilenes; b) Known active sites of phosphasilene; and c) Present work.

Without a doubt, the isolation of silene by Brook^[^ ^1^, ^2^ ^]^ disilene by West^[^ ^3^ ^]^ and diphosphene by Yoshifuji and Inamoto^[^ ^4^ ^]^ in 1981 marked one of the most significant milestones in modern main group chemistry. With that, the long‐existing so‐called “double bond rule”, which claims that heavy p‐block elements from the third period onward were incapable of forming π‐bonds with themselves or other elements, was finally disproven.^[^ ^5^, ^6^ ^]^ Building upon these pioneering findings, many efforts were then devoted to synthesizing heteronuclear phosphasilene featuring a silicon phosphorus double bond. Landmark achievements, including the spectroscopic characterization by Bickelhaupt in 1984^[^ ^7^ ^]^ and the structural elucidation by Niecke in 1993^[^ ^8^ ^]^ of stable phosphasilenes, have laid the foundation for subsequent breakthroughs in their synthesis and reactivity. Till date, a plethora of neutral phosphasilenes bearing organic substituents **I** have been isolated, characterized and exhaustively studied (Figure 1a).^[^ ^9^, ^10^, ^11^, ^12^, ^13^, ^14^ ^]^ In sharp contrast, anionic phosphasilenes with metal substituents (*P*‐metalated **II** and *Si*‐metalated **III**) are still underexplored, despite their unique structural and electronic properties.^[^ ^9^, ^15^ ^]^ Generally, neutral phosphasilenes feature classical reactivity sites, including the Si═P π bond, the phosphorus lone pair, and substitution at the phosphorus center,^[^ ^9^, ^10^, ^16^ ^]^ while *P*‐metalated phosphasilenes have thus far shown a distinctly enhanced 1,3‐dipolar reactivity at the SiPM fragment^[^ ^14^ ^]^ (Figure 1b). Metalated phosphasilenes are expected to offer the following potential applications: (i) enhanced chemical responsiveness of the Si═P bond towards inert molecules; (ii) facile synthons for the construction of hetero(multi)metallic complexes; and (iii) versatile mediators for the selective transfer of Si═P core. This motivates the synthesis of *P*/*Si*‐metalated phosphasilenes, allowing for a comparative analysis with neutral phosphasilenes and further explore their potential divergent reactivities. Since 2006, several examples of *P*‐metalated phosphasilenes have been successfully synthesized and structurally characterized by Driess and our group,^[^ ^14^, ^17^, ^18^, ^19^, ^20^ ^]^ however, to the best of our knowledge, the structural elucidation of *Si*‐metalated phosphasilene remains uncharted.^[^ ^21^ ^]^ This is likely attributed to the lack of applicable synthons and intrinsic instability of *Si*‐metalated phosphasilenes. For instance, although *Si*‐hydrophosphasilenes have been well‐documented for a long period,^[^ ^22^, ^23^ ^]^ the sila‐metalation of *Si*‐hydrophosphasilenes is thermodynamically disfavored due to the hydridic nature of the Si─H hydrogen.^[^ ^24^ ^]^ Therefore, a viable synthetic strategy must align with the thermodynamic constraints while preferentially stabilizing and preserving the integrity of the Si═P core.

Rearrangement reactions, a major category of organic reactions, enable the strategic reorganization of molecular frameworks through the cleavage of inert chemical bonds, thereby playing a pivotal role in both organic synthesis and natural biosynthetic events.^[^ ^25^ ^]^ Among these, silyl migration represents a fundamental rearrangement that has served as a powerful approach for skeletal editing in organosilane functionalization since the 1950s, exemplified by the well‐known Brook rearrangement.^[^ ^26^, ^27^, ^28^ ^]^ Metal migration is another compelling type of rearrangement, wherein metal substituents act as migrating groups. In contrast to conventional synthetic methodologies, metal migration offers distinct advantages in the formation of unique carbon–metal bonds, which can undergo further transformations into structurally complex and valuable compounds.^[^ ^29^, ^30^ ^]^ Notably, extensive mechanistic investigations have shown that the metal center remains covalently bound to the stationary scaffold through migration, resulting in a highly reversible metal migration process.^[^ ^31^, ^32^, ^33^, ^34^ ^]^ This dynamic process unlocks a broad range of divergent reaction pathways, significantly broadening the scope of accessible reactivity and offering unprecedented synthetic possibilities.

While 1,2‐silyl migrations and 1,2‐metal migrations are commonly observed in stoichiometric reactions and are often proposed as key steps in catalytic processes for the synthesis of organic and organometallic compounds,^[^ ^27^, ^29^, ^30^ ^]^ reactions involving the simultaneous occurrence of both 1,2‐silyl and 1,2‐metal migrations to construct structurally unique molecules remain scarce.^[^ ^35^, ^36^, ^37^ ^]^ Herein, we present the synthesis of the first *P*‐coinage‐metalated phosphasilenes by the coupling reaction of imino(silyl)silylene with a phosphide. Unprecedentedly, the *P*‐metalated phosphasilenes can undergo stepwise 1,2‐silyl migration and 1,2‐metal migration, allowing for the isolation of the first *Si*‐metalated phosphasilenes. The interchange of silyl and metal substituents profoundly alters their electronic properties, which in turn leads to distinct reactivity profiles. That is, *P‐*metalated phosphasilene exhibits pronounced π‐bond reactivity towards otherwise inert molecules, while *Si*‐metalated phosphasilene functions as a silylene equivalent for small molecule activation. These distinct reactivities are modulated via a reversible 1,2‐metal migration. Furthermore, formal transmetallation of *Si*‐metalated phosphasilene demonstrates its ability to transfer an unperturbed Si═P bond, while uncovering a previously unrecognized mechanistic pathway for this class of transformations (Figure 1c).

## Results and Discussion

### Synthesis of *P*/*Si*‐Metalated Phosphasilenes

In pursuit of a facile approach to access *Si*‐metalated phosphasilenes, we turned to our recently reported acyclic imino(silyl)silylene **1** bearing super silyl group (−Si*^t^* Bu_3_) and *N*‐heterocyclic imino (NHI) group as silicon skeletons.^[^ ^38^ ^]^ The principal advantage is that the NHI group provides both thermodynamic and kinetic stabilization,^[^ ^39^, ^40^ ^]^ while the Si*^t^* Bu_3_ group functions as an effective leaving group for the construction of Si–metal bond.^[^ ^41^, ^42^, ^43^ ^]^ In addition, the phosphaethynolate anion was strategically selected as the phosphorus unit owing to its well‐established propensity for decarbonylation to yield the phosphide P(I)^–^ species, which has been extensively utilized in the synthesis of phosphorus‐containing multiple bonds.^[^ ^44^, ^45^, ^46^, ^47^, ^48^, ^49^, ^50^ ^]^ We postulated that the coupling of silylene **1** with the phosphide P(I)^−^ species could result in the formation of a *P*‐metalated phosphasilene with the release of CO. This species may serve as a promising candidate for the synthesis of *Si*‐metalated phosphasilene by metallate skeletal rearrangements.

**Figure 2:**
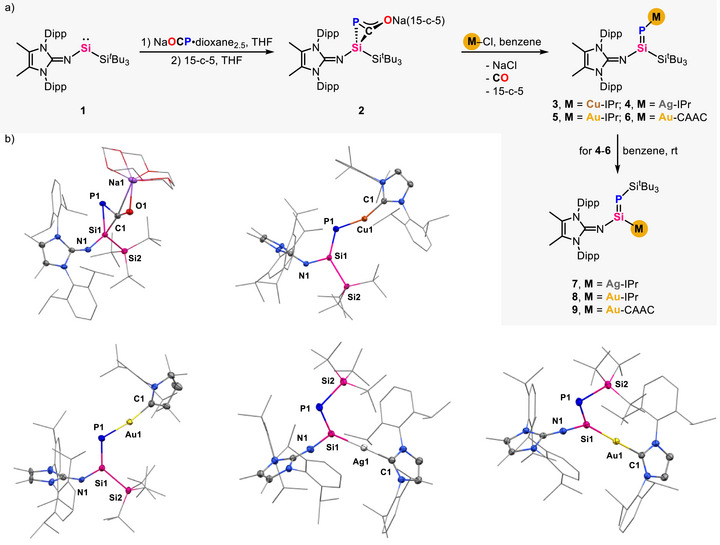
a) Preparation of 2*H*‐phosphasilirene‐3‐olate **2** and metalated phosphasilenes **3**–**9**; b) Molecular structures of **2** (top left), **3** (top right), **6** (bottom left), **7** (bottom middle), and **8** (bottom right).^[^ ^63^ ^]^ Ellipsoids set at 50% probability. Hydrogen atoms are omitted for clarity.

Thus, the dropwise addition of a THF solution of NaOCP·dioxane_2.5_^[^ ^51^ ^]^ to **1** in THF at room temperature resulted in bright yellow solution. After adding 15‐c‐5, 2*H*‐phosphasilirene‐3‐olate **2** was isolated as a yellow powder in 89% yield (Figure 2a). The single crystal X‐ray diffraction (SC‐XRD) analysis of **2** revealed slightly longer C1─P1 distance of 1.764(3) Å and C1─O1 distance of 1.258(3) Å than C═P bond in three‐membered ring systems (1.634(4) – 1.7629(20) Å)^[^ ^52^, ^53^, ^54^, ^55^, ^56^, ^57^, ^58^, ^59^, ^60^ ^]^ and typical C═O bond (1.19 –1.25 Å),^[^ ^61^, ^62^ ^]^ indicating the delocalization of negative charge in OCP motif and providing the potential for CO dissociation (Figure 2b). However, heating or irradiating a benzene or THF solution of **2** to generate the desired *P*‐sodiumiophosphasilene via decarbonylation consistently gave an ill‐defined mixture of species. Delightfully, treating **2** with coinage metal(I) chlorides (IPr·CuCl, IPr·AgCl, IPr·AuCl, and ^Me^CAAC·AuCl; IPr = 1,3‐bis‐(2,6‐diisopropylphenyl)imidazol‐2‐ylidene, ^Me^CAAC = 2,6‐diisopropylphenyl)‐4,4‐dimethyl‐2,2‐dimethyl‐pyrrolidin‐5‐ylidene) in benzene at room temperature cleanly afforded *P*‐coinage‐metalated phosphasilenes **3**–**6** with the release of CO and NaCl, respectively (Figure 2a). The ^31^P NMR spectrum displays one signal in the range of 40–70 ppm (**3**: 45.5 ppm; **4**: 40.2 ppm, ^107^Ag: *J*_P–Ag_ = 171.4 Hz, and ^109^Ag: *J*_P–Ag_ = 196.2 Hz; **5**: 66.2 ppm, **6**: 61.9 ppm). The ^29^Si{^1^H} NMR spectrum features a doublet (**3**: 122.7 ppm, *J*_Si─P_ = 174.2 Hz; **4**: 123.2 ppm, *J*_Si─P_ = 179.6 Hz; **5**: 139.2 ppm, *J*_Si─P_ = 179.4 Hz; no signal is observed for **6** due to the poor solubility and instability). These values are upfield shifted compared to *P*‐zinciophosphasilene (^31^P: 227 ppm, ^29^Si: 203 ppm)^[^ ^17^ ^]^ and *P*‐plumbyleniophosphasilene (^31^P: 292.5 ppm, ^29^Si: 227.0 ppm),^[^ ^18^ ^]^ presumably owing to the more electron‐rich phosphorus and silicon centers in **3**–**6**. The subsequent crystallographic studies unambiguously confirmed the formulation of **3** and **6**, as shown in Figure 2b. The Si1─P1 distances of 2.074(1) Å for **3** and 2.084(4) Å for **6** is close to that in the known *P*‐metalated phosphasilene (2.064(1)–2.085(2) Å).^[^ ^17^, ^18^ ^]^ It is noteworthy that **3**–**6** represent the first examples of *P*‐coinage‐metalated phosphasilenes, and its synthetic pathway introduces a novel strategy for constructing metalated agents with phosphorus‐containing multiple bonds.

Remarkably, leaving **4**–**6** at room temperature in C_6_D_6_ solution led to the appearance of a new species, as detected by the ^31^P NMR spectrum. Full consumption of **4**–**6** was achieved within 24 h, and the *Si*‐metalated phosphasilenes **7**–**9** were isolated quantitatively (Figure 2a). The ^31^P NMR spectra exhibit signals at −15.9 ppm (**7**, ^107/109^Ag: *J*_P─Ag_ = 19.7 Hz), −62.2 ppm (**8**) and −56.8 ppm (**9**), which lie in the typical range of *P*‐silylated phosphasilenes (−252.9 − 17.8 ppm).^[^ ^64^, ^65^, ^66^, ^67^ ^]^ Notably, the comparatively high‐field shifted ^31^P NMR resonances observed in **8** and **9** are most likely attributable to the greater electronegativity of gold. The relatively strong deshielding of the silicon in compounds **7**–**9**, indicated by the ^29^Si{^1^H} NMR signals at 209.4 ppm (**7**, *J*_Si─P_ = 160.4 Hz, ^107^Ag: *J*_Si─Ag_ = 338.0 Hz, and ^109^Ag: *J*_Si─Ag_ = 390.4 Hz), 202.7 ppm (**8**, *J*_Si─P_ = 148.8 Hz) and 208.7 ppm (**9**, *J*_Si─P_ = 151.2 Hz), is considered a diagnostic criterion for metalation at silicon compared to *P*‐metalated phosphasilenes **3**–**6**.^[^ ^15^ ^]^ These values are also consistent with those of *Si*‐metalated Si═E bond (E = Si, C, N, B, and O).^[^ ^68^, ^69^, ^70^, ^71^, ^72^, ^73^, ^74^, ^75^, ^76^, ^77^, ^78^, ^79^, ^80^, ^81^ ^]^ The molecular structures of **7** and **8** were definitively determined by SC‐XRD analysis, revealing a phosphasilene with Si─Ag or Si─Au bond formed by the interchange of silver or gold moiety with silyl group, respectively (Figure 2b). The Si1─P1 distances in **7** (2.127(9) Å) and **8** (2.111(1) Å) show slight deviations compared to that in **3** and **6**, suggesting more polarized Si═P bond. Notably, compounds **7**–**9** represent the first examples of *Si*‐metalated phosphasilenes featuring a Si─P double bond and a Si─M single bond. In addition, the isomerization from *P*‐metalated to *Si*‐metalated phosphasilenes is conceptualized as a rare example of a metallate skeletal rearrangement within a π‐bonded stationary scaffold,^[^ ^29^, ^82^, ^83^ ^]^ which is commonly proposed as a fundamental step in metal‐catalyzed reactions. Furthermore, the variable temperature (VT) ^31^P NMR experiments on **3** from 20 °C to −40 °C revealed an increasing signal at −198.5 ppm, indicating the potential for an equilibrium in this system (Figure S2.5). This signal is entirely distinct from those observed for **7**–**9**, which motivated a detailed investigation into the isomerization mechanism.

### DFT Calculations

**Figure 3:**
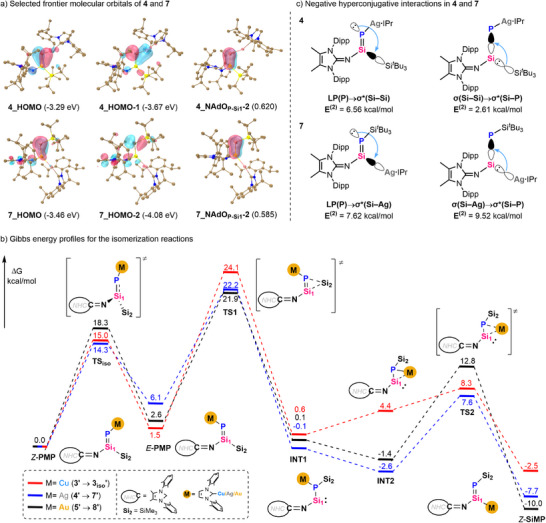
a) Selected frontier molecular orbitals of **4** and **7**. Hydrogen atoms in 3D structures are omitted for clarity. The isosurface 0.050 a.u. is plotted; b) Gibbs energy profiles for the isomerization reactions between **3′**, **4′**, **5′,** and **3_iso_’**, **7′**, **8′** in the TPSS‐D3(BJ)/def2‐TZVPP (SMD, benzene)//TPSS‐D3(BJ)/def2‐SVP level. Both isopropyl and *tert*‐butyl groups were simplified to methyl groups. The Gibbs energies are given in kcal/mol. c) Negative hyperconjugative interactions based on the second‐order perturbation analysis in **4** and **7**.

To gain insights into the electronic structure and bonding situation of *P*/*Si*‐metalated phosphasilenes, DFT calculations were carried out. The calculated structure of **3**–**9** (TPSS‐D3(BJ)/def2‐SVP level) is in good agreement with the experimental geometry (Table S2). Given the high consistency in the computational results for *P*‐metalated phosphasilenes **3**–**6** and *Si*‐metalated phosphasilenes **7**–**9** (Figures S19‐S20, S22‐S25; Tables S3‐S5), our analysis centers on silver‐substituted **4** and **7**. As shown in Figure 3a, the highest occupied molecular orbital (HOMO) of **4** (−3.29 eV) is predominantly composed of contributions from the π(Si═P) bonding orbital, whereas the HOMO‐1 (−3.67 eV) consists mainly of the lone pair on phosphorus. In contrast, the HOMO of **7** (−3.46 eV) mainly corresponds to a Si═P π‐bonding orbital, while the lone pair at P1 is located in HOMO‐2 (−4.08 eV). Natural adaptive orbital (NAdO) analysis^[^ ^84^, ^85^ ^]^ derived from the fuzzy bond order (FBO) analysis^[^ ^86^, ^87^ ^]^ confirms the explicit Si1─P1 multiple bond character with significant π‐bonding interaction for **4** and **7**, which is also reflected in the high Mayer bond order^[^ ^88^ ^]^ (MBO) of 1.643 and 1.495 (Table S3), respectively.

To gain a deeper understanding of the observed isomerization reactions and the distinct trends in copper‐, silver‐ and gold‐substituted phosphasilenes, detailed kinetic processes using simplified models (isopropyl and *tert*‐butyl groups were simplified to methyl groups) were explored through DFT calculations, as shown in Figure 3b. Certainly, the thermodynamic calculations of the isomerization reactions in the experimental model (Figures S27 and S28) show high consistency with those of the simplified model. This agreement underscores the significant validity and appropriateness of the model simplification. The computational results indicate that metallate skeletal rearrangement from *P*‐coinage‐metalated to *Si*‐coinage‐metalated phosphasilenes proceeds in two steps via a phosphinosilylene intermediate. Specifically, the first step involves the rotation of *P*‐coinage‐metalated phosphasilenes (*Z*‐**PMP**) furnishing the isomers *E*‐**PMP**, followed by 1,2‐silyl rearrangement from silicon to phosphorus to yield the phosphinosilylene intermediates **INT1**. This step is the rate‐determining step of the reaction, proceeding via a three‐membered‐ring transition state **TS1** (**TS1‐3′**, **TS1‐4′**, and **TS1‐5′**) with reaction barriers of 24.1, 22.2, and 21.9 kcal/mol, respectively, which are also validated by the full‐model molecules (Figure S27). Similar facile rotation of Si═P bond has been previously reported by our group.^[^ ^36^ ^]^ The relatively largest distances of P1─Si2 (2.796 Å) and Si1─Si2 (2.445 Å), along with their corresponding minimal MBOs (0.454 and 0.696), are the key factors for its lowest reaction barrier in **TS1‐5′** among these three transition states, which is consistent with our previous findings (Figure S29).^[^ ^89^, ^90^ ^]^ Upon the formation of phosphinosilylene intermediates, rapid rotation of the metal fragment at the phosphorus center occurs, enhancing the stabilization of the silylene through *σ*‐donation from the silylene to the metal center. This interaction also facilitates the subsequent migration of the metal unit from phosphorus to silicon. In addition, rotation of the metal unit does not significantly stabilize silylene center in the copper complex (**INT2‐3′**) compared to silver and gold complexes, with this step being endergonic by 3.8 kcal/mol. Following this, the second step is finally accomplished by the 1,2‐metal rearrangement from phosphorus to silicon through a three‐membered‐ring transition state **TS2** (**TS2‐3′** at 8.3 kcal/mol, **TS2‐4′** at 7.6 kcal/mol, and **TS2‐5′** at 12.8 kcal/mol). This results in the formation of thermodynamically more stable isomerized *Si*‐coinage‐metalated phosphasilenes (*Z*‐**SiMP**), with exergonicity by 2.5 kcal/mol (**3_iso_′**), 7.7 kcal/mol (**7′**), and 10.0 kcal/mol (**8′**), respectively. Notably, the transformation from *P*‐copperiophosphasilene **3′** to *Si*‐copperiophosphasilene **3_iso_′** is relatively thermoneutral (Δ*G* = 2.5 kcal/mol), consistent with the VT ^31^P NMR result indicating an equilibrium. Based on additional experimental evidence (vide infra), we attribute this ^31^P NMR resonance at −198.5 ppm to a *P*‐copper phosphinosilylene **3_int_**. The absence of an observable *Si*‐copperiophosphasilene **3_iso_** is likely attributable to the relatively low barrier for the reverse transformation to **INT1‐3′** (Δ*G* ^‡^ = 10.8 kcal/mol, Figure 3b), indicating **3_iso_** readily reverts to the *P*‐copper phosphinosilylene intermediate **3_int_**, even at low temperature. Furthermore, the Gibbs energy difference of **7′** (−7.7 kcal/mol) and **8′** (−10.0 kcal/mol), as well as the calculated MBOs of Si─M bond in **7** (0.842) and **8** (0.890, Table S3), suggest that **8** exhibits greater stability than **7**, likely due to enhanced relativistic effects in gold, which strengthen the bond between silicon and gold.^[^ ^91^, ^92^, ^93^ ^]^

In addition, second‐order perturbation theory analysis reveals that both compounds **4** and **7** exhibit two sets of negative hyperconjugative interactions (Figures 3c and S30). In **4**, the lone pair at P1 interacts with the σ*(Si─Si) orbital (*E* ^(2)^ = 6.56 kcal/mol), while the σ*(P─Si) orbital of the Si═P core can accept electron from the adjacent *σ*(Si─Si) orbital (*E* ^(2)^ = 2.61 kcal/mol). This results in the weakening of Si─Si bond, which enables the migration of silyl group from silicon to phosphorus. Nevertheless, the relatively weak interaction of *σ*(Si─Si)→σ*(Si─P) allows the Si═P double bond to retain a pronounced π‐character. In contrast, the stronger LP(P)→σ*(Si─Ag) and *σ*(Si─Ag)→σ*(Si–P) interactions of 7.62 and 9.52 kcal/mol in **7**, respectively, lead to significant attenuations of the Si─Ag and Si═P bond strengths. The weakening of the Si═P unit is reflected in the smaller Mayer bond order (MBO) of the Si═P bond in **7** compared to **4** (Table S3). This observation suggests that the silver moiety in **7** is kinetically more inclined to migrate back to the phosphorus center, thereby generating a phosphinosilylene intermediate that could enhance the nucleophilicity and overall reactivity of the silicon site. This finding is consistent with our calculated Gibbs energy profiles, in which the isomerization of **7′** to **INT1** via **TS2** exhibits a lower energy barrier than the corresponding isomerization of **4′** to **INT1** via **TS1** (Figure 3b).

### Distinct Reactivities of *P*/*Si*‐Metalated Phosphasilenes

Combined experimental observations and detailed DFT analysis revealed that *P*‐metalated and *Si*‐metalated phosphasilenes exhibit markedly different electronic properties, which may lead to distinct reactivities. This distinction was corroborated by subsequent reactivity studies with **4** and **7**.

**Figure 4:**
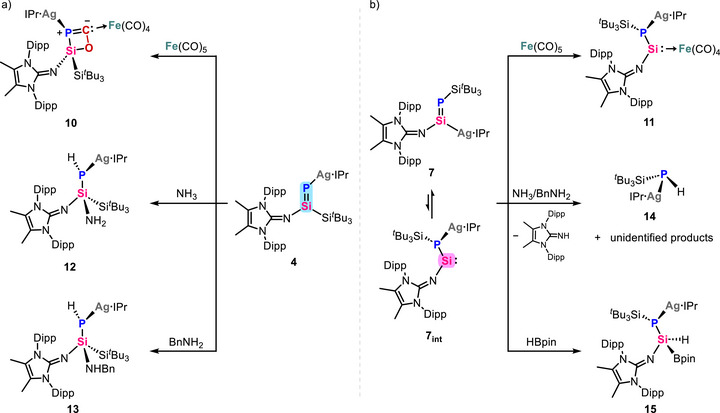
Distinct reactivities of *P/Si*‐silveriophosphasilenes towards same substrates.

**Figure 5:**
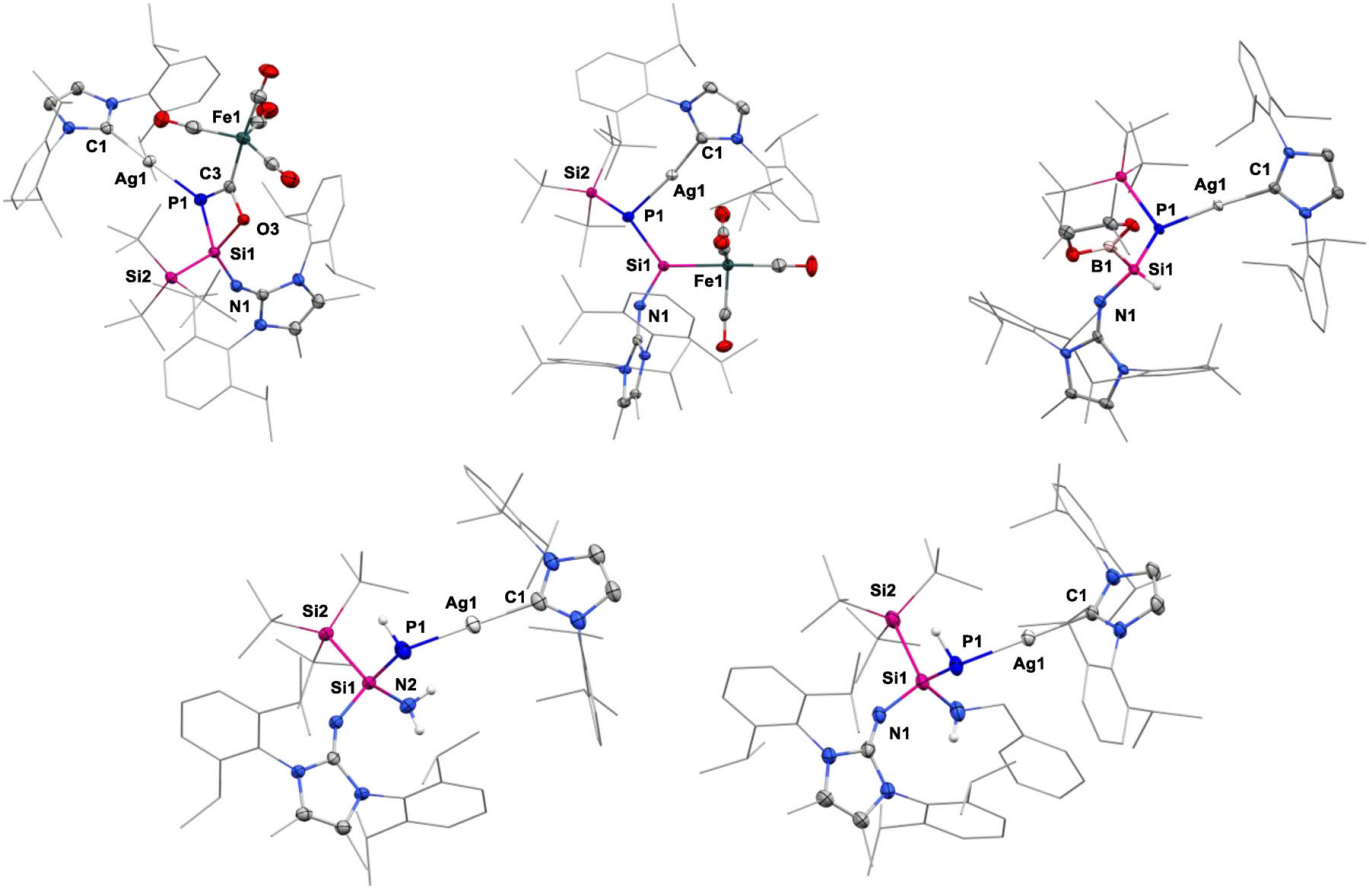
Molecular structures of **10** (top left), **11** (top middle), **12** (bottom left), **13** (bottom right), and **15** (top right).^[^ ^63^ ^]^ Ellipsoids set at 50% probability. Hydrogen atoms are omitted for clarity, except for the respective Si/N/P─H nuclei.

Reaction of **4** with iron carbonyl at room temperature resulted in the rapid and clean formation of heterobimetallic Ag‐Fe complex **10** (^31^P NMR: 168.4 ppm, ^107^Ag: *J*_P__─__Ag_ = 345.4 Hz, and ^109^Ag: *J*_P__─__Ag_ = 398.6 Hz) via [2 + 2] cycloaddition of Si═P double bond and CO moiety of iron carbonyl, which was confirmed by SC‐XRD analysis (Figures 4a and 5). The P1─C1 bond length (1.779(3) Å) falls between the typical distance of C─P single bond and double bond, indicating the partial double bond character in this C─P unit. Although carbon monoxide is widely recognized as a C1 feedstock for the synthesis of liquid hydrocarbons, its activation remains a significant challenge due to the high bond dissociation energy of the C≡O triple bond. In this context, the formation of compound **10** constitutes the first example of a [2 + 2] cycloaddition between CO and a multiple‐bonded compound, underscoring the pronounced π‐interaction of Si═P bond in **4**.^[^ ^94^, ^95^ ^]^ In contrast, reaction of **7** with iron carbonyl exclusively yielded heterobimetallic silylene‐iron complex **11** (^31^P NMR: −149.1 ppm, ^107^Ag: *J*_P─Ag_ = 292.5 Hz, and ^109^Ag: *J*_P─Ag_ = 337.7 Hz) (Figure 4b). The central silicon features a doublet at 210.4 ppm (*J*_Si─P_ = 132.0 Hz) in the ^29^Si{^1^H} NMR spectrum. In its solid‐state structure, the Si1─Fe1 bond length is 2.261(9) Å (Figure 5). The formation of **11** is considered through a retro‐1,2‐silver migration from silicon to phosphorus to generate the silylene intermediate **7_int_** followed by its coordination to the iron center. Further infrared spectroscopic analysis revealed that the CO stretching frequencies of **11** (2010, 1924, 1901, and 1870 cm^−1^) are the most red‐shifted among the reported silylene–iron carbonyl complexes,^[^ ^96^, ^97^, ^98^, ^99^ ^]^ indicating the exceptional *σ*‐donor strength of the silveriophosphino(imono)silylene **7_int_**. Notably, the synthesis of heterobimetallic Ag‐Fe complexes **10** and **11** demonstrates the unique advantage of metalated phosphasilenes as versatile and efficient synthons for the streamlined assembly of heterobimetallic frameworks.

Additionally, treating **4** with ammonia (1 bar) and benzylamine afforded the expected 1,2‐addition products **12**–**13**, respectively, while both reactions of **7** with ammonia (1 bar) or benzylamine resulted in the fragmentation of **7**, producing ^Me^IDippNH, silver‐substituted phosphine **14**, along with unidentified products (Figure 4a). Compounds **12**–**13** and **14** were fully characterized by the NMR spectroscopy, LIFDI mass. The ^31^P NMR spectra exhibit signals at −202.6 ppm (**12**, *J*_P─H_ = 174.2 Hz, ^107^Ag: *J*_P─Ag_ = 189.1 Hz, and ^109^Ag: *J*_P─Ag_ = 214.0 Hz), −233.9 ppm (**13**, *J*_P─H_ = 175.3 Hz, ^107^Ag: *J*_P__─__Ag_ = 188.2 Hz, and ^109^Ag: *J*_P─Ag_ = 217.0 Hz) and −313.0 ppm (**14**, *J*_P─H_ = 170.9 Hz, ^107^Ag: *J*_P─Ag_ = 183.7 Hz, and ^109^Ag: *J*_P─Ag_ = 212.0 Hz). The molecular structures of **12**–**13** were undoubtedly determined by SC‐XRD analysis, showing both *sp* ^3^‐hybridized Si1 and P1 centers with bond length of 2.262(1) Å for **12** and 2.251(1) Å for **13** (Figure 5). Given the inherent challenges associated with both ammonia activation and its transfer, the cleavage of N─H bond in ammonia by **4** further highlights the strong π‐interaction of Si═P bond.^[^ ^100^, ^101^, ^102^, ^103^, ^104^, ^105^ ^]^ Meanwhile, the isolation of **14** provides evidence for the existence of silylene intermediate **7_int_** via retro‐1,2‐silver migration and its follow‐up oxidative addition towards N─H bond. Additional experimental evidence supporting the involvement of the silylene intermediate **7_int_** was obtained from the reaction of **7** with HBpin, which afforded **15** by the oxidative addition of HBpin to **7_int_** (Figures 4b and 5).

Overall, *P*‐metalated phosphasilene behaves as a π‐bond, exhibiting enhanced reactivity toward robust molecules such as ammonia and CO ligand in iron carbonyl. In contrast, *Si*‐metalated phosphasilene mimics silylene behavior, showing exceptional *σ*‐donor capabilities for small molecule activation. This divergence in reactivity is governed by a reversible 1,2‐metal migration, driven by the comparatively weaker Si─Ag and Si═P bonds in the *Si*‐metalated species.

### Formal Transmetallation of *Si*‐Metalated Phosphasilene via Silylene Intermediate

**Figure 6:**
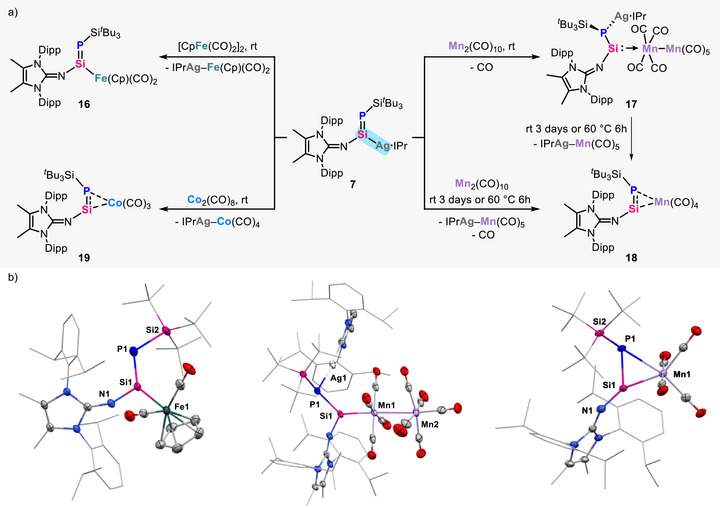
a) Formal transmetallation of **7** towards metal carbonyls. b) Molecular structures of **16** (left), **17** (middle), and **18** (right). Ellipsoids set at 50% probability.^[^ ^63^ ^]^ Hydrogen atoms are omitted for clarity.

The utility of *Si*‐metalated phosphasilene for transferring an unperturbed Si═P bond was further showcased through diverse transmetallation reactions. Treating **7** with [CpFe(CO)_2_]_2_ gave rise to the formation of novel *Si*‐ironiophosphasilene **16** with the elimination of IPrAg‐Fe(Cp)(CO)_2_ (Figure 6a). The ^31^P NMR spectrum exhibits a singlet at 39.7 ppm and its corresponding silicon signal is observed at 148.9 ppm (*J*_Si─P _= 184.5 Hz) in the ^29^Si{^1^H} NMR spectrum. In its solid‐state structure, the Si1─P1 distance of 2.107(7) Å is comparable with that in **3** and **7** (Figure 6b). Reaction of **7** with Mn_2_(CO)_10_ led to the rapid evolution of carbon monoxide gas, a behavior markedly distinct from that observed with [CpFe(CO)_2_]_2_ (Figure 6a). In the ^31^P NMR spectrum, only one signal is observed at −154.0 ppm (^107^Ag: *J*_P─Ag_ = 213.5 Hz and ^109^Ag: *J*_P─Ag_ = 244.9 Hz), providing strong evidence for a direct P─Ag bonding interaction. The molecular structure of **17** was unambiguously determined by SC‐XRD analysis, showing the presence of an imino(phosphino)silylene dimanganese framework with a Mn─Mn bond length of 2.896(7) Å (Figure 6b). The Si1─Mn1 bond length is 2.281(1) Å, which is similar to those of silylene‐manganese complexes.^[^ ^106^, ^107^ ^]^ To the best of our knowledge, **17** represents the first example of a silylene‐dimanganese complex. Concurrently, the pronounced donor strength of silylene intermediate **7_int_** was again evidenced by the significantly red‐shifted CO stretching mode (2072, 2045, 2033, 1996, 1965, 1938, and 1918 cm^−1^) compared with those of phosphine‐, NHC‐ or germylene‐dimanganese carbonyl complexes.^[^ ^106^, ^108^, ^109^ ^]^ Of particular interest, stirring a benzene solution of **17** at room temperature for 3 days or heating to 60 °C for 6 h resulted in an elimination of IPr‐AgMn(CO)_5_ to form **18**, as detected by ^31^P NMR spectrum (from −154.0 to −496.5 ppm) (Figure 6a). This unique ^31^P chemical shift is comparable to our previously proposed *Si*‐platinumiophosphasilene intermediate with an η^2^ coordination mode (−308.7 ppm).^[^ ^21^ ^]^ Subsequent SC‐XRD analysis elucidated the molecular structure of **18**, revealing that the Si1─P1 moiety engages in π‐electron coordination to the Mn1 atom in an *η* ^2^ mode, with a slightly elongated Si1─P1 bond (2.142(1) Å) (Figure 6b). Further reaction of **7** with Co_2_(CO)_8_ afforded *Si*‐cobaltiophosphasilene **19** without the observation of a silylene‐dicobalt intermediate. The ^31^P NMR spectrum displays a chemical shift at −521.9 ppm, closely matching that of **18**, thereby supporting the assignment of an *η* ^2^‐coordination mode involving the Si═P bond to the cobalt center.

Based on experimental observations, we proposed that these transmetallation reactions proceed via a silylene intermediate. That is, *Si*‐silveriophosphasilene **7** initially undergoes retro‐1,2‐silver shift from silicon to phosphorus to generate silylene intermediate **7_int_** that simultaneously binds to metal complexes, affording silylene‐metal carbonyl complexes. As revealed by detailed SC‐XRD analysis and DFT calculations (Figures 6b, S26, Table S3‐S4), these silylene‐metal complexes possess weak P─Ag and metal–metal bond, which facilitate subsequently elimination of the corresponding silver‐metal complexes to yield the new *Si*‐metalated phosphasilenes. Our approach bypasses conventional transmetallation or salt metathesis,^[^ ^15^, ^16^, ^70^, ^80^, ^110^, ^111^, ^112^ ^]^ instead harnessing silylene–metal complex intermediates to transfer main‐group multiple bonds.

## Conclusion

In summary, we report a novel and efficient strategy for synthesizing *P*‐metalated phosphasilenes, achieved by the coupling of silylene and phosphide. Unprecedentedly, *P*‐metalated phosphasilenes (M = Ag and Au) undergo metallate skeletal rearrangements to form uncharted *Si*‐metalated phosphasilenes that are challenging to access using classic methods. Mechanistically, the reaction starts with 1,2‐silyl rearrangement from silicon to phosphorus to generate a phosphinosilylene intermediate, which then undergoes 1,2‐metal rearrangement from phosphorus to silicon to ultimately deliver the thermodynamically more stable *Si*‐metalated phosphasilene, as supported by in‐depth DFT calculations. This skeletal rearrangement fundamentally alters the electronic properties of metalated phosphasilenes, as evidenced by the difference in negative hyperconjugations, thus exposing distinct reactivities. Specifically, *P‐*metalated phosphasilene exhibits pronounced π‐bond reactivity pattern towards otherwise inert molecules, while *Si*‐metalated phosphasilene functions as a silylene equivalent via retro‐1,2‐metal migration. These metalated phosphasilenes serve as versatile and efficient synthons for the modular construction of diverse hetero(multi)metallic frameworks. Furthermore, transmetallation of *Si*‐silveriophosphasilene with metal carbonyl complexes provides access to a broader array of *Si*‐metalated phosphasilenes. This formal transmetallation proceeds through the phosphinosilylene intermediate, as supported by the isolation of a silylene‐dimanganese complex, thereby revealing an uncharted mechanistic manifold for this class of transformations. Our results strongly support the proposed reversible metal‐migration process, in which the metal center remains covalently anchored to the stationary framework. These insights enhance our understanding of reactivity patterns and may inspire the development of more efficient catalytic systems.

## Conflict of Interests

The authors declare no conflict of interest.

## Supporting information



Supporting Information

Supporting Information

## Data Availability

The data that support the findings of this study are available in the Supporting Information of this article.
