# Supplementary material for: Switchable Reactivities of Metalated Phosphasilenes Regulated by Reversible 1,2‐Metal Migration
Source: Angew Chem Int Ed Engl. 2025 Dec 16;65(5):e18102. doi: 10.1002/anie.202518102 (PMC12851009; doi:10.1002/anie.202518102)
Supplement: Supplementary file 1 — Supporting Information [file ANIE-65-e18102-s002.pdf]

Supporting Information

Switchable Reactivities of Metalated Phosphasilenes Regulated by Reversible 1,2-Metal Migration

Huaiyuan Zhu,[‡] Shicheng Dong,[‡] Xufang Liu, Xiyuan Li, Tobias Weng, Jun Zhu and Shigeyoshi Inoue**

Contents

1. Experimental Procedures ... 3
1.1 General Methods and Instrumentation ... 3
1.2 Synthesis and Characterization ... 4
1.2.1 Synthesis of 2*H*-phosphasilirene-3-olate (**2**) ... 4
1.2.2 Synthesis of *P*-Copperiophosphasilene (**3**) ... 7
1.2.3 Synthesis of *P*-Silveriophosphasilene (**4**) ... 11
1.2.4 Synthesis of *P*-Goldiophosphasilene (**5**) ... 14
1.2.5 Synthesis of *P*-Goldiophosphasilene (**6**) ... 17
1.2.6 Synthesis of *Si*-Silveriophosphasilene (**7**) ... 19
1.2.7 Synthesis of *Si*-Goldiophosphasilene (**8**) ... 22
1.2.8 Synthesis of *Si*-Goldiophosphasilene (**9**) ... 25
1.2.9 Synthesis of Carbene-iron complex (**10**) ... 28
1.2.10 Synthesis of Silylene-iron complex (**11**) ... 32
1.2.11 Synthesis of Aminosilane (**12**) ... 36
1.2.12 Synthesis of Aminosilane (**13**) ... 39
1.2.13 Synthesis of Silver-substituted Hydrophosphine (**14**) ... 42
1.2.14 Synthesis of Hydrosilane (**15**) ... 45
1.2.15 Synthesis of *Si*-ironiophosphasilene (**16**) ... 49
1.2.16 Synthesis of Silylene-mangenese complex (**17**) ... 53
1.2.17 Synthesis of *Si*-mangeneseiophosphasilene (**18**) ... 57
1.2.18 Synthesis of *Si*-cobaltiophosphasilene (**19**) ... 61
2. Single Crystal X-Ray Structure Determination ... 65
3. Computational Details ... 71
4. Appendix: Cartesian coordinates of the optimized geometries ... 83
5. References ... 156

1. Experimental Procedures

1.1 General Methods and Instrumentation

All experiments and manipulations were carried out under argon atmosphere using standard Schlenk or glovebox techniques. The glassware was heat-dried under vacuum prior to use. All glass junctions were coated with PTFE-based grease Merkel Triboflon III. For stirring, PTFE-coated magnetic stirrer bars were used or glass-coated ones if stated. Liquid phases were transferred using standard PE/PP syringes equipped with stainless steel cannula or directly canted from vessel to vessel if not stated otherwise. Solvents were dried by standard methods (withdrawal from MBraun Solvent Purification System and storage over molecular sieves (3 Å) or distilled from sodium/benzophenone or CaH_2 under argon atmosphere and degassed via freeze-pump-thaw cycling). All chemicals were purchased from commercial suppliers and used as received if not stated otherwise. Deuterated benzene (C_6D_6), Tol-D_8, THF-D_8 were obtained from Deutero Deutschland GmbH and were dried over potassium minor. All NMR samples were prepared under argon in J. Young sealed tubes with PTFE caps. NMR spectra were monitored on a Bruker AV400US, DRX400, or AV500cr at ambient temperature (300 K) if not stated otherwise. ^{1}H and ^{13}C NMR spectra were calibrated against the residual proton and natural abundance carbon resonances of the respective deuterated solvent as internal standard. The following abbreviations are used to describe signal multiplicities: s = singlet, d = doublet, t = triplet, sept = septet, m = multiplet, br = broad and combinations thereof. Some NMR spectra include resonances for silicone grease (C_6D_6: (^{1}H) = 0.29 ppm, (^{13}C) = 1.4 ppm and (^{29}Si) = –21.8 ppm) derived from *B. Braun Melsungen AG Sterican*® cannulas. Liquid Injection Field Desorption Ionization Mass Spectrometry (LIFDI-MS) was measured directly from an inert atmosphere glovebox with a Thermo Fisher Scientific Exactive Plus Orbitrap equipped with an ion source from Linden CMS. ATRFT-IR spectra were recorded on a Bruker Alpha FT-IR spectrometer (diamond ATR, located inside an argon-filled glovebox) in a range of 500–4000 cm^{-1}. **1**[S1], NaOCP·dioxane$_{2.5}$[S2], IPr·CuCl[S3], IPr·AgCl[S4], IPr·AuCl[S5] and ^{Me}CAAC·AuCl[S6] were synthesized according to procedures described in literature.

1.2 Synthesis and Characterization

1.2.1 Synthesis of 2*H*-phosphasilirene-3-olate (2)

A solution of NaOCP·dioxane$_{2.5}$ (302.2 mg, 1.0 mmol) in THF (5 mL) was added dropwise to acyclic silylene **1** (658.2 mg, 1.0 mmol) in THF (10 mL) at room temperature. The color of mixture changed to bright yellow from blue gradually. After adding 15-crown-5 (220.3 mg, 1.0 mmol), the mixture was allowed to stir for 1 h at room temperature. All volatiles were removed *in vacuum*, the resulting yellow solid was washed with cold pentane (3 × 10 mL). The remaining solid was dried *in vacuum* to yield anionic phosphasilirene **2** as a yellow powder (821.6 mg, 89%). Crystal suitable for single crystal X-ray diffraction analysis was obtained by storing a THF/pentane (1:1) solution at –30 °C for 2 days.

^{1}H NMR (400.1 MHz, THF-D$_8$): δ [ppm] 7.18-7.21 (m, 2H, *p*-C*H*-Dipp), 7.07-7.12 (m, 4H, *m*-C*H*-Dipp), 3.68 (s, 20H, 15-c-5), 3.25 (sept, J = 6.8 Hz, 2H, C*H*(CH$_3$)$_2$), 3.10 (sept, J = 6.8 Hz, 2H, C*H*(CH$_3$)$_2$), 1.62 (s, 6H, NCC*H*$_3$), 1.39 (d, J = 6.8 Hz, 6H, CH(C*H*$_3$)$_2$), 1.34 (d, J = 6.8 Hz, 6H, CH(C*H*$_3$)$_2$), 1.14 (d, J = 6.8 Hz, 6H, CH(C*H*$_3$)$_2$), 1.09 (d, J = 6.8 Hz, 6H, CH(C*H*$_3$)$_2$), 0.97 (s, 27H, C(C*H*$_3$)$_3$).

^{13}C{^{1}H} NMR (100.6 MHz, THF-D$_8$): δ [ppm] 262.7 (P*C*O, J_{C-P} = 103.2 Hz), 147.5 (N*C*N), 147.1 (Ar*C*), 140.1 (Ar*C*), 134.9 (Ar*C*), 127.8 (Ar*C*) 123.5 (Ar*C*), 123.2 (Ar*C*), 115.7 (N*C*-CH$_3$), 69.3 (15-c-5), 32.2 (C(*C*H$_3$)$_3$), 28.5 (*C*H(CH$_3$)$_2$), 28.3 (*C*H(CH$_3$)$_2$), 23.7 (CH(*C*H$_3$)$_2$), 23.4 (CH(*C*H$_3$)$_2$), 23.0 (*C*(CH$_3$)$_3$), 9.7 (NC-*C*H$_3$).

^{29}Si{^{1}H} NMR (79.5 MHz, THF-D$_8$): δ [ppm] 3.2 (*Si*^{t}Bu$_3$), –109.4 (Si*Si*P, J_{Si-P} = 42.4 Hz).

^{31}P NMR (162.0 MHz, THF-D$_8$): δ [ppm] 93.4 (Si*P*C)

LIFDI-MS: Calcd (-Na(15-c-5)): 716.4560; Found: 716.4575.

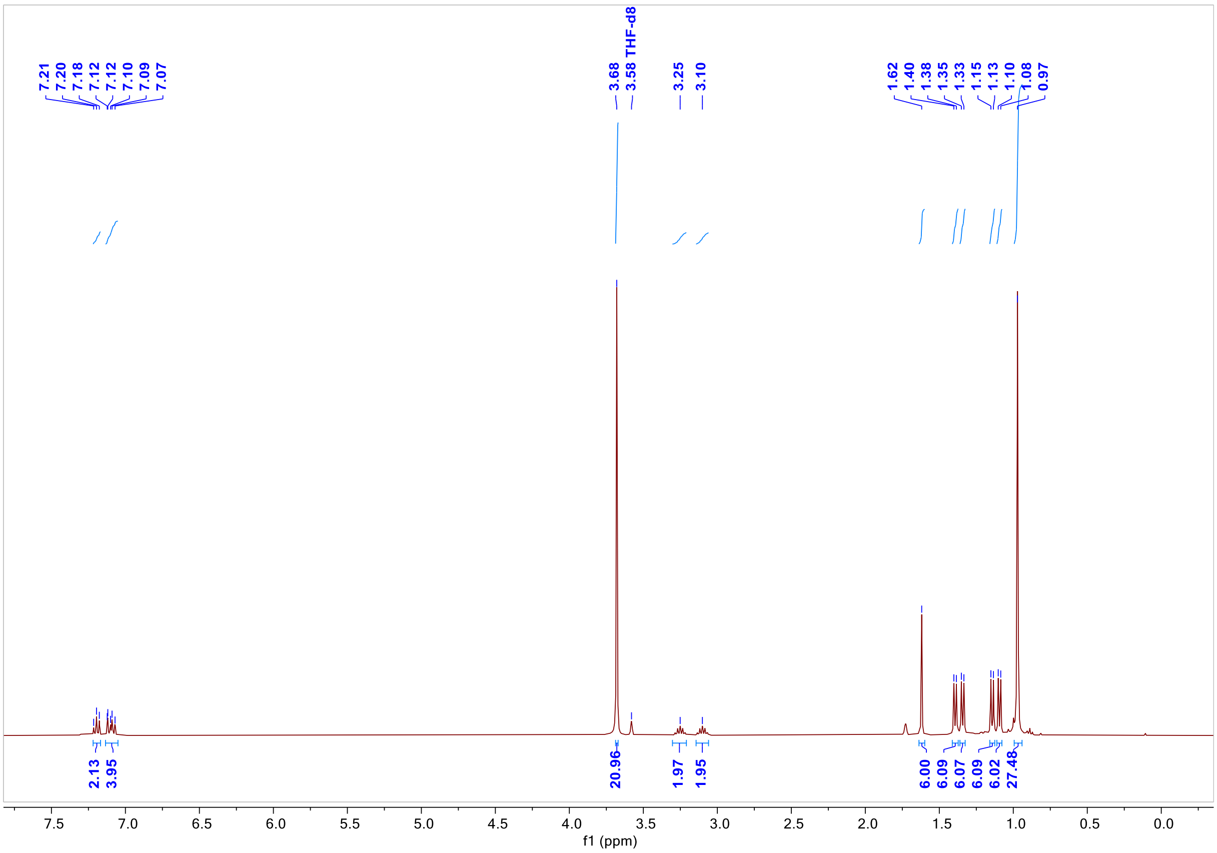

Figure S1.1 ^{1}H NMR spectrum of **2** in THF-D$_8$ at 300 K.

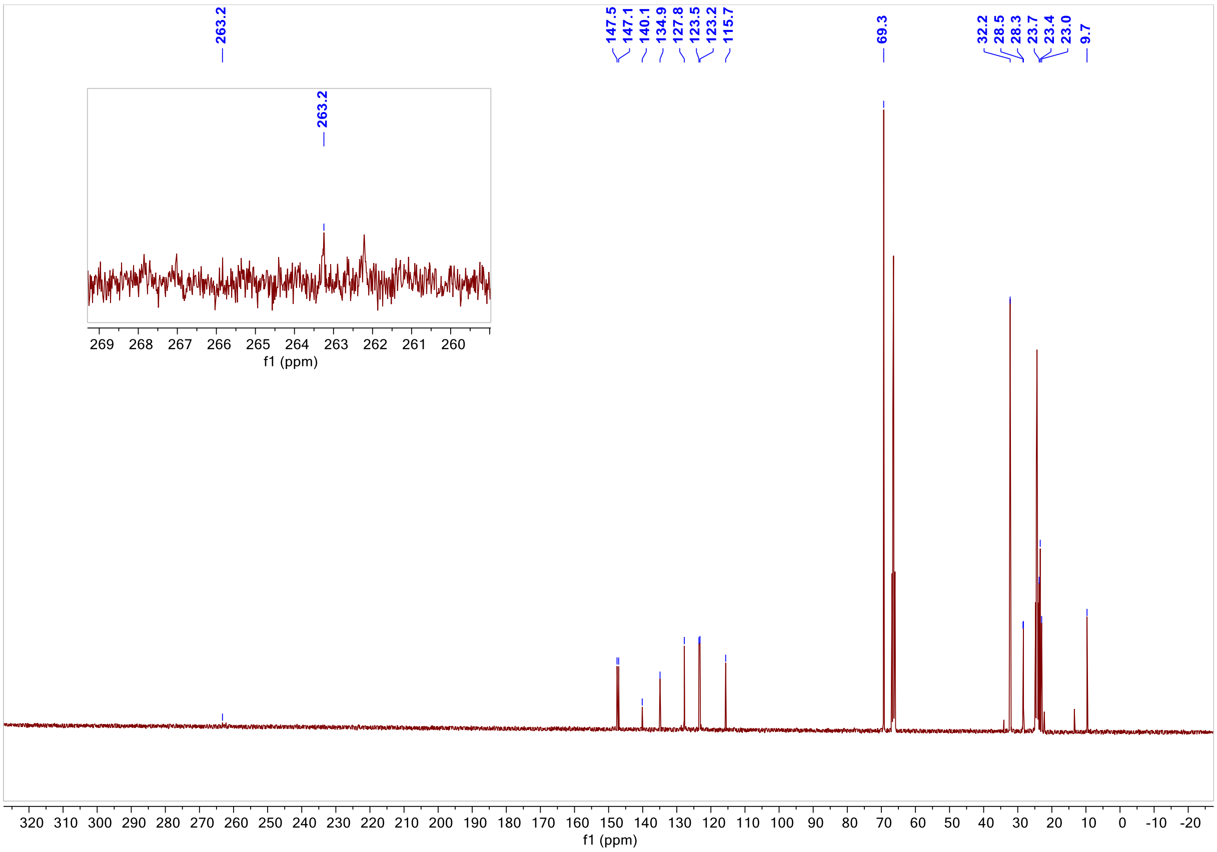

Figure S1.2 $^{13}C\{^{1}H\}$ NMR spectrum of **2** in THF-D$_8$ at 300 K.

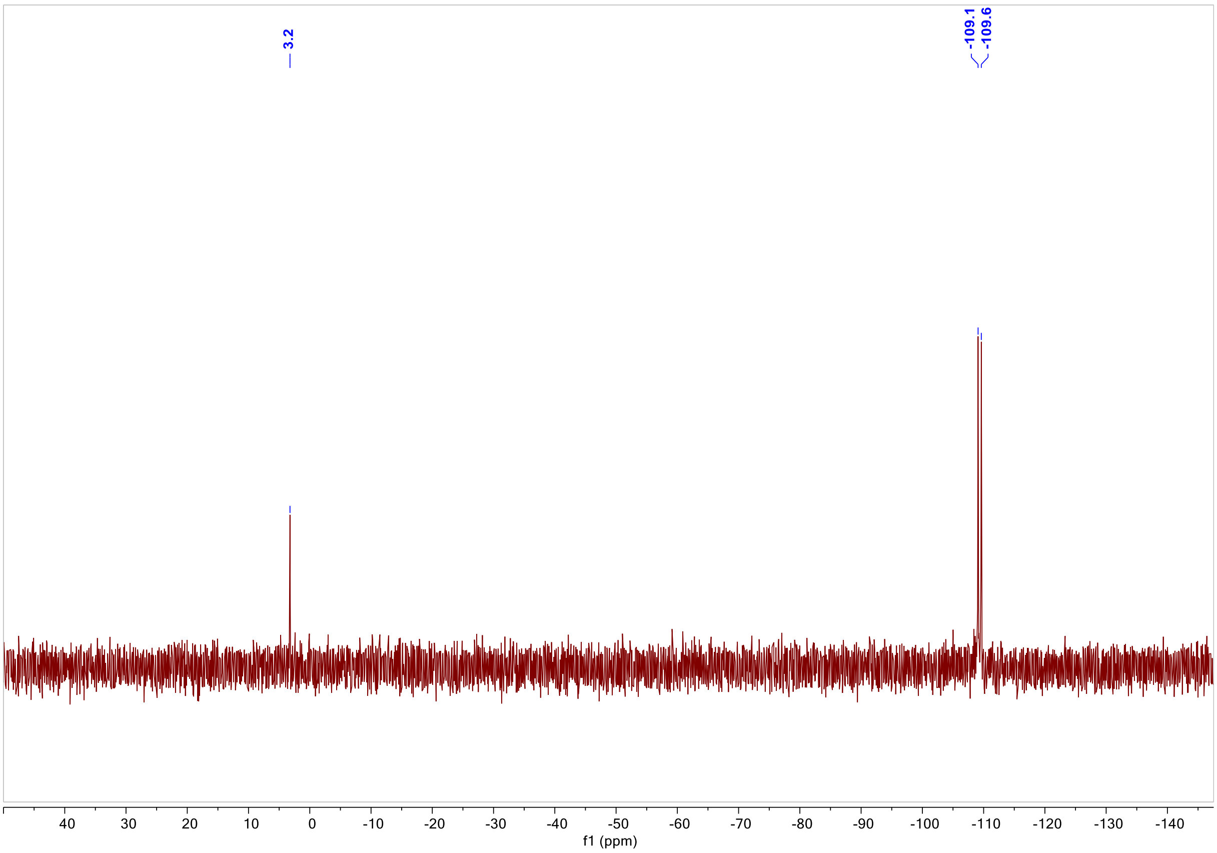

Figure S1.3 $^{29}Si\{^{1}H\}$ NMR spectrum of **2** in THF-D$_8$ at 300 K.

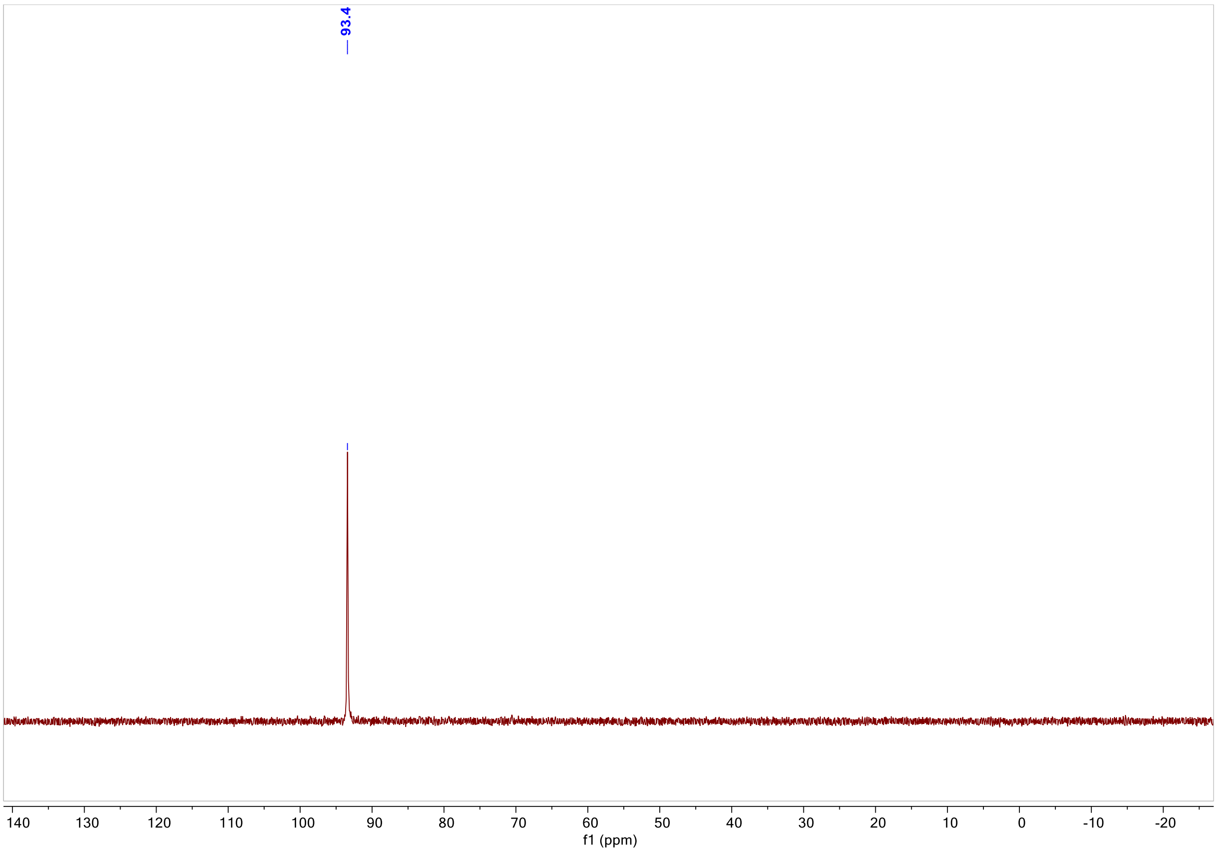

Figure S1.4 ^{31}P NMR spectrum of **2** in THF-D$_8$ at 300 K.

1.2.2 Synthesis of *P*-Copperiophosphasilene (3)

2*H*-phosphasilirene-3-olate **2** (96 mg, 0.1 mmol) and IPr·CuCl (48.8 mg, 0.1 mmol) were combined in benzene (2 mL) at room temperature. The color of mixture turned to orange from yellow rapidly with the release of CO. After work-up, all volatiles were removed *in vacuum* and the residue was extracted with pentane (3 × 3 mL). Pentane was removed *in vacuum* to yield *P*-copperiophosphasilene **2** (105 mg, 92%) as an orange power. Crystal suitable for single crystal X-ray diffraction analysis was obtained by storing a saturated pentane solution at –30 °C for 3 days.

^{1}H NMR (400.1 MHz, C_6D_6): δ [ppm] 7.31-7.34 (m, 2H, Ar*H*), 7.23-7.25 (m, 6H, Ar*H*), 7.18-7.20 (m, 4H, Ar*H*), 6.34 (s, 2H, NC*H*), 3.77 (sept, *J* = 6.8 Hz, 4H, C*H*(CH_3)$_2$), 2.83 (sept, *J* = 6.8 Hz, 4H, C*H*(CH_3)$_2$), 1.67 (s, 6H, NCC*H*$_3$), 1.51 (d, *J* = 6.8 Hz, 12H, CH(C*H*$_3$)$_2$), 1.42 (d, *J* = 6.8 Hz, 12H, CH(C*H*$_3$)$_2$), 1.15 (s, 27H, C(C*H*$_3$)$_3$), 1.15 (d, *J* = 6.8 Hz, 12H, CH(C*H*$_3$)$_2$), 1.11 (d, *J* = 6.8 Hz, 12H, CH(C*H*$_3$)$_2$).

^{13}C{^{1}H} NMR (100.6 MHz, C_6D_6): δ [ppm] 184.4 (C_{NHC}, $J_{C–P}$ = 32.0 Hz), 151.6 (N*C*N), 148.7 (Ar*C*), 145.5 (Ar*C*), 135.8 (Ar*C*), 133.3 (Ar*C*), 129.8 (Ar*C*), 128.8 (Ar*C*), 124.1 (Ar*C*), 124.0 (Ar*C*), 121.9 (N*C*H), 118.0 (N*C*-CH_3), 32.4 (C(*C*H$_3$)$_3$), 28.6 (*C*H(CH_3)$_2$), 28.1 (*C*H(CH_3)$_2$), 26.2 (*C*H(CH_3)$_2$), 26.1 (*C*H(CH_3)$_2$), 25.0 (CH(*C*H$_3$)$_2$), 24.3 (CH(*C*H$_3$)$_2$), 24.2 (CH(*C*H$_3$)$_2$), 24.0 (*C*(CH_3)$_3$), 10.4 (NC-*C*H$_3$).

^{29}Si{^{1}H} NMR (79.5 MHz, C_6D_6): δ [ppm] 0.8 (d, $J_{Si–P}$ = 14.6 Hz, *Si*^{t}Bu$_3$), 122.7 (d, $J_{Si–P}$ = 174.2 Hz, *Si*=P).

^{31}P NMR (162.0 MHz, C_6D_6): δ [ppm] 45.5 (Si=*P*).

LIFDI-MS: Calcd: 1139.6780; Found: 1139.6777.

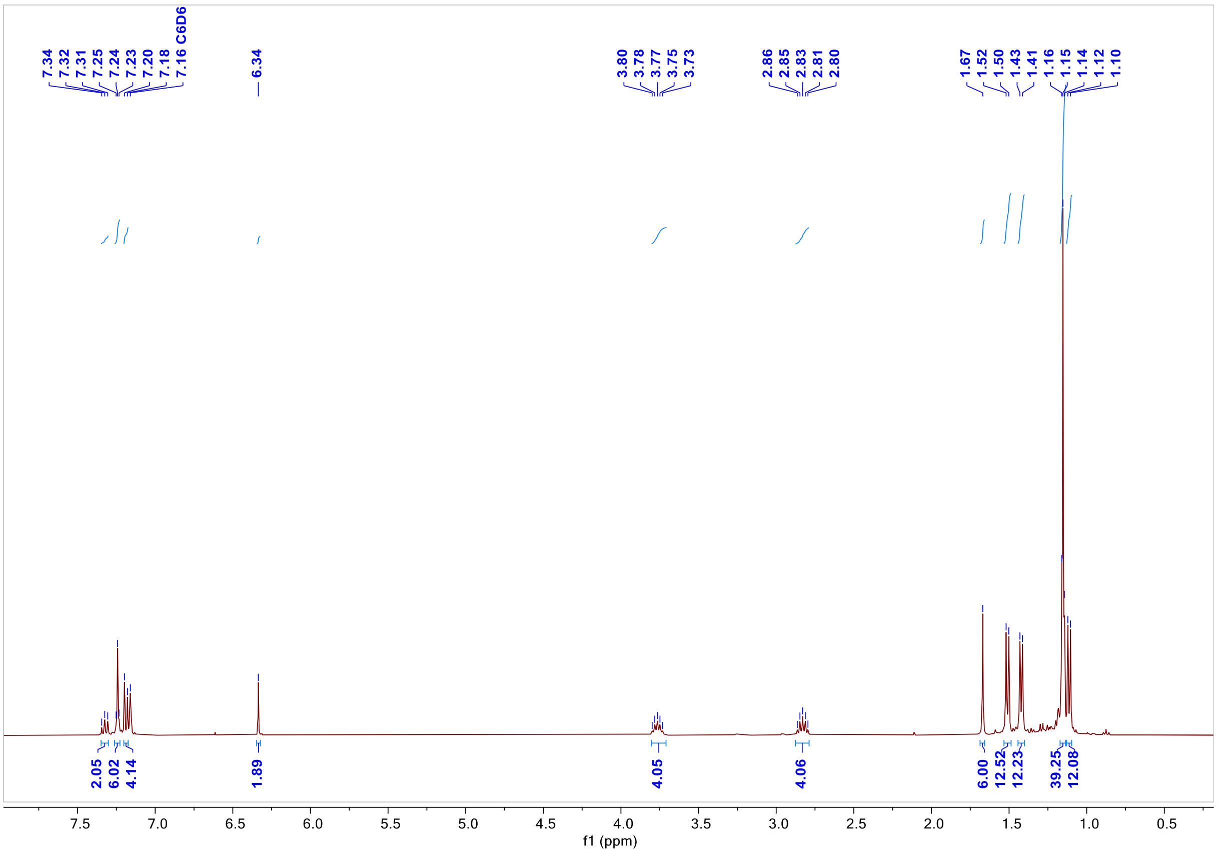

Figure S2.1 ^{1}H NMR spectrum of **3** in C_6D_6 at 300 K.

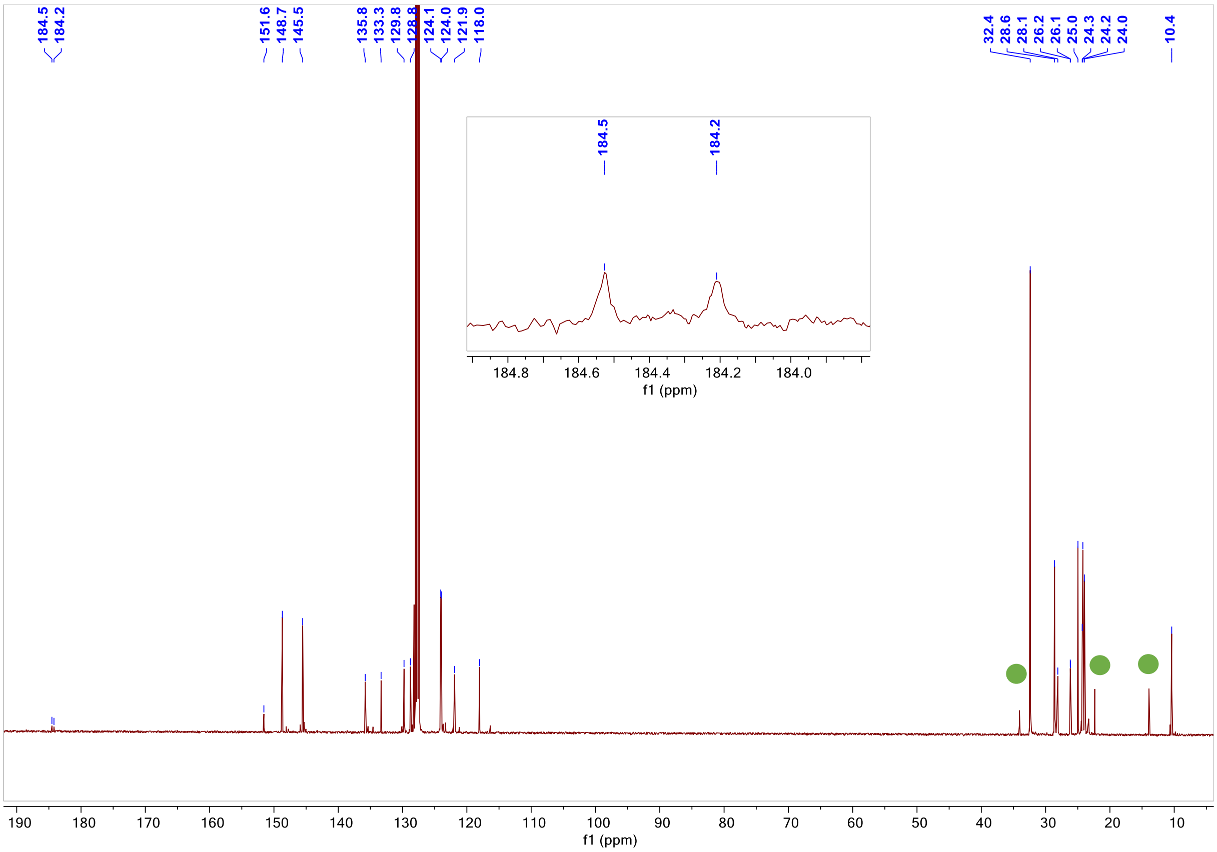

Figure S2.2 $^{13}C\{^{1}H\}$ NMR spectrum of **3** in C_6D_6 at 300 K (●: pentane).

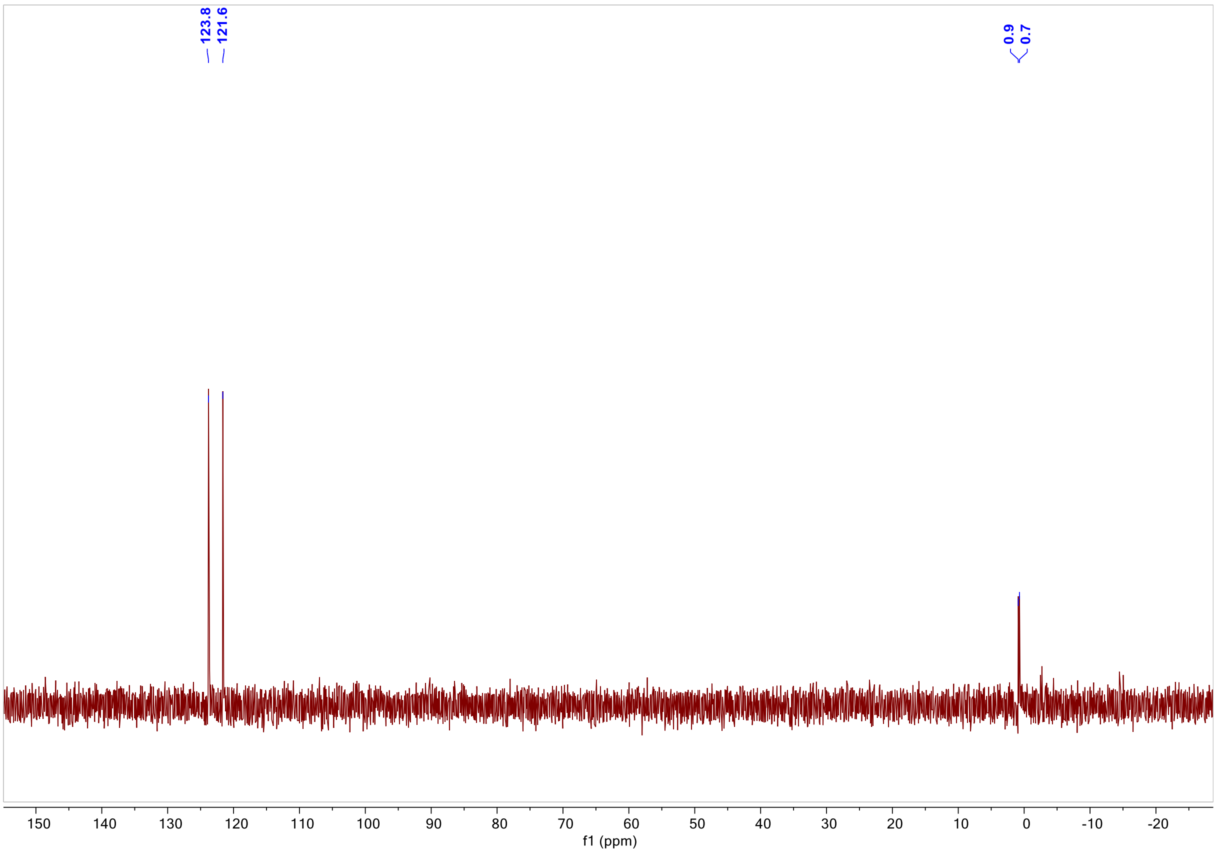

Figure S2.3 $^{29}Si\{^{1}H\}$ NMR spectrum of **3** in C_6D_6 at 300 K.

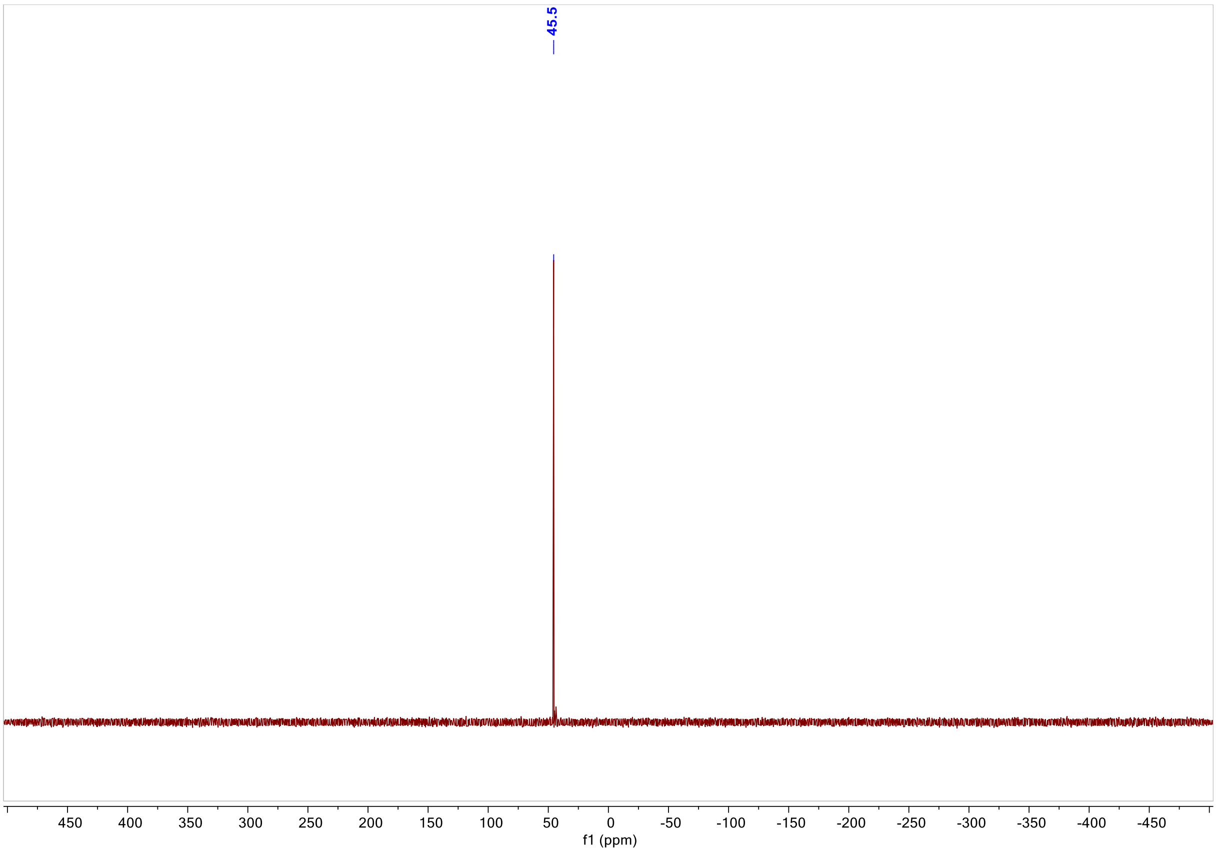

Figure S2.4 ^{31}P NMR spectrum of **3** in C_6D_6 at 300 K.

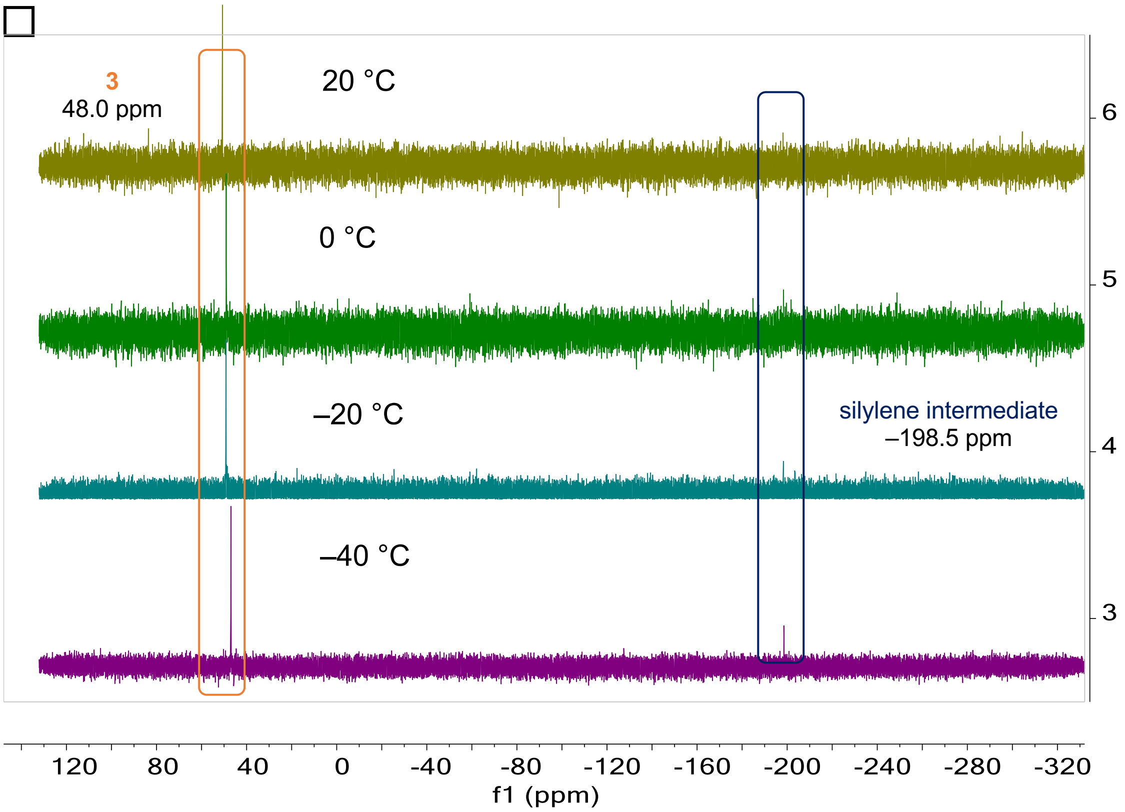

Figure S2.5 Stacked VT ^{31}P NMR spectrum (Tol-D_8) for **3** from –40 °C to 20 °C.

1.2.3 Synthesis of *P*-Silveriophosphasilene (4)

2*H*-phosphasilirene-3-olate **2** (96 mg, 0.1 mmol) and IPr·AgCl (53.2 mg, 0.1 mmol) were combined in benzene (2 mL) at room temperature. The color of mixture turned to orange from yellow rapidly with the release of CO. After work-up, all volatiles were removed *in vacuum* and the residue was extracted with pentane (3 × 3 mL). Pentane was removed *in vacuum* to yield *P*-silveriophosphasilene **4** (104.5 mg, 88%) as an orange power.

^{1}H NMR (400.1 MHz, C_6D_6): δ [ppm] 7.29-7.33 (m, 2H, Ar*H*), 7.23-7.24 (m, 6H, Ar*H*), 7.15-7.17 (m, 4H, Ar*H*, overlapping with C_6D_6), 6.38 (s, 2H, NC*H*), 3.78 (sept, *J* = 6.8 Hz, 4H, C*H*(CH_3)$_2$), 2.72 (sept, *J* = 6.8 Hz, 4H, C*H*(CH_3)$_2$), 1.69 (s, 6H, NCC*H*$_3$), 1.47 (d, *J* = 6.8 Hz, 12H, CH(C*H*$_3$)$_2$), 1.45 (d, *J* = 6.8 Hz, 12H, CH(C*H*$_3$)$_2$), 1.17 (s, 27H, C(C*H*$_3$)$_3$), 1.13 (d, *J* = 6.8 Hz, 12H, CH(C*H*$_3$)$_2$), 1.10 (d, *J* = 6.8 Hz, 12H, CH(C*H*$_3$)$_2$).

^{13}C{^{1}H} NMR (100.6 MHz, C_6D_6): δ [ppm] 151.9 (N*C*N), 148.8 (Ar*C*), 145.5 (Ar*C*), 135.7 (Ar*C*), 133.4 (Ar*C*), 129.9 (Ar*C*), 128.7 (Ar*C*), 124.1 (Ar*C*), 124.0 (Ar*C*), 122.4 (N*C*H), 118.0 (N*C*-CH_3), 32.5 (C(*C*H$_3$)$_3$), 28.6 (*C*H(CH_3)$_2$), 28.3 (*C*H(CH_3)$_2$), 28.2 (*C*H(CH_3)$_2$), 26.1 (*C*H(CH_3)$_2$), 26.0 (*C*H(CH_3)$_2$), 25.1 (CH(*C*H$_3$)$_2$), 24.4 (CH(*C*H$_3$)$_2$), 24.2 (CH(*C*H$_3$)$_2$), 24.0 (CH(*C*H$_3$)$_2$), 24.0 (*C*(CH_3)$_3$), 10.4 (NC-*C*H$_3$).

^{29}Si{^{1}H} NMR (79.5 MHz, C_6D_6): δ [ppm] 2.1 (d, $J_{Si–P}$ = 14.5 Hz, *Si*^{t}Bu$_3$), 123.2 (d, $J_{Si–P}$ = 179.6 Hz, *Si*=P).

^{31}P NMR (162.0 MHz, C_6D_6): δ [ppm] 40.2 (dd, ^{107}Ag: $J_{P–Ag}$ = 171.4 Hz and ^{109}Ag: $J_{P–Ag}$ = 196.2 Hz, Si=*P*).

LIFDI-MS: Calcd: 1183.6535; Found: 1183.6521.

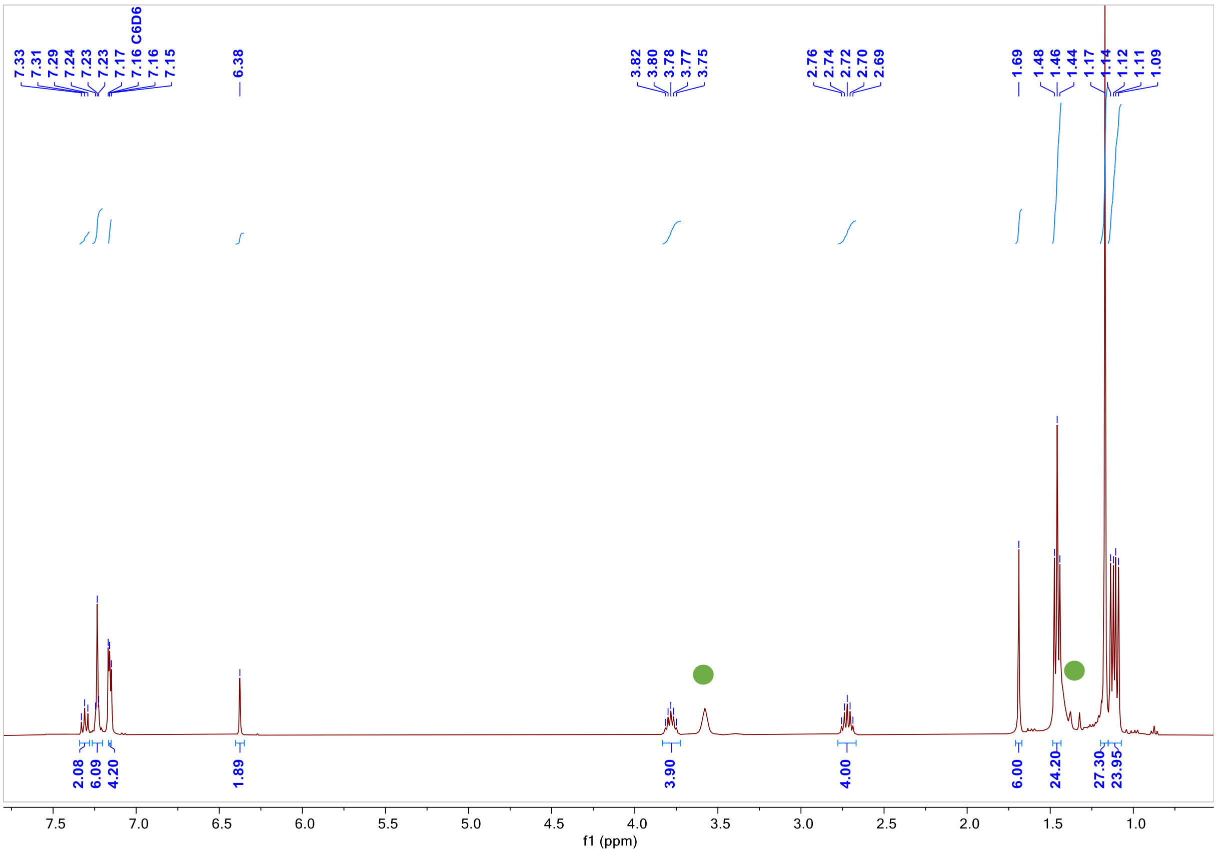

Figure S3.1 ^{1}H NMR spectrum of **4** in C_6D_6 at 300 K (●: THF).

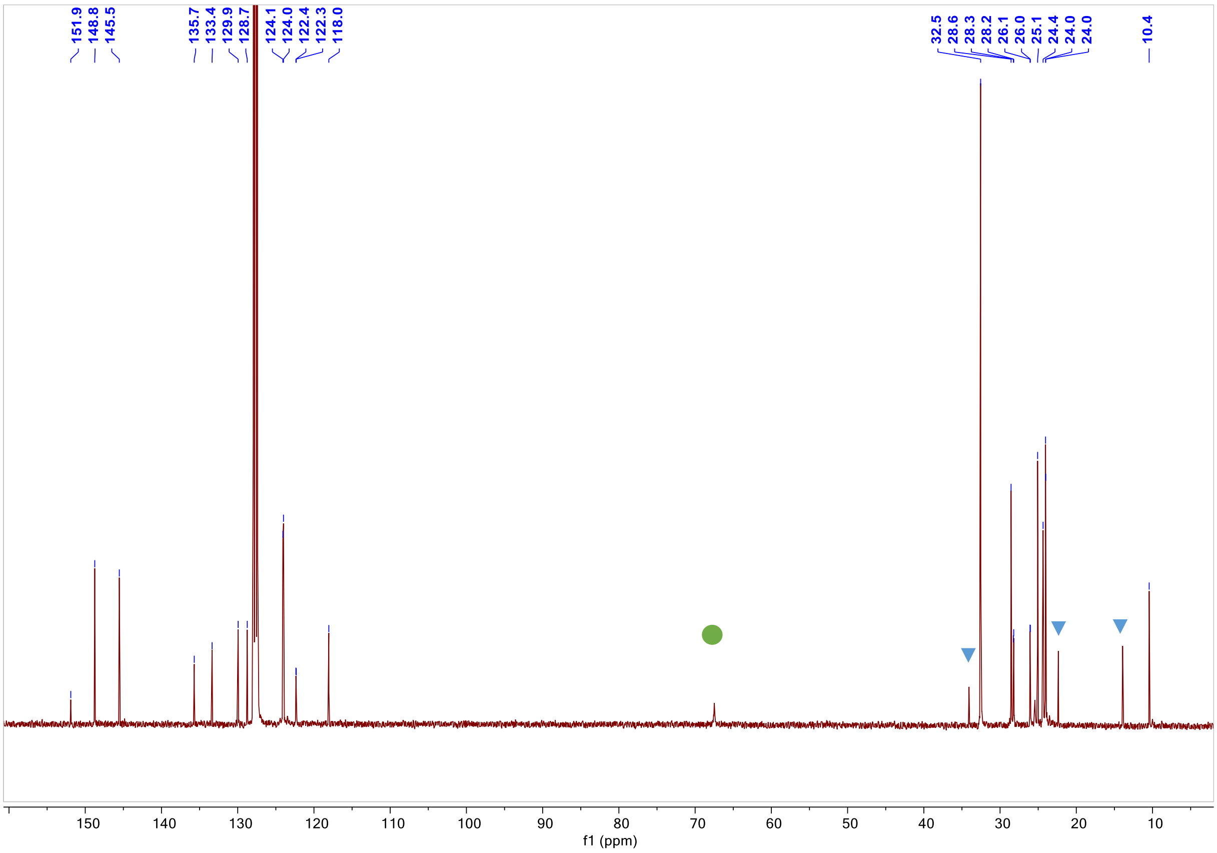

Figure S3.2 $^{13}C\{^{1}H\}$ NMR spectrum of **4** in C_6D_6 at 300 K (●: THF; ▼: pentane).

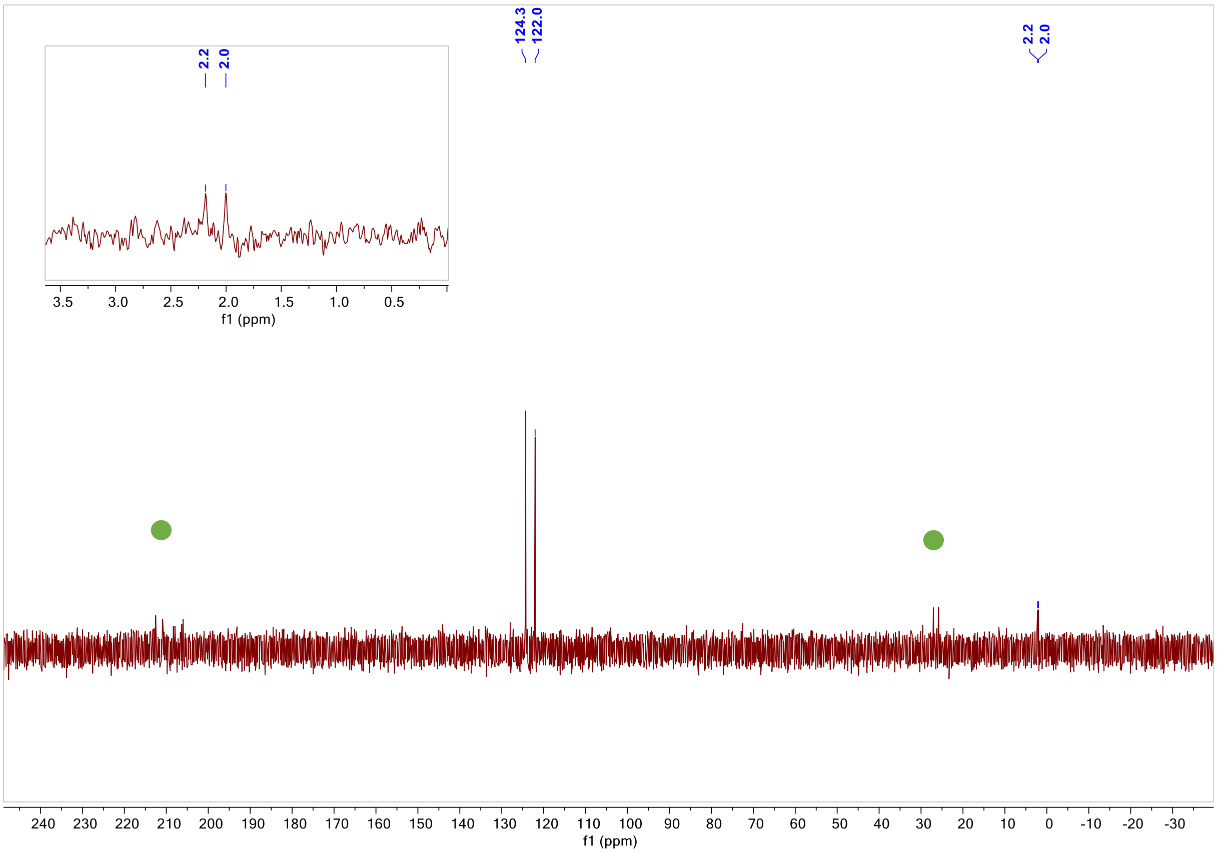

Figure S3.3 $^{29}Si\{^{1}H\}$ NMR spectrum of **4** in C_6D_6 at 300 K (●: *Si*-silveriophosphasilene **7**).

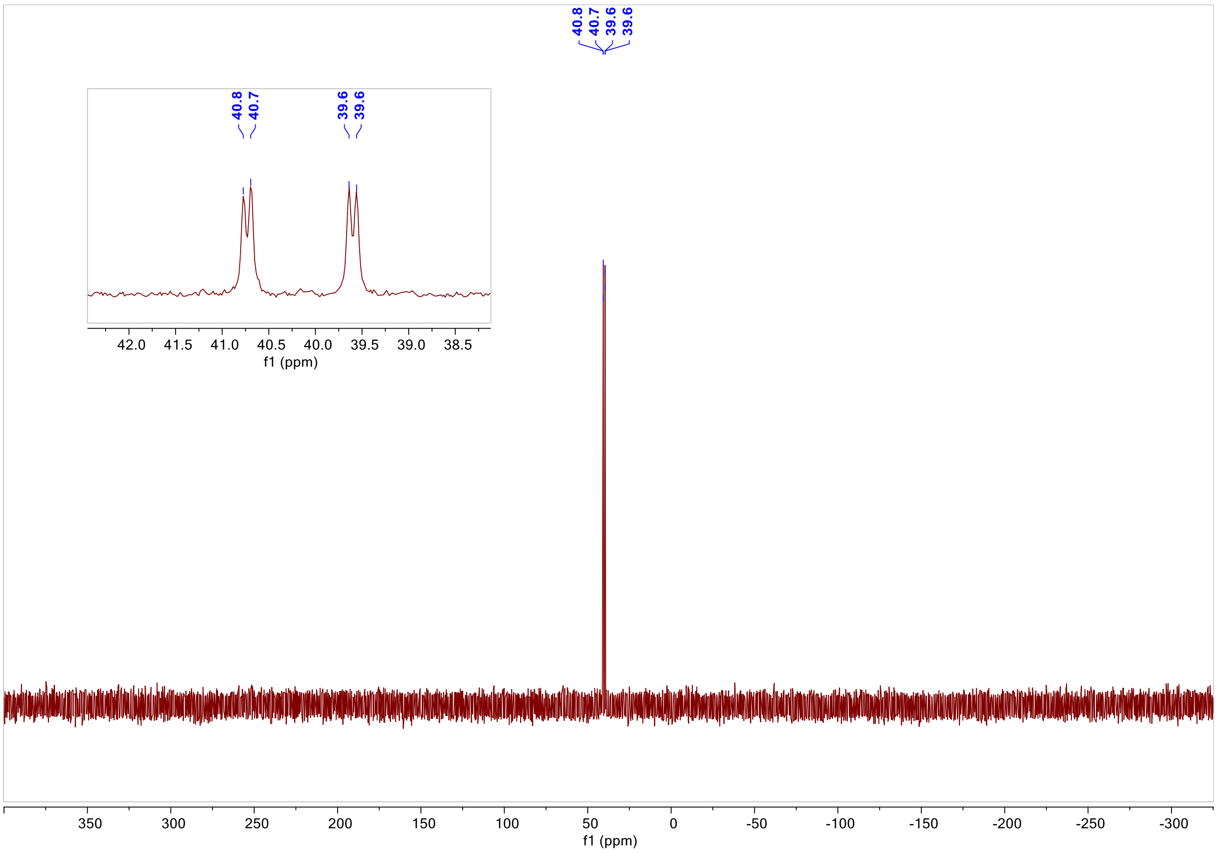

Figure S3.4 ^{31}P NMR spectrum of **4** in C_6D_6 at 300 K.

1.2.4 Synthesis of *P*-Goldiophosphasilene (5)

2*H*-phosphasilirene-3-olate **2** (96 mg, 0.1 mmol) and IPr·AuCl (62.1 mg, 0.1 mmol) were combined in benzene (2 mL) at room temperature. The color of mixture turned to deep orange from yellow rapidly with the release of CO. After work-up, all volatiles were removed *in vacuum* and the residue was extracted with pentane (3 × 3 mL). Pentane was removed *in vacuum* to yield *P*-goldiophosphasilene **5** (110.2 mg, 86%) as an orange power.

^{1}H NMR (400.1 MHz, C_6D_6): δ [ppm] 7.29-7.33 (m, 2H, Ar*H*), 7.18-7.20 (m, 6H, Ar*H*), 7.14-7.16 (m, 4H, Ar*H*, overlapping with C_6D_6), 6.37 (s, 2H, NC*H*), 3.64 (sept, *J* = 6.8 Hz, 4H, C*H*(CH_3)$_2$), 2.79 (sept, *J* = 6.8 Hz, 4H, C*H*(CH_3)$_2$), 1.65 (s, 6H, NCC*H*$_3$), 1.49 (d, *J* = 6.8 Hz, 12H, CH(C*H*$_3$)$_2$), 1.39 (d, *J* = 6.8 Hz, 12H, CH(C*H*$_3$)$_2$), 1.11 (d, *J* = 6.8 Hz, 12H, CH(C*H*$_3$)$_2$), 1.10 (s, 27H, C(C*H*$_3$)$_3$), 1.08 (d, *J* = 6.8 Hz, 12H, CH(C*H*$_3$)$_2$).

^{13}C{^{1}H} NMR (100.6 MHz, C_6D_6): δ [ppm] 202.5 (C_{NHC}, J_{C-P} = 44.4 Hz), 151.8 (N*C*N), 148.7 (Ar*C*), 145.6 (Ar*C*), 135.6 (Ar*C*), 133.1 (Ar*C*), 129.8 (Ar*C*), 128.8 (Ar*C*), 124.1 (Ar*C*), 123.9 (Ar*C*), 121.9 (N*C*H), 118.1 (N*C*-CH_3), 32.4 (C(*C*H$_3$)$_3$), 28.6 (*C*H(CH_3)$_2$), 28.2 (*C*H(CH_3)$_2$), 26.0 (*C*H(CH_3)$_2$), 25.9 (*C*H(CH_3)$_2$), 25.1 (CH(*C*H$_3$)$_2$), 24.1 (CH(*C*H$_3$)$_2$), 24.1 (CH(*C*H$_3$)$_2$), 24.0 (*C*(CH_3)$_3$), 10.4 (NC-*C*H$_3$).

^{29}Si{^{1}H} NMR (79.5 MHz, C_6D_6): δ [ppm] 6.5 (d, J_{Si-P} = 15.7 Hz, *Si*^{t}Bu$_3$), 139.2 (d, J_{Si-P} = 179.4 Hz, *Si*=P).

^{31}P NMR (162.0 MHz, C_6D_6): δ [ppm] 66.2 (Si=*P*).

LIFDI-MS: Calcd: 1273.7149; Found: 1273.7139.

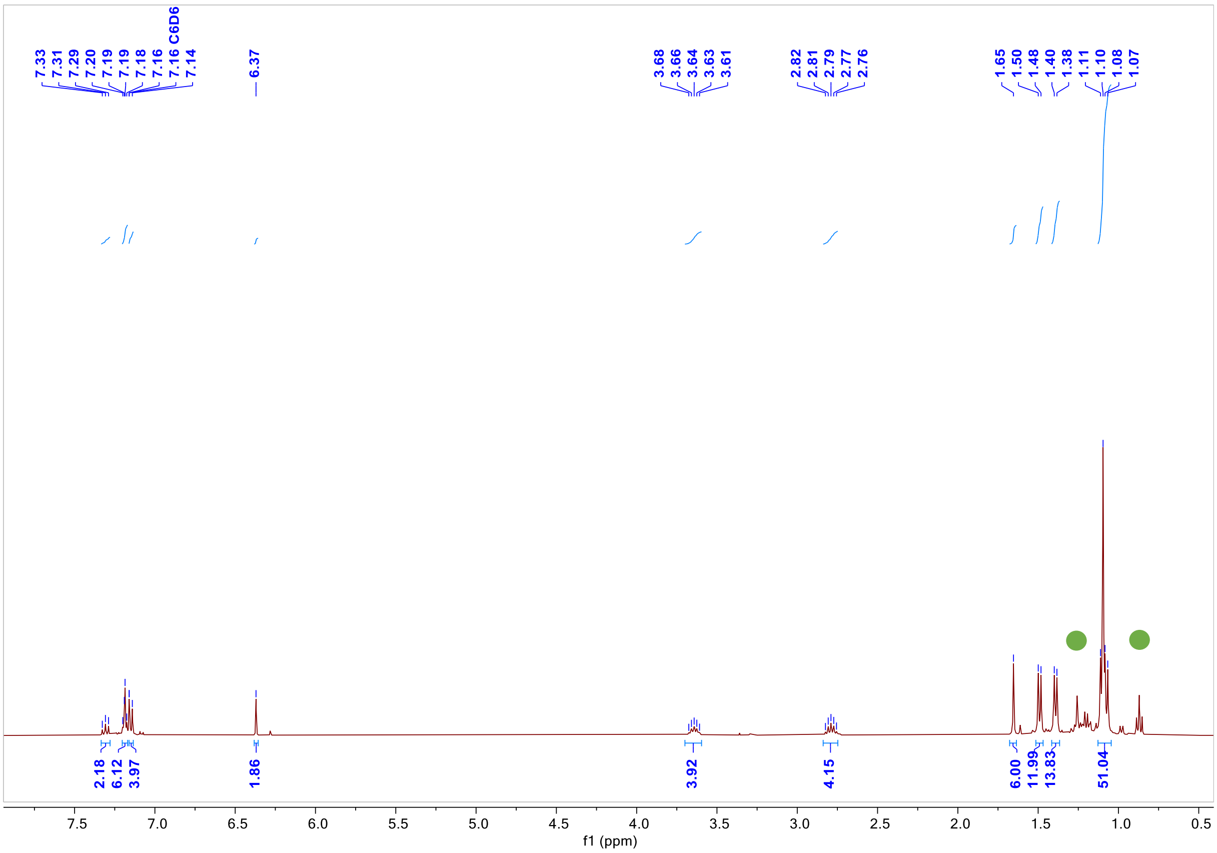

Figure S4.1 ^{1}H NMR spectrum of **5** in C_6D_6 at 300 K (●: pentane).

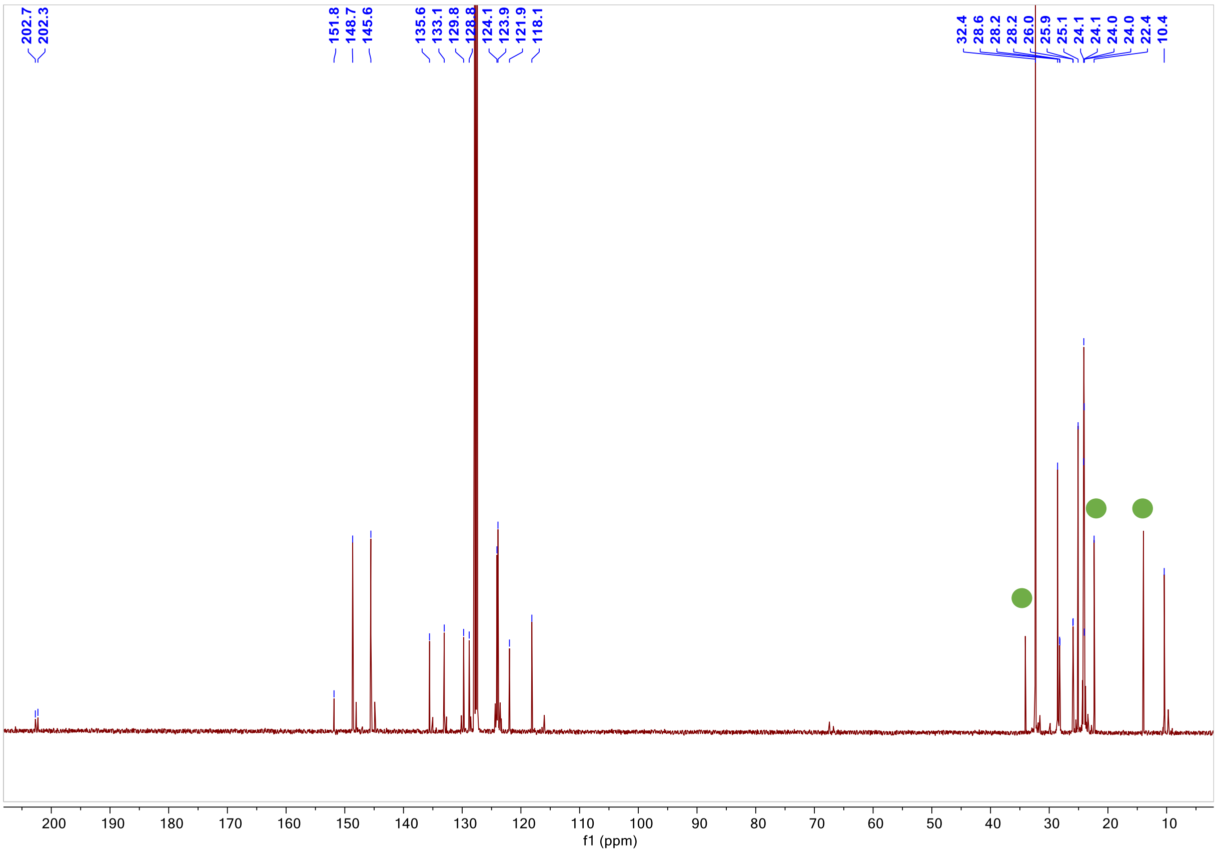

Figure S4.2 $^{13}C\{^{1}H\}$ NMR spectrum of **5** in C_6D_6 at 300 K (●: pentane).

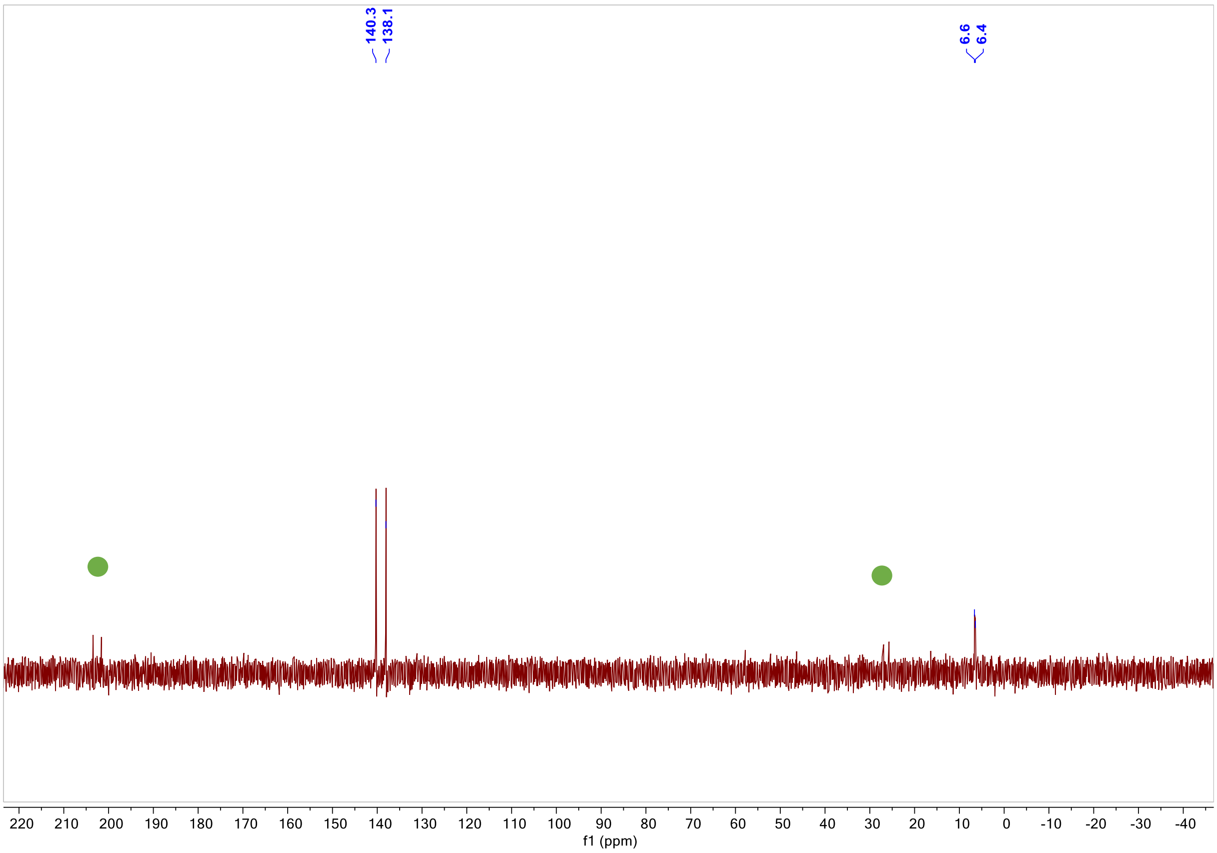

Figure S4.3 $^{29}Si\{^{1}H\}$ NMR spectrum of **5** in C_6D_6 at 300 K (●: *Si*-goldiophosphasilene **8**).

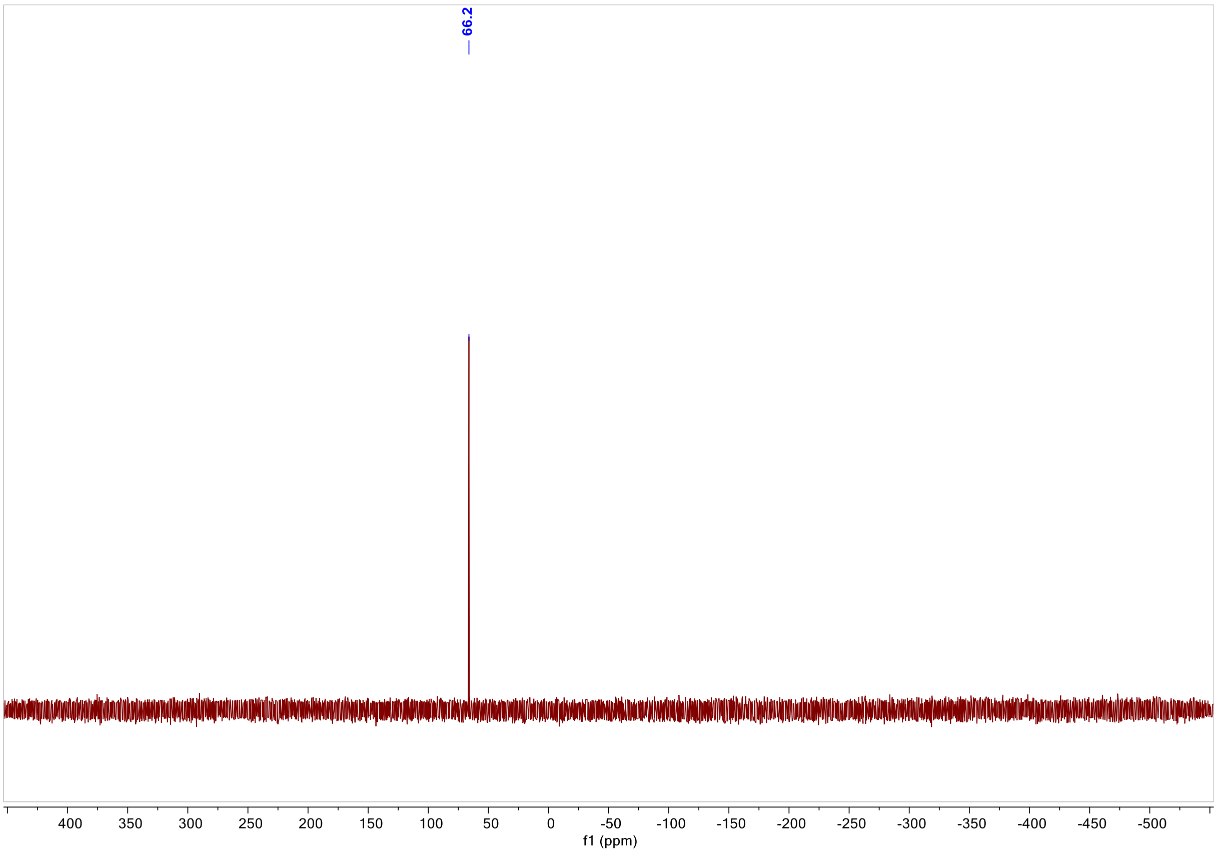

Figure S4.4 ^{31}P NMR spectrum of **5** in C_6D_6 at 300 K.

1.2.5 Synthesis of *P*-Goldiophosphasilene (6)

2*H*-phosphasilirene-3-olate **2** (96 mg, 0.1 mmol) and ^{Me}CAAC·AuCl (51.8 mg, 0.1 mmol) were combined in benzene (2 mL) at room temperature. The color of mixture turned to sanguine from yellow rapidly with the release of CO. After work-up, all volatiles were removed *in vacuum* and the residue was extracted with Et_2O (2 × 3 mL). The crude red crystals were obtained by the rapid evaporation of saturated Et_2O solution at room temperature and then washed with pentane (1 × 3 mL). All volatiles were removed *in vacuum* to yield *P*-goldiophosphasilene **6** (50.2 mg, 43%) as a red powder.

Note: Due to the poor solubility of *P*-goldiophosphasilene **6**, we couldn't get the ^{13}C and ^{29}Si NMR spectra.

^{1}H NMR (400.1 MHz, C_6D_6): δ [ppm] 7.21-7.29 (m, 7H, Ar*H*), 7.11-7.13 (m, 2H, Ar*H*), 3.67 (sept, *J* = 6.8 Hz, 4H, C*H*(CH_3)$_2$), 2.80 (sept, *J* = 6.8 Hz, 2H, C*H*(CH_3)$_2$), 1.67 (s, 6H, NCC*H*$_3$), 1.49 (d, *J* = 6.8 Hz, 12H, CH(C*H*$_3$)$_2$), 1.45 (s, 6H, C(C*H*$_3$)$_2$), 1.38-1.42 (m, *33*H, C(C*H*$_3$)$_3$ and CH(C*H*$_3$)$_2$), 1.29 (s, 2H, C*H*$_2$),1.19 (d, *J* = 6.8 Hz, 6H, CH(C*H*$_3$)$_2$), 1.11 (d, *J* = 6.8 Hz, 12H, CH(C*H*$_3$)$_2$), 0.88 (s, 6H, C(C*H*$_3$)$_2$).

^{31}P NMR (162.0 MHz, C_6D_6): δ [ppm] 61.9 (Si=*P*).

LIFDI-MS: Calcd: 1170.6727; Found: 1170.6704.

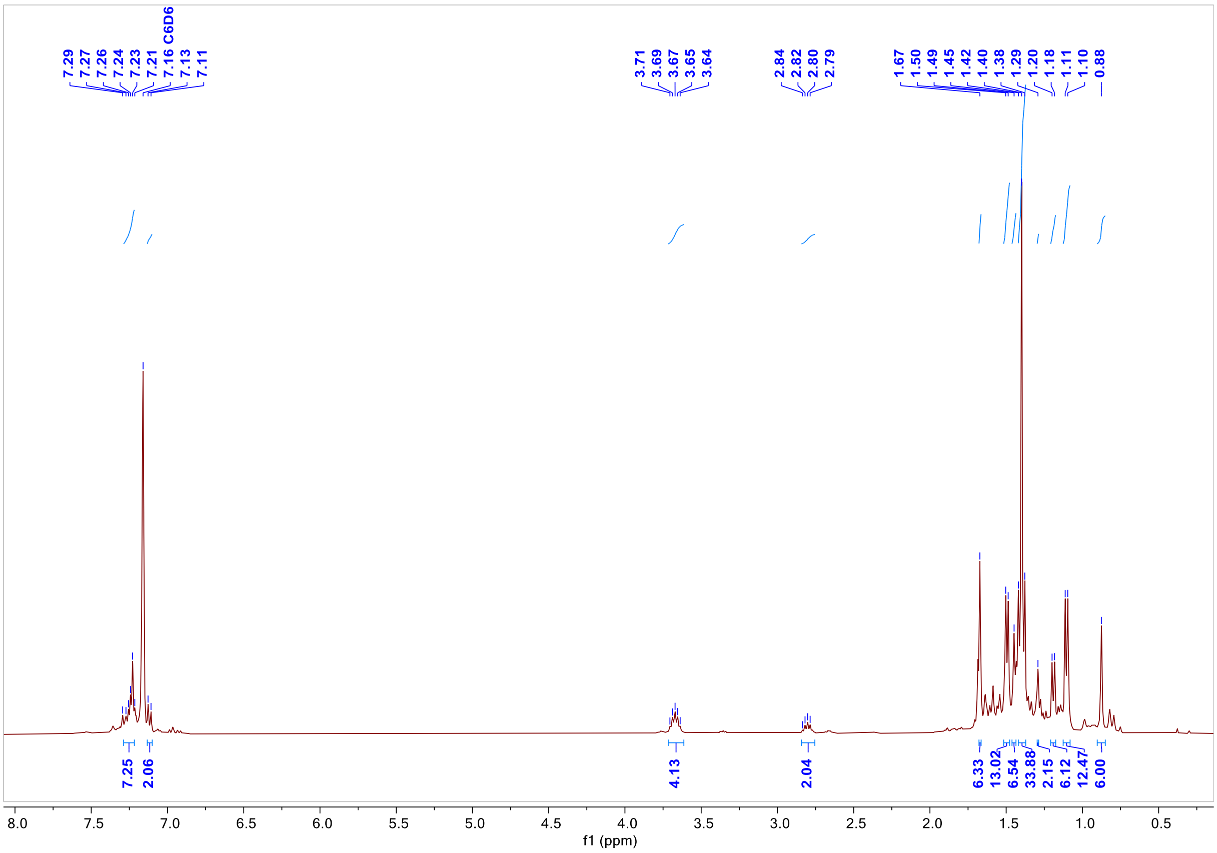

Figure S5.1 ^{1}H NMR spectrum of **6** in C_6D_6 at 300 K.

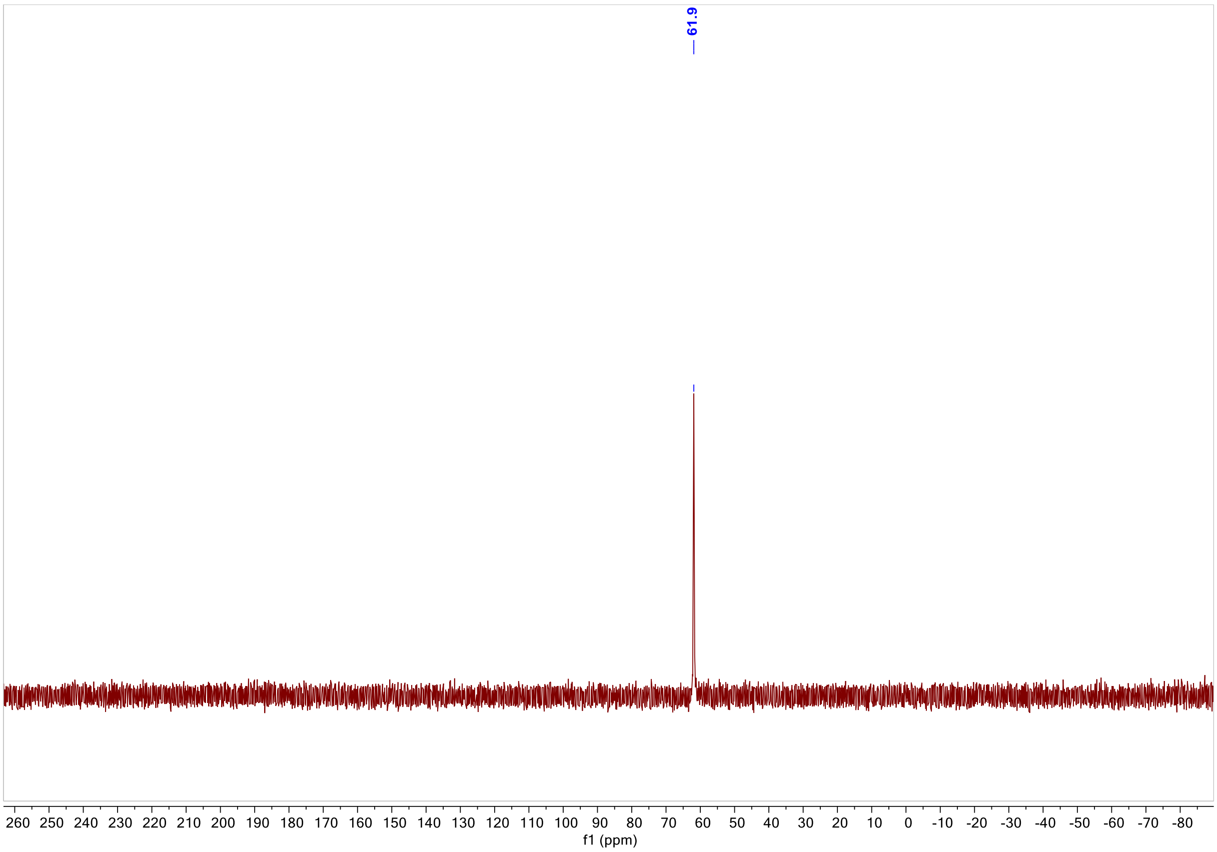

Figure S5.2 ^{31}P NMR spectrum of **6** in C_6D_6 at 300 K.

1.2.6 Synthesis of *Si*-Silveriophosphasilene (7)

A benzene solution of *P*-silveriophosphasilene **4** (59.3 mg, 0.05 mmol) was allowed to stir at room temperature for 24 h. The color of mixture turned to yellow from orange. After work-up, all volatiles were removed *in vacuum* to yield *Si*-silveriophosphasilene **7** (58.5 mg, 98%) as a yellow power. Crystal suitable for single crystal X-ray diffraction analysis was obtained by storing a saturated pentane solution at –30 °C for 2 days.

^{1}H NMR (400.1 MHz, C_6D_6): δ [ppm] 7.20-7.29 (m, 8H, Ar*H*), 7.07-7.09 (m, 4H, Ar*H*), 6.29 (s, 2H, NC*H*), 3.39 (sept, *J* = 6.8 Hz, 4H, C*H*(CH_3)$_2$), 2.68 (sept, *J* = 6.8 Hz, 4H, C*H*(CH_3)$_2$), 1.63 (s, 6H, NCC*H*$_3$), 1.43 (d, *J* = 6.8 Hz, 12H, CH(C*H*$_3$)$_2$), 1.31 (s, 27H, C(C*H*$_3$)$_3$), 1.24 (d, *J* = 6.8 Hz, 12H, CH(C*H*$_3$)$_2$), 1.19 (d, *J* = 6.8 Hz, 12H, CH(C*H*$_3$)$_2$), 0.99 (d, *J* = 6.8 Hz, 12H, CH(C*H*$_3$)$_2$).

^{13}C{^{1}H} NMR (100.6 MHz, C_6D_6): δ [ppm] 192.2 (d, ^{107}Ag: $J_{C–Ag}$ = 120.6 Hz and ^{109}Ag: $J_{C–Ag}$ = 139.23 Hz, C_{NHC}), 148.2 (Ar*C*), 144.8 (Ar*C*), 135.4 (Ar*C*), 132.9 (Ar*C*), 130.2 (Ar*C*), 128.5 (Ar*C*), 124.4 (Ar*C*), 123.5 (Ar*C*), 123.3 (N*C*H), 115.9 (N*C*-CH_3), 32.5 (C(*C*H$_3$)$_3$), 28.5 (*C*H(CH_3)$_2$), 28.3 (*C*H(CH_3)$_2$), 25.2 (*C*H(CH_3)$_2$), 25.1 (*C*H(CH_3)$_2$), 24.2 (*C*(CH_3)$_3$), 24.0 (CH(*C*H$_3$)$_2$), 23.9 (CH(*C*H$_3$)$_2$), 23.8 (CH(*C*H$_3$)$_2$), 23.7 (CH(*C*H$_3$)$_2$), 9.8 (NC-*C*H$_3$).

^{29}Si{^{1}H} NMR (79.5 MHz, C_6D_6): δ [ppm] 26.5 (d, $J_{Si–P}$ = 97.5 Hz, *Si*^{t}Bu$_3$), 209.4 (dddd, $J_{Si–P}$ = 160.4 Hz, ^{107}Ag: $J_{Si–Ag}$ = 338.0 Hz and ^{109}Ag: $J_{Si–Ag}$ = 390.4 Hz, *Si*=P).

^{31}P NMR (162.0 MHz, C_6D_6): δ [ppm] –15.9 (d, $^{107/109}$Ag: $J_{P–Ag}$ = 19.7 Hz, Si=*P*).

LIFDI-MS: Calcd: 1183.6535; Found: 1183.6543.

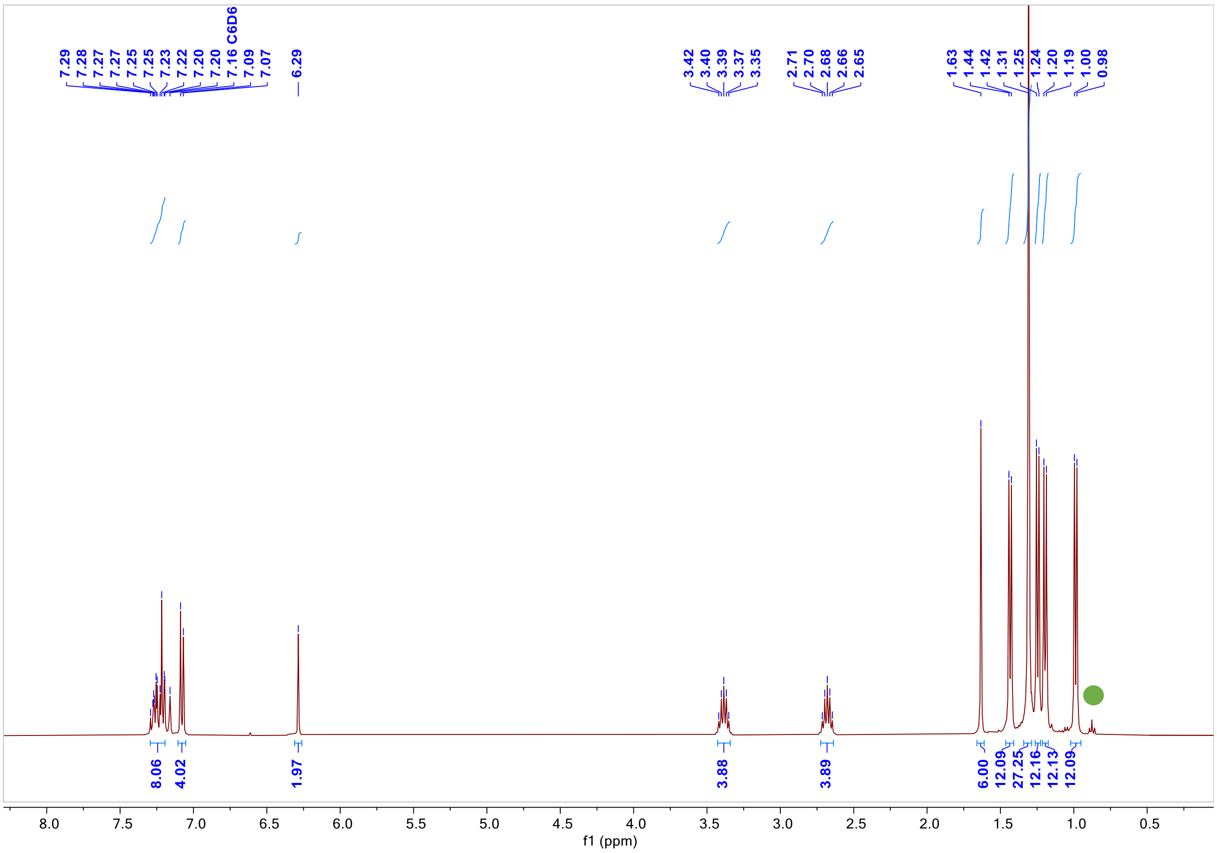

Figure S6.1 ^{1}H NMR spectrum of **7** in C_6D_6 at 300 K (●: pentane).

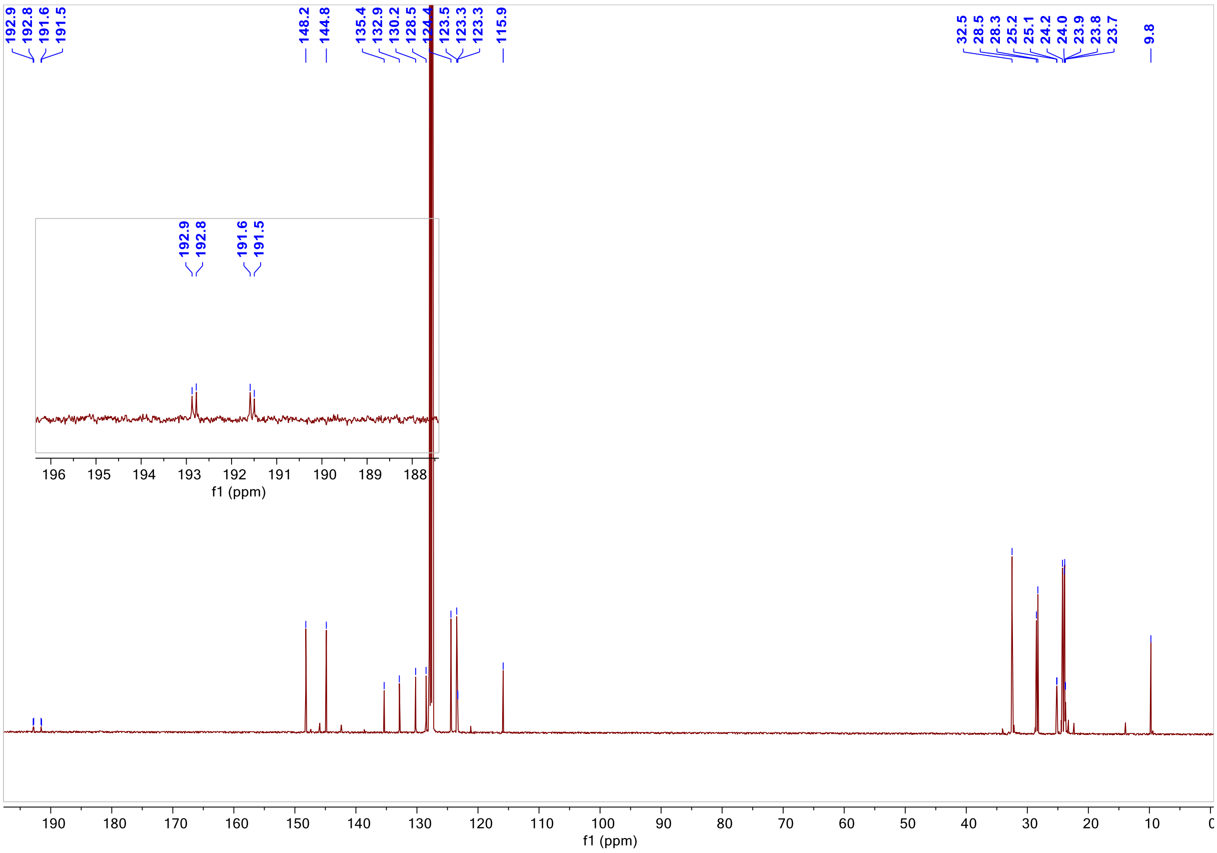

Figure S6.2 $^{13}C\{^{1}H\}$ NMR spectrum of **7** in C_6D_6 at 300 K.

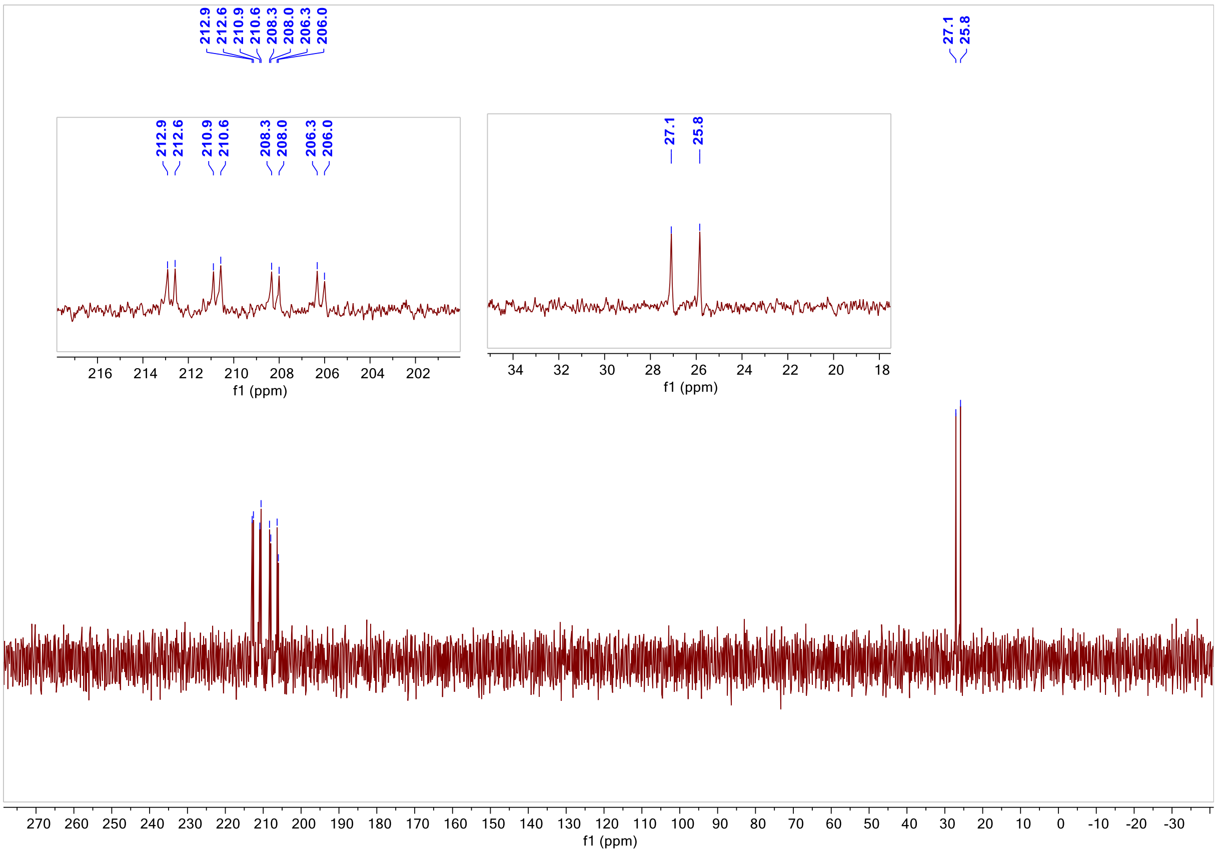

Figure S6.3 $^{29}Si\{^1H\}$ NMR spectrum of **7** in C_6D_6 at 300 K.

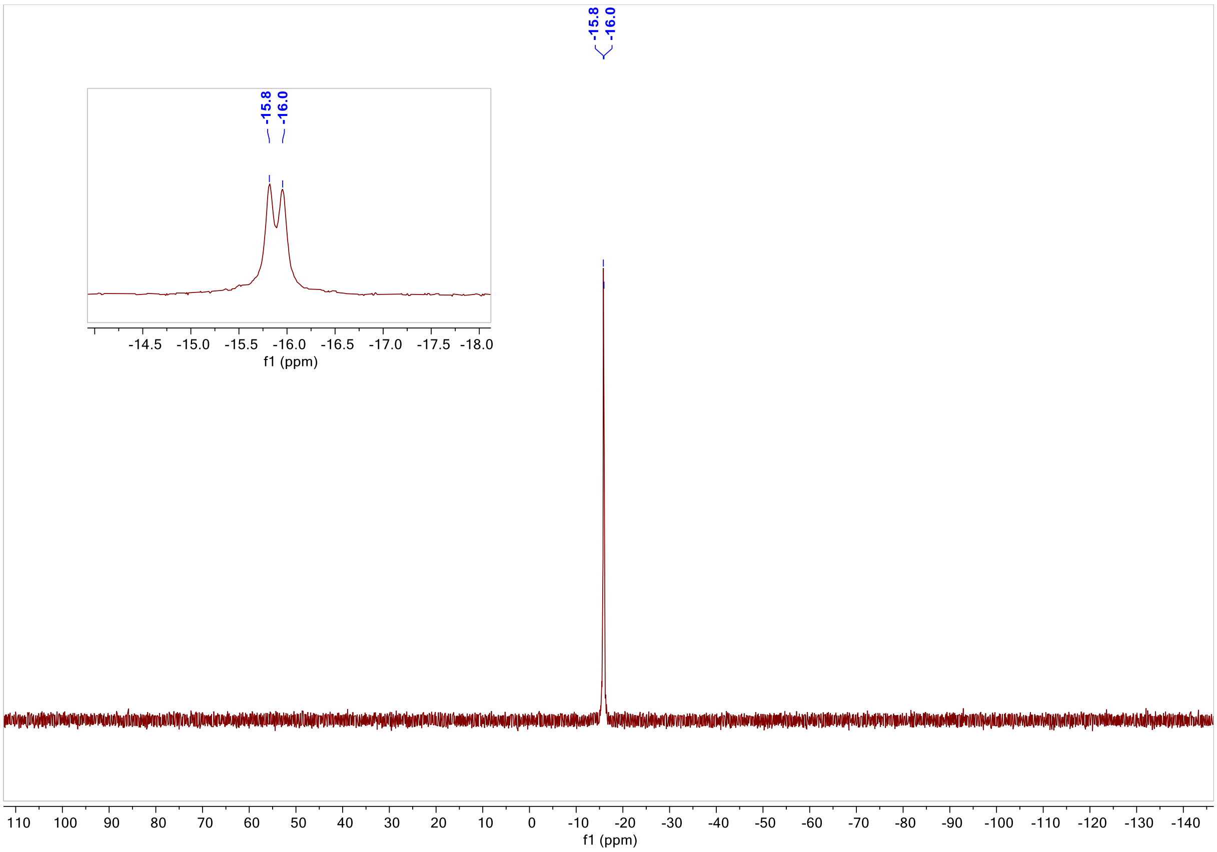

Figure S6.4 ^{31}P NMR spectrum of **7** in C_6D_6 at 300 K.

1.2.7 Synthesis of *Si*-Goldiophosphasilene (8)

A benzene solution of *P*-goldiophosphasilene **5** (68.7 mg, 0.05 mmol) was allowed to stir at room temperature for 24 h. The color of mixture turned to yellow from orange. After work-up, all volatiles were removed *in vacuum* to yield *Si*-goldiophosphasilene **8** (67.6 mg 99%) as a yellow power. Crystal suitable for single crystal X-ray diffraction analysis was obtained by storing a saturated pentane solution at –30 °C for 24 h.

^{1}H NMR (400.1 MHz, C_6D_6): δ [ppm] 7.24-7.28 (m, 4H, Ar*H*), 7.18-7.20 (m, 4H, Ar*H*), 7.08-7.10 (m, 4H, Ar*H*), 6.30 (s, 2H, NC*H*), 3.30 (sept, *J* = 6.8 Hz, 4H, C*H*(CH_3)$_2$), 2.76 (sept, *J* = 6.8 Hz, 4H, C*H*(CH_3)$_2$), 1.62 (s, 6H, NCC*H*$_3$), 1.39 (d, *J* = 6.8 Hz, 12H, CH(C*H*$_3$)$_2$), 1.26 (s, 27H, C(C*H*$_3$)$_3$), 1.21 (d, *J* = 6.8 Hz, 24H, CH(C*H*$_3$)$_2$), 0.99 (d, *J* = 6.8 Hz, 12H, CH(C*H*$_3$)$_2$).

^{13}C{^{1}H} NMR (100.6 MHz, C_6D_6): δ [ppm] 206.2 (C_{NHC}), 148.1 (Ar*C*), 144.9 (Ar*C*), 143.0 (N*C*N), 135.0 (Ar*C*), 132.7 (Ar*C*), 130.2 (Ar*C*), 128.6 (Ar*C*), 124.4 (Ar*C*), 123.6 (Ar*C*), 123.3 (N*C*H), 116.1 (N*C*-CH_3), 32.4 (C(*C*H$_3$)$_3$), 28.5 (*C*H(CH_3)$_2$), 28.3 (*C*H(CH_3)$_2$), 25.2 (*C*H(CH_3)$_2$), 25.1 (*C*H(CH_3)$_2$), 24.3 (*C*(CH_3)$_3$), 24.0 (CH(*C*H$_3$)$_2$), 23.8 (CH(*C*H$_3$)$_2$), 23.5 (CH(*C*H$_3$)$_2$), 23.4 (CH(*C*H$_3$)$_2$), 9.7 (NC-*C*H$_3$).

^{29}Si{^{1}H} NMR (79.5 MHz, C_6D_6): δ [ppm] 26.3 (d, $J_{Si\text{–}P}$ = 96.5 Hz, *Si*^{t}Bu$_3$), 202.7 (d, $J_{Si\text{–}P}$ = 148.8 Hz, *Si*=P).

^{31}P NMR (162.0 MHz, C_6D_6): δ [ppm] –62.2 (Si=*P*).

LIFDI-MS: Calcd: 1273.7149; Found: 1273.7142.

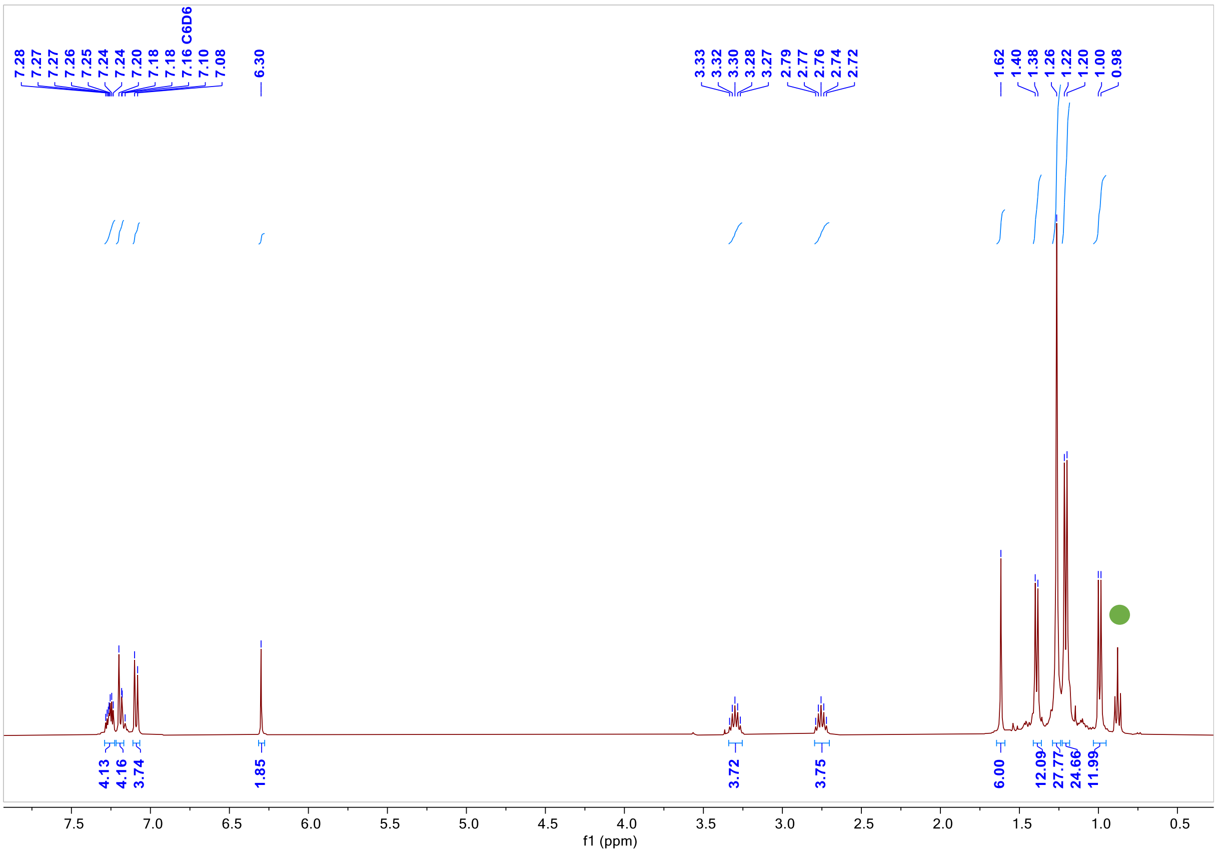

Figure S7.1 ^{1}H NMR spectrum of **8** in C_6D_6 at 300 K (●: pentane).

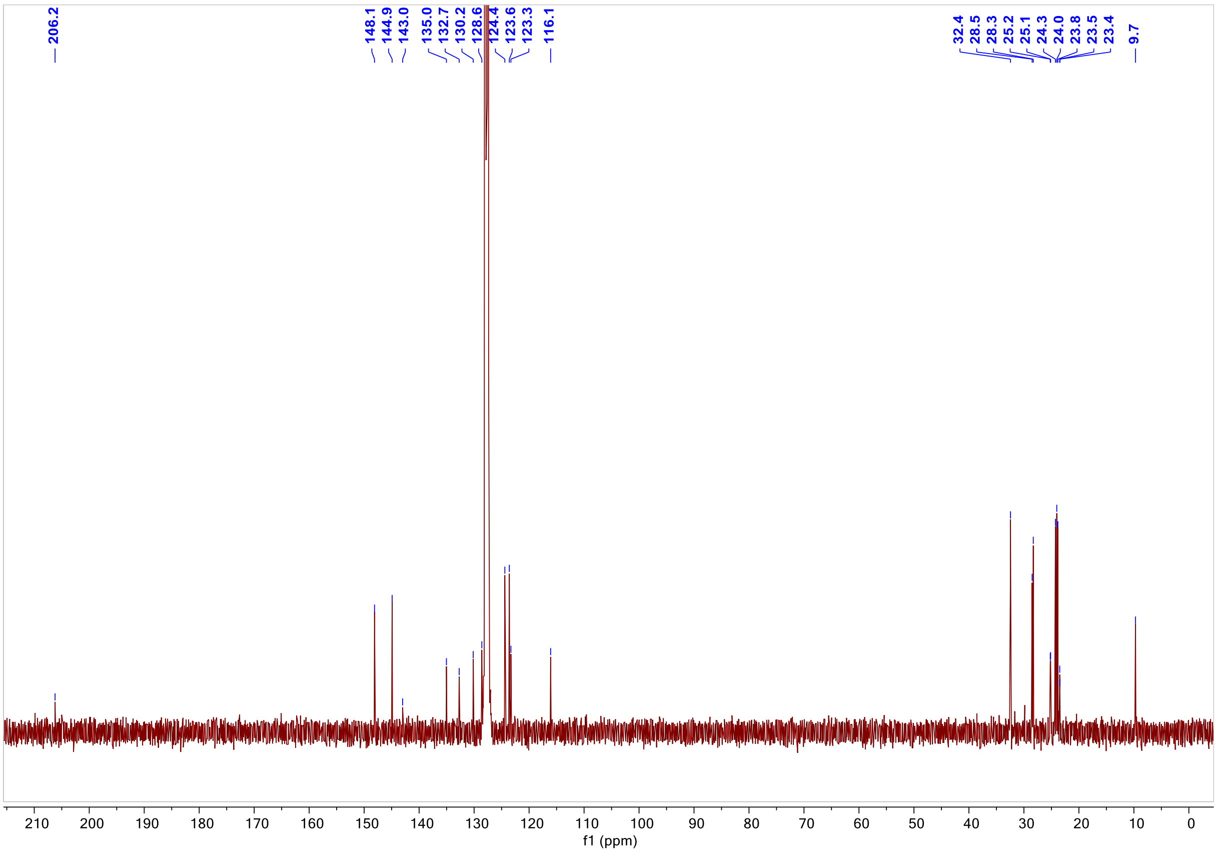

Figure S7.2 $^{13}C\{^{1}H\}$ NMR spectrum of **8** in C_6D_6 at 300 K.

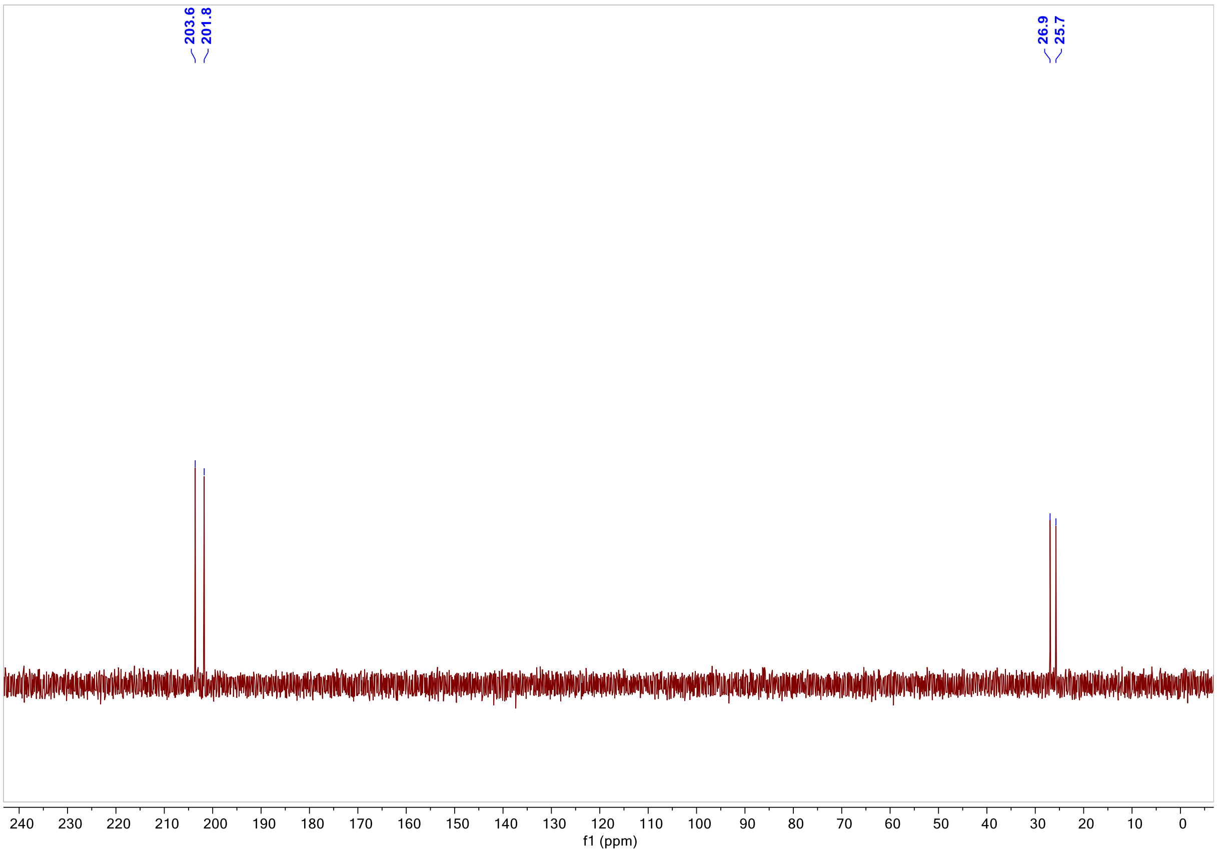

Figure S7.3 $^{29}Si\{^1H\}$ NMR spectrum of **8** in C_6D_6 at 300 K.

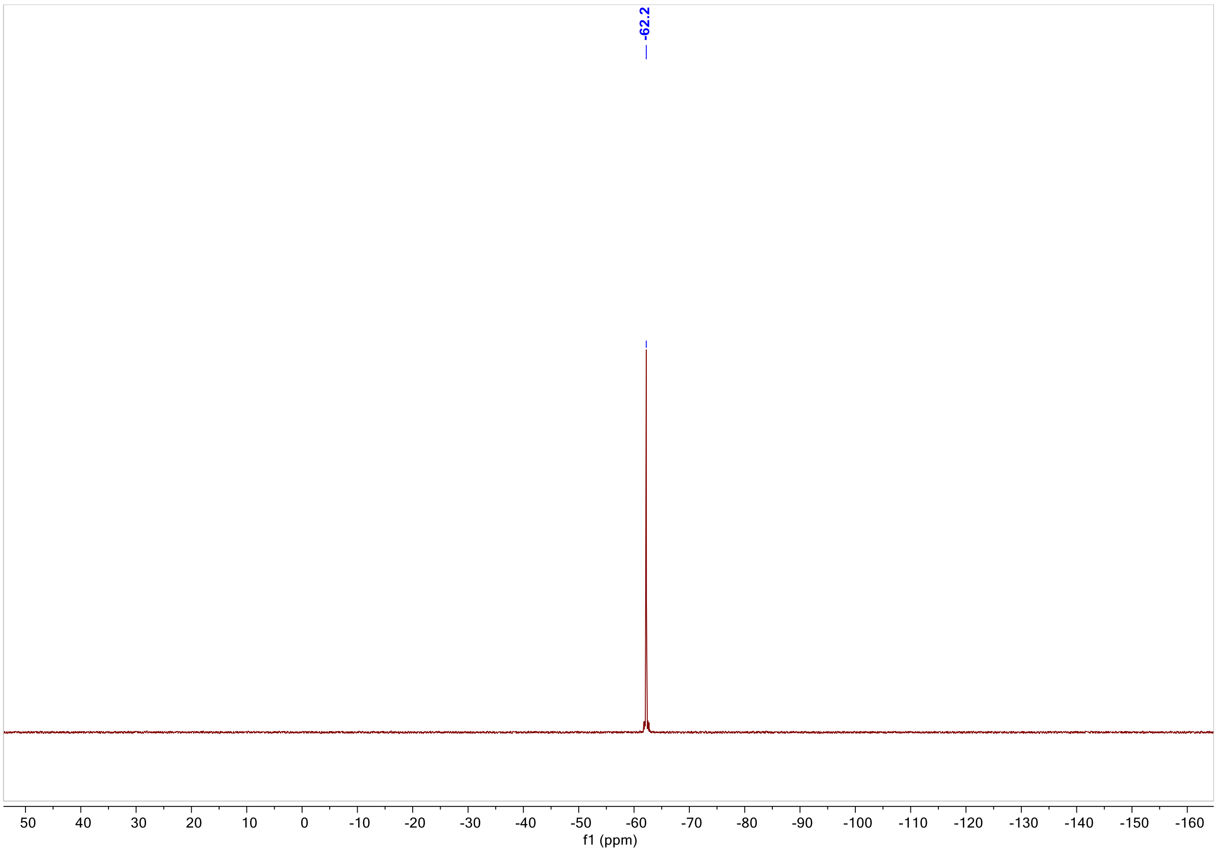

Figure S7.4 ^{31}P NMR spectrum of **8** in C_6D_6 at 300 K.

1.2.8 Synthesis of *Si*-Goldiophosphasilene (9)

A benzene suspension of *P*-goldiophosphasilene **6** (23.4 mg, 0.02 mmol) was allowed to stir at room temperature for 24 h. The color of mixture turned to orange from red. After work-up, all volatiles were removed *in vacuum* to yield *Si*-goldiophosphasilene **9** (22.6 mg, 97%) as an orange power.

^{1}H NMR (400.1 MHz, C_6D_6): δ [ppm] 7.23-7.36 (m, 7H, Ar*H*), 6.96-6.98 (m, 2H, Ar*H*), 3.45 (sept, *J* = 6.8 Hz, 4H, C*H*(CH_3)$_2$), 2.52 (sept, *J* = 6.8 Hz, 2H, C*H*(CH_3)$_2$), 1.61 (m, 12H, CH(CH_3)$_2$ and NCCH_3), 1.43 (s, 27H, C(CH_3)$_3$), 1.29 (s, 6H, C(CH_3)$_2$), 1.21-1.26 (d, *24*H, CH(CH_3)$_2$), 1.14 (s, 2H, CH_2), 1.11 (d, *J* = 6.8 Hz, 6H, CH(CH_3)$_2$), 0.75 (s, 6H, C(CH_3)$_2$).

^{13}C{^{1}H} NMR (100.6 MHz, C_6D_6): δ [ppm] 148.2 (N*C*N), 144.8 (Ar*C*), 133.5 (Ar*C*), 132.8 (Ar*C*), 129.5 (Ar*C*), 128.9 (Ar*C*), 128.2 (Ar*C*), 124.6 (Ar*C*), 124.2 (Ar*C*), 123.7 (Ar*C*), 116.2 (N*C*-CH_3), 81.0 (C(*C*H_3)$_2$), 54.9 (C(*C*H_3)$_2$), 49.8 (*C*H_2), 32.6 (C(*C*H_3)$_3$), 31.9 (C(*C*H_3)$_2$), 29.9 (C(*C*H_3)$_2$), 28.9 (*C*H(CH_3)$_2$), 28.7 (*C*H(CH_3)$_2$), 28.6 (*C*H(CH_3)$_2$), 28.2 (*C*H(CH_3)$_2$), 27.5 (*C*H(CH_3)$_2$), 25.7 (CH(*C*H_3)$_2$), 25.6 (CH(*C*H_3)$_2$), 23.9 (*C*(CH_3)$_3$), 23.7 (CH(*C*H_3)$_2$), 23.6 (CH(*C*H_3)$_2$), 23.2 (CH(*C*H_3)$_2$), 22.4 (CH(*C*H_3)$_2$), 9.7 (NC-*C*H_3).

^{29}Si{^{1}H} NMR (79.5 MHz, C_6D_6): δ [ppm] 26.9 (d, $J_{Si–P}$ = 97.0 Hz, *Si*^{t}Bu$_3$), 208.7 (d, $J_{Si–P}$ = 151.2 Hz, *Si*=P).

^{31}P NMR (162.0 MHz, C_6D_6): δ [ppm] –56.8 (Si=*P*).

LIFDI-MS: Calcd: 1170.6727; Found: 1170.6763.

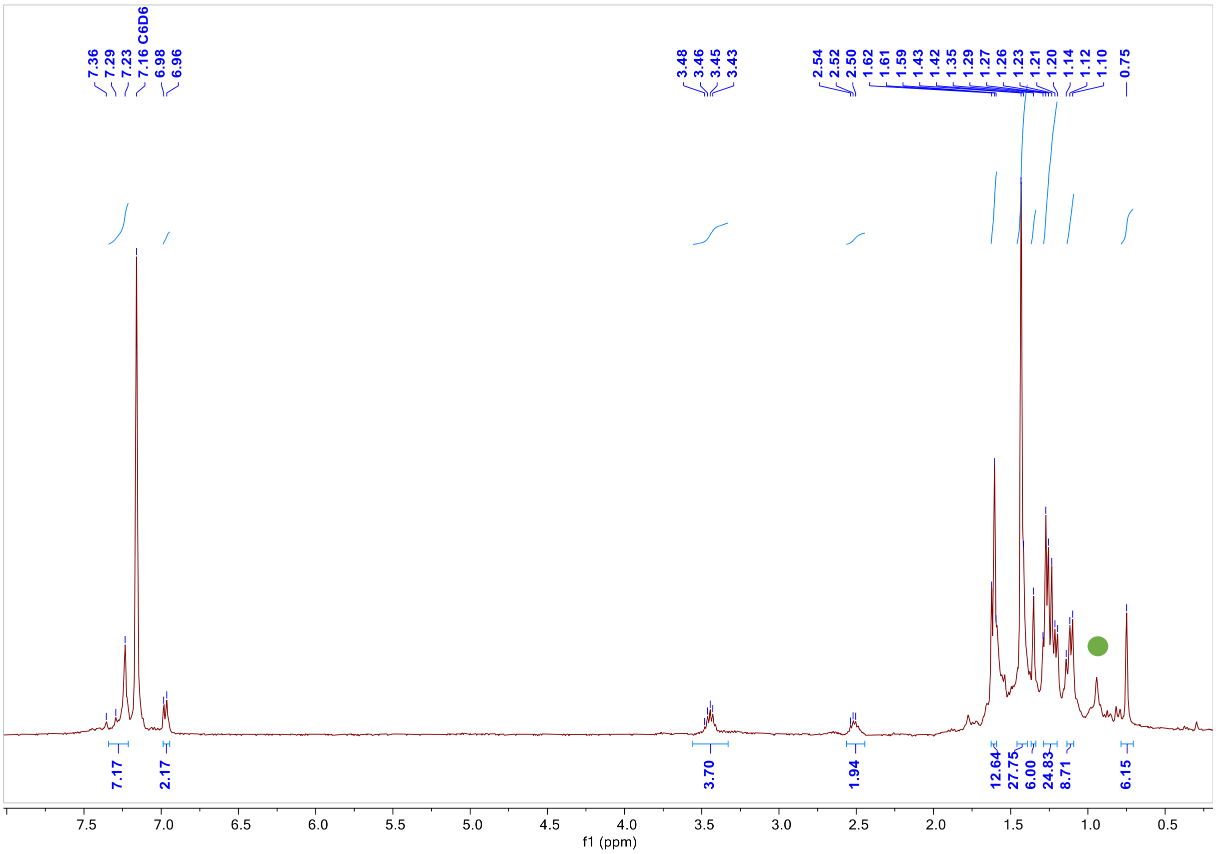

Figure S8.1 ^{1}H NMR spectrum of **9** in C_6D_6 at 300 K (●: pentane).

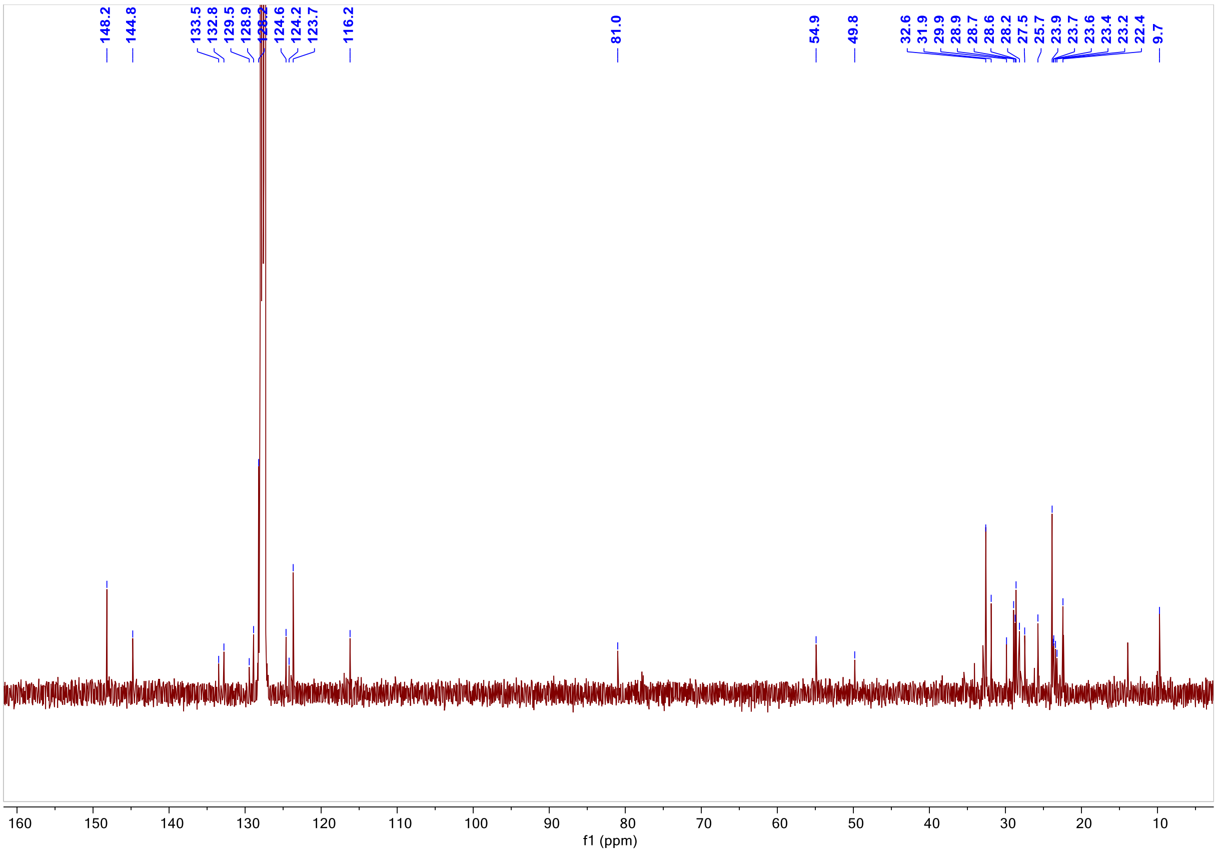

Figure S8.2 $^{13}C\{^{1}H\}$ NMR spectrum of **9** in C_6D_6 at 300 K.

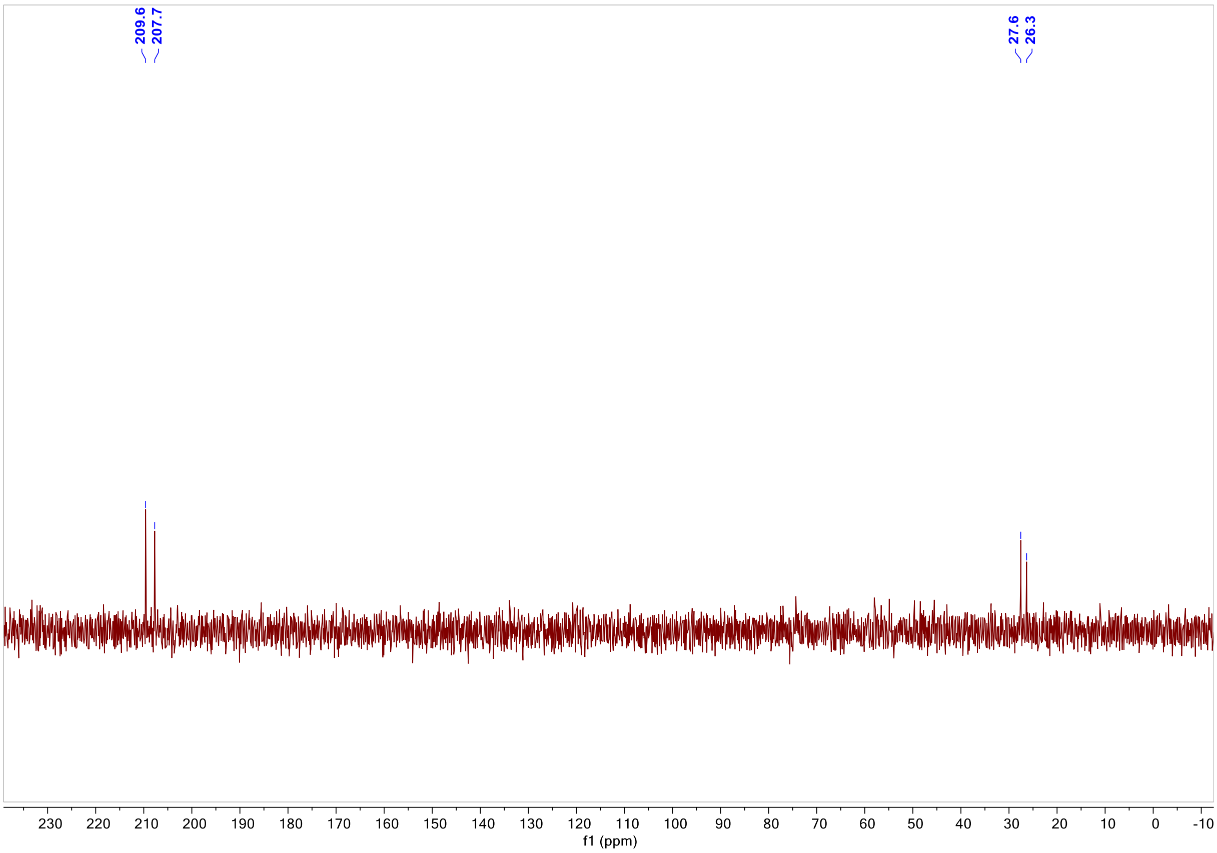

Figure S8.3 $^{29}Si\{^{1}H\}$ NMR spectrum of **9** in C_6D_6 at 300 K.

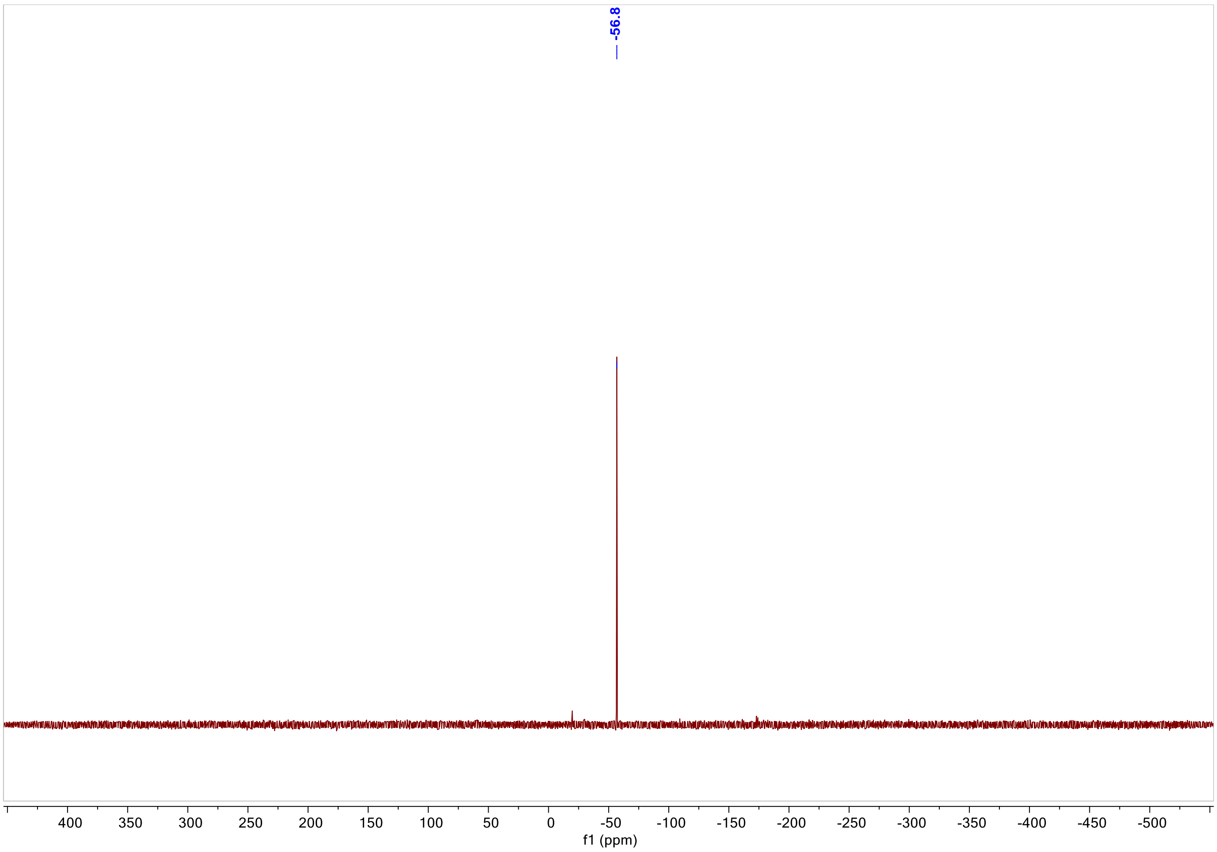

Figure S8.4 ^{31}P NMR spectrum of **9** in C_6D_6 at 300 K.

1.2.9 Synthesis of Carbene-iron complex (10)

Iron carbonyl (4.0 mg, 0.02 mmol) was added to a benzene solution of *P*-silveriophosphasilene **4** (23.7 mg, 0.02 mmol) at room temperature. The color of mixture turned deep orange rapidly. After removing all volatiles *in vacuum*, the orange residue was washed with cold pentane and dried *in vacuum* to yield carbene-iron complex **10** (25.6 mg, 94%) as a bright yellow powder. Yellow crystals suitable single crystal X-ray diffraction analysis was obtained by slow diffusion of pentane into saturated toluene solution at –30 °C for 2 days.

^{1}H NMR (400.1 MHz, C_6D_6): δ [ppm] 7.17-7.37 (m, 12H, Ar*H*, overlapping with C_6D_6), 6.29 (s, 2H, NC*H*), 3.37 (sept, *J* = 6.8 Hz, 2H, C*H*(CH_3)$_2$), 3.07 (sept, *J* = 6.8 Hz, 2H, C*H*(CH_3)$_2$), 2.57 (sept, *J* = 6.8 Hz, 4H, C*H*(CH_3)$_2$), 1.50 (s, 6H, NCCH_3), 1.44-1.48 (m, 24H, CH(CH_3)$_2$), 1.19 (s, 27H, C(CH_3)$_3$), 1.16 (d, *J* = 7.2 Hz, 12H, CH(CH_3)$_2$), 1.09 (d, *J* = 6.8 Hz, 12H, CH(CH_3)$_2$).

^{13}C{^{1}H} NMR (100.6 MHz, C_6D_6): δ [ppm] 219.6 (Fe*C*O), 219.5 (Fe*C*O), 147.0 (N*C*N), 146.5 (Ar*C*), 145.3 (Ar*C*), 145.3 (Ar*C*), 144.2 (Ar*C*), 134.9 (Ar*C*), 132.9 (Ar*C*), 130.6 (Ar*C*), 129.7 (Ar*C*), 124.8 (ArC), 124.5 (ArC), 124.2 (ArC), 124.2 (ArC), 123.2 (N*C*H), 123.1 (N*C*H), 117.6 (N*C*-CH_3), 32.3 (C(*C*H_3)$_3$), 28.7 (*C*H(CH_3)$_2$), 28.6 (*C*H(CH_3)$_2$), 28.4 (*C*H(CH_3)$_2$), 24.8 (CH(*C*H_3)$_2$), 24.7 (CH(*C*H_3)$_2$), 24.6 (CH(*C*H_3)$_2$), 24.6 (CH(*C*H_3)$_2$), 24.0 (*C*(CH_3)$_3$), 24.0 (CH(*C*H_3)$_2$), 23.8 (CH(*C*H_3)$_2$), 23.7 (CH(*C*H_3)$_2$), 23.6 (CH(*C*H_3)$_2$), 10.0 (NC-*C*H_3).

^{29}Si{^{1}H} NMR (79.5 MHz, C_6D_6): δ [ppm] 4.1 (d, $J_{Si–P}$ = 9.0 Hz, *Si*^{t}Bu$_3$), –24.7 (d, $J_{Si–P}$ = 50.3 Hz, *Si*P).

^{31}P NMR (162.0 MHz, C_6D_6): δ [ppm] 168.4 (dd, ^{107}Ag: $J_{P–Ag}$ = 345.4 Hz and ^{109}Ag: $J_{P–Ag}$ = 398.6 Hz).

LIFDI-MS: Calcd (-(CO)$_4$): 1267.5833; Found: 1267.5821.

IR (Fe–CO, cm^{-1}): 2016, 1926, 1912, 1901.

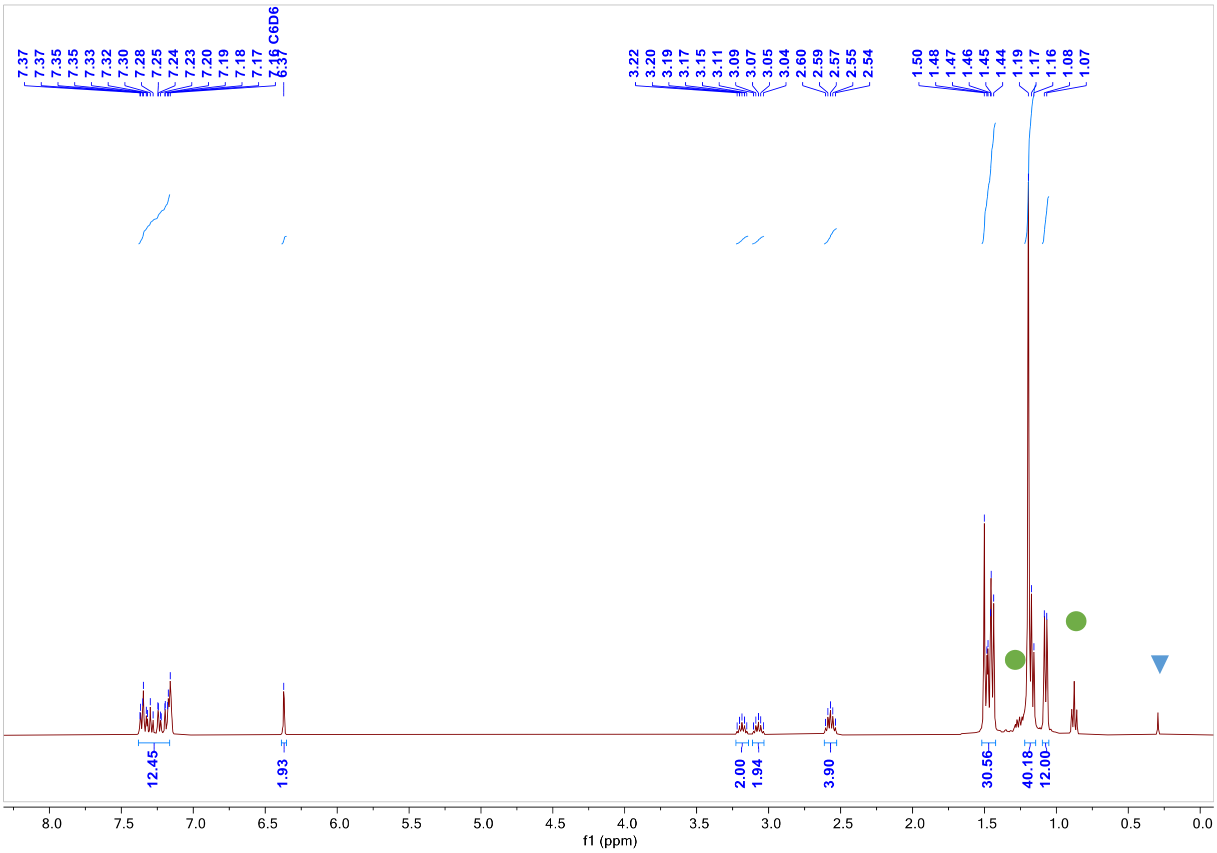

Figure S9.1 ^{1}H NMR spectrum of **10** in C_6D_6 at 300 K (●: pentane; ▼: silicon grease).

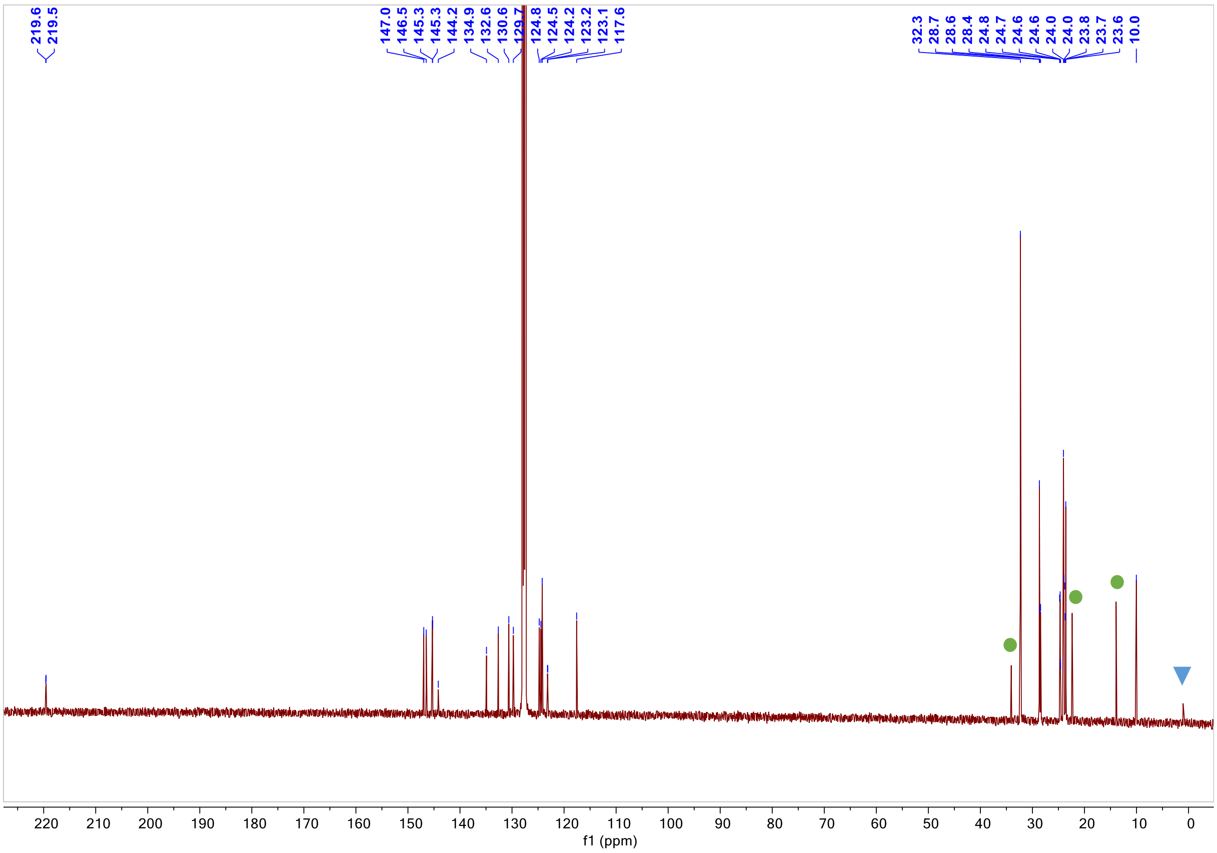

Figure S9.2 $^{13}C\{^{1}H\}$ NMR spectrum of **10** in C_6D_6 at 300 K (●: pentane; ▼: silicon grease).

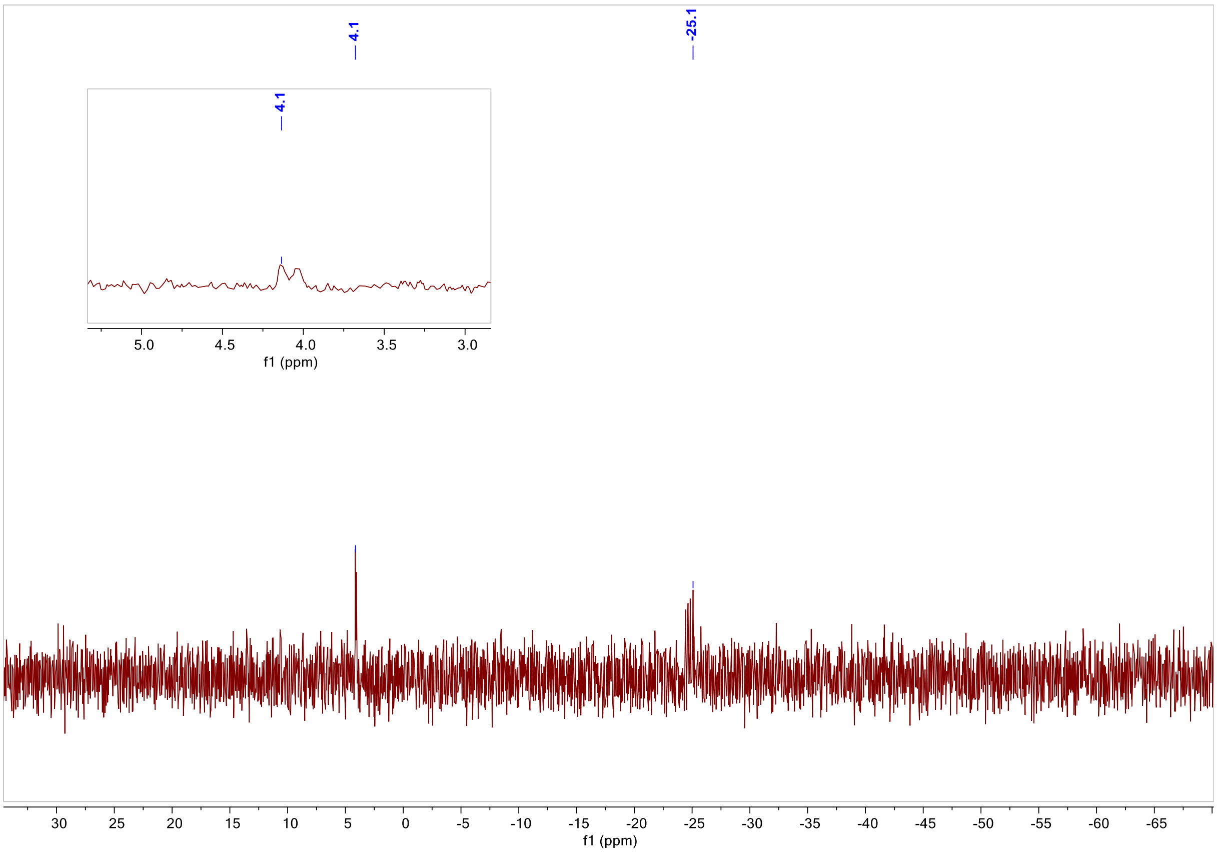

Figure S9.3 $^{29}Si\{^1H\}$ NMR spectrum of **10** in C_6D_6 at 300 K.

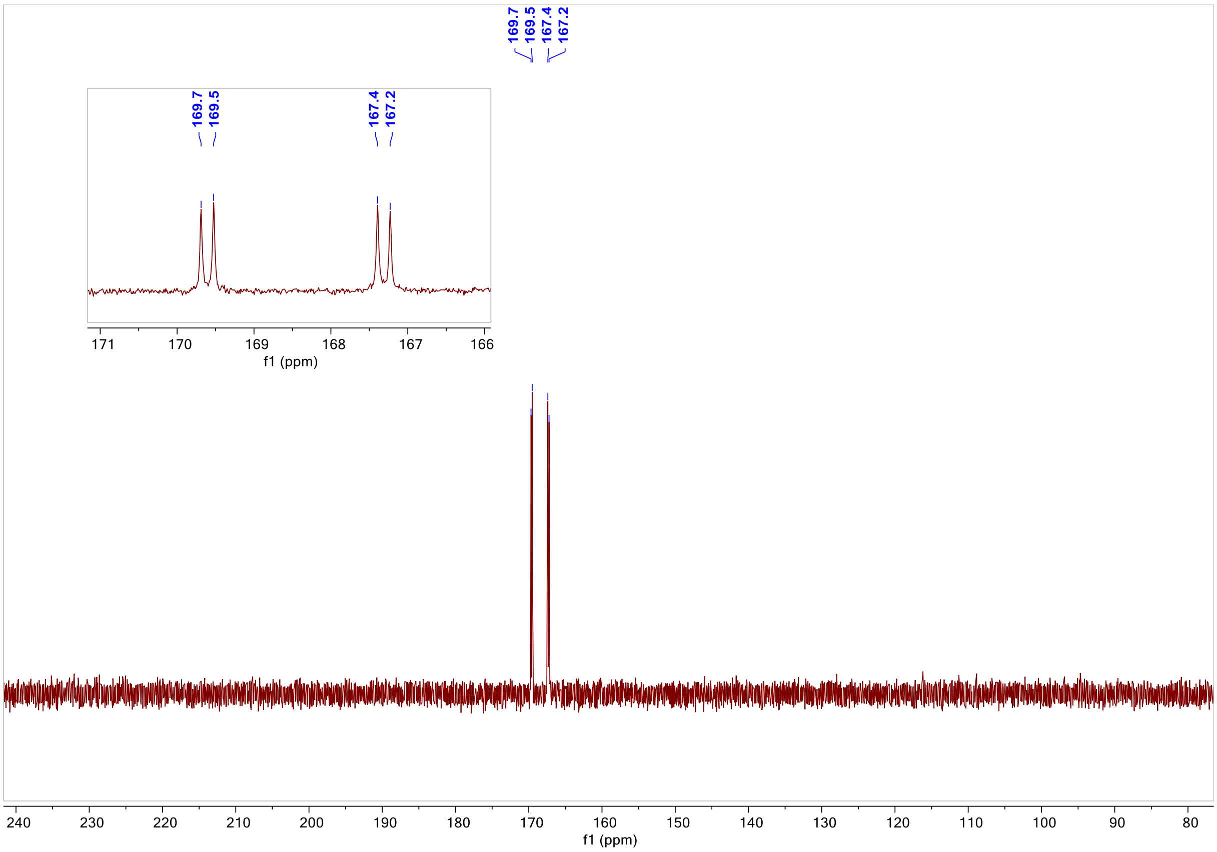

Figure S9.4 ^{31}P NMR spectrum of **10** in C_6D_6 at 300 K.

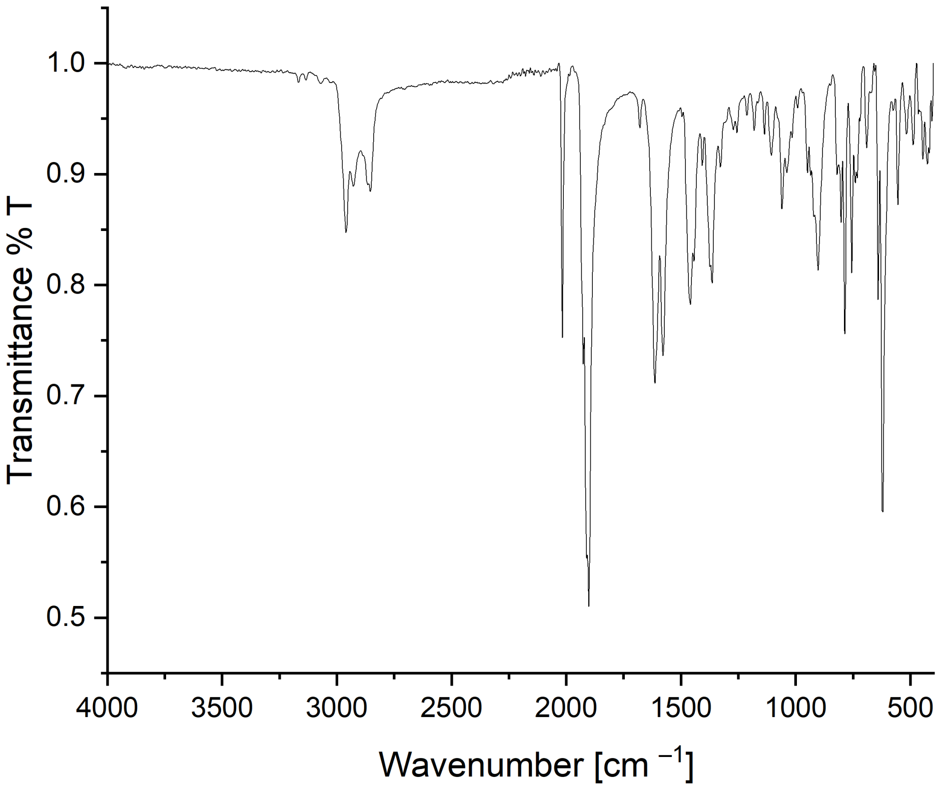

Figure S9.5 IR spectrum of **10**.

1.2.10 Synthesis of Silylene-iron complex (11)

Iron carbonyl (4.0 mg, 0.02 mmol) was added to a benzene solution of *Si*-silveriophosphasilene **4** (23.7 mg, 0.02 mmol) at room temperature. The color of solution changed to red from yellow rapidly. All volatiles were removed *in vacuum*, the red residue was extracted with pentane and recrystallized from pentane at –30 °C to yield silylene-iron complex **11** (24.6 mg, 89%) as orange crystals.

^{1}H NMR (400.1 MHz, C_6D_6): δ [ppm] 7.20-7.34 (m, 12H, Ar*H*), 6.42 (s, 2H, NC*H*), 3.36 (br, 4H, C*H*(CH_3)$_2$), 2.89 (sept, J = 6.8 Hz, 4H, C*H*(CH_3)$_2$), 1.62 (d, J = 6.6 Hz, 12H, CH(CH_3)$_2$), 1.53 (s, 6H, NCCH_3), 1.40 (d, J = 7.2 Hz, 12H, CH(CH_3)$_2$), 1.15 (s, 27H, C(CH_3)$_3$), 1.13 (d, J = 6.8 Hz, 12H, CH(CH_3)$_2$), 1.02 (d, J = 6.8 Hz, 12H, CH(CH_3)$_2$).

^{13}C{^{1}H} NMR (100.6 MHz, C_6D_6): δ [ppm] 216.9 (Fe*C*O), 216.9 (Fe*C*O), 190.5 (NHC, $J_{P–C}$ = 13.9 Hz), 147.9 (N*C*N), 145.9 (Ar*C*), 144.7 (Ar*C*), 135.8 (Ar*C*), 130.0 (Ar*C*), 129.5 (Ar*C*), 129.0 (Ar*C*), 124.6 (Ar*C*), 124.5 (Ar*C*), 123.2 (N*C*H), 123.2 (N*C*H), 118.4 (N*C*-CH_3), 32.3 (C(*C*H_3)$_3$), 32.1 (C(*C*H_3)$_3$), 28.5 (*C*H(CH_3)$_2$), 28.1 (*C*H(CH_3)$_2$), 25.1 (CH(*C*H_3)$_2$), 24.9 (CH(*C*H_3)$_2$), 24.7 (*C*(CH_3)$_3$), 24.2 (CH(*C*H_3)$_2$), 24.1 (CH(*C*H_3)$_2$), 23.3 (CH(*C*H_3)$_2$), 10.2 (NC-*C*H_3).

^{29}Si{^{1}H} NMR (79.5 MHz, C_6D_6): δ [ppm] 32.7 (d, $J_{Si–P}$ = 60.2 Hz, *Si*^{t}Bu$_3$), 210.4 (d, $J_{Si–P}$ = 132.0 Hz, *Si*P).

^{31}P NMR (162.0 MHz, C_6D_6): δ [ppm] –149.1 (dd, ^{107}Ag: $J_{P–Ag}$ = 292.5 Hz and ^{109}Ag: $J_{P–Ag}$ = 337.7 Hz).

LIFDI-MS: Calcd (-(CO)$_3$): 1267.5833; Found: 1267.5909.

IR (Fe–CO, cm^{-1}): 2010, 1924, 1901, 1870.

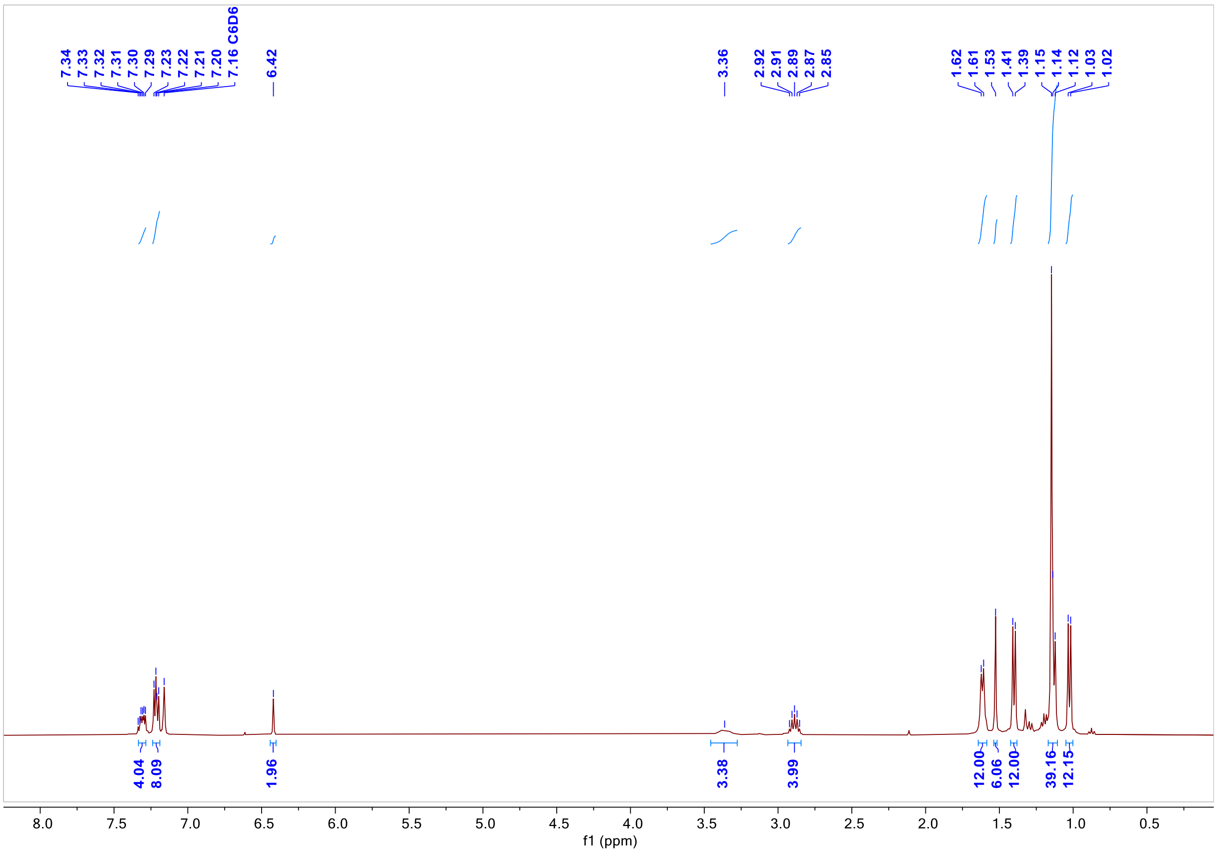

Figure S10.1 ^{1}H NMR spectrum of **11** in C_6D_6 at 300 K.

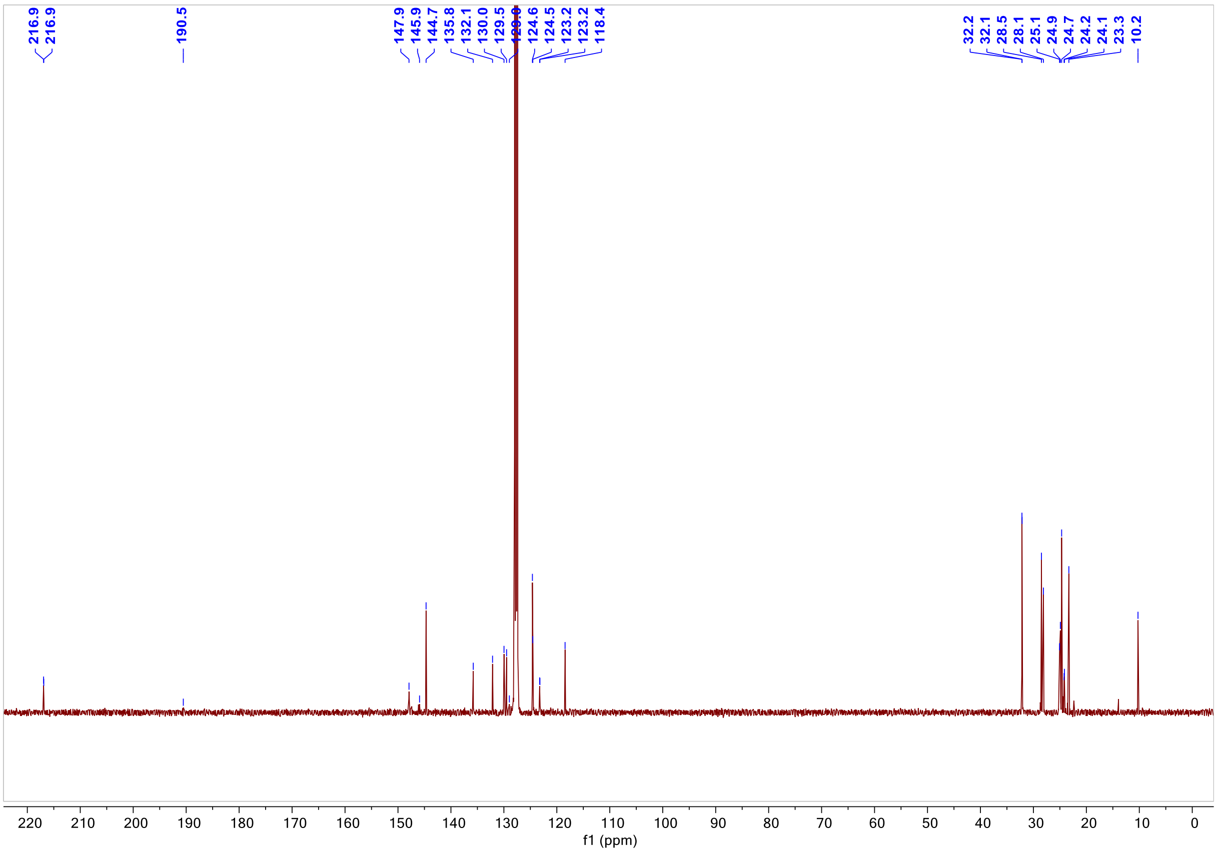

Figure S10.2 $^{13}C\{^{1}H\}$ NMR spectrum of **11** in C_6D_6 at 300 K.

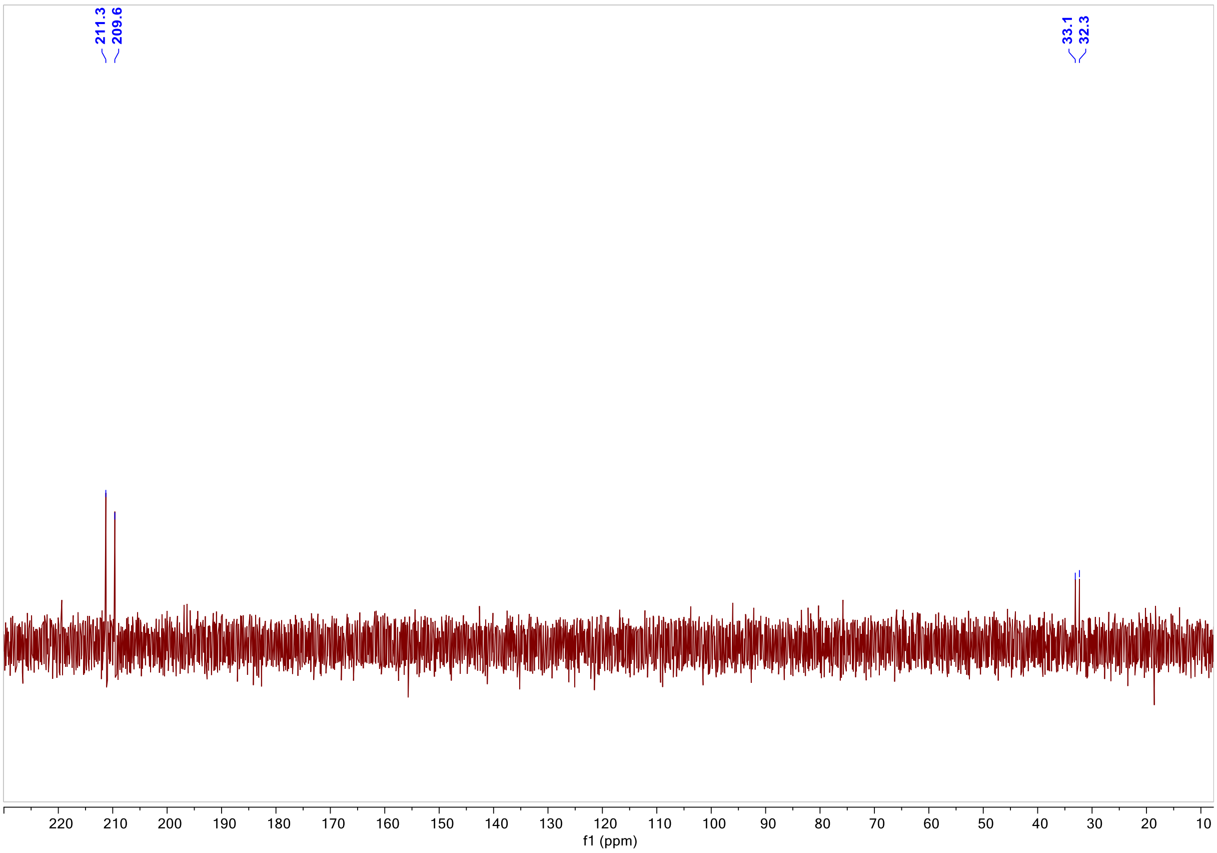

Figure S10.3 $^{29}Si\{^1H\}$ NMR spectrum of **11** in C_6D_6 at 300 K.

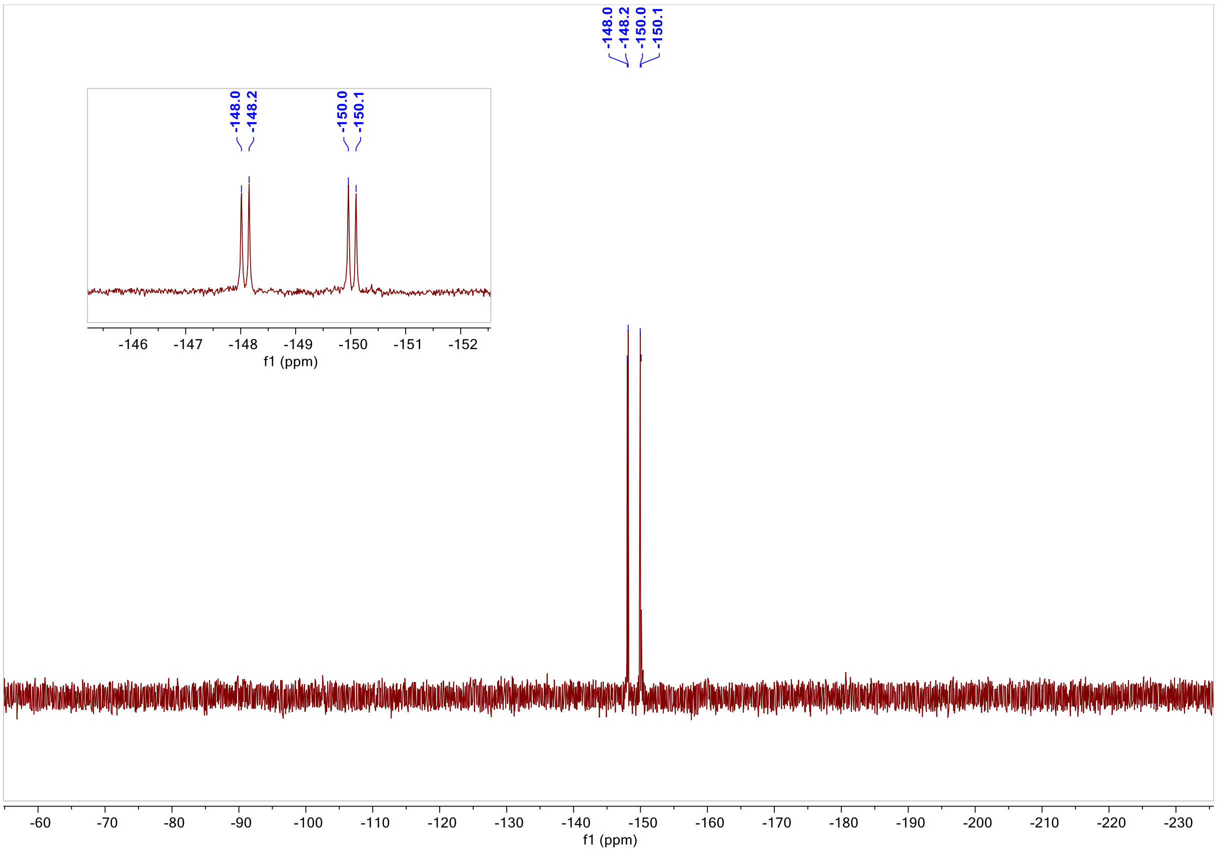

Figure S10.4 ^{31}P NMR spectrum of **11** in C_6D_6 at 300 K.

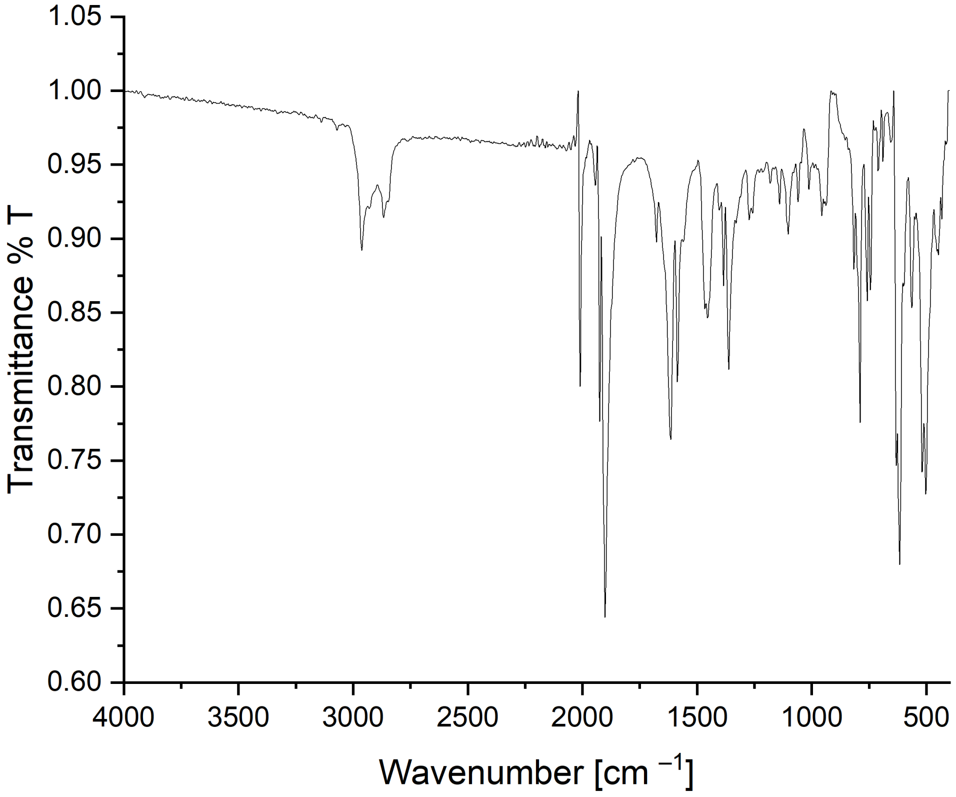

Figure S10.5 IR spectrum of **11**.

1.2.11 Synthesis of Aminosilane (12)

A toluene solution of *P*-silveriophosphasilene **4** (29.6 mg, 0.025 mmol) was exposed to ammonia (1 bar) at –30 °C. The mixture was allowed to warm to room temperature slowly and the color of mixture vanished from bright yellow. The pure aminosilane **12** (28.6 mg, 95%) was obtained by removing all volatiles *in vacuum* as a colorless powder. Colorless crystals suitable for single X-ray diffraction analysis was obtained by storing saturated pentane solution at –30 °C for 2 days.

^{1}H NMR (400.1 MHz, C_6D_6): δ [ppm] 7.08-7.38 (m, 12H, Ar*H*, overlapping with C_6D_6), 6.30 (s, 2H, NC*H*), 3.78 (sept, *J* = 6.8 Hz, 2H, C*H*(CH_3)$_2$), 3.45 (sept, *J* = 6.8 Hz, 2H, C*H*(CH_3)$_2$), 2.56-2.62 (m, 4H, C*H*(CH_3)$_2$), 1.54 (d, *J* = 5.6 Hz, 6H, CH(C*H*$_3$)$_2$), 1.55 (s, 6H, NCC*H*$_3$), 1.49 (d, *J* = 5.6 Hz, 6H, CH(C*H*$_3$)$_2$), 1.36 (d, *J* = 6.8 Hz, 6H, CH(C*H*$_3$)$_2$), 1.26-1.31 (m, 45H, CH(C*H*$_3$)$_2$ and C(C*H*$_3$)$_3$), 1.14 (d, *J* = 6.8 Hz, 6H, CH(C*H*$_3$)$_2$), 1.07 (d, *J* = 6.8 Hz, 6H, CH(C*H*$_3$)$_2$), 1.04 (d, *J* = 6.8 Hz, 6H, CH(C*H*$_3$)$_2$), 0.50 (dd, ^{107}Ag: $J_{H–Ag}$ = 163.9 Hz and ^{109}Ag: $J_{H–Ag}$ = 184.9 Hz, P*H*), –0.28 (s, 2H, N*H*$_2$).

^{13}C{^{1}H} NMR (100.6 MHz, C_6D_6): δ [ppm] 148.5 (Ar*C*), 148.3 (Ar*C*), 145.4 (N*C*N), 135.4 (Ar*C*), 135.3 (Ar*C*), 130.2 (Ar*C*), 128.3 (Ar*C*), 124.2 (Ar*C*), 124.1 (Ar*C*), 123.9 (ArC), 123.8 (ArC), 122.6 (N*C*H), 122.5 (N*C*H), 116.3 (N*C*-CH_3), 32.6 (C(*C*H$_3$)$_3$), 28.6 (*C*H(CH_3)$_2$), 28.3 (*C*H(CH_3)$_2$), 26.3 (*C*H(CH_3)$_2$), 26.3 (*C*H(CH_3)$_2$), 23.8 (*C*H(CH_3)$_2$), 23.8 (*C*H(CH_3)$_2$), 23.7 (*C*H(CH_3)$_2$), 23.7 (CH(*C*H$_3$)$_2$), 23.6 (CH(*C*H$_3$)$_2$), 23.4 (*C*(CH_3)$_3$), 10.7 (NC-*C*H$_3$).

^{29}Si{^{1}H} NMR (79.5 MHz, C_6D_6): δ [ppm] –2.9 (d, $J_{Si–P}$ = 16.8 Hz, *Si*^{t}Bu$_3$), –51.7 (d, $J_{Si–P}$ = 29.9 Hz, H_2N*Si*P).

^{31}P NMR (162.0 MHz, C_6D_6): δ [ppm] –202.6 (m).

LIFDI-MS: Calcd (-Si^{t}Bu$_3$): 1001.4924; Found: 1001.4984.

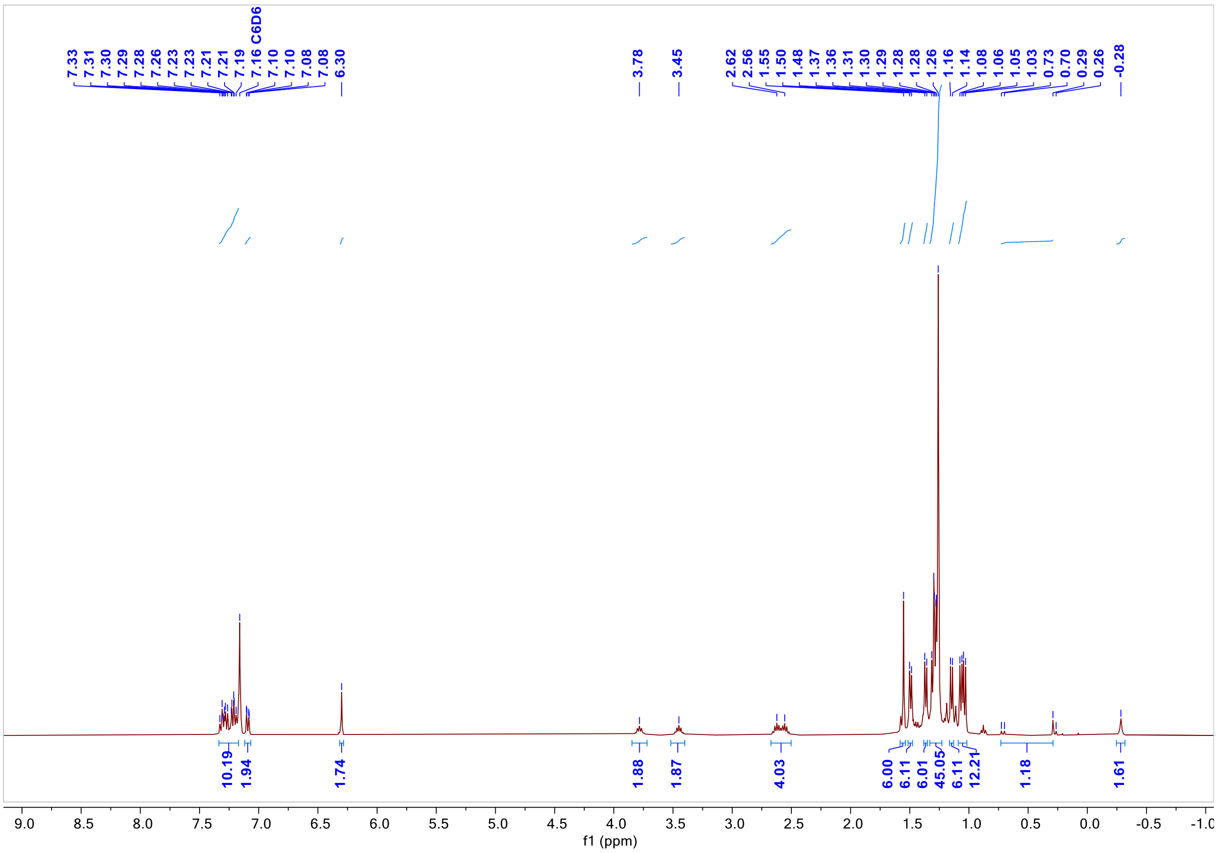

Figure S11.1 ^{1}H NMR spectrum of **12** in C_6D_6 at 300 K.

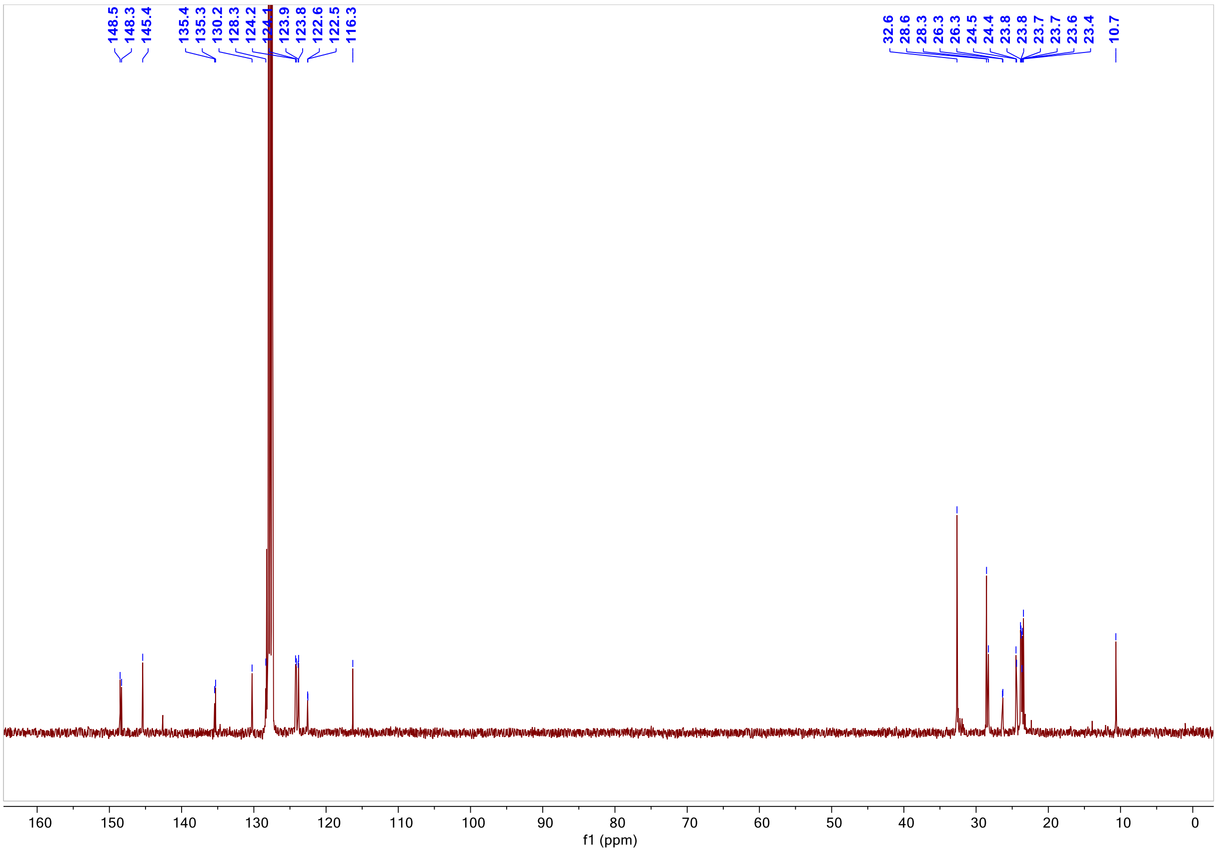

Figure S11.2 $^{13}C\{^{1}H\}$ NMR spectrum of **12** in C_6D_6 at 300 K.

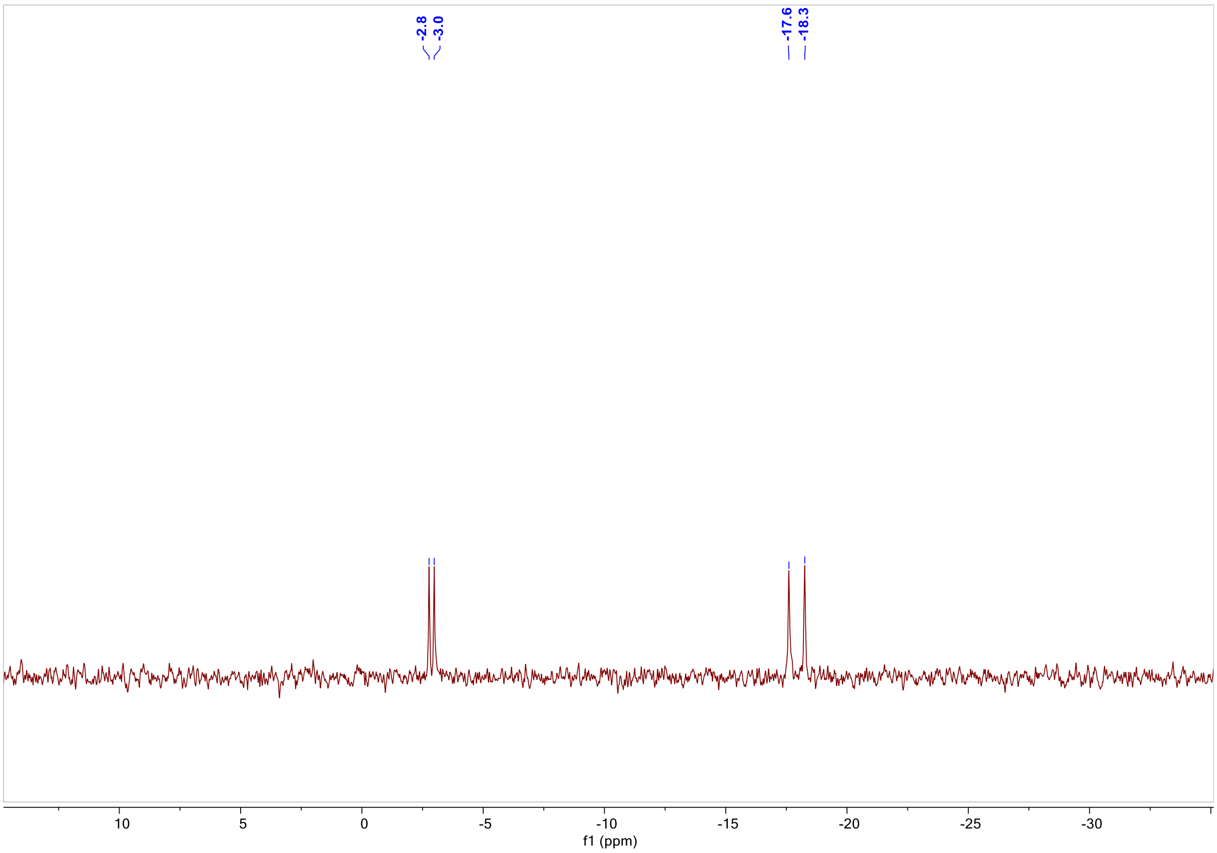

Figure S11.3 $^{29}Si\{^1H\}$ NMR spectrum of **12** in C_6D_6 at 300 K.

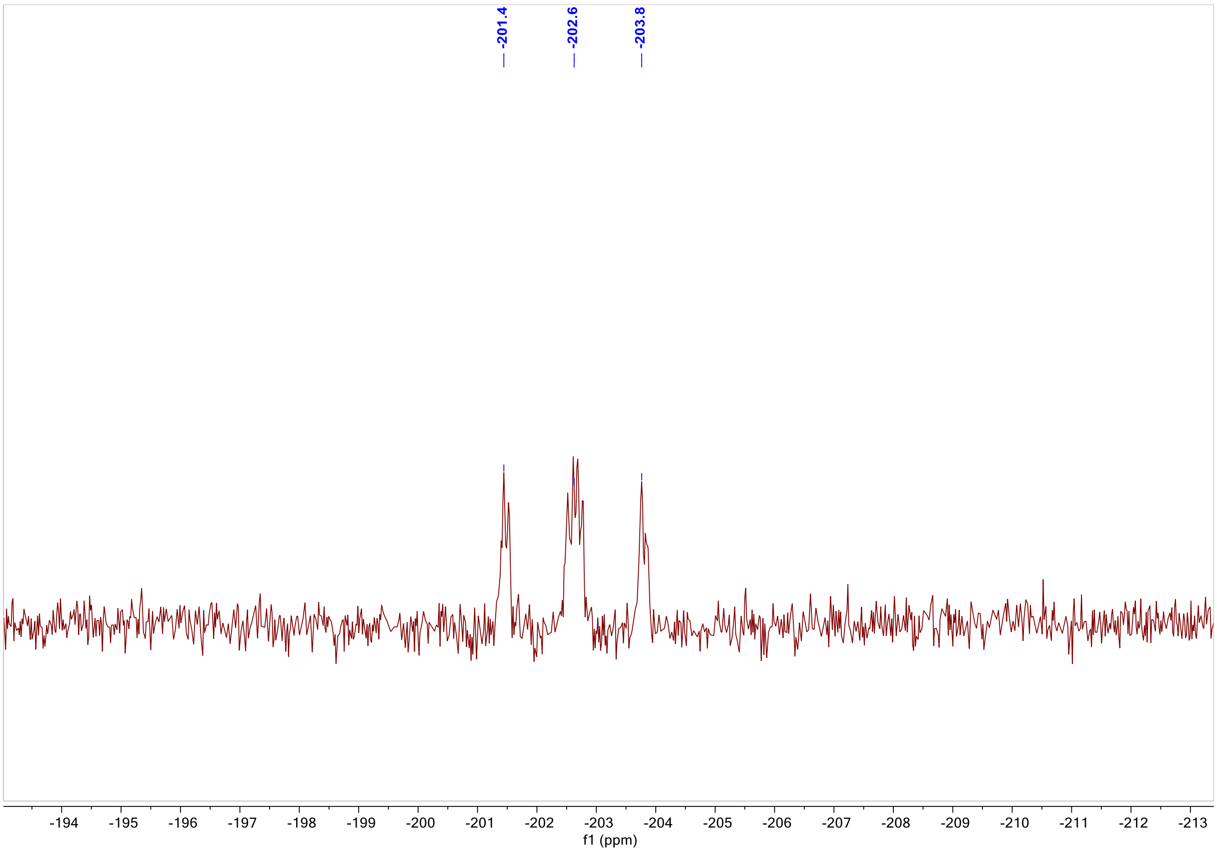

Figure S11.4 ^{31}P NMR spectrum of **12** in C_6D_6 at 300 K.

1.2.12 Synthesis of Aminosilane (13)

$BnNH_2$ (2.2 mg, 0.02 mmol) was added to a benzene solution of *P*-silveriophosphasilene **4** (23.7 mg, 0.02 mmol) at –30 °C. The mixture was allowed to warm to room temperature slowly and the color of mixture vanished from bright yellow. The pure aminosilane **13** (25.2 mg, 97%) was obtained by removing all volatiles *in vacuum* as a colorless powder. Colorless crystals suitable for single X-ray diffraction analysis was obtained by storing saturated pentane solution at –30 °C for 24 h.

^{1}H NMR (400.1 MHz, C_6D_6): δ [ppm] 6.96-7.32 (m, 17H, Ar*H*, overlapping with C_6D_6), 6.31 (s, 2H, NC*H*), 3.95-4.01 (m, 1H, PhC*H*$_2$N), 3.81 (br, 2H, C*H*(CH_3)$_2$), 3.38-3.47 (m, 3H, PhC*H*$_2$N and C*H*(CH_3)$_2$), 3.15 (sept, *J* = 6.8 Hz, 2H, C*H*(CH_3)$_2$), 2.55-2.68 (m, 4H, C*H*(CH_3)$_2$), 1.54 (d, *J* = 5.6 Hz, 6H, CH(C*H*$_3$)$_2$), 1.47 (s, 6H, NCC*H*$_3$), 1.41 (d, *J* = 5.6 Hz, 6H, CH(C*H*$_3$)$_2$), 1.36 (d, *J* = 6.8 Hz, 6H, CH(C*H*$_3$)$_2$), 1.23-1.28 (m, 39H, CH(C*H*$_3$)$_2$ and C(C*H*$_3$)$_3$), 1.14 (d, *J* = 6.8 Hz, 6H, CH(C*H*$_3$)$_2$), 1.08 (d, *J* = 6.8 Hz, 6H, CH(C*H*$_3$)$_2$), 1.03 (d, *J* = 6.8 Hz, 6H, CH(C*H*$_3$)$_2$), –0.05 (dd, ^{107}Ag: $J_{H–Ag}$ = 164.0 Hz and ^{109}Ag: $J_{H–Ag}$ = 186.3 Hz, P*H*), –0.62 (m, 1H, N*H*).

^{13}C{^{1}H} NMR (100.6 MHz, C_6D_6): δ [ppm] 147.9 (Ar*C*), 147.9 (Ar*C*), 145.6 (Ar*C*), 145.4 (Ar*C*), 145.4 (Ar*C*), 143.3 (N*C*N), 135.4 (Ar*C*), 135.0 (Ar*C*), 130.2 (Ar*C*), 128.7 (Ar*C*), 128.0 (ArC), 127.1 (ArC), 125.2 (ArC), 124.2 (ArC), 124.1 (ArC), 123.8 (ArC), 122.6 (N*C*H), 122.5 (N*C*H), 115.2 (N*C*-CH_3), 48.4 (Ph*C*H$_2$N), 32.8 (C(*C*H$_3$)$_3$), 28.6 (*C*H(CH_3)$_2$), 28.6 (*C*H(CH_3)$_2$), 28.3 (*C*H(CH_3)$_2$), 28.3 (*C*H(CH_3)$_2$), 25.8 (*C*H(CH_3)$_2$), 25.7 (*C*H(CH_3)$_2$), 24.7 (*C*(CH_3)$_3$), 24.2 (CH(*C*H$_3$)$_2$), 24.2 (CH(*C*H$_3$)$_2$), 23.9 (CH(*C*H$_3$)$_2$), 23.9 (CH(*C*H$_3$)$_2$), 23.7 (CH(*C*H$_3$)$_2$), 23.6 (CH(*C*H$_3$)$_2$), 22.3 (CH(*C*H$_3$)$_2$), 10.6 (NC-*C*H$_3$).

^{29}Si{^{1}H} NMR (79.5 MHz, C_6D_6): δ [ppm] –1.4 (d, $J_{Si–P}$ = 19.6 Hz, *Si*^{t}Bu$_3$), –14.6 (d, $J_{Si–P}$ = 61.5 Hz, Bn(H)N*Si*P).

^{31}P NMR (162.0 MHz, C_6D_6): δ [ppm] –233.9 (dddd, $J_{P–H}$ = 175.3 Hz, ^{107}Ag: $J_{P–Ag}$ = 188.2 Hz and ^{109}Ag: $J_{P–Ag}$ = 217.0 Hz).

LIFDI-MS: Calcd (-Si^{t}Bu$_3$): 1091.5394; Found: 1091.5462.

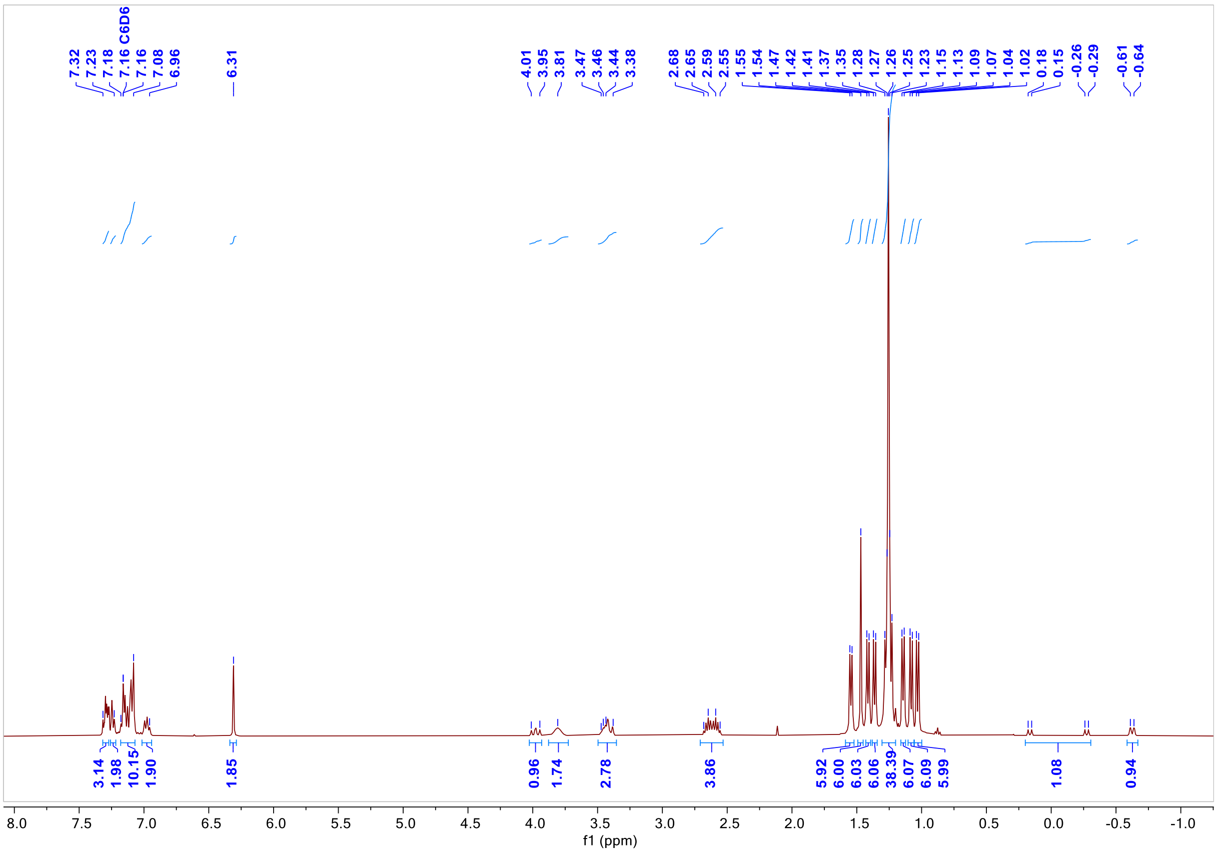

Figure S12.1 ^{1}H NMR spectrum of **13** in C_6D_6 at 300 K.

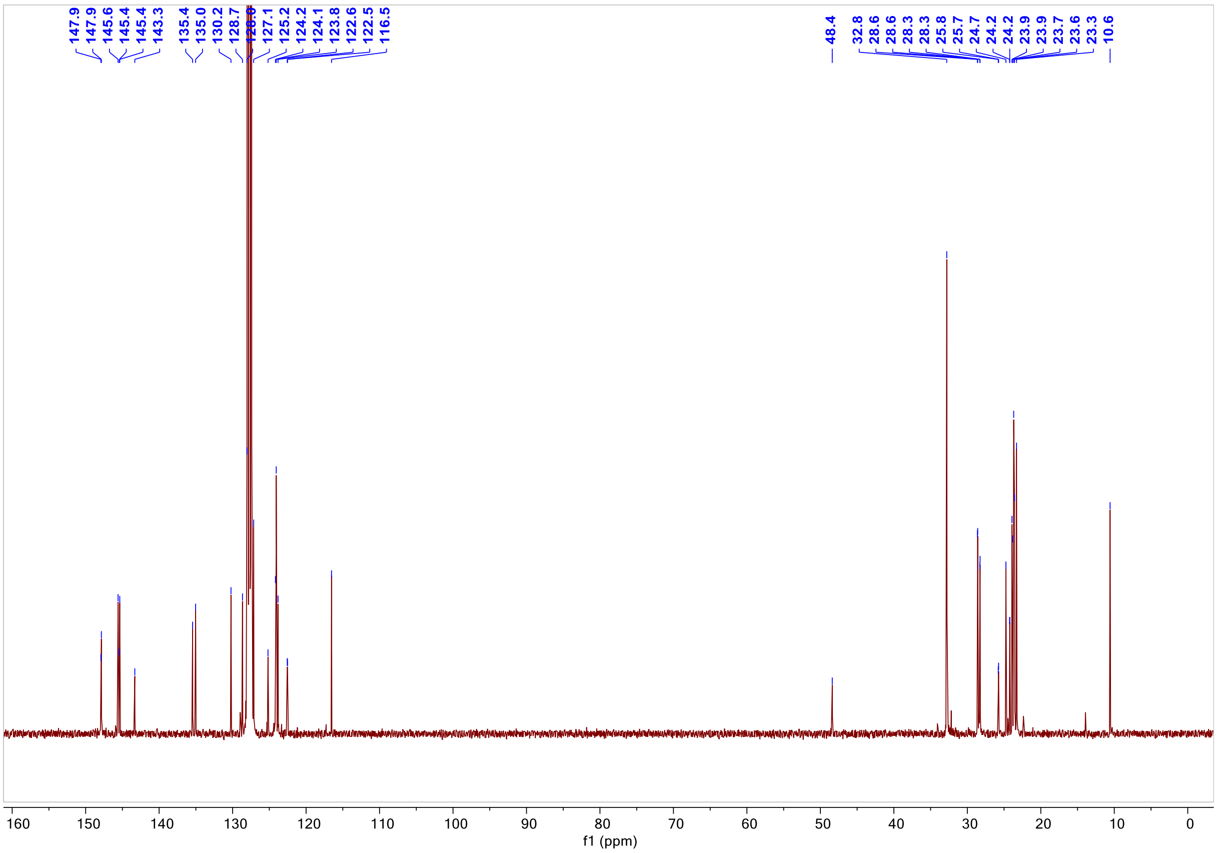

Figure S12.2 $^{13}C\{^{1}H\}$ NMR spectrum of **13** in C_6D_6 at 300 K.

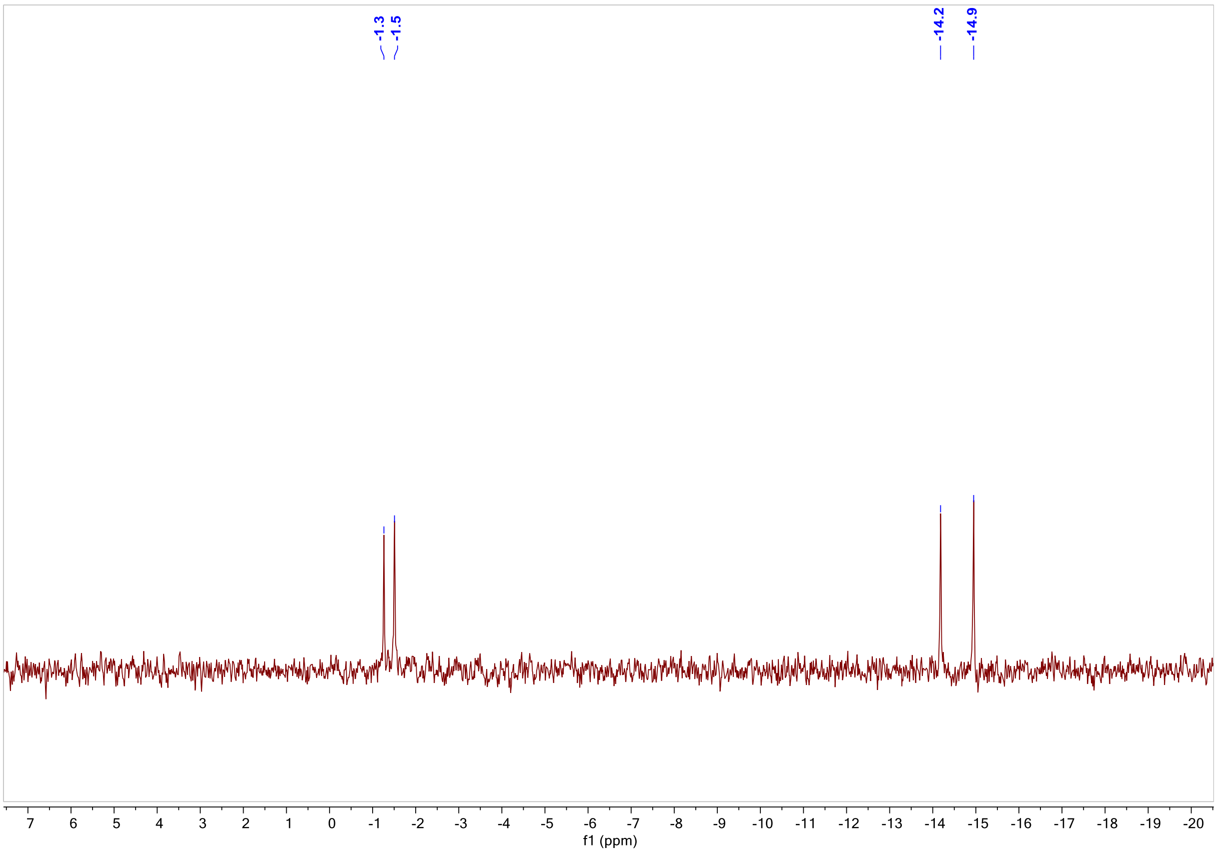

Figure S12.3 $^{29}Si\{^{1}H\}$ NMR spectrum of **13** in C_6D_6 at 300 K.

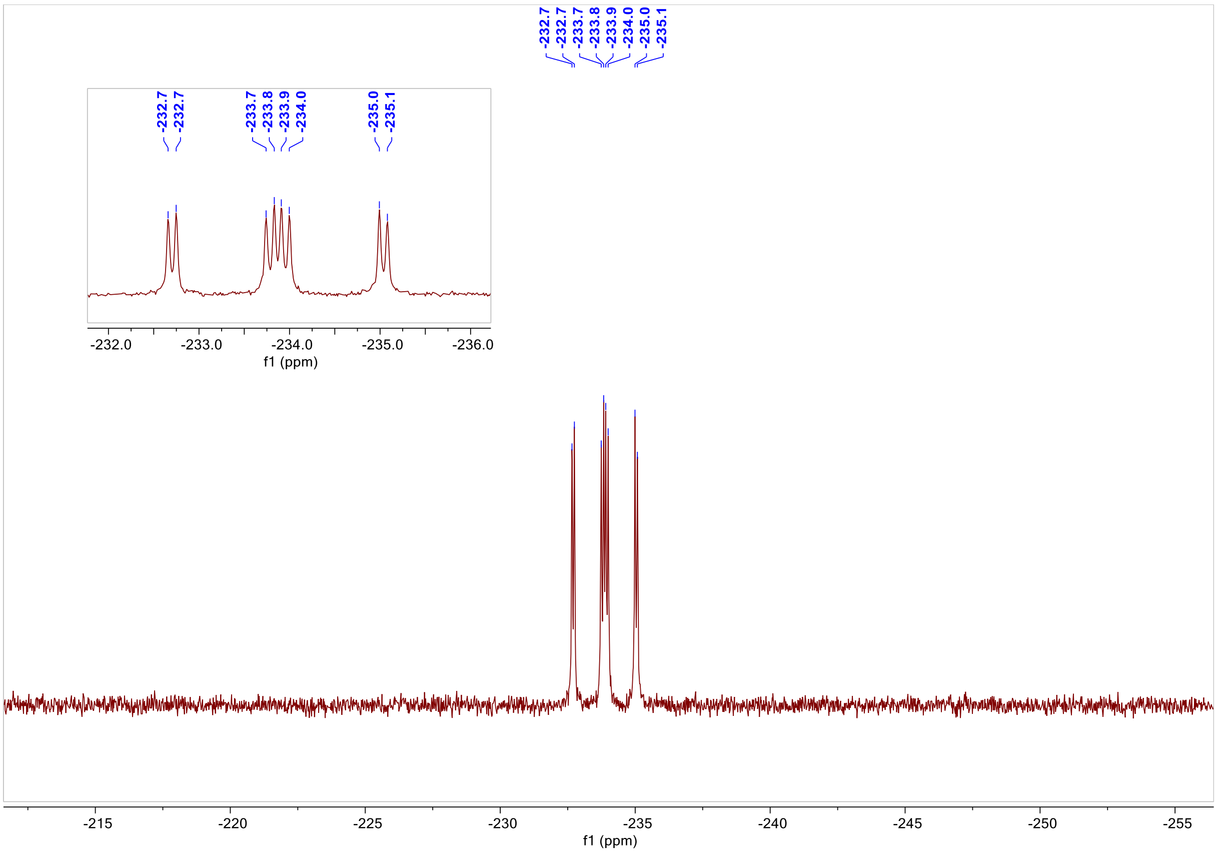

Figure S12.4 ^{31}P NMR spectrum of **13** in C_6D_6 at 300 K.

1.2.13 Synthesis of Silver-substituted Hydrophosphine (14)

$BnNH_2$ (2.2 mg, 0.02 mmol) was added to a benzene solution of *Si*-silveriophosphasilene **7** (23.7 mg, 0.02 mmol) at room temperature. The color of mixture vanished from bright yellow after stirring for 1 h. All volatiles were removed *in vacuum*, the pale yellow residue was extracted with pentane and recrystallized from pentane at –30 °C to yield hydrophosphine **14** (8.6 mg, 59%) as colorless crystals.

Note: Changing $BnNH_2$ to ammonia also led to the formation of **14** as detected by ^{1}H and ^{31}P NMR.

^{1}H NMR (400.1 MHz, C_6D_6): δ [ppm] 7.18-7.20 (m, 2H, Ar*H*), 7.05-7.07 (d, *J* = 7.6 Hz, 4H, Ar*H*), 6.34 (s, 2H, NC*H*), 3.19 (br, 1H, P*H*), 3.65 (sept, *J* = 6.8 Hz, 2H, C*H*(CH_3)$_2$), 2.58 (sept, *J* = 6.8 Hz, 4H, C*H*(CH_3)$_2$), 1.38 (d, *J* = 6.8 Hz, 12H, CH(C*H*$_3$)$_2$), 1.31 (s, 27H, C(C*H*$_3$)$_3$), 1.05 (d, *J* = 6.8 Hz, 12H, CH(C*H*$_3$)$_2$).

$^{13}C\{^{1}H\}$ NMR (100.6 MHz, C_6D_6): δ [ppm] 145.3 (Ar*C*), 135.0 (Ar*C*), 130.3 (Ar*C*), 123.9 (Ar*C*), 122.4 (N*C*H), 122.4 (N*C*H), 31.8 (C(*C*H$_3$)$_3$), 31.7 (C(*C*H$_3$)$_3$), 28.6 (*C*H(CH_3)$_2$), 24.2 (*C*(CH_3)$_3$), 23.8 (CH(*C*H$_3$)$_2$), 23.2 (CH(*C*H$_3$)$_2$), 23.1 (CH(*C*H$_3$)$_2$).

^{31}P NMR (162.0 MHz, C_6D_6): δ [ppm] –313.0 (dddd, $J_{P\text{–}H}$ = 170.9 Hz, ^{107}Ag: $J_{P\text{–}Ag}$ = 183.7 Hz and ^{109}Ag: $J_{P\text{–}Ag}$ = 212.0 Hz).

LIFDI-MS: Calcd: 726.3627; Found: 726.3753.

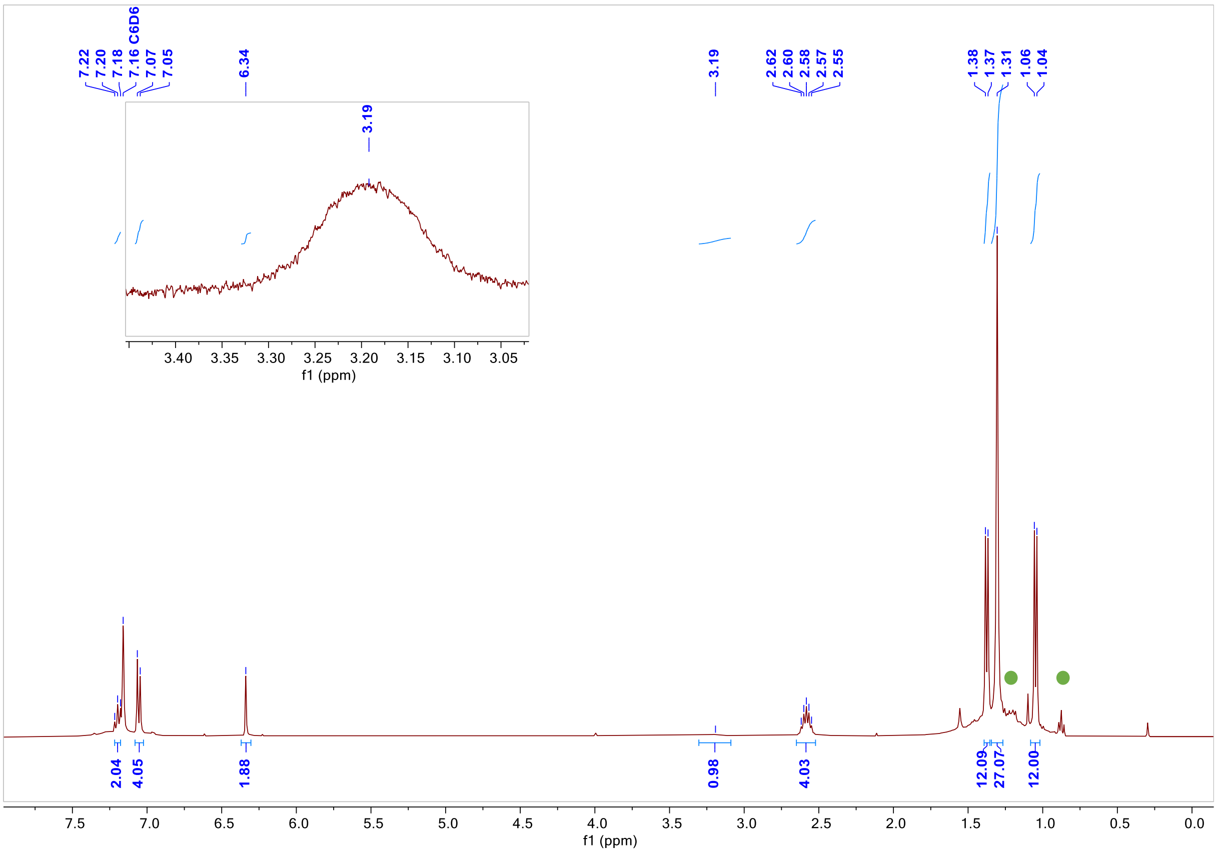

Figure S13.1 ^{1}H NMR spectrum of **14** in C_6D_6 at 300 K (●: pentane).

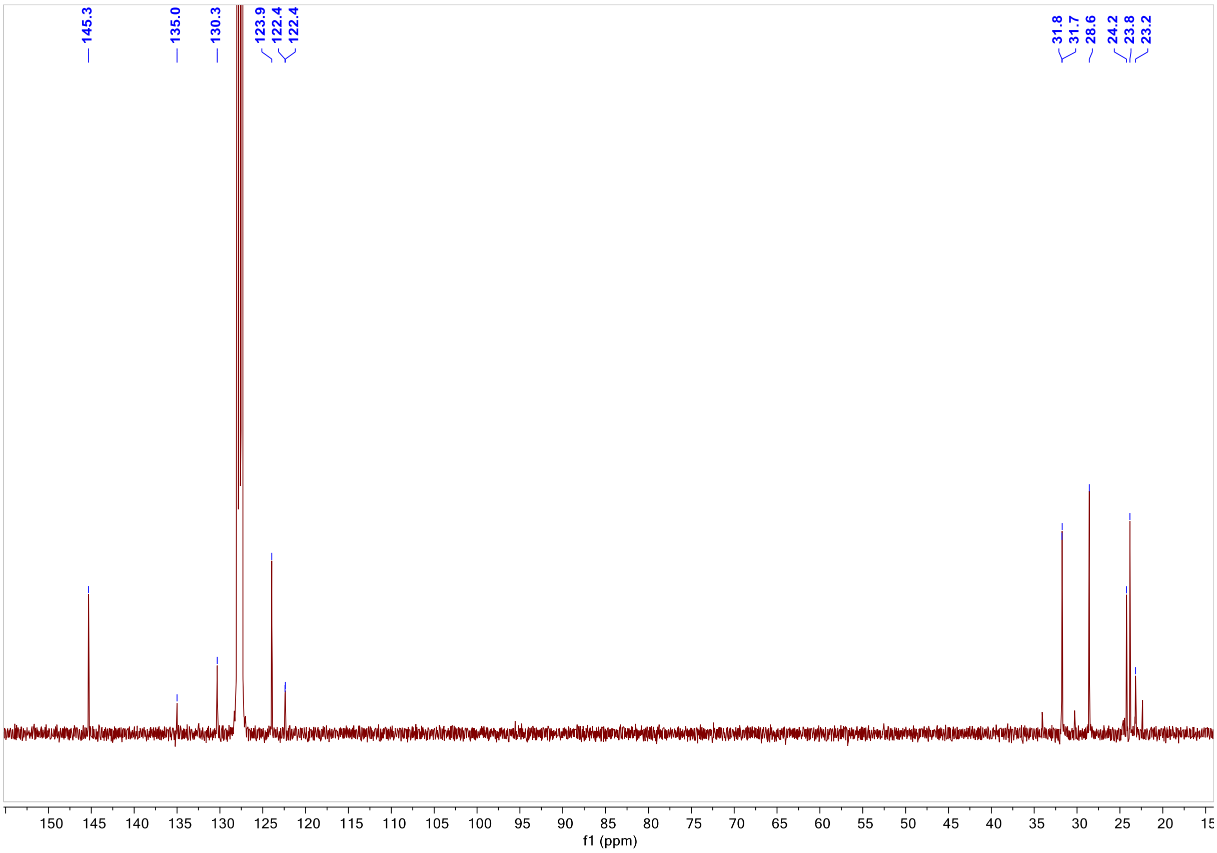

Figure S13.2 ^{13}C{^{1}H} NMR spectrum of **14** in C_6D_6 at 300 K.

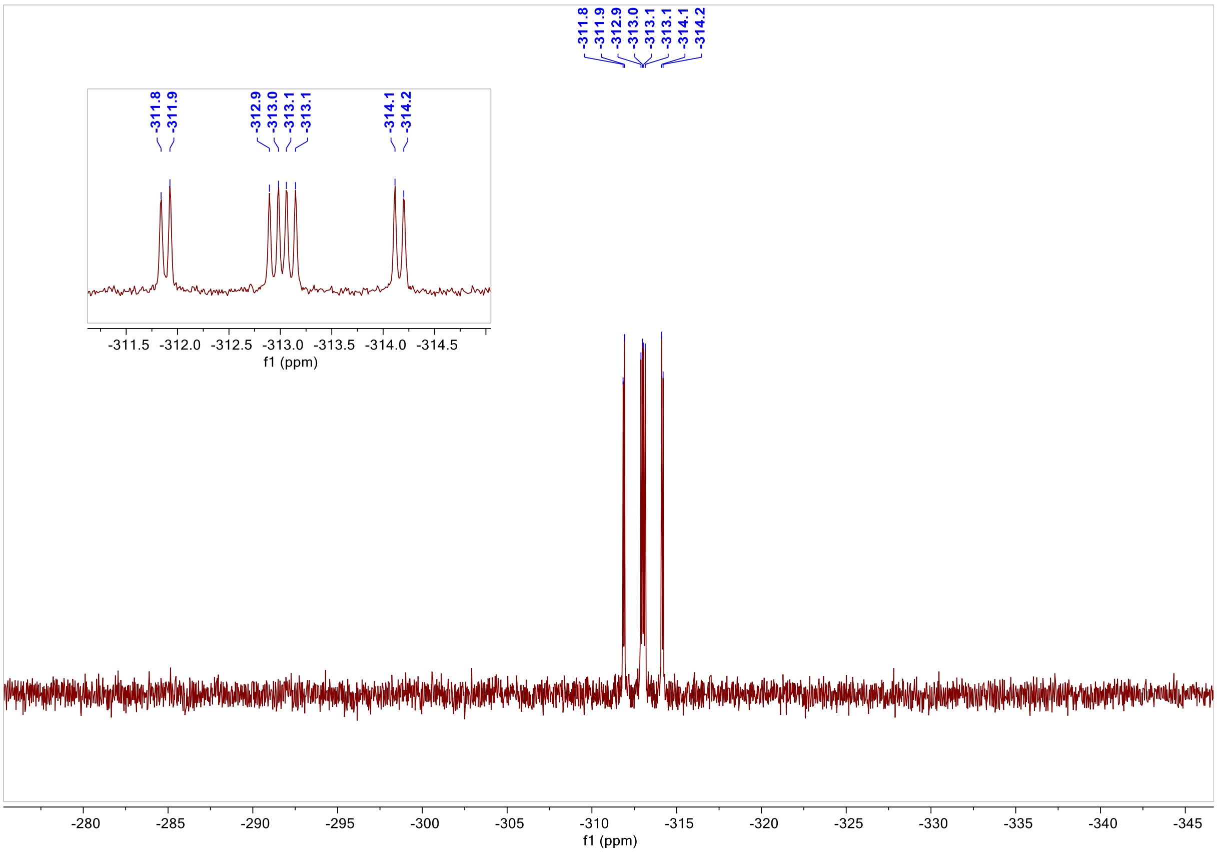

Figure S13.3 ^{31}P NMR spectrum of **14** in C_6D_6 at 300 K.

1.2.14 Synthesis of Hydrosilane (15)

Excess HBpin was added to a benzene solution of *Si*-silveriophosphasilene **7** (23.7 mg, 0.02 mmol) at room temperature. The color of mixture vanished from bright yellow after stirring for 1 h. All volatiles were removed *in vacuum*, the pale yellow residue was extracted with pentane and recrystallized from pentane at –30 °C to yield hydrosilane **15** (24.6 mg, 94%) as colorless crystals.

^{1}H NMR (400.1 MHz, C_6D_6): δ [ppm] 7.16-7.38 (m, 12H, Ar*H*, overlapping with C_6D_6), 6.29 (s, 2H, NC*H*), 4.37 (d, $J_{Si–H}$ = 183.8 Hz, $J_{P–H}$ = 58.6 Hz, 1H, Si*H*), 3.65 (sept, *J* = 6.8 Hz, 2H, C*H*(CH_3)$_2$), 3.15 (sept, *J* = 6.8 Hz, 2H, C*H*(CH_3)$_2$), 2.69 (sept, *J* = 6.8 Hz, 2H, C*H*(CH_3)$_2$), 2.64 (sept, *J* = 6.8 Hz, 2H, C*H*(CH_3)$_2$), 1.53 (d, *J* = 5.6 Hz, 6H, CH(CH_3)$_2$), 1.52 (d, *J* = 5.6 Hz, 6H, CH(CH_3)$_2$), 1.50 (s, 6H, NCCH_3), 1.40 (d, *J* = 6.8 Hz, 6H, CH(CH_3)$_2$), 1.32 (d, *J* = 6.8 Hz, 6H, CH(CH_3)$_2$), 1.29 (d, *J* = 6.8 Hz, 6H, CH(CH_3)$_2$), 1.28 (d, *J* = 6.8 Hz, 6H, CH(CH_3)$_2$), 1.17 (s, 12H, OC(CH_3)$_2$), 1.15 (s, 27H, C(CH_3)$_3$), 1.12 (d, *J* = 6.8 Hz, 6H, CH(CH_3)$_2$), 1.09 (d, *J* = 6.8 Hz, 6H, CH(CH_3)$_2$).

^{13}C{^{1}H} NMR (100.6 MHz, C_6D_6): δ [ppm] 149.1 (Ar*C*), 147.4 (Ar*C*), 145.4 (Ar*C*), 145.0 (Ar*C*), 140.5 (N*C*N), 130.2 (Ar*C*), 128.4 (Ar*C*), 124.1 (N*C*H), 123.8 (Ar*C*), 123.2 (Ar*C*), 122.9 (ArC), 122.9 (ArC), 115.2 (N*C*-CH_3), 81.5 (BO*C*), 32.6 (C(*C*H_3)$_3$), 32.5 (C(*C*H_3)$_3$), 28.6 (*C*H(CH_3)$_2$), 28.6 (*C*H(CH_3)$_2$), 28.5 (*C*H(CH_3)$_2$), 28.3 (*C*H(CH_3)$_2$), 26.2 (*C*H(CH_3)$_2$), 26.2 (*C*H(CH_3)$_2$), 25.0 (*C*(CH_3)$_3$), 24.7 (CH(*C*H_3)$_2$), 24.5 (CH(*C*H_3)$_2$), 24.4 (CH(*C*H_3)$_2$), 24.3 (CH(*C*H_3)$_2$), 24.2 (CH(*C*H_3)$_2$), 24.0 (CH(*C*H_3)$_2$), 23.8 (CH(*C*H_3)$_2$), 23.7 (OC*C*H_3), 23.6 (OC*C*H_3), 23.6 (OC*C*H_3), 23.6 (OC*C*H_3), 22.9 (CH(*C*H_3)$_2$), 9.8 (NC-*C*H_3).

^{29}Si{^{1}H} NMR (79.5 MHz, C_6D_6): δ [ppm] 29.5 (d, $J_{Si–P}$ = 68.7 Hz, *Si*^{t}Bu$_3$), –55.4 (d, $J_{Si–P}$ = 29.9 Hz, H*Si*P).

^{31}P NMR (162.0 MHz, C_6D_6): δ [ppm] –279.4 (dddd, $J_{P–H}$ = 58.6 Hz, ^{107}Ag: $J_{P–Ag}$ = 209.0 Hz and ^{109}Ag: $J_{P–Ag}$ = 325.9 Hz).

^{11}B NMR (128.4 MHz, C_6D_6): δ [ppm] 22.2 (*Bpin*).

LIFDI-MS: Calcd: 1311.7549; Found: 1311.7562.

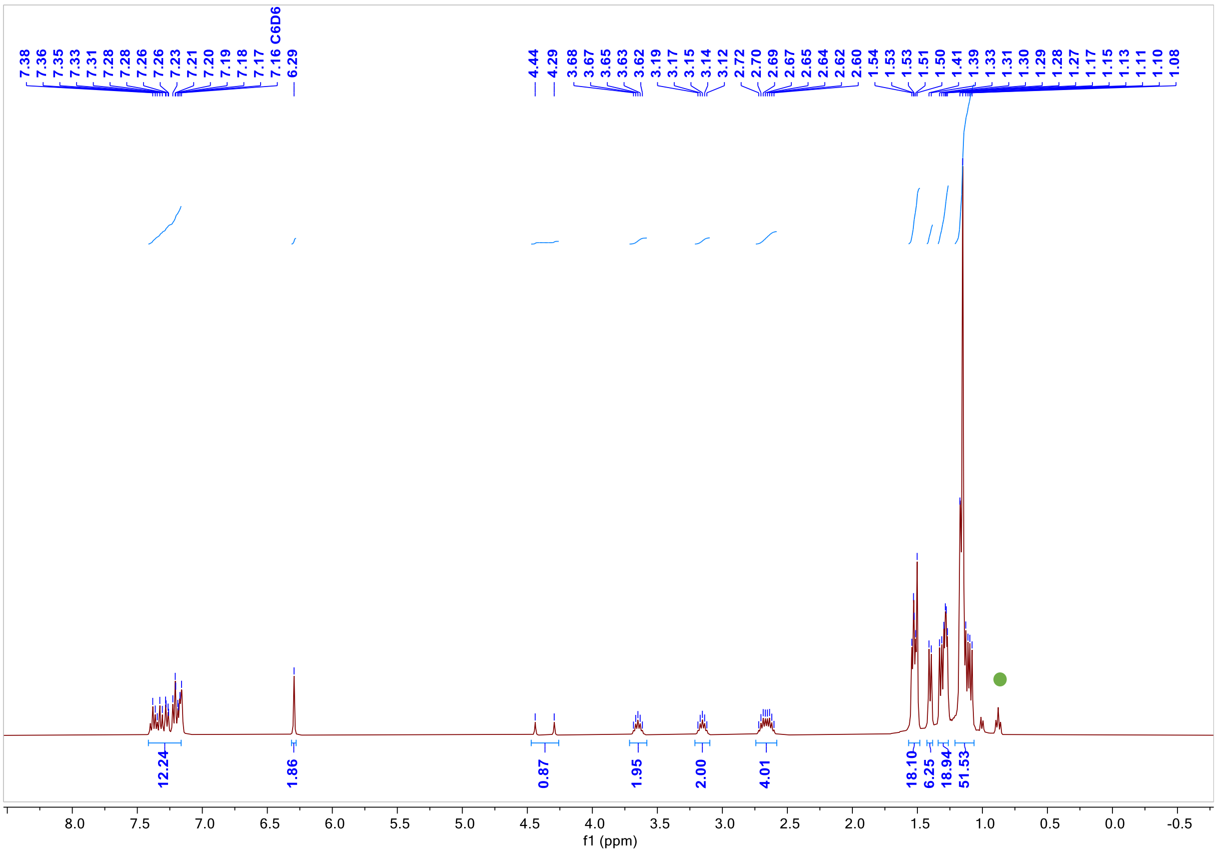

Figure S14.1 ^{1}H NMR spectrum of **15** in C_6D_6 at 300 K (●: pentane).

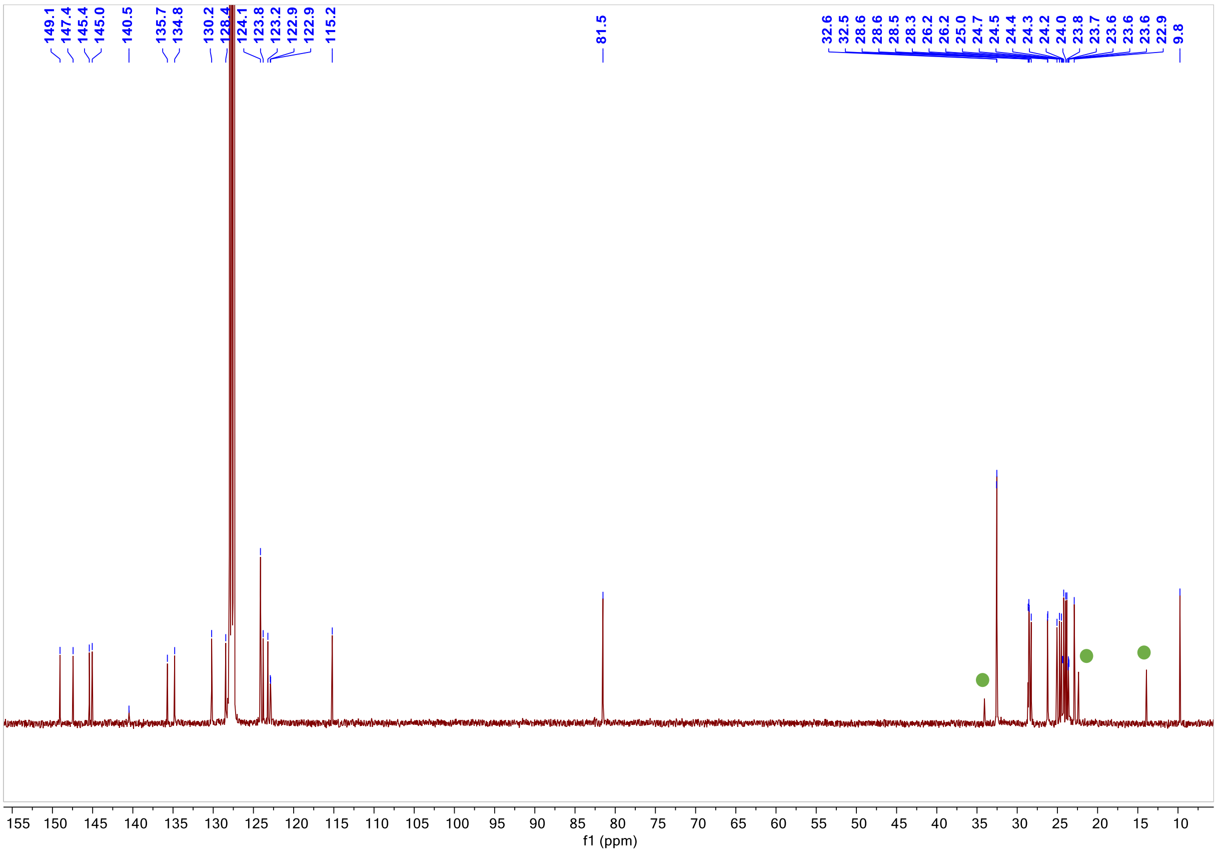

Figure S14.2 $^{13}C\{^{1}H\}$ NMR spectrum of **15** in C_6D_6 at 300 K.

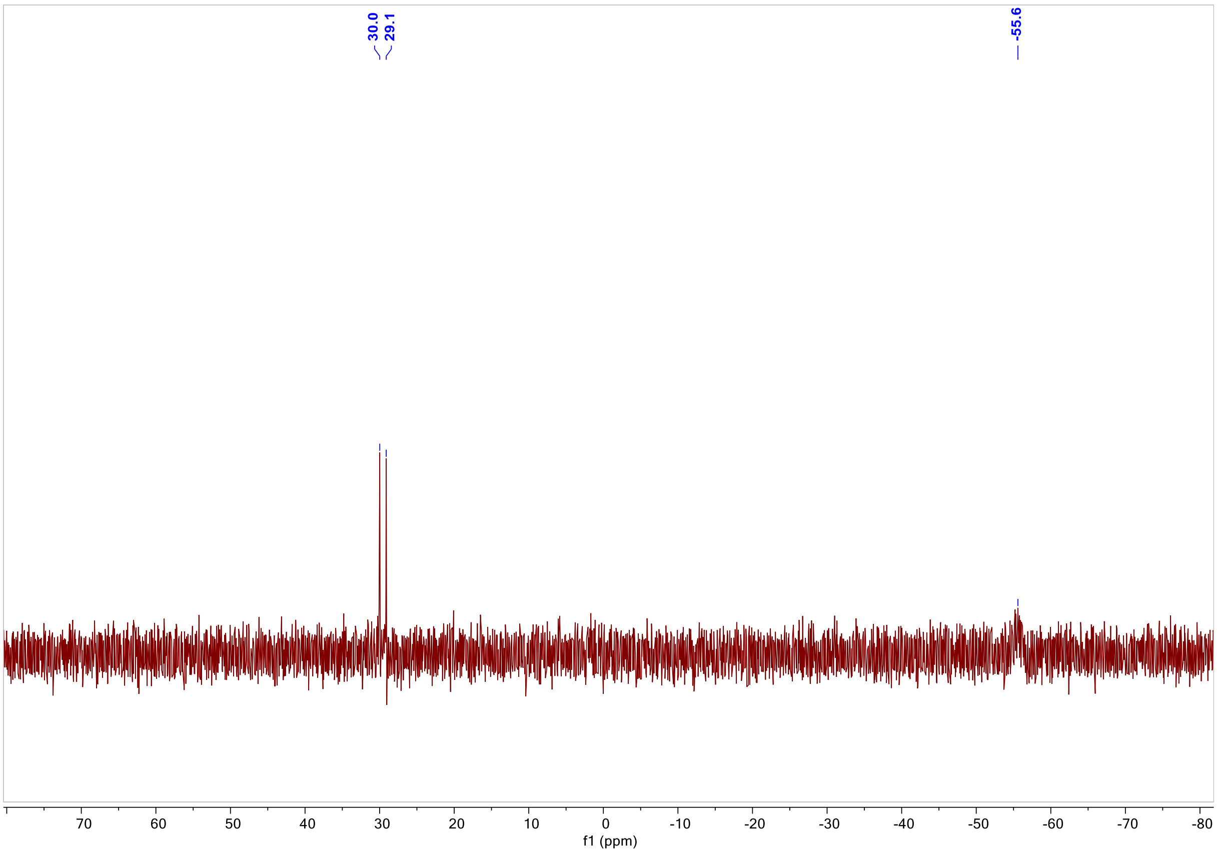

Figure S14.3 $^{29}Si\{^1H\}$ NMR spectrum of **15** in C_6D_6 at 300 K.

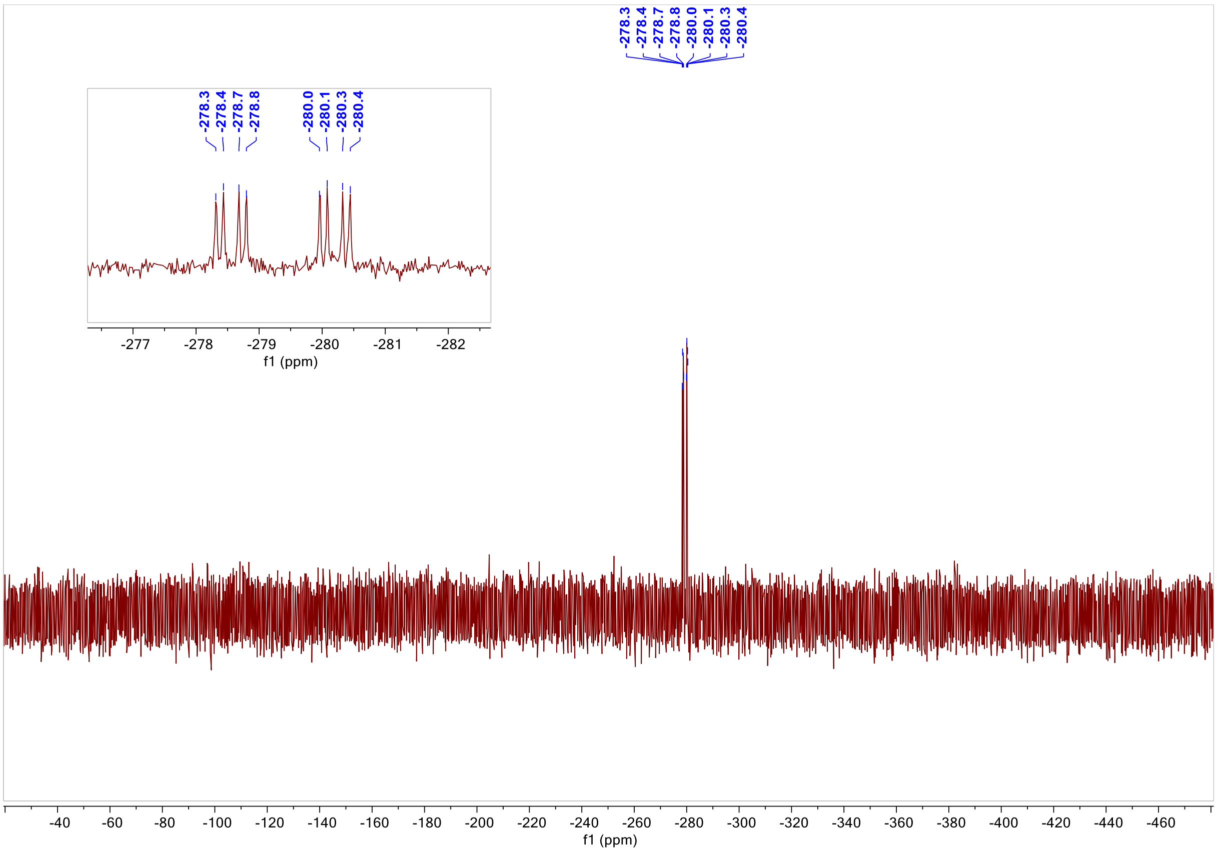

Figure S14.4 ^{31}P NMR spectrum of **15** in C_6D_6 at 300 K.

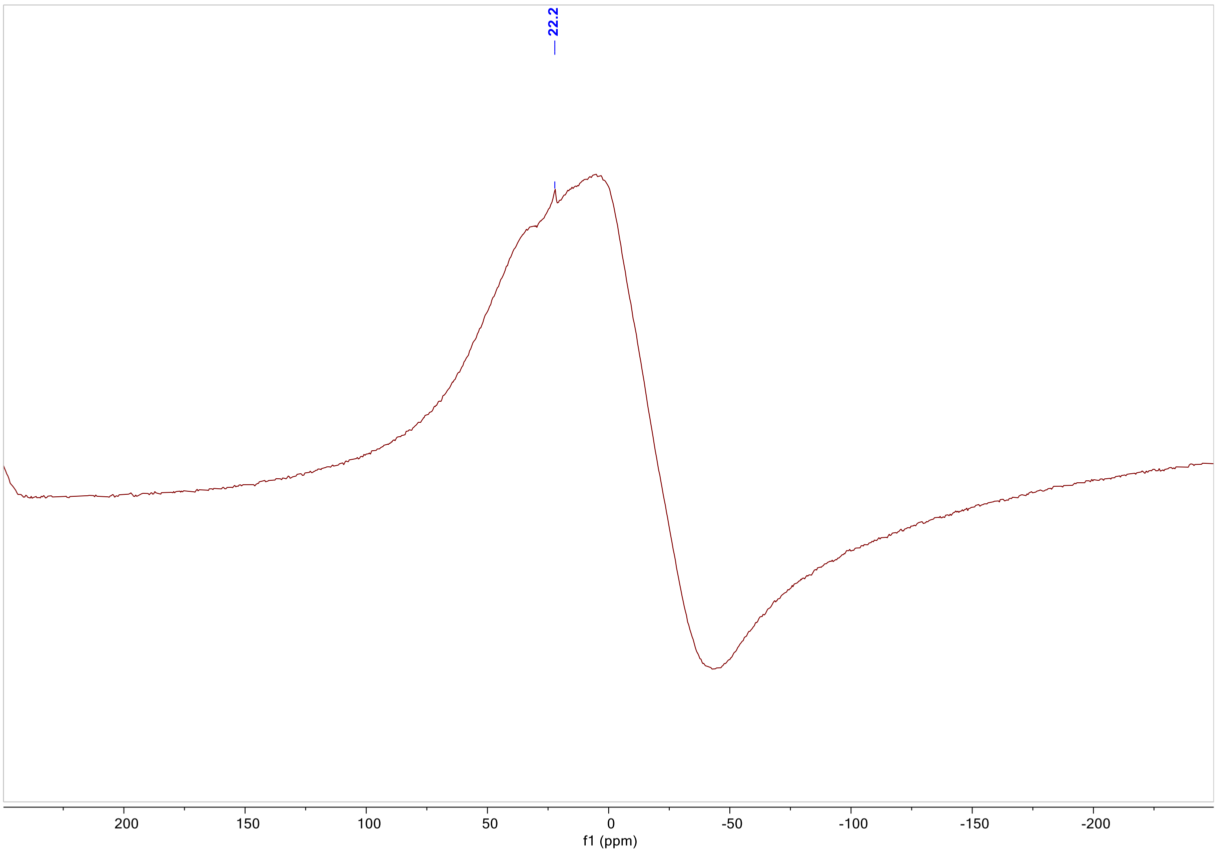

Figure S14.5 ^{11}B NMR spectrum of **15** in C_6D_6 at 300 K.

1.2.15 Synthesis of *Si*-ironiophosphasilene (16)

Si-silveriophosphasilene **4** (23.7 mg, 0.02 mmol) and [CpFe(CO)$_2$]$_2$ (7.1 mg, 0.02 mmol) were combined in benzene (1.5 mL) at room temperature. The deep red mixture was allowed to stir for 24h at room temperature. After work-up, all volatiles were removed *in vacuum* and the residue was extracted with pentane (3 × 1 mL). Pentane solution was concentrated and stored at –30 °C to yield *Si*-ironiophosphasilene **16** (13.5 mg, 78%) as red crystals.

^{1}H NMR (400.1 MHz, C$_6$D$_6$): δ [ppm] 7.18-7.26 (m, 6H, Ar*H*, overlapping with C$_6$D$_6$), 4.08 (s, 5H, Cp*H*), 3.19 (br, 4H, C*H*(CH$_3$)$_2$), 1.62 (d, *J* = 6.6 Hz, 12H, CH(C*H*$_3$)$_2$), 1.56-1.58 (m, 18H, NCC*H*$_3$ and CH(C*H*$_3$)$_2$), 1.41 (s, 27H, C(C*H*$_3$)$_3$), 1.16 (d, *J* = 6.8 Hz, 12H, CH(C*H*$_3$)$_2$).

^{13}C{^{1}H} NMR (100.6 MHz, C$_6$D$_6$): δ [ppm] 213.6 (Fe*C*O), 147.8 (N*C*N), 145.6 (Ar*C*), 132.5 (Ar*C*), 129.5 (Ar*C*), 124.1 (Ar*C*), 117.0 (N*C*-CH$_3$), 84.1 (*Cp*), 32.2 (C(*C*H$_3$)$_3$), 32.2 (C(*C*H$_3$)$_3$), 28.4 (*C*H(CH$_3$)$_2$), 24.5 (CH(*C*H$_3$)$_2$), 24.4 (CH(*C*H$_3$)$_2$), 24.4 (CH(*C*H$_3$)$_2$), 24.3 (*C*(CH$_3$)$_3$), 9.5 (NC-*C*H$_3$).

^{29}Si{^{1}H} NMR (79.5 MHz, C$_6$D$_6$): δ [ppm] 29.1 (d, $J_{Si–P}$ = 109.1 Hz, *Si*^{t}Bu$_3$), 148.9 (d, $J_{Si–P}$ = 184.5 Hz, *Si*=P).

^{31}P NMR (162.0 MHz, C$_6$D$_6$): δ [ppm] 39.7(Si=*P*).

LIFDI-MS: Calcd (-CO): 837.4395; Found: 837.4443.

IR (Fe–CO, cm^{-1}): 2002, 1953.

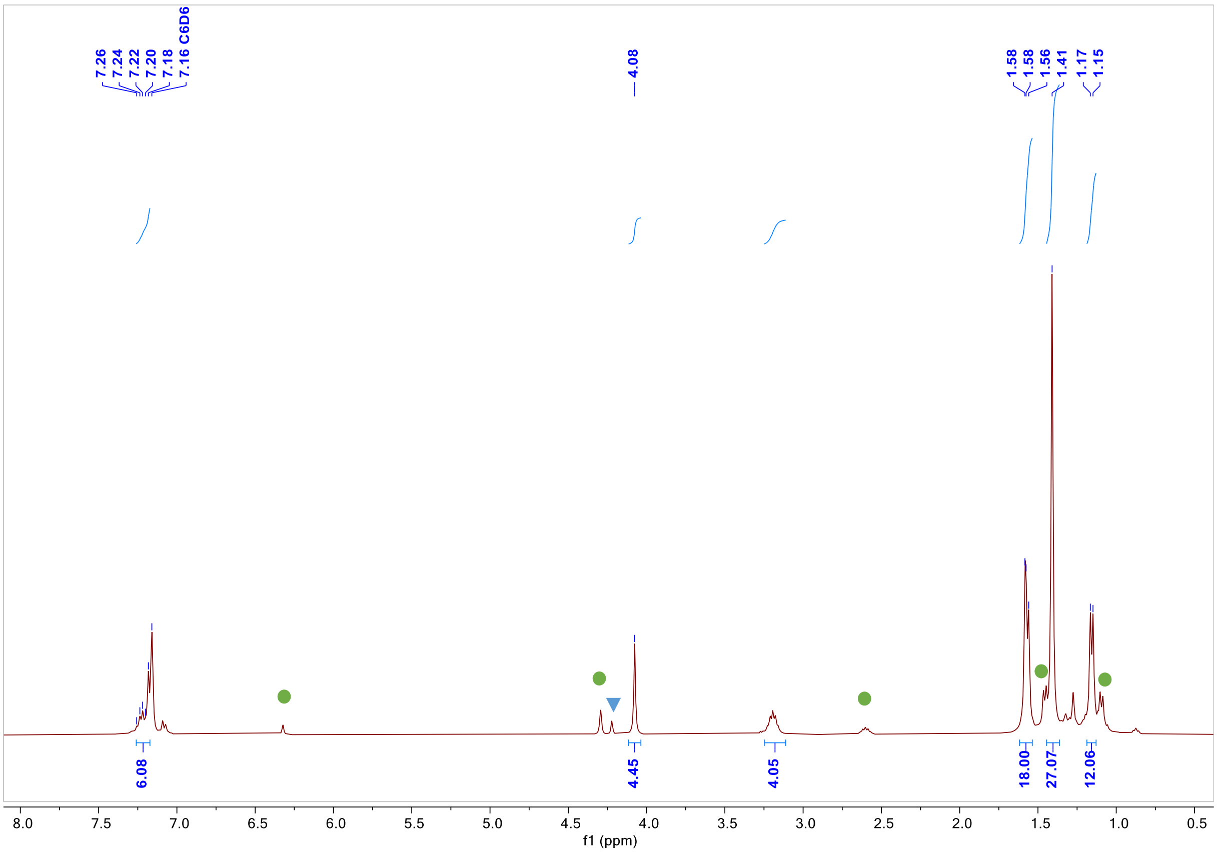

Figure S15.1 ^{1}H NMR spectrum of **16** in C_6D_6 at 300 K (●: IPrAg-CpFe(CO)$_2$; ▼: [CpFe(CO)$_2$]$_2$).

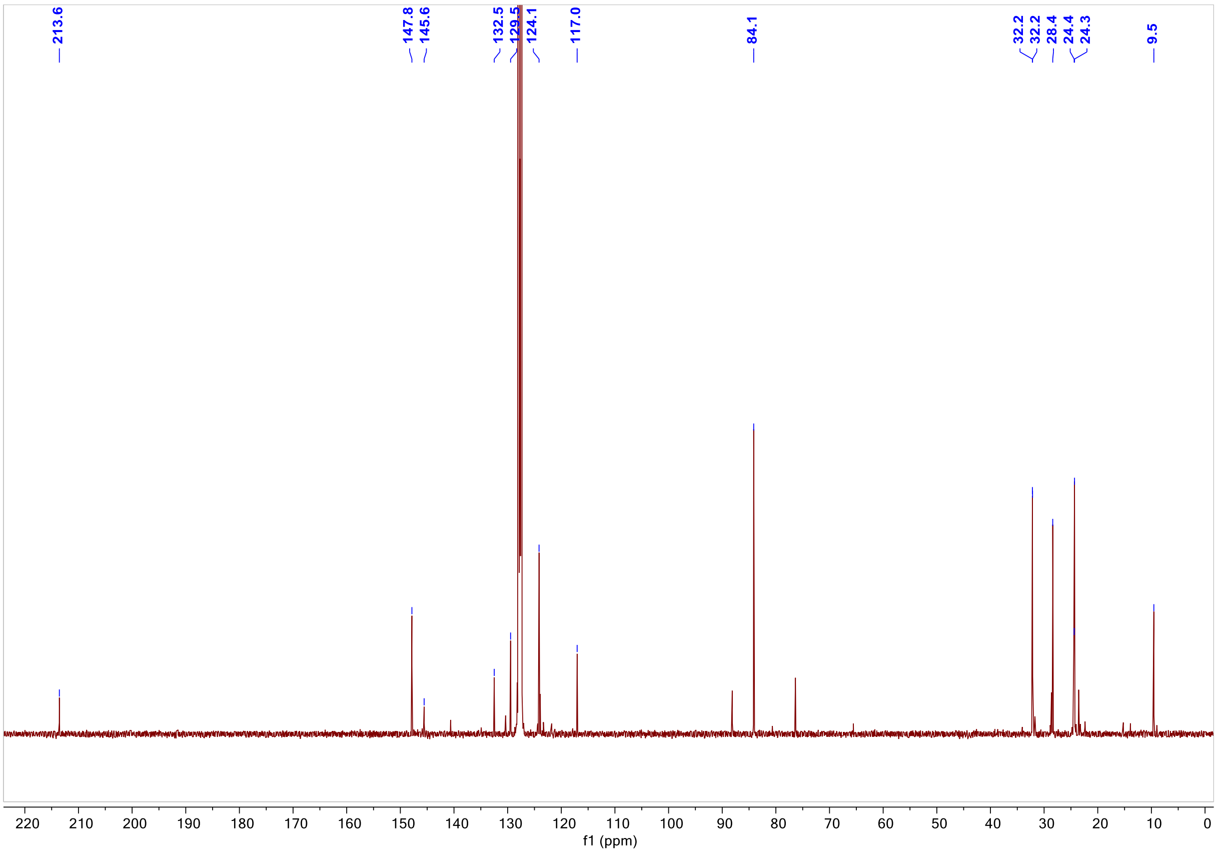

Figure S15.2 $^{13}C\{^{1}H\}$ NMR spectrum of **16** in C_6D_6 at 300 K.

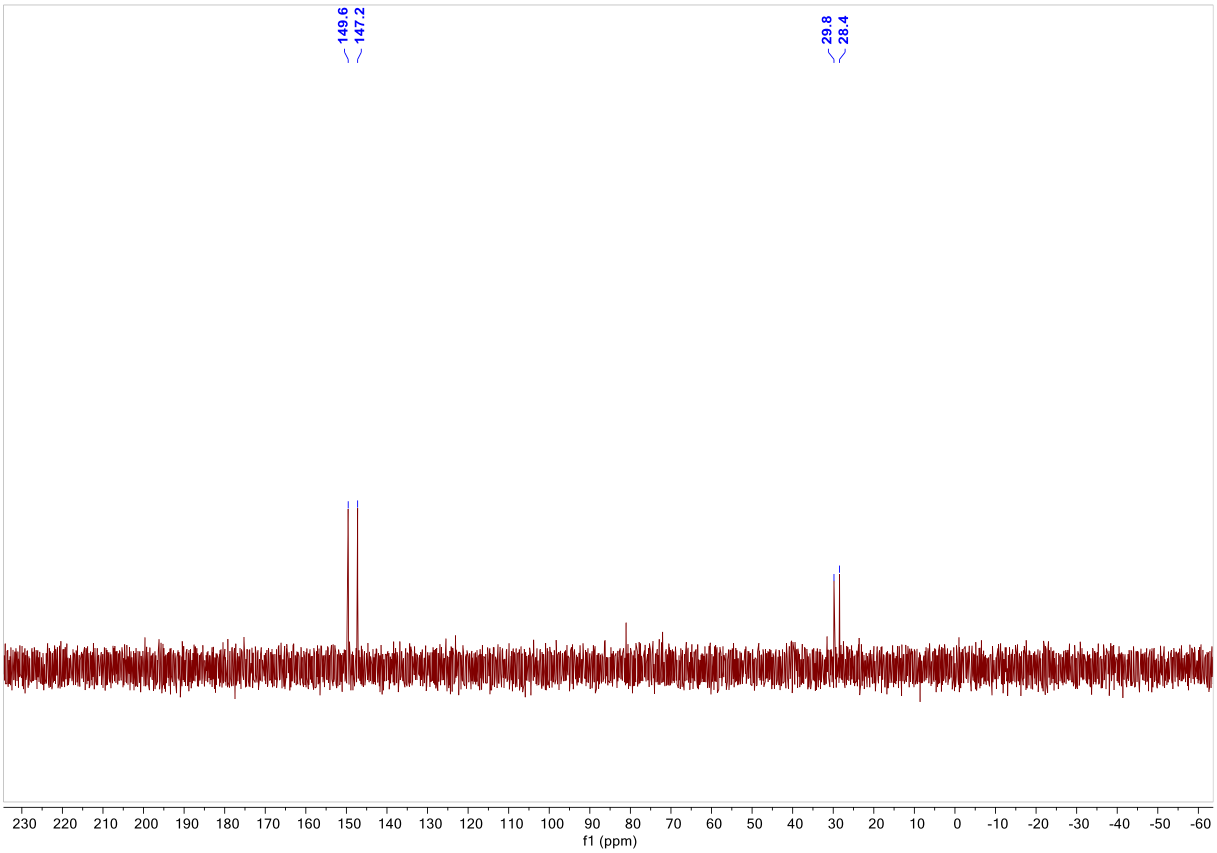

Figure S15.3 $^{29}Si\{^1H\}$ NMR spectrum of **16** in C_6D_6 at 300 K.

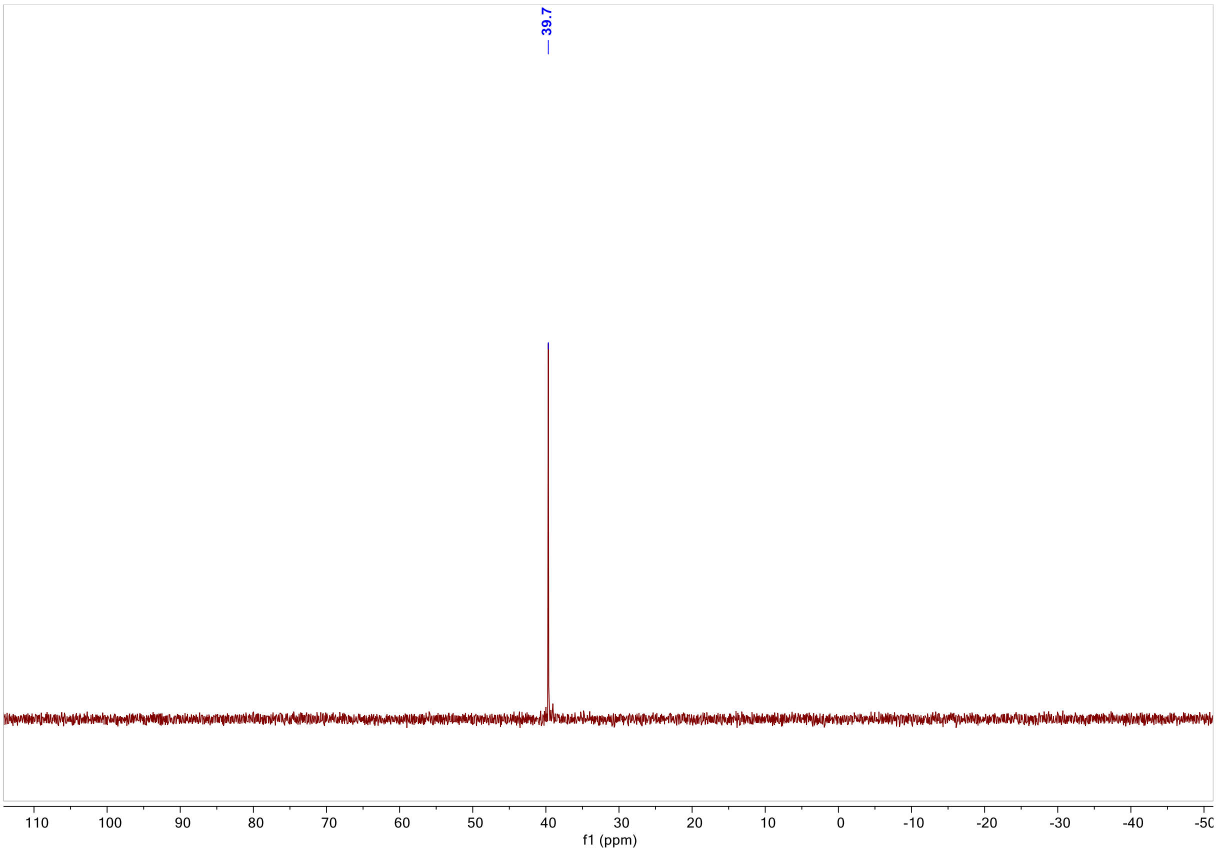

Figure S15.4 ^{31}P NMR spectrum of **16** in C_6D_6 at 300 K.

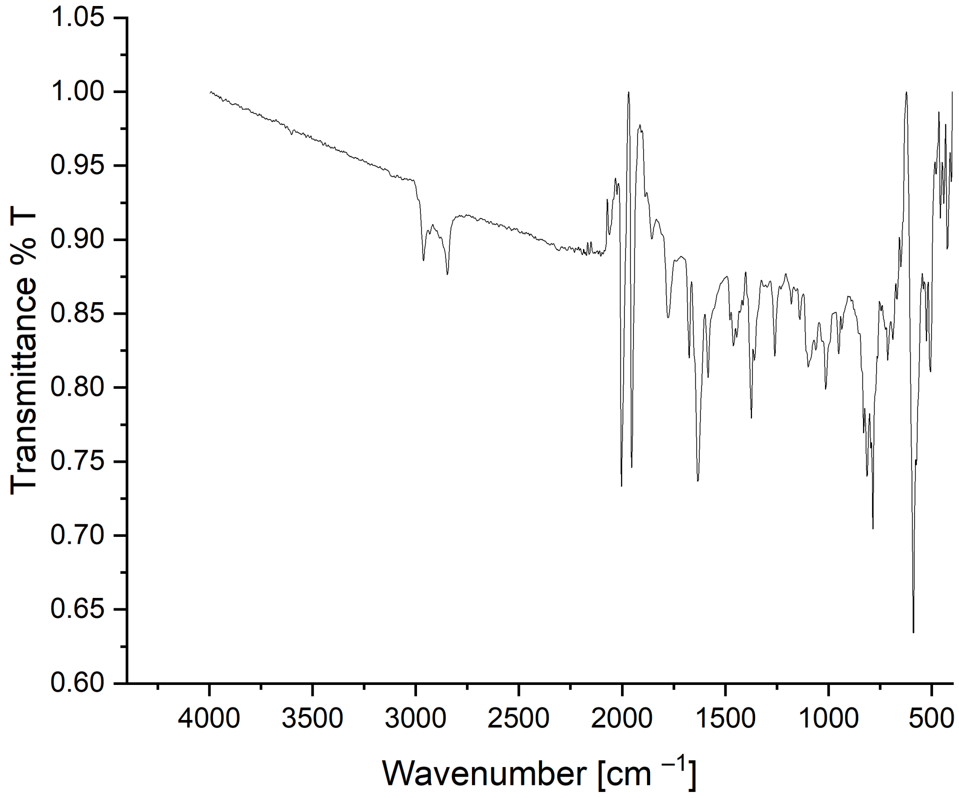

Figure S15.5 IR spectrum of **16**.

1.2.16 Synthesis of Silylene-mangenese complex (17)

Si-silveriophosphasilene **7** (23.7 mg, 0.02 mmol) and $Mn_2(CO)_{10}$ (7.6 mg, 0.02 mmol) were combined in Et_2O (1.5 mL) at room temperature. The color of mixture turned to red rapidly with the release of bubble. The red solution was subsequently stored at –30 °C, in which the orange crystals was formed within 6 h. After removing top solution, the residue was dried *in vacuum* to yield silylene-mangenese complex **17** (29.8 mg, 92%) as an orange power.

^{1}H NMR (400.1 MHz, C_6D_6): δ [ppm] 7.08-7.42 (m, 12H, Ar*H*, overlapping with C_6D_6), 6.30 (s, 2H, NC*H*), 3.80 (br, 1H, C*H*(CH_3)$_2$), 3.44 (br, 1H, C*H*(CH_3)$_2$), 3.28 (br, 1H, C*H*(CH_3)$_2$), 2.60-2.75 (m, 5H, C*H*(CH_3)$_2$), 1.62 (br, 6H, CH(C*H*$_3$)$_2$), 1.54 (s, 6H, NCC*H*$_3$), 1.31-1.38 (m, 18H, CH(C*H*$_3$)$_2$), 1.26-1.31 (m, 39H, CH(C*H*$_3$)$_2$ and C(C*H*$_3$)$_3$), 1.04 (d, *J* = 6.8 Hz, 6H, CH(C*H*$_3$)$_2$), 1.00 (d, *J* = 6.8 Hz, 6H, CH(C*H*$_3$)$_2$).

^{13}C{^{1}H} NMR (100.6 MHz, C_6D_6): δ [ppm] 225.6 (Mn*C*O), 223.7 (Mn*C*O), 146.4 (N*C*N), 145.3 (Ar*C*), 145.2 (Ar*C*), 145.0 (Ar*C*), 145.0 (Ar*C*), 141.6 (Ar*C*), 136.0 (Ar*C*), 130.4 (Ar*C*), 125.2 (Ar*C*), 124.5 (Ar*C*), 124.3 (ArC), 124.2 (ArC), 124.1 (N*C*H), 124.0 (N*C*H), 118.5 (N*C*-CH_3), 32.3 (C(*C*H$_3$)$_3$), 32.3 (C(*C*H$_3$)$_3$), 31.6 (C(*C*H$_3$)$_3$), 28.9 (*C*H(CH_3)$_2$), 28.6 (*C*H(CH_3)$_2$), 28.5 (*C*H(CH_3)$_2$), 28.5 (*C*H(CH_3)$_2$), 28.4 (*C*H(CH_3)$_2$), 28.2 (*C*H(CH_3)$_2$), 24.5 (CH(*C*H$_3$)$_2$), 24.4 (*C*(CH_3)$_3$), 24.3 (CH(*C*H$_3$)$_2$), 24.0 (CH(*C*H$_3$)$_2$), 24.0 (CH(*C*H$_3$)$_2$), 23.9 (CH(*C*H$_3$)$_2$), 23.6 (CH(*C*H$_3$)$_2$), 23.5 (CH(*C*H$_3$)$_2$), 23.2 (CH(*C*H$_3$)$_2$), 8.9 (NC-*C*H$_3$).

^{29}Si{^{1}H} NMR (79.5 MHz, C_6D_6): δ [ppm] 39.1 (m, *Si*^{t}Bu$_3$), 241.0 (m, P=*Si*Mn).

^{31}P NMR (162.0 MHz, C_6D_6): δ [ppm] –154.0 (dd, ^{107}Ag: $J_{P–Ag}$ = 213.5 Hz and ^{109}Ag: $J_{P–Ag}$ = 244.9 Hz, Si*P*Ag).

LIFDI-MS: Calcd (-(CO)$_5$): 1405.5098; Found: 1405.5056.

IR (Mn–CO, cm^{-1}): 2072, 2045, 2033, 1996, 1965, 1938, 1918.

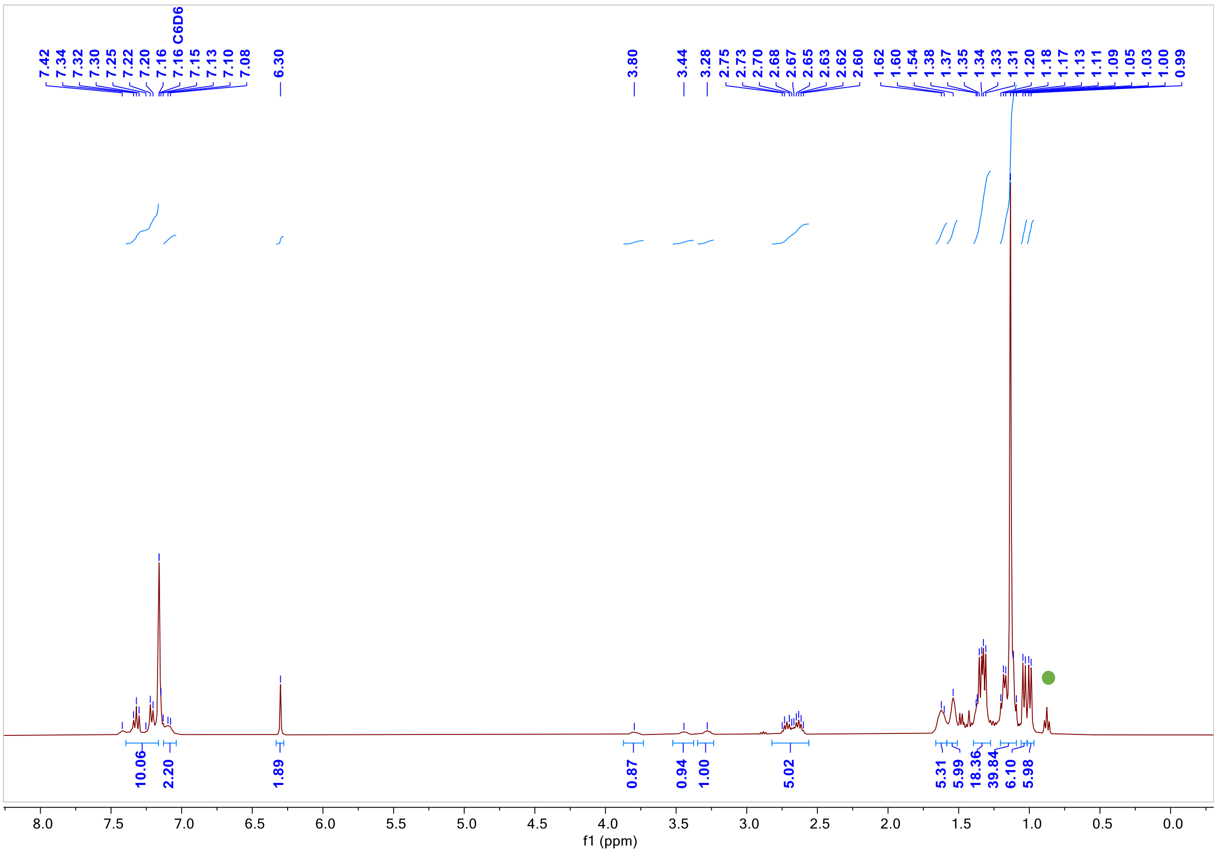

Figure S16.1 ^{1}H NMR spectrum of **17** in C_6D_6 at 300 K(●: pentane).

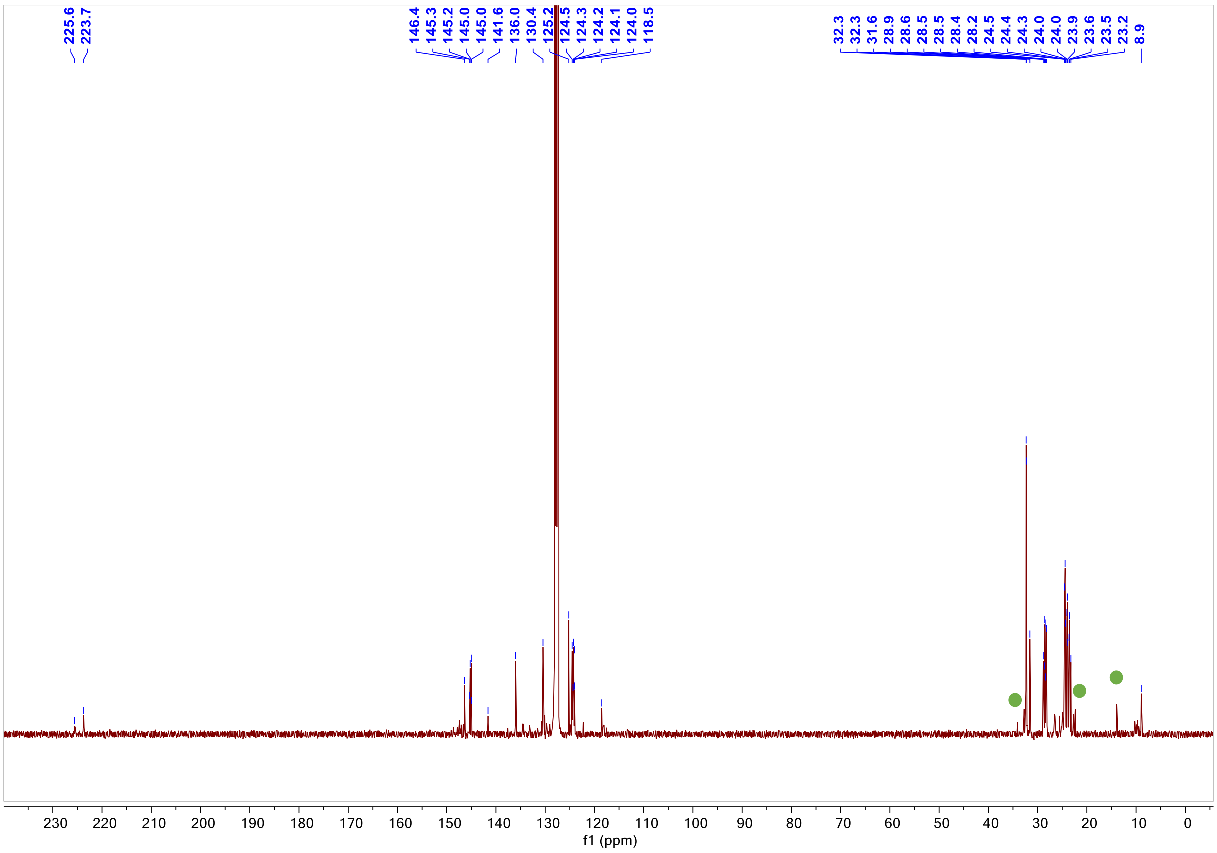

Figure S16.2 ^{13}C NMR spectrum of **17** in C_6D_6 at 300 K (●: pentane).

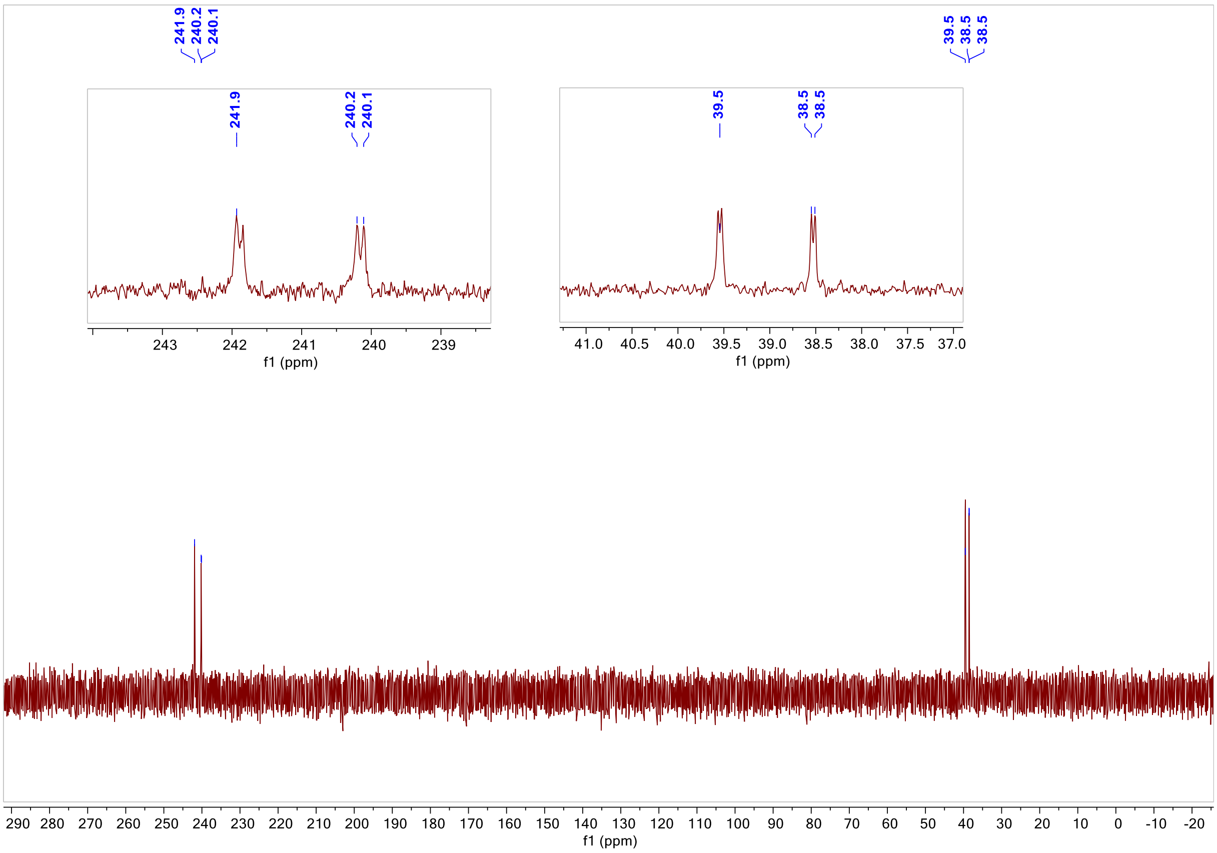

Figure S16.3 ^{29}Si{^{1}H} NMR spectrum of **17** in C_6D_6 at 300 K.

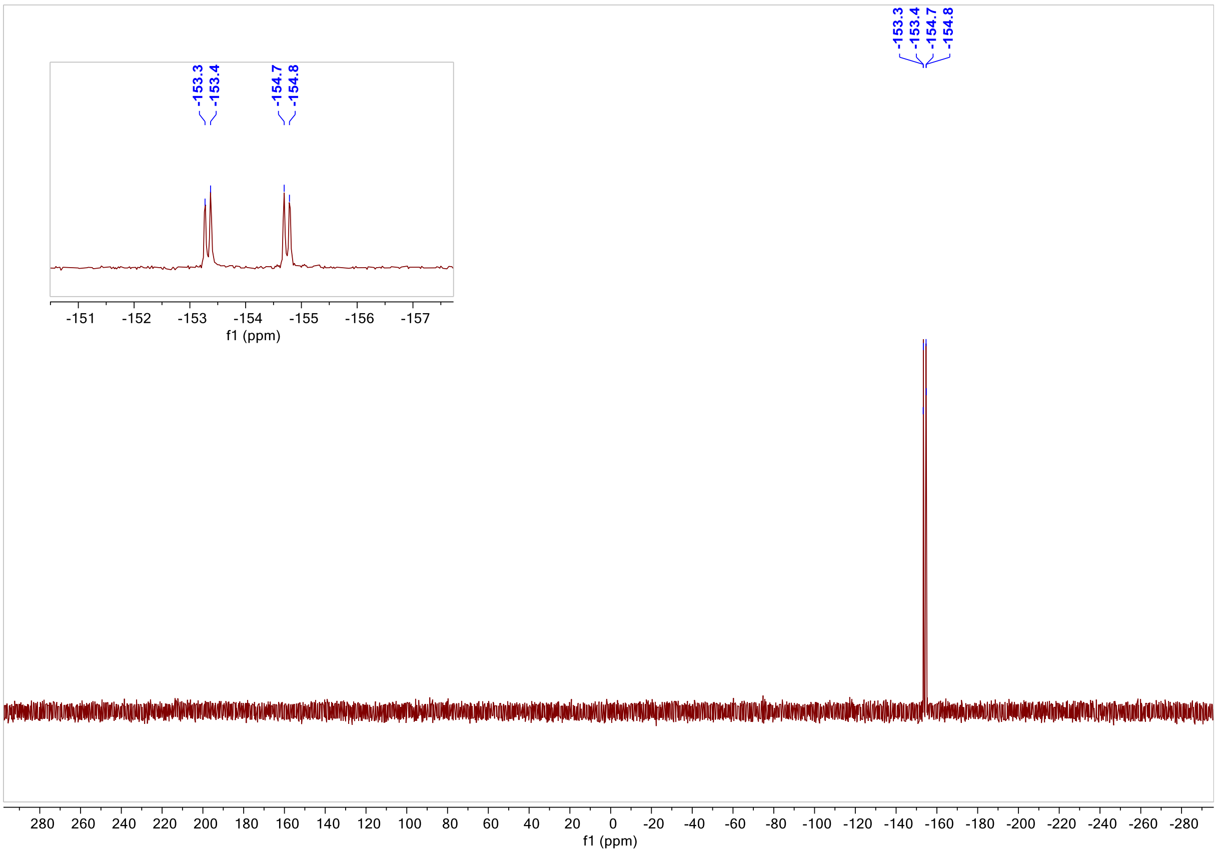

Figure S16.4 ^{31}P NMR spectrum of **17** in C_6D_6 at 300 K.

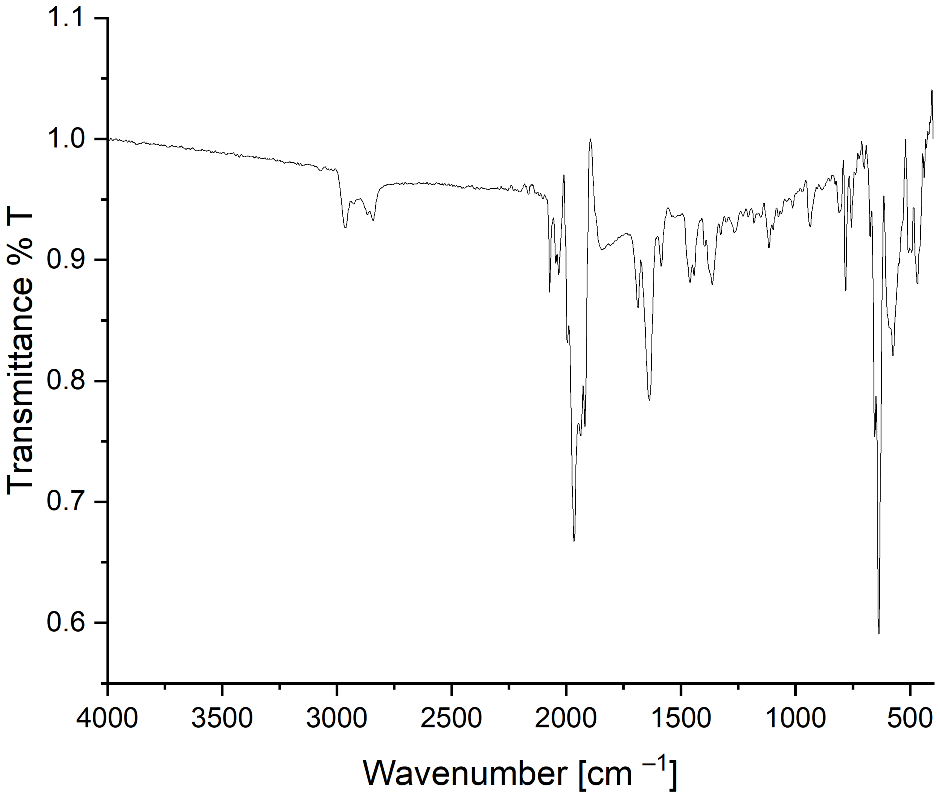

Figure S16.5 IR spectrum of **17**.

1.2.17 Synthesis of *Si*-mangeneseiophosphasilene (18)

A benzene solution of silylene-mangenese complex **17** (29.8 mg, 0.018 mmol) was heated to 60 °C for 6 h. The color of mixture turned to light yellow gradually from red. After work-up, all volatiles were removed in vacuum. The light orange residue was washed with pentane and dried *in vacuum* to yield *Si*-mangeneseiophosphasilene **18** (10.6 mg, 64%) as a yellow powder. Crystal suitable for single crystal X-ray diffraction analysis was obtained by storing a saturated pentane solution at –30 °C for 2 days.

^{1}H NMR (400.1 MHz, C_6D_6): δ [ppm] 7.27-7.31 (m, 2H, Ar*H*), 7.18 (d, *J* = 7.6 Hz, 4H, Ar*H*, overlapping with C_6D_6), 2.80 (sept, *J* = 6.8 Hz, 4H, C*H*(CH_3)$_2$, 1.46 (d, *J* = 6.8 Hz, 6H, CH(C*H*$_3$)$_2$), 1.41 (s, 6H, NCC*H*$_3$), 1.21 (s, 27H, C(C*H*$_3$)$_3$), 1.09 (d, *J* = 7.2 Hz, 6H, CH(C*H*$_3$)$_2$).

^{13}C{^{1}H} NMR (100.6 MHz, C_6D_6): δ [ppm] 224.6 (Mn*C*O), 146.6 (N*C*N), 146.4 (Ar*C*), 134.5 (Ar*C*), 130.4 (Ar*C*), 130.1 (Ar*C*), 125.2 (Ar*C*), 118.5 (N*C*-CH_3), 31.6 (C(*C*H$_3$)$_3$), 28.9 (*C*H(CH_3)$_2$), 24.9 (CH(*C*H$_3$)$_2$), 24.9 (CH(*C*H$_3$)$_2$), 24.0 (CH(*C*H$_3$)$_2$), 23.5 (CH(*C*H$_3$)$_2$), 23.2 (*C*(CH_3)$_3$), 10.7 (NC-*C*H$_3$).

^{29}Si{^{1}H} NMR (79.5 MHz, C_6D_6): δ [ppm] 32.8 (d, $J_{Si–P}$ = 108.4 Hz, *Si*^{t}Bu$_3$), 245.0 (d, $J_{Si–P}$ = 223.2 Hz, *Si*=P).

^{31}P NMR (162.0 MHz, C_6D_6): δ [ppm] –496.5 (Si=*P*).

LIFDI-MS: Calcd (-(Me)$_3$): 810.3084; Found: 810.3173.

IR (Mn–CO, cm^{-1}): 2043, 2025, 1980, 1922.

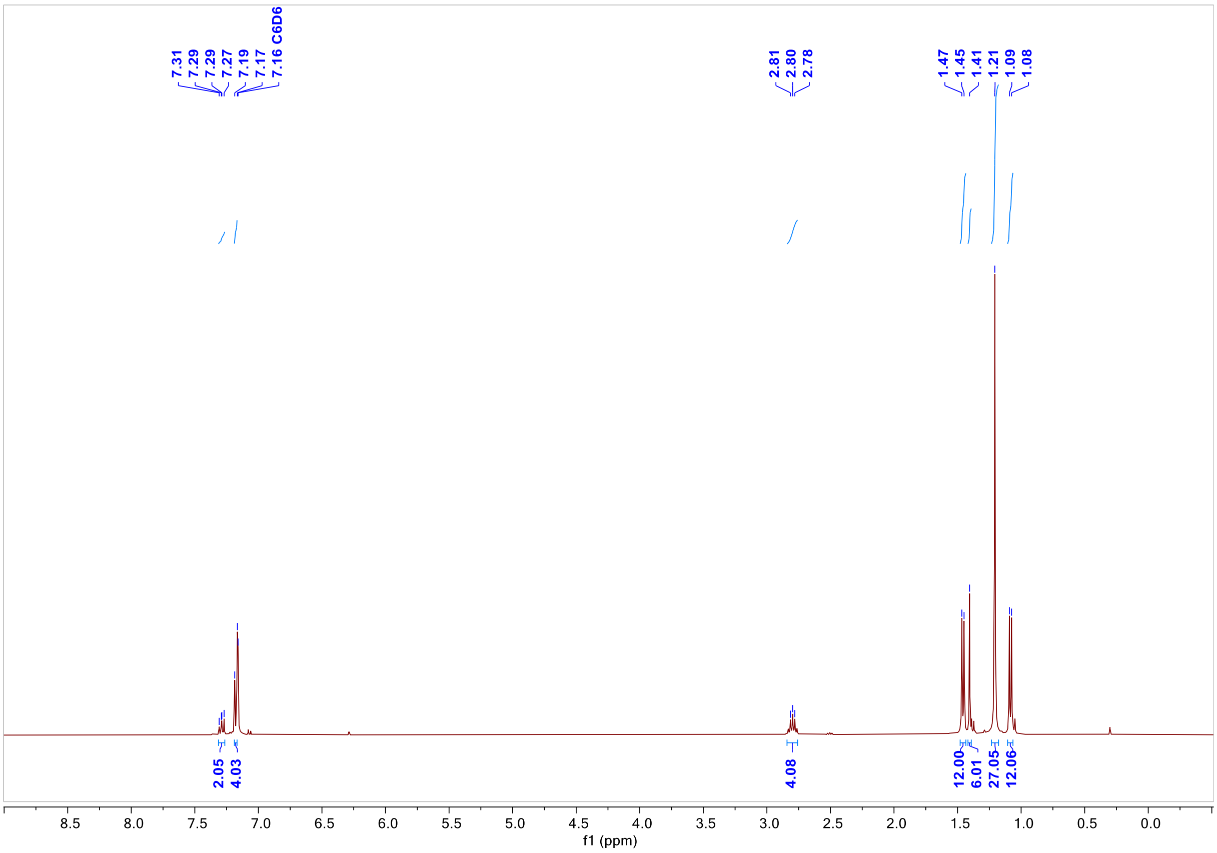

Figure S17.1 ^{1}H NMR spectrum of **18** in C_6D_6 at 300 K.

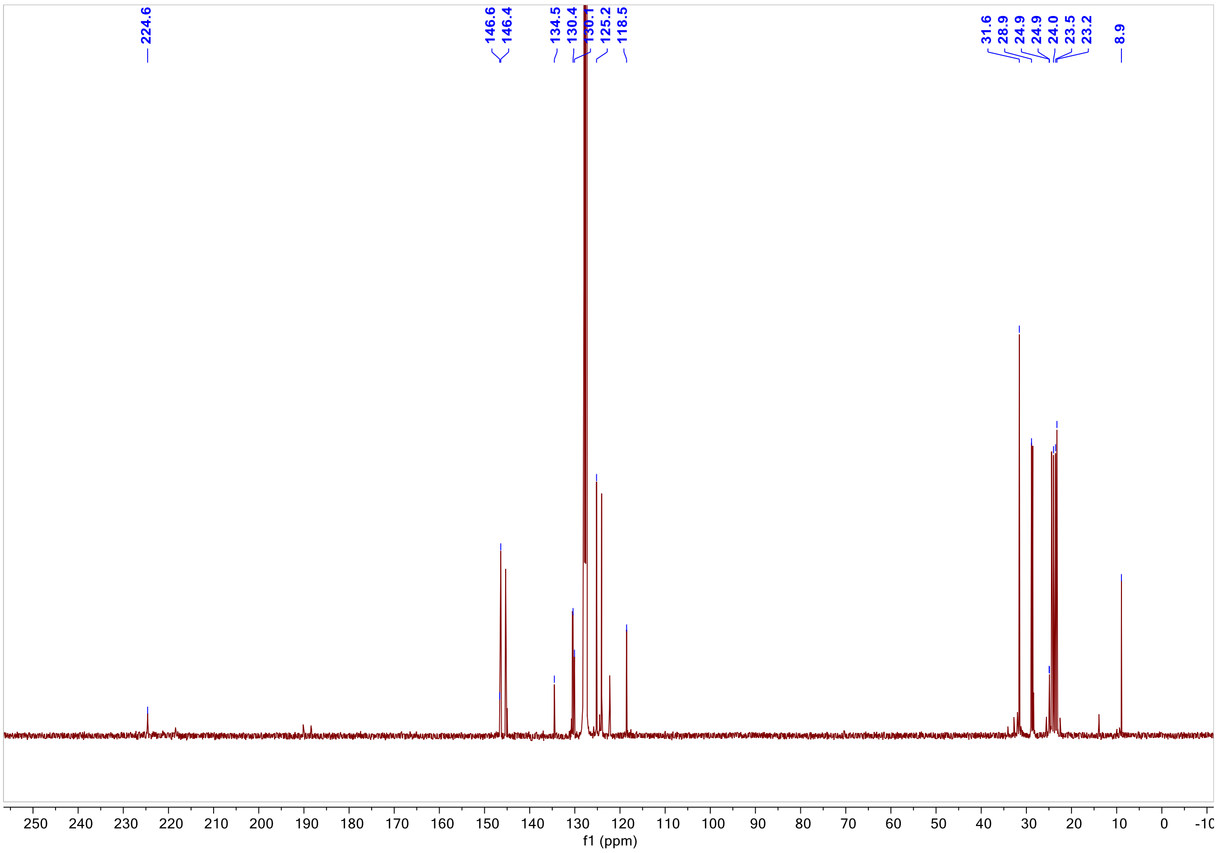

Figure S17.2 ^{13}C NMR spectrum of **18** in C_6D_6 at 300 K.

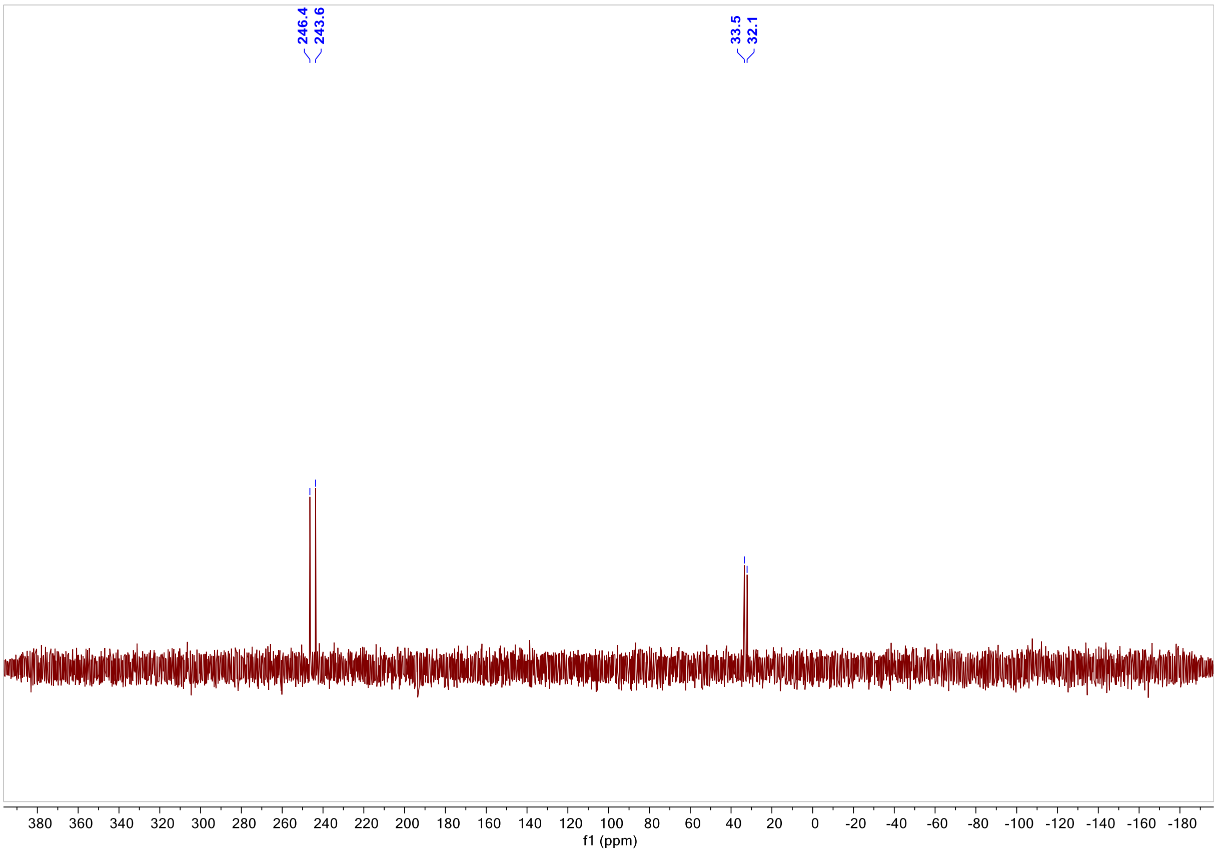

Figure S17.3 $^{29}Si\{^1H\}$ NMR spectrum of **18** in C_6D_6 at 300 K.

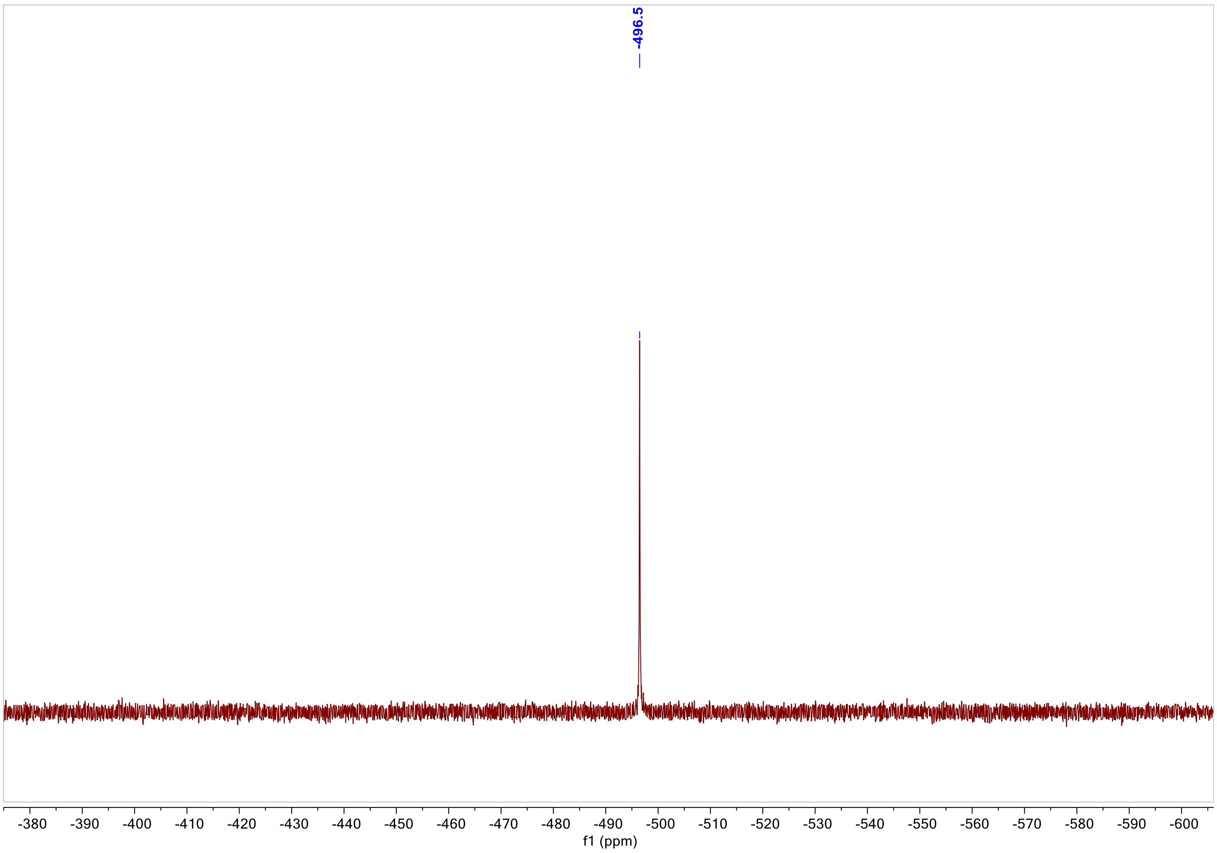

Figure S17.4 ^{31}P NMR spectrum of **18** in C_6D_6 at 300 K.

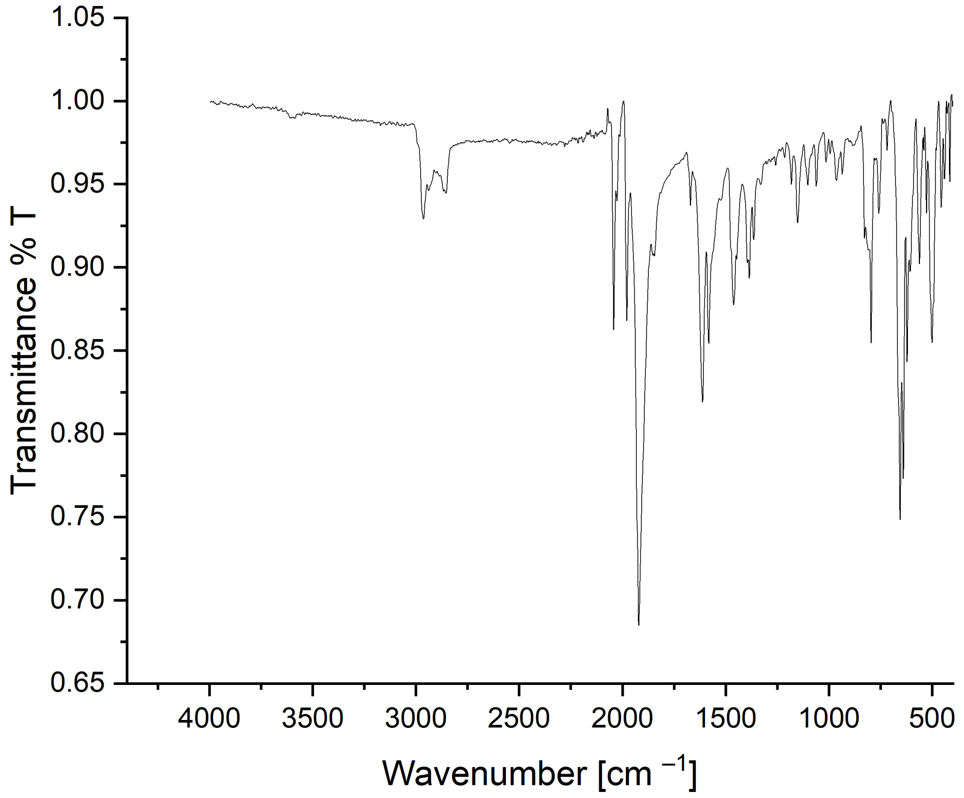

Figure S17.5 IR spectrum of **18**.

1.2.18 Synthesis of *Si*-cobaltiophosphasilene (19)

Si-silveriophosphasilene **4** (23.7 mg, 0.02 mmol) and $Co_2(CO)_8$ (6.8 mg, 0.02 mmol) were combined in benzene (1.5 mL) at room temperature. The color of mixture changed to deep brown rapidly with the release of bubble. After work-up, all volatiles were removed *in vacuum*. The dark residue was washed with pentane to yield *Si*-cobaltiophosphasilene **19** (10.2 mg, 61%) as a brown powder.

^{1}H NMR (400.1 MHz, C_6D_6): δ [ppm] 7.12-7.27 (m, 6H, Ar*H*, overlapping with C_6D_6), 3.01 (br, 2H, C*H*(CH_3)$_2$), 2.66 (br, 2H, C*H*(CH_3)$_2$), 1.49 (br, 6H, CH(CH_3)$_2$), 1.36-1.39 (m, 12H, NCCH_3 and CH(CH_3)$_2$), 1.26 (s, 27H, C(CH_3)$_3$), 1.14 (br, 6H, CH(CH_3)$_2$), 1.00 (br, 6H, CH(CH_3)$_2$).

^{13}C{^{1}H} NMR (100.6 MHz, C_6D_6): δ [ppm] 208.0 (Co*C*O), 145.3 (N*C*N), 144.8 (Ar*C*), 134.5 (Ar*C*), 130.7 (Ar*C*), 124.1 (Ar*C*), 118.8 (N*C*-CH_3), 31.6 (C(*C*H_3)$_3$), 31.6 (C(*C*H_3)$_3$), 28.8 (*C*H(CH_3)$_2$), 28.6 (*C*H(CH_3)$_2$), 25.5 (CH(*C*H_3)$_2$), 25.4 (CH(*C*H_3)$_2$), 24.4 (CH(*C*H_3)$_2$), 23.6 (*C*(CH_3)$_3$), 9.0 (NC-*C*H_3).

^{29}Si{^{1}H} NMR (79.5 MHz, C_6D_6): δ [ppm] 26.9 (d, $J_{Si–P}$ = 111.4 Hz, *Si*^{t}Bu$_3$), 186.7 (d, $J_{Si–P}$ = 261.4 Hz, *Si*=P).

^{31}P NMR (162.0 MHz, C_6D_6): δ [ppm] –521.9 (Si=*P*).

LIFDI-MS: Calcd (-(CO)$_2$): 775.3992; Found: 775.4020.

IR (Co–CO, cm^{-1}): 2019, 1959, 1934.

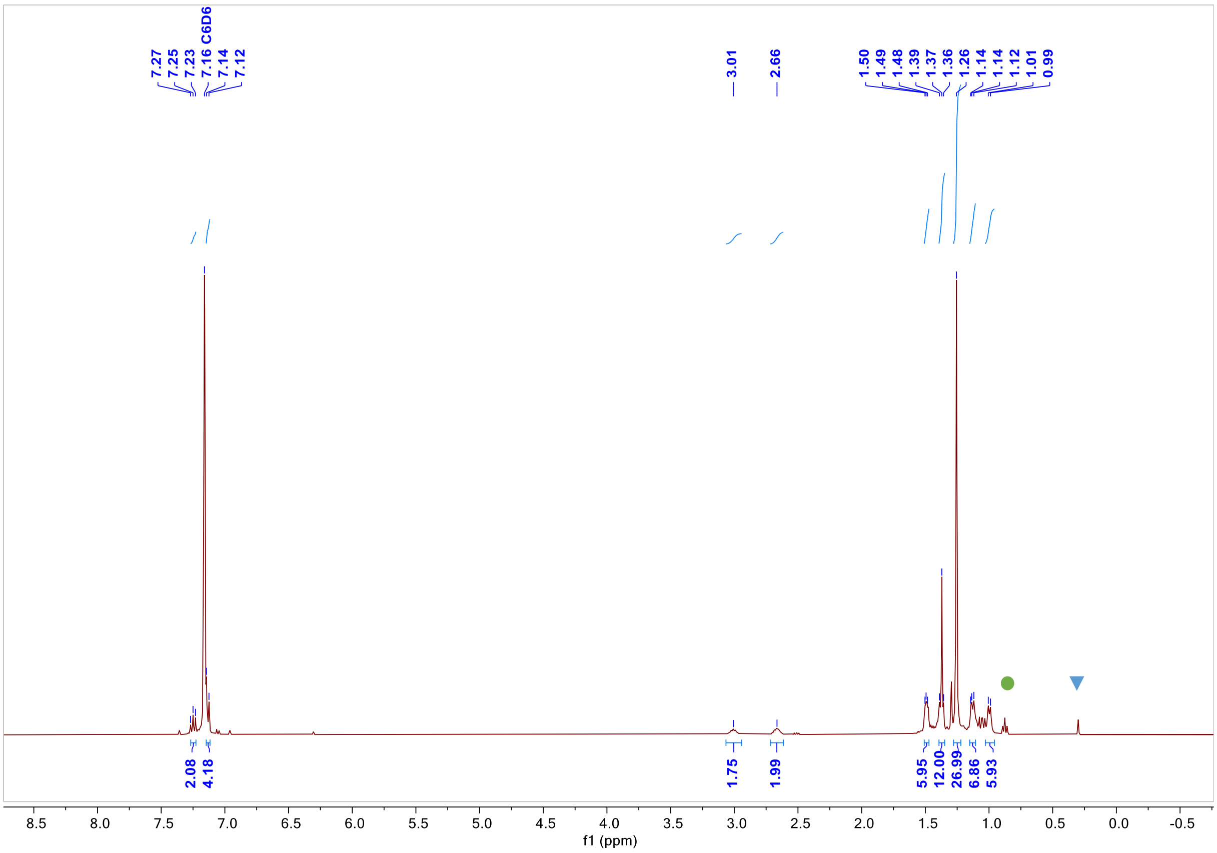

Figure S18.1 ^{1}H NMR spectrum of **19** in C_6D_6 at 300 K (●: pentane; ▼: silicon grease).

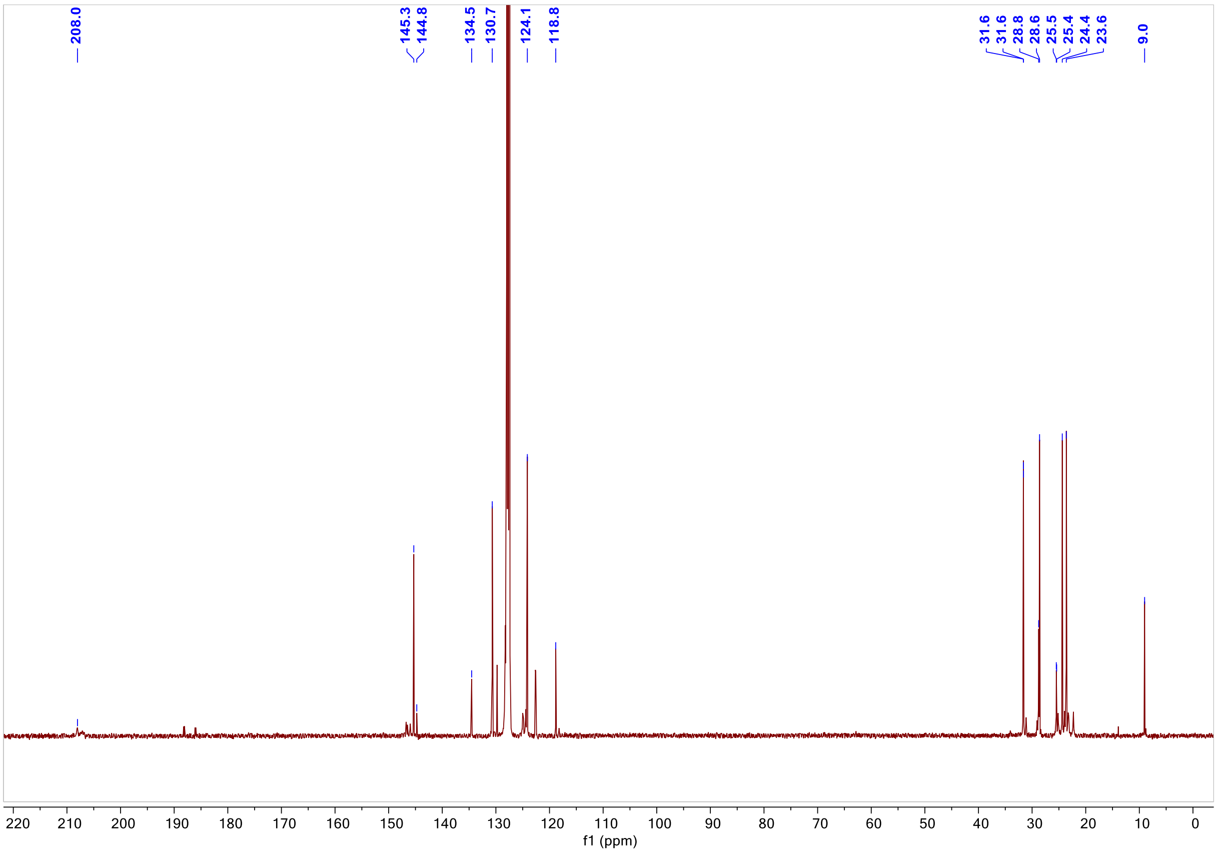

Figure S18.2 ^{13}C NMR spectrum of **19** in C_6D_6 at 300 K.

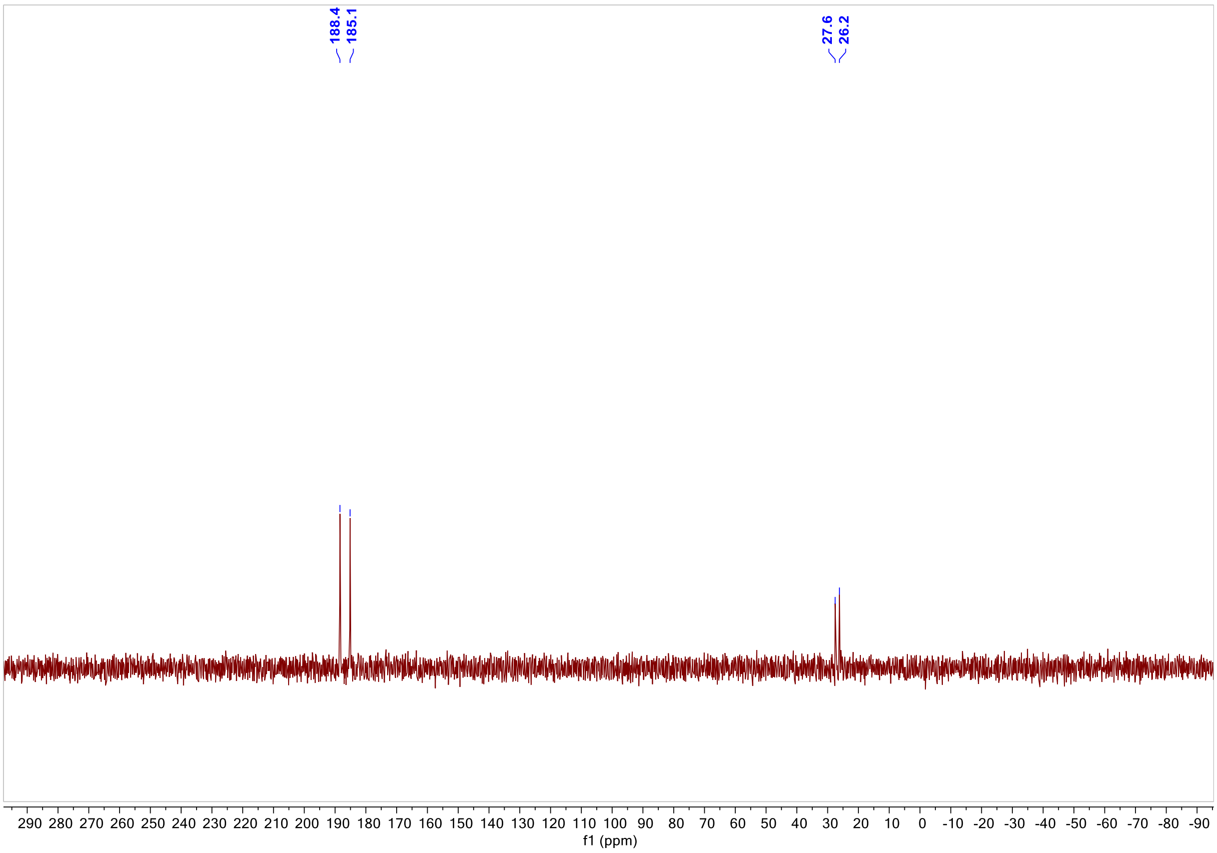

Figure S18.3 $^{29}Si\{^{1}H\}$ NMR spectrum of **19** in C_6D_6 at 300 K.

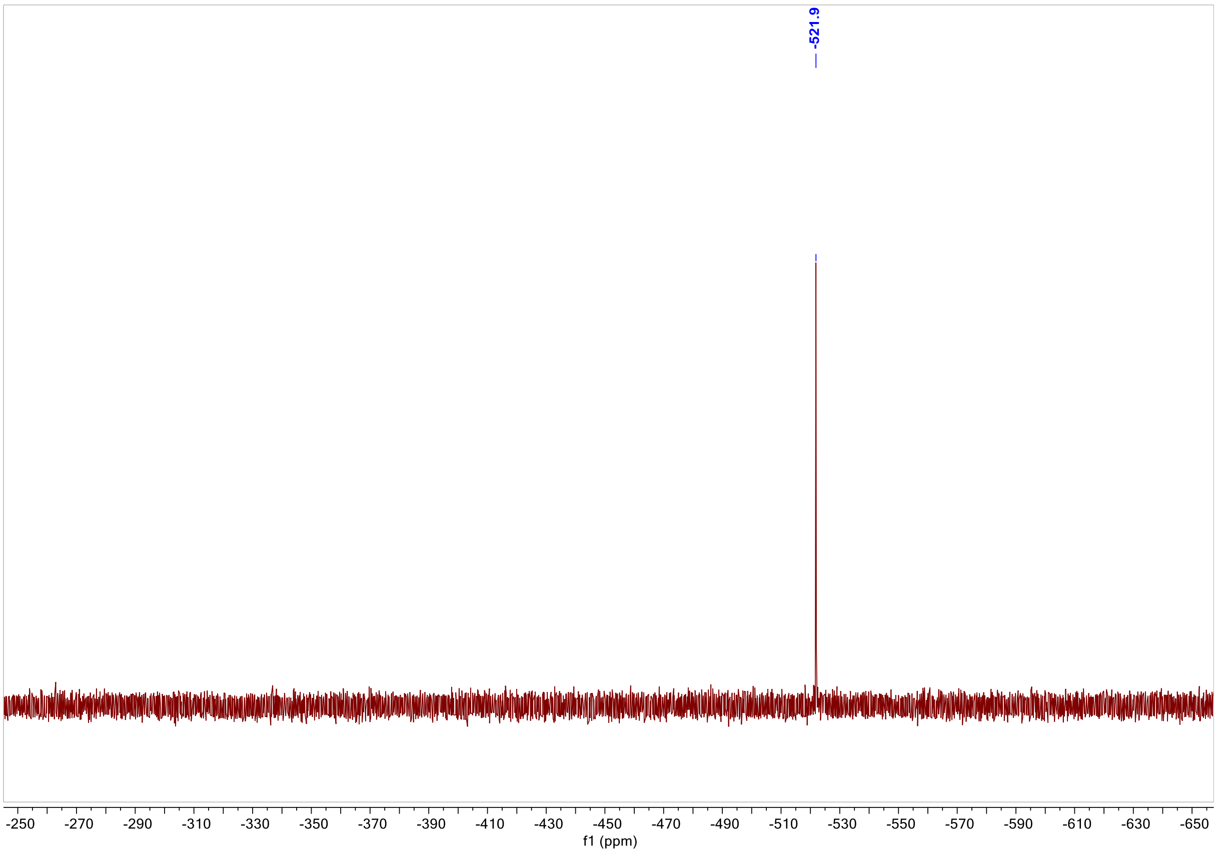

Figure S18.4 ^{31}P NMR spectrum of **19** in C_6D_6 at 300 K.

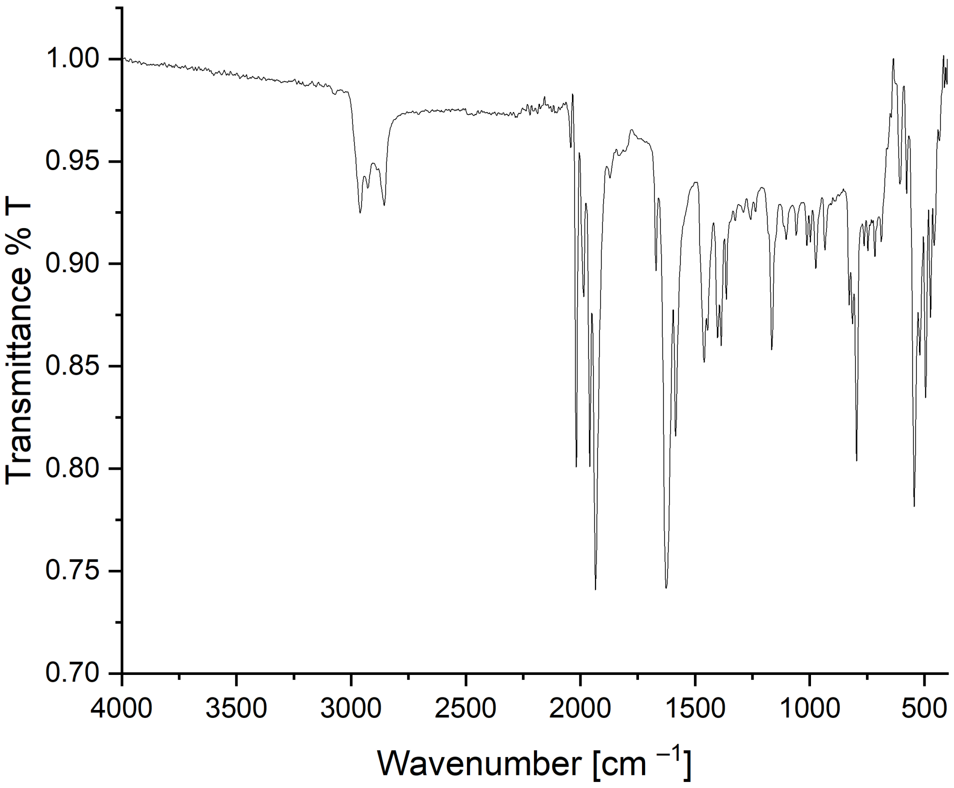

Figure S18.5 IR spectrum of **19**.

2. Single Crystal X-Ray Structure Determination

Single crystal diffraction data were recorded on a Bruker Photon D8 Venture DUO IMS system equipped with a Helios optic monochromator and a Mo IMS microsource (λ = 0.71073 Å) and an Atlas SuperNova system equipped with a mirror monochromator and a Cu micro-focus sealed X-ray tube (λ = 1.54178 Å). The data collection was performed, using the APEX IV software package[S7] and CrysAlisPro[S8] on single crystals coated with Fomblin®Y as perfluorinated ether. The single crystal was picked on a micro sampler, transferred to the diffractometer, and measured frozen under a stream of cold nitrogen or at room temperature. A matrix scan was used to determine the initial lattice parameters. Reflections were merged and corrected for Lorenz and polarization effects, scan speed, and background using SAINT.[S9] Absorption corrections, including odd and even ordered spherical harmonics were performed using SADABS.[S9] Space group assignments were based upon systematic absences, E statistics, and successful refinement of the structures. Structures were solved by direct methods with the aid of successive difference Fourier maps and were refined against all data using the APEX IV software in conjunction with SHELXL-2014[S10] and SHELXLE.[S11] H atoms were placed in calculated positions and refined using a riding model, with methylene and aromatic C–H distances of 0.99 and 0.95 Å, respectively, and Uiso(H) = 1.2·Ueq(C). Non-hydrogen atoms were refined with anisotropic displacement parameters. Full-matrix least-squares refinements were carried out by minimizing $\Sigma w(Fo^2\text{-}Fc^2)^2$ with the SHELXL weighting scheme.[S12] Neutral atom scattering factors for all atoms and anomalous dispersion corrections for the non-hydrogen atoms were taken from International Tables for Crystallography.[S13] The images of the crystal structures were generated by Mercury.[S14] The CCDC numbers 2424011-2424016 and 2480971-2480977 contain the supplementary crystallographic data for the structures **2**-**3**, **6**-**8**, **10**-**13**, **15**-**18**. These data can be obtained free of charge from the Cambridge Crystallographic Data Centre via https://www.ccdc.cam.ac.uk/structures/.

Table S1. Crystallographic details

	**compound_2**	**compound_3**	**compound_6**
**CCDC-Number**	2424011	2424012	2424013
Chemical formula	$C_{57}H_{87}N_3Si_2NaO_6P$	$C_{68}H_{103}N_5Si_2PCu$	$[C_{61}H_{98}N_4Si_2PAu]_2 \cdot C_4H_{10}O$
M_r	960.38	1141.24	2417.21
Crystal system, space group	Triclinic, $P\bar{1}$	Monoclinic, $P2_1/c$	Orthorhombic, $Pca2_1$
Temperature (K)	100	100	100
a (Å), α(°)<br>*b (Å)*, β(°)<br>*c (Å)*, γ(°)	12.8259(16), 83.051(4)<br>14.8361(19), 87.933(4)<br>20.480(3), 71.658(4)	13.2002(10), 90<br>14.6309(11), 91.517(3)<br>34.694(3), 90	16.752(2), 90<br>13.881(2), 90<br>54.704(8), 90
V (Å^3)	3671.9(8)	6698.1(9)	12721(3)
Z	2	4	4
F(000)	1044	2472	5080
D_x (g/cm^3)	0.869	1.132	1.262
Radiation type	Mo $K\alpha$	Mo $K\alpha$	Mo $K\alpha$
μ (mm^{-1})	0.112	0.427	2.416
θ range (°) for cell meas.	2.34–25.55	2.17–24.98	2.21–21.52
Crystal size (mm)	0.46 × 0.32 × 0.176	0.336 × 0.240 × 0.233	0.228 × 0.144 × 0.101
Diffractometer	Bruker Photon CMOS	Bruker Photon CMOS	Bruker Photon CMOS
Radiation source	TXS rotating anode	TXS rotating anode	TXS rotating anode
Monochromator	Helios optic	Helios optic	Helios optic
Absorption correction	Multi-scan	Multi-scan	Multi-scan
T_{min}, T_{max}	0.680, 0.745	0.681, 0.745	0.636, 0.745
θ_{max} (°)	25.866	25.710	25.76
Range of h, k, l	h = -15→15, k = -18→18, l = -24→24	h = -16→16, k = -17→17, l = -42→42	h = 0→20, k = 0→16, l = -66→66
Refinement method	Full-matrix least-squares on F^2	Full-matrix least-squares on F^2	Full-matrix least-squares on F^2
Data/restraints/ parameters	13951/115/633	12703/75/792	24287/31/1345
Goodness-of-fit on F^2	1.046	1.050	1.055
Final R indices (I>2σ(I))	R_1 = 0.0584, wR_2 = 0.1699	R_1 = 0.0477, wR_2 = 0.1136	R_1 = 0.0489, wR_2 = 0.1124
$\Delta\rho_{max}$, $\Delta\rho_{min}$ (e Å^{-3})	0.984, -0.262	0.496, -0.407	3.459, -2.048

	compound_7	compound_8	compound_10
**CCDC-Number**	2424014	2424015	2480971
Chemical formula	$C_{68}H_{103}N_5Si_2PAg\cdot C_3H_3$	$[C_{68}H_{103}N_5Si_2PAu]_2\cdot 2C_5H_{12}$	$[C_{73}H_{103}N_5Si_2PO_5AgFe]\cdot C_7H_8$
M_r	1224.62	2771.73	1473.60
Crystal system, space group	Triclinic, $P\bar{1}$	Triclinic, $P\bar{1}$	Triclinic, $P\bar{1}$
Temperature (K)	100	100	100
a (Å), α(°)<br>*b (Å)*, β(°)<br>*c (Å)*, γ(°)	13.333(2), 102.806(6)<br>14.462(2), 96.868(6)<br>22.038(4), 112.891(4)	13.3476(9), 73.310(3)<br>21.8120(16), 84.296(3)<br>26.829(2), 83.488(3)	12.5289(7), 82.503(2)<br>13.1785(6), 82.091(2)<br>26.0357(13), 73.158(2)
V (Å^3)	3715.9(10)	7415.3(9)	4056.6(4)
Z	2	2	2
F(000)	1314	2924	1564
D_x (g/cm^3)	1.094	1.241	1.206
Radiation type	Mo $K\alpha$	Mo $K\alpha$	Mo $K\alpha$
μ (mm^{-1})	0.364	2.081	0.519
θ range (°) for cell meas.	2.66–25.63	2.63–25.39	2.21–25.61
Crystal size (mm)	0.28 × 0.227 × 0.166	0.255 × 0.222 × 0.178	0.331 × 0.150 × 0.118
Diffractometer	Bruker Photon CMOS	Bruker Photon CMOS	Bruker Photon CMOS
Radiation source	TXS rotating anode	TXS rotating anode	TXS rotating anode
Monochromator	Helios optic	Helios optic	Helios optic
Absorption correction	Multi-scan	Multi-scan	Multi-scan
T_{min}, T_{max}	0.714, 0.745	0.660, 0.745	0.694, 0.745
θ_{max} (°)	25.689	25.749	25.698
Range of h, k, l	h = -16→16, k = -17→17, l = -26→26	h = -16→16, k = -26→26, l = -32→32	h = -15→15, k = -16→16, l = -31→31
Refinement method	Full-matrix least-squares on F^2	Full-matrix least-squares on F^2	Full-matrix least-squares on F^2
Data/restraints/ parameters	14066/37/747	28264/242/1699	15366/0/884
Goodness-of-fit on F^2	1.100	1.034	1.027
Final R indices (I>2σ(I))	R_1 = 0.0388, wR_2 = 0.1137	R_1 = 0.0332, wR_2 = 0.0826	R_1 = 0.0417, wR_2 = 0.1127
$\Delta\rho_{max}$, $\Delta\rho_{min}$ (e Å^{-3})	0.665, -0.482	0.496, -0.407	2.511, -0.698

	**compound_11**	**compound_12**	**compound_13**
**CCDC-Number**	2480972	2480973	2480974
Chemical formula	$[C_{72}H_{103}N_5Si_2PO_4AgFe]\cdot C_5H_{12}$	$C_{68}H_{106}N_6Si_2PAg$	$C_{75}H_{112}N_6Si_2PAg$
M_r	1425.60	1202.60	1292.72
Crystal system, space group	Monoclinic, $P2_1/c$	Monoclinic, $P2_1/c$	Monoclinic, $P2_1/c$
Temperature (K)	100	100	100
a (Å), α(°)<br>*b (Å)*, β(°)<br>*c (Å)*, γ(°)	23.8696(8), 90<br>14.4404(4), 97.5480(10)<br>22.2679(8), 90	20.6511(18), 90<br>17.6665(13), 108.065(3)<br>20.6247(18), 90	20.6271(10), 90<br>12.4775(5), 101.691(2)<br>29.7113(16), 90
V (Å^3)	7608.9(4)	7153.6(10)	7488.3(6)
Z	4	4	4
F(000)	2040	2594	2776
D_x (g/cm^3)	1.244	1.117	1.147
Radiation type	Mo $K\alpha$	Mo $K\alpha$	Mo $K\alpha$
μ (mm^{-1})	0.550	0.378	0.365
θ range (°) for cell meas.	2.32–25.53	2.44–25.50	2.49–25.72
Crystal size (mm)	0.245 × 0.128 × 0.103	0.296 × 0.237 × 0.156	0.373 × 0.296 × 0.215
Diffractometer	Bruker Photon CMOS	Bruker Photon CMOS	Bruker Photon CMOS
Radiation source	TXS rotating anode	TXS rotating anode	TXS rotating anode
Monochromator	Helios optic	Helios optic	Helios optic
Absorption correction	Multi-scan	Multi-scan	Multi-scan
T_{min}, T_{max}	0.685, 0.745	0.681, 0.745	0.642, 0.745
θ_{max} (°)	25.684	25.739	25.728
Range of *h*, *k*, *l*	h = -29→69, k = -17→17, l = -27→27	h = -25→25, k = -21→21, l = -25→25	h = -25→25, k = -15→15, l = -36→36
Refinement method	Full-matrix least-squares on F^2	Full-matrix least-squares on F^2	Full-matrix least-squares on F^2
Data/restraints/ parameters	14435/144/918	13611/2/740	14285/0/801
Goodness-of-fit on F^2	1.032	1.120	1.099
Final R indices (I>2σ(I))	R_1 = 0.0396, wR_2 = 0.0957	R_1 = 0.0546, wR_2 = 0.1667	R_1 = 0.0507, wR_2 = 0.1190
$\Delta\rho_{max}$, $\Delta\rho_{min}$ (e Å^{-3})	0.589, -0.490	3.242, -0.742	1.377, -0.751

	compound_15	compound_16	compound_17
**CCDC-Number**	2424016	2480975	2480976
Chemical formula	$C_{74}H_{116}N_5Si_2PBO_2Ag$	$C_{48}H_{72}N_3Si_2PO_2Fe$	$[C_{77}H_{103}N_5Si_2PO_9AgMn_2]\cdot C_4H_{10}O$
M_r	1313.54	866.08	1621.66
Crystal system, space group	Triclinic, $P\bar{1}$	Monoclinic, $P2_1/c$	Triclinic, $P\bar{1}$
Temperature (K)	100	100	100
a (Å), α(°)<br>*b (Å)*, β(°)<br>*c (Å)*, γ(°)	12.602(3), 74.537(8)<br>15.817(4), 76.765(8)<br>21.958(5), 69.725(8)	10.4461(4), 90<br>24.2659(8),103.5740(10)<br>20.7376(8), 90	13.1012(6), 105.944(2)<br>16.3575(8), 95.661(2)<br>22.6549(9), 101.565(2)
V (Å^3)	3911.6(16)	5109.8(3)	4512.2(4)
Z	2	4	2
F(000)	1412	1864	1708
D_x (g/cm^3)	1.115	1.126	1.194
Radiation type	Mo $K\alpha$	Mo $K\alpha$	Mo $K\alpha$
μ (mm^{-1})	0.352	0.410	0.588
θ range (°) for cell meas.	2.27–25.58	2.59–25.64	2.241–25.674
Crystal size (mm)	0.245 × 0.183 × 0.126	0.255 × 0.214 × 0.104	0.365 × 0.256 × 0.198
Diffractometer	Bruker Photon CMOS	Bruker Photon CMOS	Bruker Photon CMOS
Radiation source	TXS rotating anode	TXS rotating anode	IMS microsource
Monochromator	Helios optic	Helios optic	Helios optic
Absorption correction	Multi-scan	Multi-scan	Multi-scan
T_{min}, T_{max}	0.504, 0.745	0.699, 0.745	0.706, 0.745
θ_{max} (°)	25.792	25.683	25.711
Range of h, k, l	h = -15→15, k = -19→19, l = -26→26	h = -12→12, k = -29→29, l = -25→25	h = -15→15, k = -19→19, l = -27→27
Refinement method	Full-matrix least-squares on F^2	Full-matrix least-squares on F^2	Full-matrix least-squares on F^2
Data/restraints/ parameters	14210/4/806	9686/0/533	17117/0/948
Goodness-of-fit on F^2	1.011	1.061	1.130
Final R indices (I>2σ(I))	R_1 = 0.0523, wR_2 = 0.1497	R_1 = 0.0412, wR_2 = 0.1132	R_1 = 0.0454, wR_2 = 0.1040
$\Delta\rho_{max}$, $\Delta\rho_{min}$ (e Å^{-3})	1.744, -1.176	0.603, -0.415	0.970, -1.040

	**compound_18**
**CCDC-Number**	2480977
Chemical formula	$C_{45}H_{67}N_3Si_2PO_4Mn$
M_r	856.10
Crystal system, space group	Triclinic, $P\bar{1}$
Temperature (K)	100
a (Å), α(°)<br>*b (Å)*, β(°)<br>*c (Å)*, γ(°)	10.7312(6), 87.560(3)<br>12.2037(8), 82.045(2)<br>18.2799(12), 78.184(3)
V (Å^3)	2320.4(3)
Z	2
F(000)	916
D_x (g/cm^3)	1.225
Radiation type	Mo *K*α
μ (mm^{-1})	0.414
θ range (°) for cell meas.	2.32–25.67
Crystal size (mm)	0.145 × 0.112 × 0.065
Diffractometer	Bruker Photon CMOS
Radiation source	TXS rotating anode
Monochromator	Helios optic
Absorption correction	Multi-scan
T_{min}, T_{max}	0.606, 0.745
$θ_{max}$ (°)	25.694
Range of *h*, *k*, *l*	*h* = -13→13, *k* = -14→14 *l* = -22→22
Refinement method	Full-matrix least-squares on F^2
Data/restraints/ parameters	8701/0/524
Goodness-of-fit on F^2	1.080
Final *R* indices (I>2σ(I))	R_1 = 0.0588, wR_2 = 0.1680
$Δρ_{max}$, $Δρ_{min}$ (e Å^{-3})	0.982, -0.531

3. Computational Details

All the calculations were performed using the Gaussian 16 software package.[S15] Geometry optimization of the compounds was conducted at the TPSS-D3(BJ),[S16-17] density functional theory level, according to the best agreement with the metric data from X-ray structure analyses (Table S2). The def2-SVP[S18] basis set is used to describe all of atoms. In addition, frequency calculations are carried out at the same level of theory to confirm the stationary points are minima with no imaginary frequencies. The stability of the wavefunction was checked for all optimized structures at the TPSS-D3(BJ)/def2-SVP level. The single-point energy calculations were performed at the TPSS-D3(BJ)/def2-TZVPP level of theory, and the SMD solvent model[S19] was used to simulate the solvent effect of benzene. The relative Gibbs free energy (ΔG) was the outcome of the formula $\Delta G = \Delta E + \Delta G_{ZPE}$, where ΔG_{ZPE} is the Gibbs free energy correction acquired from the TPSS-D3(BJ)/def2-SVP method and the electronic energy ΔE was obtained from single-point energies calculations at the TPSS-D3(BJ)/def2-TZVPP level. IRC calculations were used to check further whether the TSs connect the corresponding reactants and products.[S20] The natural population analysis (NPA) analysis was obtained using the NBO 7.0 program[S21]. The fuzzy atom bond order (FBO), bond order density (BOD), NMR spectra, and the natural adaptive orbital (NAdO) analyses were carried out using the *Multiwfn* program.[S22] Viewing of optimized structures and rendering of molecular orbitals were performed using the program *CYLview*[S23] and *VMD*[S24], respectively. Furthermore, the B97-2[S25]/def2-TZVP method is used to calculate the ^{31}P NMR absolute shielding constants are converted to ^{31}P NMR chemical shifts, with that of H_3PO_4 calculated at the same level ($\sigma(H_3PO_4)$ = 285.2 ppm) as reference.

Table S2. Key distances (Å) of experimental and DFT-optimized structures of compound **8**.

Functional	Exp.	TPSS	PBE0	CAM-B3LYP
C1-N1	1.281	1.287	1.276	1.273
N1-Si1	1.672	1.709	1.695	1.689
Si1-P1	2.111	2.118	2.108	2.099
P1-Si2	2.227	2.246	2.241	2.239
Si2-Au1	2.339	2.356	2.358	2.361
Au1-C2	2.106	2.106	2.096	2.113
∠C1-N1-Si1	142.8	142.8	145.1	147.6
∠N1-Si1-P1	107.4	110.5	110.1	109.9
∠N1-Si1-Au1	111.9	110.8	111.3	111.9
∠P1-Si1-Au1	140.4	137.5	137.5	137.2
∠Si2-Au1-C2	173.8	169.2	169.3	170.4
∠P1-Si1-C1-N1	138.4	138.7	136.4	136.8
RD(%)[a]	0.00	1.12	1.22	1.26

[a] RD = $\frac{\sum_{i=1}^{n}\frac{|\mathrm{BL(DFT)}-\mathrm{BL(Exp)}|}{\mathrm{BL(Exp)}}*100\%}{n}$, BL means bond length.

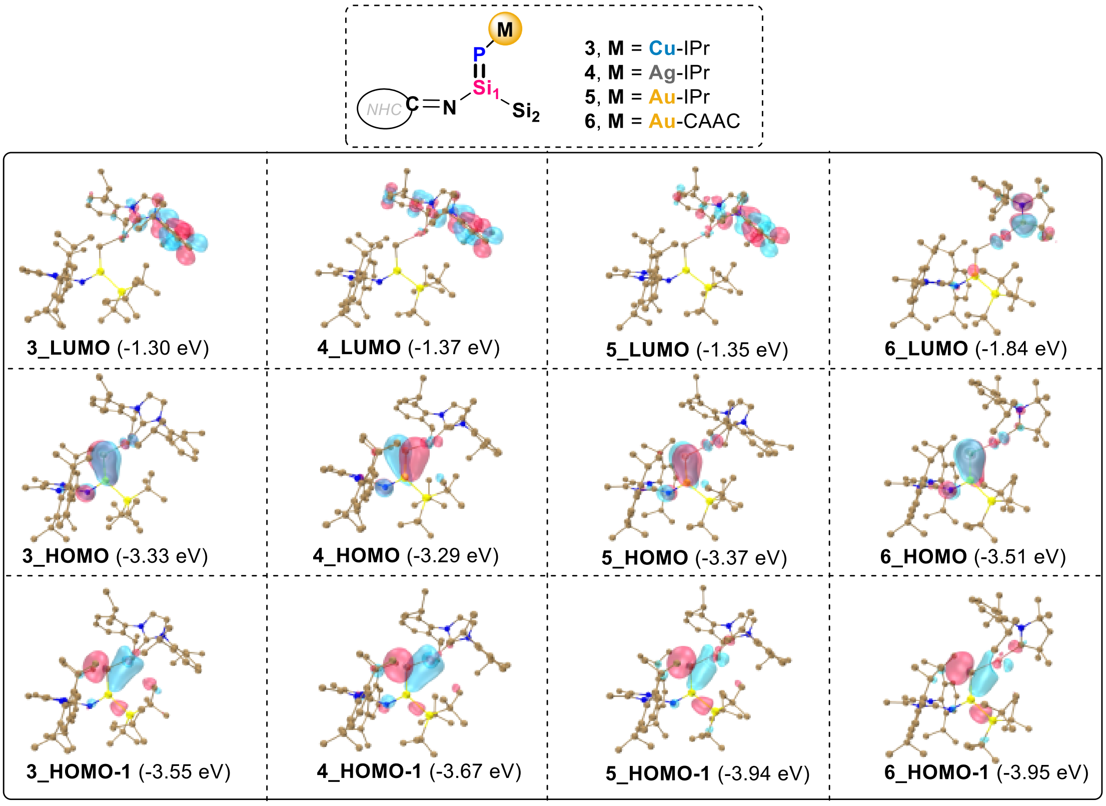

Figure S19. LUMO, HOMO and HOMO-1 of the compounds **3-6**. Hydrogen atoms in 3D structures are omitted for clarity. The isosurface 0.040 a.u. is plotted.

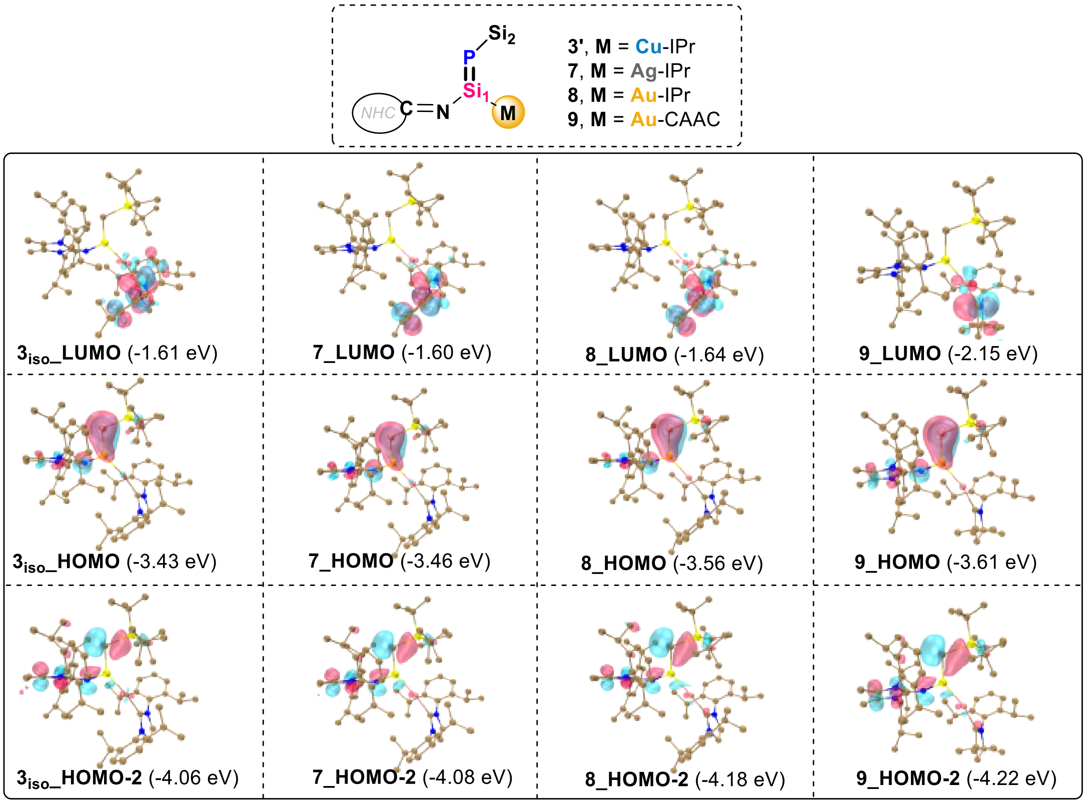

Figure S20. LUMO, HOMO and HOMO-2 of the compounds $\mathbf{3_{iso}}$ and **7-9**. Hydrogen atoms in 3D structures are omitted for clarity. The isosurface 0.040 a.u. is plotted.

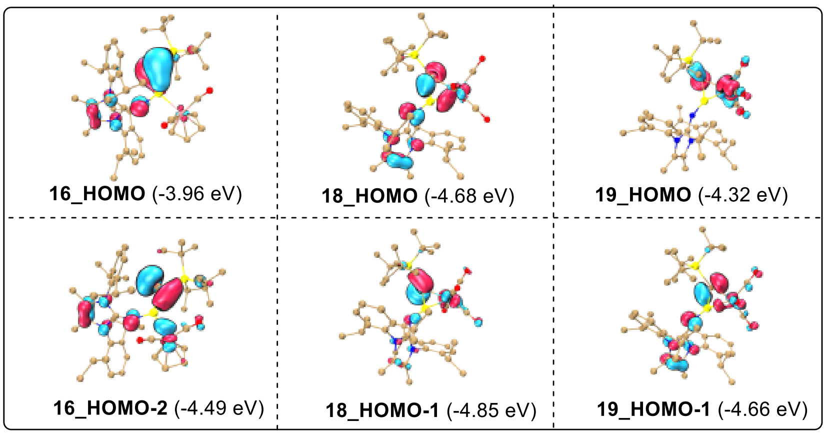

Figure S21. HOMO and HOMO-1 of the compounds **16** and **18-19**. Hydrogen atoms in 3D structures are omitted for clarity. The isosurface 0.040 a.u. is plotted.

Table S3. Mayer bond order (MBO) for complexes **3-9** and $\mathbf{3_{iso}}$

Complexes	C=N	N-Si$_1$	Si$_1$-Si$_2$/M	Si$_1$=P	P-M/Si$_2$
**3**	1.556	1.163	0.881	1.663	0.938
**4**	1.536	1.191	0.894	1.643	0.834
**5**	1.527	1.205	0.883	1.585	0.949
**6**	1.526	1.212	0.876	1.556	0.938
**3$_{iso}$**	1.611	1.144	0.915	1.447	1.085
**7**	1.610	1.138	0.842	1.495	1.083
**8**	1.590	1.158	0.890	1.511	1.074
**9**	1.620	1.165	0.811	1.530	1.068
**16**			0.882	1.498	1.041
**17**	1.508	1.317	1.118	-	1.063
**18**			0.958	1.120	0.682/0.943
**19**			1.120	1.121	0.648/0.937

Table S4. NPA charges for complexes **3**-**9** and **3$_{iso}$**

Complexes	**C**	**N**	**Si$_1$**	**Si$_2$**	**P**	**M** (Cu, Ag, Au, Fe, Mn, Co)
**3**	+0.59	-1.08	+0.86	+1.45	-1.06	+0.60
**4**	+0.59	-1.08	+0.88	+1.43	-1.04	+0.51
**5**	+0.59	-1.08	+0.89	+1.44	-0.96	+0.33
**6**	+0.59	-1.08	+0.89	+1.44	-0.91	+0.36
**3$_{iso}$**	+0.57	-1.05	+0.62	+1.72	-0.88	+0.50
**7**	+0.57	-1.05	+0.66	+1.71	-0.88	+0.40
**8**	+0.58	-1.05	+0.79	+1.71	-0.87	+0.21
**9**	+0.57	-1.05	+0.76	+1.71	-0.84	+0.24
**16**			+1.174		-0.839	-0.44
**17**			+1.382		-1.059	-1.217/-1.045
**18**			+1.281		-0.679	-1.142
**19**			+1.192		-0.712	-0.192

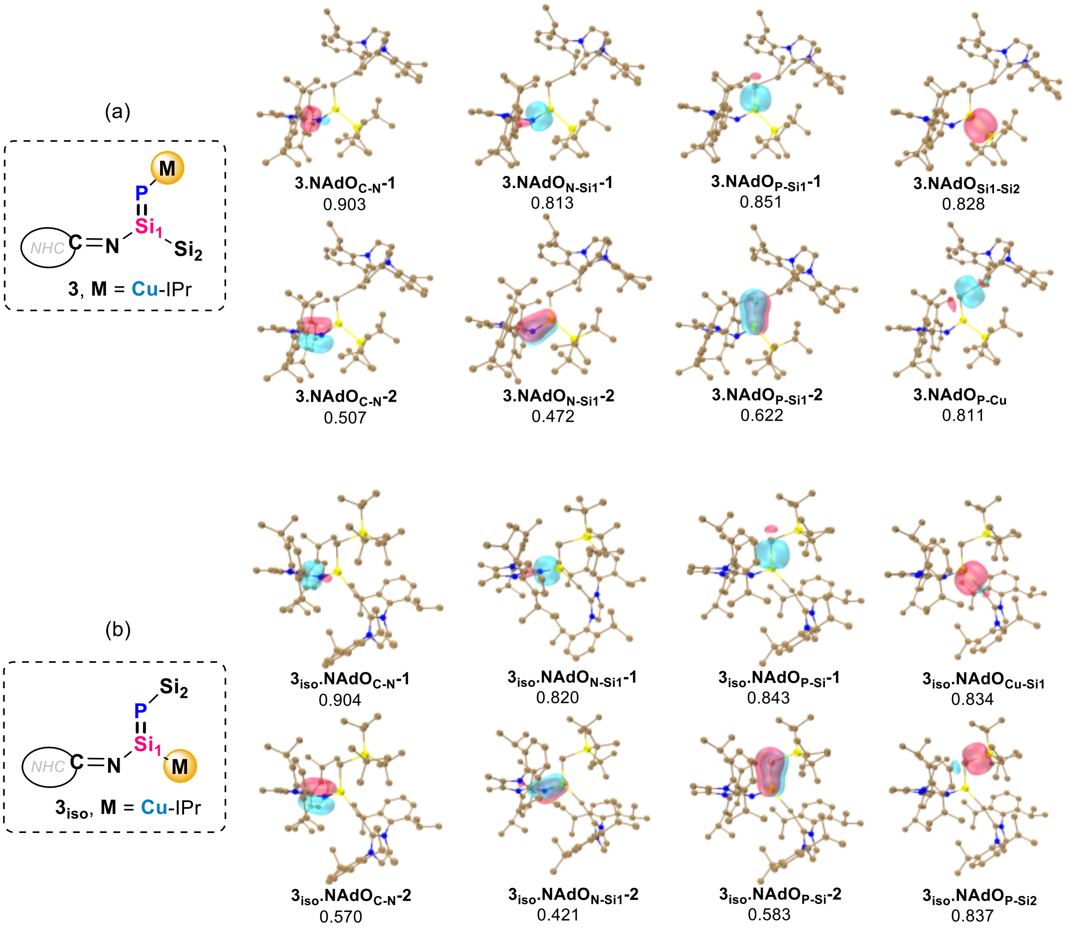

Figure S22. Natural adaptive orbital (NAdO) analysis on the bonding modes of **3** (a), and $\mathbf{3}_{\mathbf{iso}}$ (b). Hydrogen atoms in 3D structures are omitted for clarity. The isosurface 0.050 a.u. is plotted.

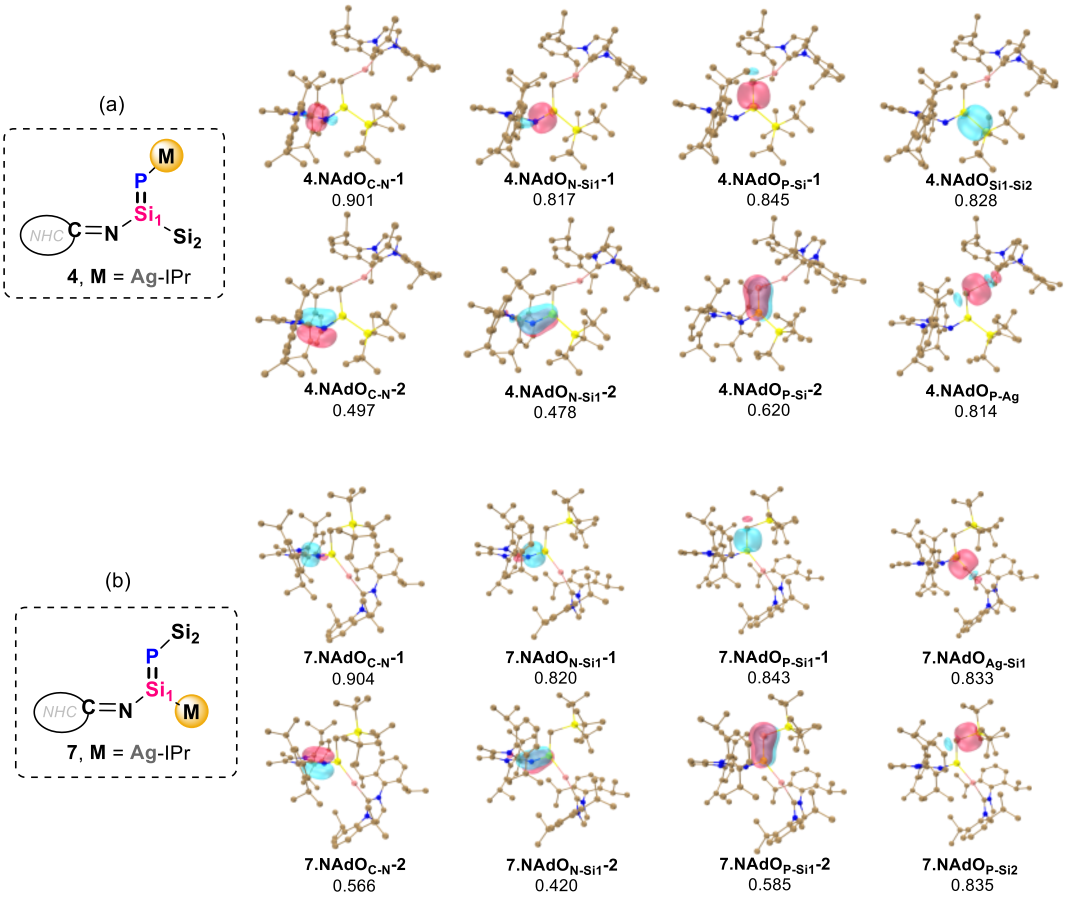

Figure S23. NAdO analysis on the bonding modes of **4** (a), and **7** (b). Hydrogen atoms in 3D structures are omitted for clarity. The isosurface 0.050 a.u. is plotted.

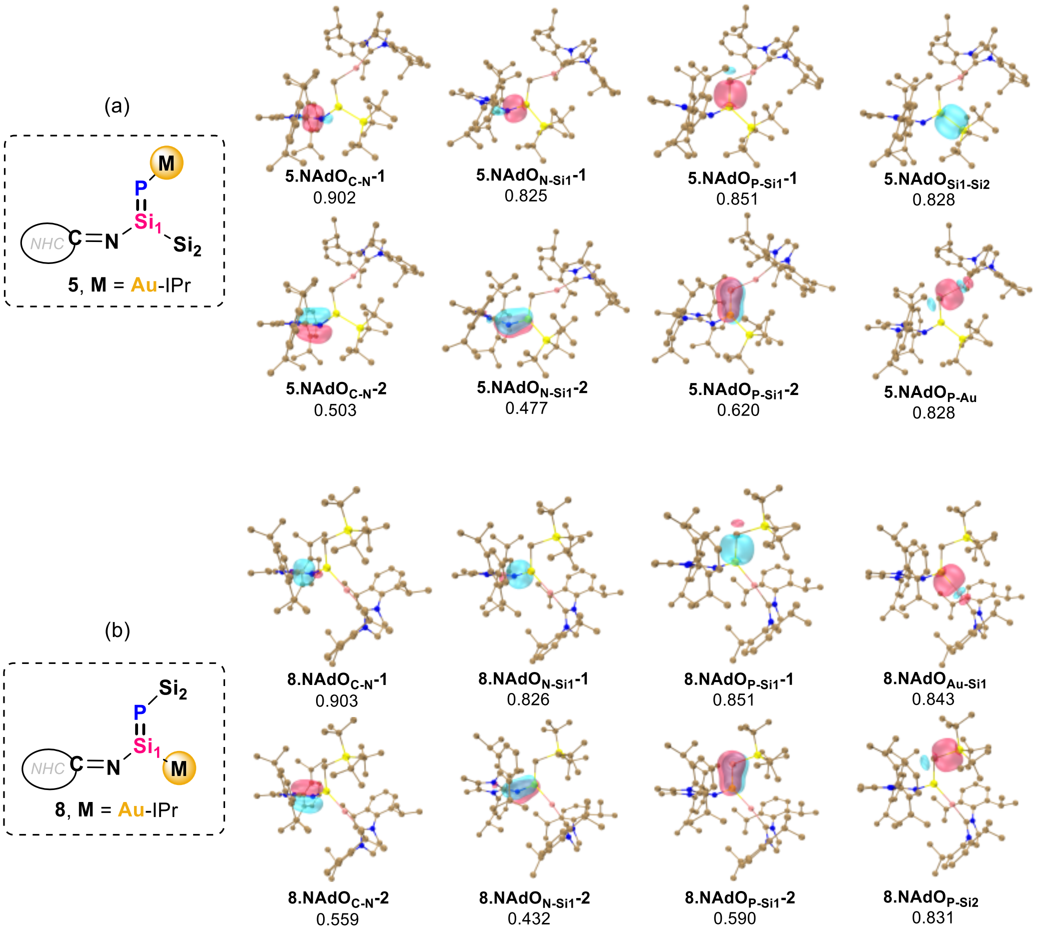

Figure S24. NAdO analysis on the bonding modes of **5** (a), and **8** (b). Hydrogen atoms in 3D structures are omitted for clarity. The isosurface 0.050 a.u. is plotted.

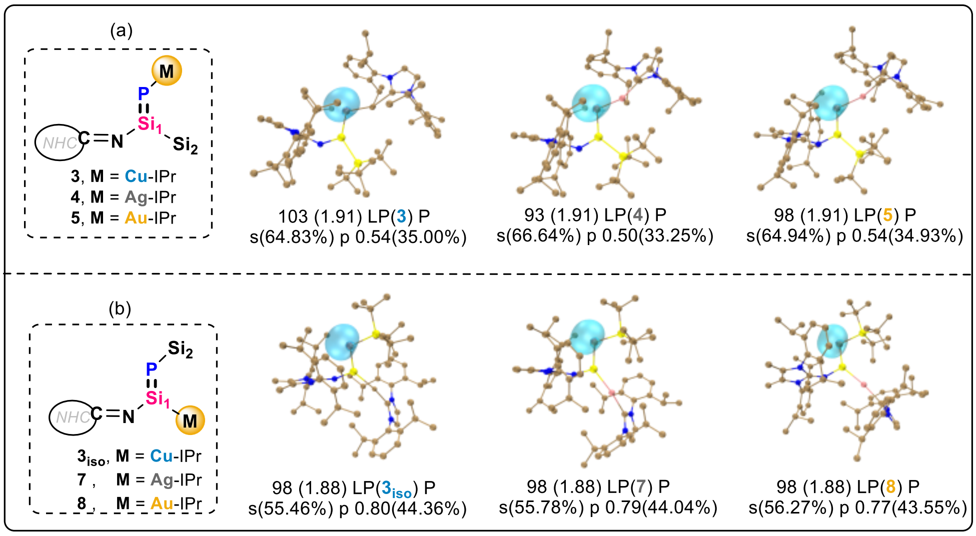

Figure S25. Natural bond orbital (NBO) analysis of complexes **3-5**, $\mathbf{3_{iso}}$, **7**, and **8**. Hydrogen atoms in 3D structures are omitted for clarity. The isosurface 0.050 a.u. is plotted.

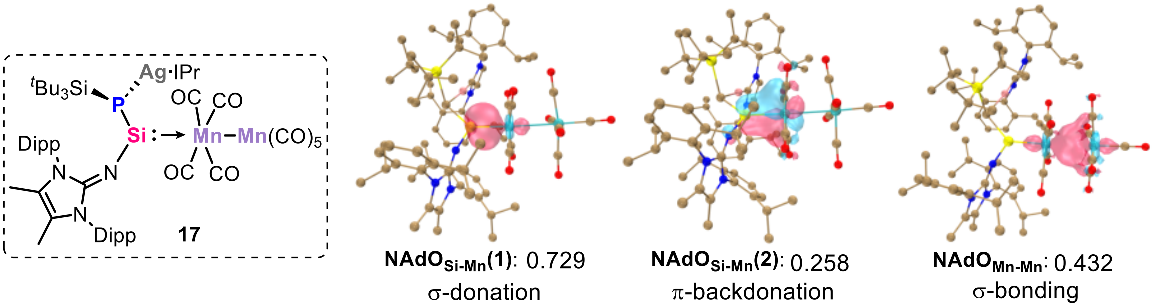

Figure S26. NAdO analysis on the bonding modes of **17**. Hydrogen atoms in 3D structures are omitted for clarity. The isosurface 0.050 a.u. is plotted.

Table S5. NBO analysis of complexes **3-5**, **7**, **8**, and **3$_{iso}$**.

===

3

(Occupancy) Bond orbital / Coefficients / Hybrids

103. (1.90554) LP (1) P   s (64.83%) p 0.54(35.00%)

0.0000 0.0000 0.8052 -0.0019 -0.0011
0.0000 -0.0787 -0.0004 -0.0004 0.0001
0.0000 0.5124 0.0033 0.0010 -0.0002
0.0000 0.2850 0.0020 0.0001 -0.0001
0.0038 -0.0031 0.0024 -0.0016 -0.0230
0.0012 0.0321 -0.0049 -0.0012 0.0020
-0.0006 -0.0009 0.0005 -0.0024 0.0002
0.0008 -0.0027

4

93. (1.90958) LP (1) P   s (66.64%) p 0.50(33.25%)

0.0000 0.0000 0.8163 -0.0026 -0.0007
0.0000 -0.0664 -0.0032 -0.0001 -0.0001
0.0000 0.4111 -0.0005 0.0008 0.0001
0.0000 0.3988 -0.0005 0.0009 0.0002
0.0038 -0.0030 0.0010 -0.0014 -0.0230
0.0010 0.0201 -0.0042 -0.0128 0.0028
0.0008 -0.0014 0.0024 -0.0031 -0.0001
0.0005 -0.0004

5

98. (1.91052) LP (1) P   s (64.94%) p 0.54(34.93%)

0.0000 0.0000 0.8059 -0.0037 -0.0005
0.0000 0.1092 -0.0003 0.0000 0.0002
0.0000 0.4529 -0.0047 0.0016 0.0002
0.0000 -0.3637 -0.0008 -0.0005 -0.0001
-0.0043 0.0031 0.0051 -0.0024 0.0244
-0.0028 0.0221 -0.0058 -0.0080 0.0035
0.0007 0.0009 0.0013 0.0025 -0.0009
-0.0009 -0.0004

3_{iso}

98. (1.88135) LP (1) P s(55.46%) p 0.80(44.36%)

0.0000 0.0000 0.7447 -0.0061 -0.0002
0.0000 0.6604 -0.0215 0.0029 -0.0003
0.0000 0.0425 0.0029 0.0014 0.0000
0.0000 0.0722 -0.0005 0.0000 -0.0001
-0.0020 0.0006 -0.0051 0.0003 -0.0007
0.0002 -0.0351 0.0044 0.0222 0.0003
-0.0014 -0.0059 0.0007 0.0004 -0.0003
0.0029 0.0013

7

98. (1.87904) LP (1) P s(55.78%) p 0.79(44.04%)

0.0000 0.0000 0.7469 -0.0056 -0.0003
0.0000 0.6570 -0.0197 0.0033 -0.0003
0.0000 -0.0277 0.0042 0.0012 0.0000
0.0000 0.0869 -0.0003 0.0001 -0.0001
0.0055 -0.0007 -0.0069 0.0004 -0.0002
0.0002 -0.0349 0.0045 0.0212 0.0004
-0.0016 -0.0057 0.0013 0.0000 -0.0004
0.0031 0.0006

8

98. (1.87536) LP (1) P s(56.27%) p 0.77(43.55%)

0.0000 0.0000 0.7501 -0.0052 -0.0003
0.0000 0.6528 -0.0200 0.0032 -0.0003
0.0000 0.0429 0.0016 0.0015 0.0000
0.0000 0.0838 -0.0025 0.0003 -0.0001

-0.0033 0.0002 -0.0077 0.0003 -0.0003
-0.0004 -0.0357 0.0045 0.0197 0.0006
-0.0016 -0.0058 0.0008 0.0013 0.0003
0.0026 0.0017

==

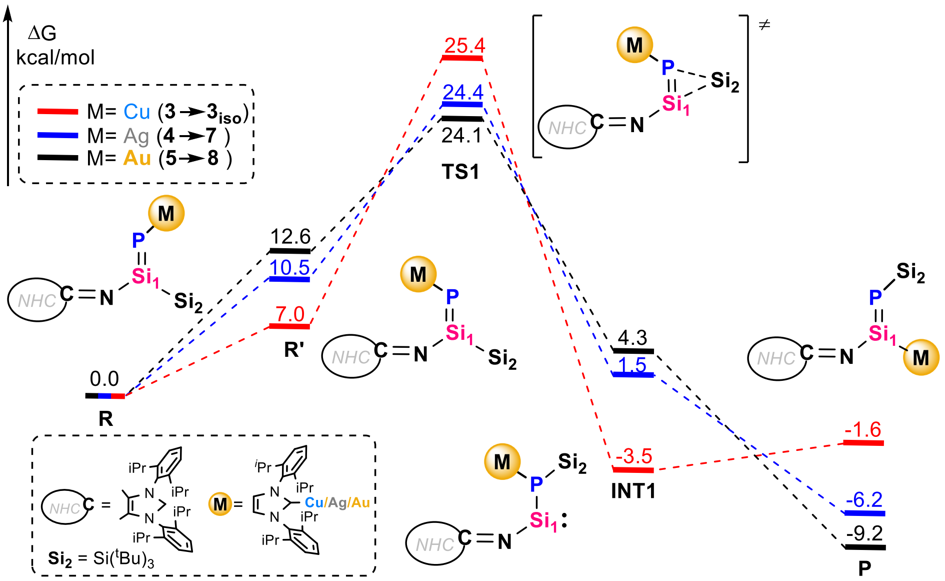

Figure S27. Gibbs energy profiles for the isomerization reactions between **3**, **4**, **5** and **3**$_{\mathbf{iso}}$, **7**, **8** in the TPSS-D3(BJ)/def2-TZVPP//TPSS-D3(BJ)/def2-SVP level. The Gibbs energies are given in kcal/mol.

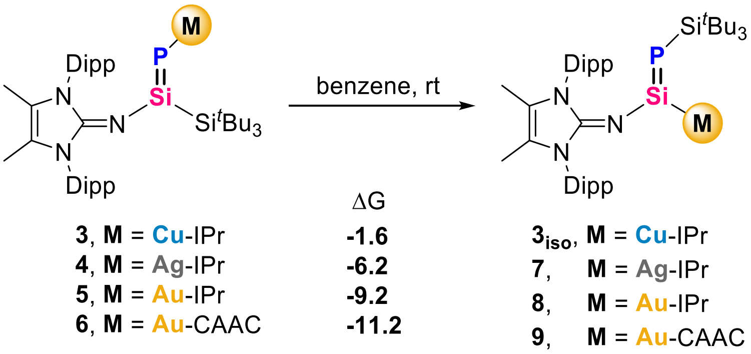

Figure S28. Gibbs energies (ΔG, kcal/mol) of these reactions at the TPSS(D3BJ)/def2-TZVPP // TPSS(D3BJ)/def2-SVP level.

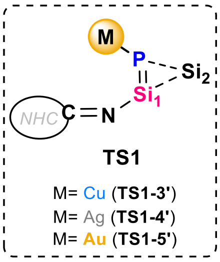

d(P-Si_2)	MBO(P-Si_2)	*d*(Si_1-Si_2)	MBO(Si_1-Si_2)	∠P-Si1-Si2
2.780	0.474	2.437	0.705	74.0
2.788	0.469	2.436	0.709	74.4
**2.796**	**0.454**	**2.445**	**0.696**	**74.3**

Figure S29. Optimized geometries of transition states (**TS1-3'**, **TS1-4'**, and **TS1-5'**). Distances are in Å, and the MBOs of key bonds are calculated.

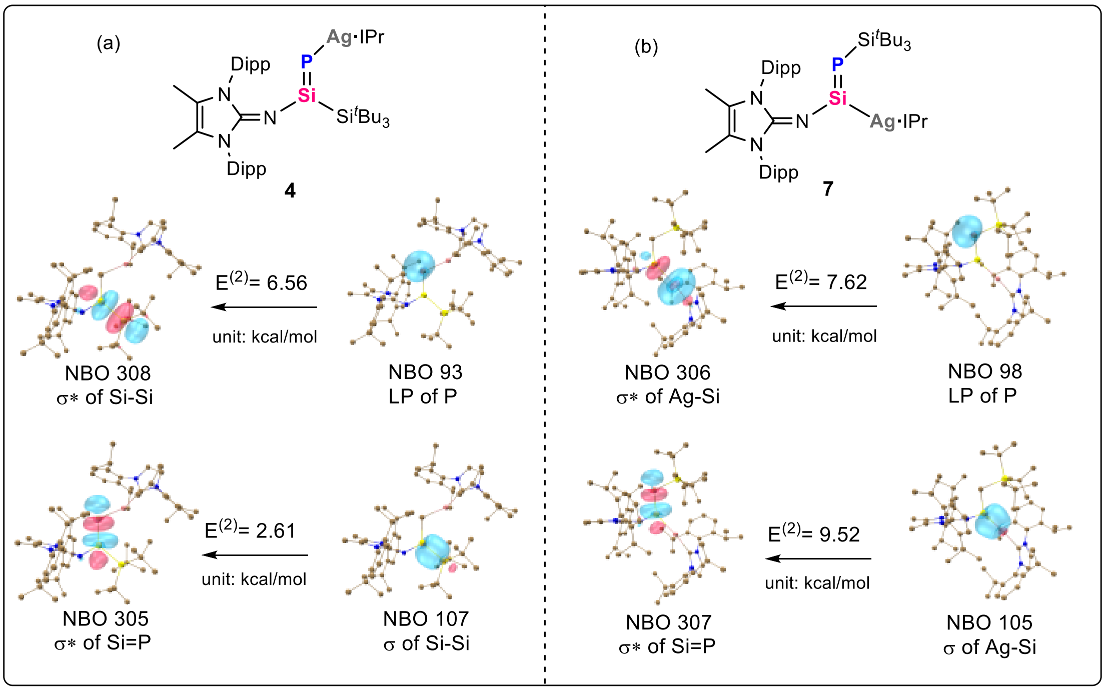

Figure S30. Selected NBOs of metalated phosphasilenes **4** (a) and **7** (b) for the second-order perturbation theory analysis. (isovalue = 0.05)

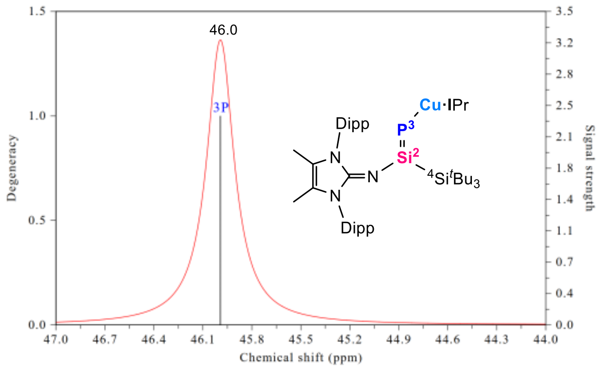

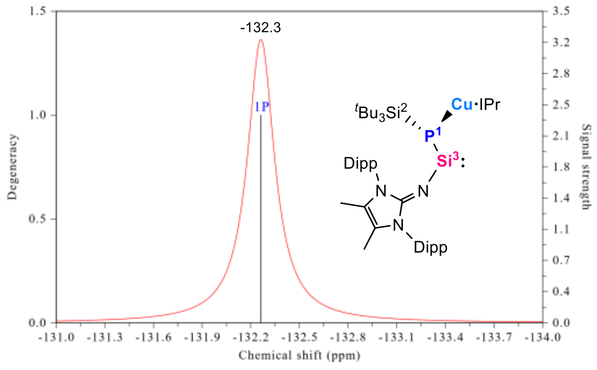

Figure S31. Calculated ^{31}P NMR spectrum of compounds **3** and **INT1-3**. According to the DFT calculation, the ^{31}P NM peaks located at +46.0, and -132.3 ppm are assigned to the P^3 and P^1 atoms, respectively.

Table S6. Calculated ^{31}P NMR spectrum of compounds **H_3PO_4**, **3**, and **INT1-3** using different methods. **H_3PO_4** calculated at the same level as reference.

Compounds	Computational method	Atomic number	Calculated GIAO magnetic shielding tensor (ppm)	^{31}P NMR chemical shift (ppm)
**H_3PO_4**	B97-2/def2-TZVP (SMD, toluene)	P^1	285.2	reference point
	B97-2/cc-pVTZ/SDD (SMD, toluene)	P^1	321.5	reference point
	TPSSh/def2-TZVP (SMD, toluene)	P^1	286.8	reference point
**3**	B97-2/def2-TZVP (SMD, toluene)	P^3	239.2	46.0
	B97-2/cc-pVTZ/SDD (SMD, toluene)	P^3	267.3	54.2
	TPSSh/def2-TZVP (SMD, toluene)	P^3	238.3	48.4
**INT1-3**	B97-2/def2-TZVP (SMD, toluene)	P^1	417.5	-132.3
	B97-2/cc-pVTZ/SDD (SMD, toluene)	P^1	451.6	-130.1
	TPSSh/def2-TZVP (SMD, toluene)	P^1	416.0	-129.2

4. Appendix: Cartesian coordinates of the optimized geometries

2

TPSS-D3(BJ)/def2-SVP

E = -3730.869216 a.u.

Si	0.35856300	0.93593100	-0.38148100
P	-1.46094700	0.34251700	-1.55192600
Si	0.97149200	3.21911200	-0.42330000
Na	-4.23350100	-0.11245400	0.51767800
O	-4.98809100	-2.13928900	-0.30403100
N	1.71516700	-0.08583300	-0.04569200
O	-4.94146400	1.04379800	2.56895600
O	-2.16012600	0.49768100	1.21626000
O	-5.42978200	0.09907200	-1.69415700
O	-4.77509300	2.10932800	0.02466400
O	-4.61900900	-1.72701900	2.28972600
C	-1.37939800	0.57624700	0.22267100
N	1.69837900	-2.46068100	-0.71100400
N	3.34238900	-1.72016700	0.55477900
C	3.75912900	-0.50873700	2.64624100
C	5.42054600	-0.46157500	0.79881700
C	-5.37728300	-2.27541800	-1.66776200
H	-6.48014300	-2.37924600	-1.73150300
H	-4.90805900	-3.16597800	-2.12981800
C	2.16123800	-1.29115800	-0.07866300
C	0.46509300	-2.63702300	-1.41864500
C	-0.58608700	-3.32443800	-0.76852200
C	-0.47977700	-3.69939200	0.70679100
H	0.59139900	-3.83382300	0.93768300
C	-1.18932300	-5.01859100	1.05365200
H	-2.28711000	-4.93154300	0.95621400
H	-0.97801900	-5.29696900	2.10103200
H	-0.85812200	-5.84647500	0.40186600
C	-4.22374800	0.27620400	3.53783100
H	-4.31711100	0.73092100	4.54678100
H	-3.15392300	0.22389900	3.25566100
C	1.60185500	3.62916400	-2.23953500
C	0.39323300	3.69947100	-3.20083600
H	-0.24321300	2.80001400	-3.11460600
H	0.75567700	3.76347800	-4.24733500
H	-0.23468300	4.58748600	-3.01619100
C	2.40680900	4.94223100	-2.34543200
H	1.82321800	5.81921700	-2.01875500
H	2.70786400	5.11666700	-3.39909600
H	3.33068200	4.90721900	-1.74269900
C	2.48973900	2.46608500	-2.73461800
H	3.42053100	2.37286500	-2.15857700
H	2.76392800	2.62872100	-3.79723200
H	1.95531400	1.50252700	-2.66950200
C	-0.60711800	4.31614700	0.04046600
C	-1.89294800	3.91048000	-0.71714700
H	-1.78537000	3.96168700	-1.81079500
H	-2.70332800	4.61477900	-0.42992100
H	-2.21817700	2.89180400	-0.46285800
C	-0.92757800	4.14114400	1.54323500
H	-1.06226500	3.07559900	1.80128800
H	-1.87235900	4.67318400	1.78217400
H	-0.14431800	4.56368900	2.19408600
C	-0.35359600	5.81469900	-0.24674100
H	0.55411800	6.20016100	0.24480700
H	-1.21269600	6.41289200	0.12176500
H	-0.26278300	6.01094000	-1.32906500
C	2.38709800	3.46618900	0.91964200
C	2.02950900	2.65585600	2.18752900
H	1.10746200	3.00093400	2.67967000
H	2.85748200	2.74012000	2.92013700
H	1.91269200	1.58813600	1.93717000
C	3.70500200	2.85608800	0.39379200
H	3.55690400	1.80292700	0.10533600
H	4.46785000	2.87609300	1.19706700
H	4.11538400	3.41215700	-0.46647800
C	2.64882900	4.93411300	1.31806700
H	2.90688200	5.56587300	0.45050800
H	3.50149800	4.97853600	2.02647000
H	1.78103500	5.39003400	1.82499900
C	3.59537400	-3.08439200	0.30972500
C	4.76404600	-3.79569700	0.91024200
H	5.72978500	-3.38898700	0.55988000
H	4.72737700	-4.86539900	0.65173000
H	4.75953200	-3.70493900	2.01163400
C	2.57714500	-3.54495300	-0.47670100
C	2.33028100	-4.89738300	-1.06036800
H	1.41165100	-5.36377800	-0.66036900
H	3.17896800	-5.56310300	-0.83849000
H	2.20872900	-4.84245600	-2.15720500
C	0.37171000	-2.19444000	-2.75792100
C	1.49828600	-1.37262200	-3.37611500
H	1.85612600	-0.69937000	-2.57599500

C	2.68829000	-2.25212500	-3.81223700
H	3.11859900	-2.80199100	-2.95905700
H	3.48706000	-1.62285500	-4.24368100
H	2.37584900	-2.98447900	-4.57886200
C	1.01789400	-0.48249500	-4.53182500
H	0.72039000	-1.07910100	-5.41392500
H	1.83151700	0.19151600	-4.84847700
H	0.15953200	0.13603000	-4.21778400
C	-0.79823000	-2.50847300	-3.46798300
H	-0.90614300	-2.17993600	-4.50485100
C	-1.83841600	-3.21809100	-2.85822700
H	-2.73802300	-3.46165300	-3.43506500
C	-1.74266100	-3.60315100	-1.51750100
H	-2.57392000	-4.13590500	-1.04642200
C	-0.98685700	-2.54375700	1.59245900
H	-0.44872300	-1.60820300	1.37851700
H	-0.84818400	-2.79225300	2.66060800
H	-2.05789300	-2.34691100	1.42199700
C	4.18120700	-0.87337300	1.34452000
C	2.40936700	-0.98083900	3.18782500
H	1.67416200	-0.79722800	2.38329200
C	2.40927700	-2.49461200	3.49503800
H	2.59060700	-3.09859800	2.59229300
H	1.42895600	-2.79473100	3.90561200
H	3.18341000	-2.74109800	4.24436400
C	1.93548200	-0.20647600	4.42744000
H	2.56588400	-0.42309800	5.30973800
H	0.90322800	-0.50898300	4.67540100
H	1.93737900	0.88181700	4.25813300
C	4.62979300	0.29098400	3.40690600
H	4.33805600	0.60118200	4.41383500
C	5.86743300	0.69907500	2.89553000
H	6.53000000	1.32044000	3.50780900
C	6.25928300	0.32787600	1.60487700
H	7.22399700	0.66672600	1.21612900
C	5.79017600	-0.81996500	-0.63973200
H	5.36830300	-1.81749000	-0.85216000
C	7.30541300	-0.90082300	-0.88151300
H	7.78592700	0.09085800	-0.80186300
H	7.50423900	-1.27859200	-1.89971100
H	7.79759600	-1.57697000	-0.16000600
C	5.11843000	0.15227900	-1.63006500
H	4.02874500	0.18392300	-1.47252900
H	5.31358100	-0.16193500	-2.67113100
H	5.51061600	1.17551600	-1.49737300
C	-5.00429900	1.36479200	-2.20927000
H	-5.48440500	1.57047100	-3.18891600
H	-3.90132100	1.36689100	-2.33550000
C	-5.41575200	2.42492000	-1.20271100
H	-5.10055600	3.42379600	-1.56760800
H	-6.51822200	2.42821000	-1.06704700
C	-5.09700700	2.93879600	1.13301700
H	-6.19726800	2.96494800	1.28224000
H	-4.73686800	3.97419800	0.96652000
C	-4.40687900	2.35429600	2.35246600
H	-3.31907600	2.28303100	2.16829900
H	-4.59551800	3.00240600	3.23372900
C	-4.90527400	-1.02748700	-2.39647400
H	-3.79538600	-0.98283900	-2.40932100
H	-5.27387500	-1.05303700	-3.44383400
C	-5.18726500	-3.25596900	0.55281900
H	-4.25909400	-3.85822800	0.59869700
H	-6.00762600	-3.90214200	0.18424800
C	-5.55716900	-2.73120500	1.93550100
H	-6.58048400	-2.29922100	1.91825800
H	-5.54682800	-3.57062400	2.66262000
C	-4.84816200	-1.10782600	3.55727700
H	-4.40399400	-1.71775500	4.36978500
H	-5.93855800	-1.01766700	3.73799000

3

TPSS-D3(BJ)/def2-SVP

E = -5486.473187 a.u.

Cu	2.43778200	0.35825400	0.12783700
Si	-0.98004000	-0.55583900	-0.33057800
P	0.32761600	0.87568700	0.46448000
Si	-0.76478700	-2.64846400	-1.43107100
N	-4.05508500	0.58400300	1.58891600
N	-3.89185800	1.78475900	-0.25184700
N	5.40957800	-0.25525100	0.50212000
N	-2.67093900	-0.31210000	-0.17200500
N	4.86804500	1.82982600	0.53663600
C	-3.43518400	0.60370300	0.33753100
C	5.71754200	-3.67410800	-0.93579100
H	6.07204400	-4.16775200	-1.84514000
C	-4.04856100	-0.55259700	2.46787100
C	-2.95273000	-0.74979200	3.34176300
C	4.77582000	-2.43484900	1.43047600
C	-4.87162800	1.72116100	1.76201900

C	5.33677100	-1.68869600	0.37341800	H	2.47453400	-4.21183600	-3.00836700
C	5.14663600	-4.44093300	0.08845100	H	0.78171900	-4.48993300	-3.49993600
H	5.06131000	-5.52633600	-0.02884900	C	-4.66415400	2.07575900	-3.88136900
C	-1.76927300	0.20613100	3.34572100	H	-5.38035300	1.68795700	-4.61120500
H	-1.59367900	0.52266000	2.30153500	C	-2.05226800	1.48345300	4.16036300
C	-4.76809900	2.46968400	0.62196100	H	-2.91080100	2.04013400	3.75094500
C	5.82195300	-2.27842000	-0.81796400	H	-1.17318900	2.15075700	4.11974700
C	-5.14235400	-2.54353000	3.29554200	H	-2.26247200	1.24621800	5.21966100
H	-5.97789100	-3.25126100	3.27517700	C	6.34960700	-1.41144000	-1.95768200
C	-3.75298000	2.13493500	-1.64180100	H	6.81081300	-0.51480500	-1.50716300
C	6.57226300	0.45253900	0.80695700	C	-2.76685600	3.49345900	-3.37611700
H	7.52050400	-0.04983800	0.98611800	H	-2.00555300	4.20598000	-3.70880700
C	4.68249200	-3.82866200	1.25538100	C	-0.46506400	-0.45544800	3.80807700
H	4.23743700	-4.43921100	2.04783100	H	-0.46318100	-0.66807400	4.89396000
C	6.22892300	1.77551000	0.82376700	H	0.37676500	0.21807100	3.58342200
H	6.81541700	2.67212400	1.01207500	H	-0.28474300	-1.39675000	3.26287400
C	-2.99420100	-1.86847900	4.19315700	C	3.01716200	4.74963500	-0.84918400
H	-2.15979000	-2.05279100	4.87537600	H	2.77441100	5.19876900	-1.81734100
C	-5.14909000	-1.44092600	2.42226400	C	2.80396000	4.75260700	1.56932700
C	4.07478100	3.03359500	0.46451000	H	2.39656400	5.20587400	2.47806300
C	-6.29068400	-1.26340600	1.42650700	C	-3.72377300	3.03212600	-4.28184000
H	-6.18588100	-0.26581200	0.96874100	H	-3.72243200	3.39702700	-5.31480900
C	-4.69673600	1.60142400	-2.55923900	C	-6.98333700	1.10487900	-1.51692100
C	-4.07724100	-2.75441400	4.17517800	H	-6.77679300	1.64226000	-0.57889000
H	-4.08491900	-3.62098800	4.84530800	H	-7.69260700	0.28997200	-1.28628900
C	4.33162100	0.58057700	0.33386600	H	-7.47871800	1.79929700	-2.21917300
C	-5.69785900	0.52327500	-2.14380300	C	-7.67699600	-1.32346200	2.09480300
H	-5.19500800	-0.09299800	-1.37867300	H	-7.88524600	-2.32616200	2.50883700
C	-2.74796400	3.04542500	-2.04068900	H	-8.46558800	-1.09896800	1.35474200
C	-5.39662500	3.77344900	0.25430200	H	-7.76259900	-0.59503100	2.91986500
H	-4.65546100	4.59105200	0.21334300	C	2.52309400	5.32737200	0.32471900
H	-6.16534900	4.04375100	0.99485500	H	1.90189400	6.22751500	0.26850200
H	-5.87370300	3.72309700	-0.73992700	C	-1.63472200	3.49635300	-1.10379800
C	-5.66573100	1.94080700	3.00691100	H	-1.59996100	2.78870400	-0.25663000
H	-6.41418200	1.14249500	3.15594900	C	-6.17820200	-2.30322500	0.29459500
H	-6.19565100	2.90425300	2.95236800	H	-5.20594800	-2.22029200	-0.21386200
H	-5.02227700	1.94906800	3.90434900	H	-6.97845300	-2.15401700	-0.45191900
C	3.59160900	3.59305800	1.66885900	H	-6.26728700	-3.32993200	0.69255700
C	3.80302000	3.58488400	-0.80672700	C	-6.07311500	-0.40847600	-3.30936200
C	4.28491900	-1.78173000	2.71699500	H	-6.73473400	0.09686700	-4.03609900
H	4.55355300	-0.71256000	2.67509600	H	-6.61768100	-1.28750000	-2.92414400
C	4.27614900	2.91553100	-2.09176900	H	-5.17982400	-0.76575700	-3.84506300
H	4.99504000	2.12659600	-1.81249500	C	-1.47778200	-5.40846200	-0.77270100
C	1.42490800	-4.21285500	-2.64944600	H	-2.33628200	-5.39303300	-1.46580600
H	1.34439400	-5.00804100	-1.88773800	H	-1.69963000	-6.16439500	0.00803800

H	-0.59621600	-5.76101600	-1.33511000
C	3.83801500	2.91497300	3.01249400
H	4.72442300	2.26591100	2.90130400
C	-1.68827200	-3.59810800	-4.05148000
H	-0.72455300	-3.38153300	-4.54272100
H	-2.47148300	-3.56172200	-4.83595200
H	-1.65157700	-4.63291000	-3.66805800
C	2.75160100	-1.86037800	2.83376700
H	2.26150100	-1.30430300	2.01173800
H	2.41908100	-1.41331600	3.78569300
H	2.39879700	-2.90647700	2.80301800
C	1.08376400	-2.82688400	-2.05790100
C	-0.26445900	3.40725200	-1.79908200
H	-0.12496800	2.41876000	-2.26430100
H	0.53565200	3.52925300	-1.05214800
H	-0.14451900	4.19116700	-2.57150000
C	4.98215100	-2.38446000	3.95265900
H	4.71889200	-3.44874200	4.08793200
H	4.66949900	-1.84536300	4.86435800
H	6.08088400	-2.31465700	3.86786600
C	5.00645400	3.89349000	-3.02993500
H	4.33282600	4.69206300	-3.38849100
H	5.38544300	3.35670500	-3.91767600
H	5.86238300	4.37271900	-2.52312400
C	3.08772200	2.22783400	-2.79388200
H	2.57838800	1.53328900	-2.09795000
H	3.43087300	1.65824700	-3.67509600
H	2.34295500	2.97042800	-3.12804500
C	4.14054200	3.91216400	4.14401000
H	4.98160000	4.57837400	3.88290400
H	4.40399300	3.36655600	5.06705300
H	3.26447300	4.54334900	4.37635200
C	7.42858200	-2.10267300	-2.80597900
H	8.25614500	-2.48042700	-2.18050200
H	7.84560200	-1.38919500	-3.53780200
H	7.01581400	-2.95263900	-3.37801300
C	-1.85179700	4.91435400	-0.54179600
H	-1.92120200	5.66071900	-1.35506100
H	-1.00242100	5.19146500	0.10766800
H	-2.77170800	4.98300900	0.06181000
C	-2.03377800	-1.15139900	-3.55728000
H	-2.38245800	-0.40624400	-2.82148500
H	-2.73330600	-1.12587700	-4.41763900
H	-1.04667000	-0.83089700	-3.92605200
C	2.64458800	2.00197700	3.36217700
H	1.72326000	2.59556800	3.49618900
H	2.84189500	1.44237500	4.29418800
H	2.44713900	1.28311000	2.54693300
C	5.17583600	-0.92547400	-2.83423600
H	4.71133100	-1.77769600	-3.36009200
H	5.52947400	-0.20302700	-3.59135700
H	4.39618000	-0.43979400	-2.22473000
C	-1.23996700	-4.03056400	-0.11506900
C	-0.10155600	-4.16738500	0.92199700
H	0.80058600	-4.63591400	0.49496200
H	-0.44316800	-4.80829500	1.75948900
H	0.18392500	-3.18666700	1.34384100
C	-3.45617500	-2.84746300	-2.44794500
H	-3.57826300	-3.86613300	-2.04428000
H	-4.16441400	-2.74343400	-3.29286800
H	-3.73731000	-2.11984100	-1.66861300
C	-2.02134000	-2.57446300	-2.94390400
C	-2.50329700	-3.62833800	0.68169300
H	-2.38178600	-2.65524200	1.18564400
H	-2.70081100	-4.39131900	1.46111600
H	-3.40010500	-3.55866800	0.05078400
C	1.33351500	-1.73486900	-3.12429600
H	0.71736800	-1.87518300	-4.02750900
H	2.39410800	-1.76019900	-3.44015000
H	1.13073800	-0.72901300	-2.71153500
C	2.08322500	-2.55926200	-0.91348600
H	1.83739400	-1.61653100	-0.37885600
H	3.10363500	-2.46349800	-1.32630800
H	2.10936900	-3.36325400	-0.16665300

4

TPSS-D3(BJ)/def2-SVP

E = -3993.035334 a.u.

P	-0.03234200	0.44234600	1.03089000
Si	-0.78665300	-2.56702900	-1.61973500
Si	-1.26724900	-0.70541700	-0.21196600
N	-3.79824100	1.82535800	-0.25313000
N	-2.95899800	-0.44847600	-0.09142900
N	-4.43898500	0.62152600	1.48040400
C	-2.24123900	2.93661600	-1.82803700
C	-3.89104800	2.09785500	-3.96875100
C	-1.26909200	3.33460100	-0.72255500
C	-4.71218900	2.60926000	0.49092000
C	-3.56494400	-2.61267300	-2.45992700
C	-4.21369600	1.68386100	-2.66548100

C	-2.45242900	-3.55568200	-1.94984400	H	1.40896900	-0.28885200	-3.88000100
C	-1.34583500	-0.79186400	4.21020200	H	0.53047100	0.10947600	-2.37285200
C	-4.07889500	-1.85751700	4.24736500	H	1.85517700	-1.06931800	-2.33886100
C	-0.01384300	-1.82602100	-3.26911600	H	-6.52847700	0.00351500	0.55118800
C	-3.39178500	2.14348300	-1.59954000	H	-2.11353200	0.24530300	2.52508100
C	-2.30434100	-4.69454500	-2.98412500	H	-5.11755000	0.08356200	-1.59601900
C	-2.79232200	2.92971900	-4.21166400	H	-6.02459900	4.29165500	0.61408000
C	1.00467300	-0.71175700	-2.93793600	H	-5.33618800	4.01775500	-1.01288200
C	-6.78759200	-0.98426000	0.96778900	H	-4.33137700	4.73160300	0.26452500
C	-2.47604700	0.00315800	3.54090900	H	-0.71636800	5.01820100	0.54217500
C	-3.63607300	0.57259300	0.34599300	H	-2.46647400	4.93305100	0.21741900
C	-5.40375900	0.75403100	-2.42401400	H	-1.35392500	5.50449200	-1.05655800
C	-5.11905200	3.98295400	0.06882200	H	1.06373700	-2.44582900	-5.06794300
C	-3.76391500	-0.79153000	3.38536500	H	1.54529900	-3.35353700	-3.61232900
C	-1.47097300	4.78048200	-0.22867300	H	-0.00437100	-3.71768400	-4.41595800
C	0.68257100	-2.90239000	-4.13170000	H	-6.43101000	-0.92415000	-3.34929100
C	-5.71909100	-0.13329800	-3.64091100	H	-6.18731100	0.44716000	-4.45657100
C	-1.12186000	-1.15404600	-4.11338700	H	-4.81361700	-0.61857000	-4.03686200
C	0.19127800	3.14893700	-1.17687200	H	-0.65691600	-0.58504500	-4.94341600
C	-2.70102200	1.34520100	4.26484600	H	-1.80632700	-1.89136700	-4.56398400
C	0.14678300	-3.68784200	0.88530500	H	-1.72102200	-0.44110000	-3.51793800
C	-1.97310200	3.32838500	-3.15499300	H	0.86302700	3.21413400	-0.30625900
C	0.55865700	-5.13186500	-1.13216100	H	0.49678700	3.92679500	-1.90123000
C	-5.87241100	-1.24027700	2.15939400	H	0.34216000	2.15764600	-1.63031500
C	-8.27980400	-0.95186500	1.34115000	H	-3.42046700	1.98016000	3.72243100
C	-6.06908900	2.16152900	2.66754300	H	-3.07796200	1.18925400	5.29271200
C	1.92686200	-3.09231700	-0.72842100	H	-1.74709200	1.89876000	4.32192800
C	-6.50178600	-2.03291000	-0.12627900	H	0.89681800	-4.29380500	1.43335200
C	-5.10780900	1.86103400	1.56564000	H	0.16260000	-2.66526800	1.30246900
C	-6.14286000	-2.29378600	3.05121700	H	-0.84612600	-4.12162600	1.08554600
C	-5.25799300	-2.59522100	4.09033000	H	1.35385900	-5.67951300	-0.58666200
C	-2.94331300	-4.13884300	-0.60345800	H	-0.38781200	-5.67062800	-0.95817100
C	0.50323300	-3.67415700	-0.62185800	H	0.79539500	-5.19213800	-2.20910800
C	-4.68799300	-0.49254700	2.35505900	H	-8.63484300	-1.93705900	1.69269600
C	-6.67997700	1.51407300	-2.00246600	H	-8.48264500	-0.21594300	2.13897000
H	-1.42866000	2.64493200	0.12548300	H	-8.88534100	-0.67824900	0.45888700
H	-4.53130500	-3.15578100	-2.45535800	H	-6.43654400	3.19544200	2.57792300
H	-3.66293300	-1.72173400	-1.81842400	H	-5.59814200	2.04588000	3.65975300
H	-3.38428800	-2.28440200	-3.49332000	H	-6.93867000	1.48043200	2.64304900
H	-0.40387100	-0.22646800	4.11017000	H	2.31757400	-3.11001900	-1.75803300
H	-1.53440300	-0.96501200	5.28668000	H	1.96566200	-2.04816900	-0.35844800
H	-1.19772400	-1.76676600	3.71520300	H	2.61649600	-3.68560300	-0.10093800
H	-3.27382000	-5.22279500	-3.09019200	H	-7.10067700	-1.82785500	-1.03161900
H	-1.54812000	-5.44330300	-2.70107600	H	-6.75402600	-3.04791900	0.23035900
H	-2.03855400	-4.30182500	-3.98142400	H	-5.43578000	-2.02382300	-0.39948100

H	-3.93777100	-4.60811400	-0.74498000
H	-2.26656100	-4.91457900	-0.20768200
H	-3.05423300	-3.34647700	0.15852100
H	-7.51878200	0.80456200	-1.88632100
H	-6.55071900	2.03633300	-1.04252800
H	-6.96357800	2.25509300	-2.77142200
H	-3.38515500	-2.11648100	5.05208400
H	-5.48126100	-3.41957600	4.77654500
H	-7.05075000	-2.89174000	2.91982500
H	-1.09295300	3.94639800	-3.35704200
H	-2.56332500	3.24780300	-5.23467300
H	-4.50568700	1.76179800	-4.80824000
C	4.28675600	0.47955000	0.31384000
N	5.31978700	-0.37739100	0.58365500
N	4.86177300	1.71965600	0.40675000
C	6.50617200	0.30952800	0.84248100
C	5.17988700	-1.81155800	0.67583600
C	6.21524600	1.64017800	0.72890200
C	4.12519200	2.95687400	0.27961100
H	7.43077600	-0.21059500	1.08352300
C	5.61720100	-2.59791100	-0.41573100
C	4.63851300	-2.36152100	1.85782800
H	6.83325600	2.52786100	0.84356800
C	4.08630200	3.59053000	-0.98203700
C	3.48196800	3.47649000	1.42554200
C	6.10975700	-1.93911800	-1.70157600
C	5.52037300	-3.99305300	-0.28451100
C	4.56076500	-3.76509100	1.93495300
C	4.18189700	-1.49912300	3.02940300
C	3.42090000	4.82580700	-1.06378400
C	4.64488800	2.90334900	-2.22232600
C	2.82712800	4.71382900	1.28781700
C	3.50230800	2.74417800	2.76267400
H	6.61294500	-0.99700400	-1.41805100
C	4.90922000	-1.56050100	-2.59593600
C	7.13083000	-2.78872800	-2.47434400
H	5.84503100	-4.63710700	-1.10650900
C	5.00281700	-4.57091800	0.88205500
H	4.14463100	-4.22844900	2.83523100
H	4.22818000	-0.44511500	2.70860000
C	5.13794300	-1.66028600	4.22894100
C	2.71851800	-1.78287900	3.41698800
H	3.36604900	5.34551800	-2.02507600
C	2.80974400	5.38809500	0.06251100
H	5.39615100	2.16652000	-1.88743200
C	5.34388100	3.86801700	-3.19437300
C	3.50899000	2.11989800	-2.91629000
H	2.31698700	5.14839700	2.15289000
H	3.79833200	1.70072300	2.55969100
C	2.11170300	2.69156200	3.42100200
C	4.55958300	3.35647800	3.70476700
H	4.37745600	-2.46644900	-2.93461200
H	5.25080300	-1.00590300	-3.48800000
H	4.18973600	-0.92972000	-2.04950200
H	7.97613500	-3.09219800	-1.83235200
H	7.53233200	-2.21137300	-3.32513400
H	6.66976300	-3.70264500	-2.88928000
H	4.93239700	-5.66065000	0.96318100
H	5.12966900	-2.69753500	4.60968100
H	4.83210000	-0.99301800	5.05435700
H	6.17664200	-1.41051100	3.94910200
H	2.04140700	-1.59293100	2.56615600
H	2.41207100	-1.11707700	4.24262500
H	2.57953800	-2.82564300	3.75381100
H	2.29160900	6.34904000	-0.02228300
H	4.63238700	4.58626600	-3.63911700
H	5.79952500	3.30218300	-4.02569500
H	6.13961600	4.44351700	-2.68988100
H	3.02891800	1.42019900	-2.21138300
H	3.89811300	1.54168700	-3.77279000
H	2.72964300	2.80964700	-3.28493100
H	1.76650900	3.69515100	3.72868300
H	2.15312100	2.06321600	4.32831100
H	1.36466500	2.25396100	2.73582600
H	5.56491200	3.33641900	3.24867300
H	4.60152700	2.79369500	4.65442500
H	4.31475700	4.40812400	3.93971200
Ag	2.20230200	0.20058800	0.33652100

5

TPSS-D3(BJ)/def2-SVP

E = -3981.860010 a.u.

Au	-2.13151000	0.26920400	-0.27203000
P	0.12340000	0.61656800	-0.74015200
Si	1.00622000	-2.67203700	1.53105900
Si	1.40950400	-0.63547800	0.35703600
N	4.01142400	1.89377900	0.29405500
N	3.08227500	-0.35235200	0.14792800
N	4.64873700	0.62791800	-1.38759700
C	2.32695400	3.28933000	1.42343000

C	3.41073300	2.48749800	3.91798500	H	1.58005400	-0.77396700	-5.11566000
C	1.64632100	3.63746600	0.09970900	H	1.21622700	-1.64046100	-3.58687200
C	4.99118800	2.61755400	-0.42523400	H	3.57043100	-5.34817200	2.79324700
C	3.75297600	-2.67095700	2.44502500	H	1.87326600	-5.60339700	2.31089900
C	3.93661300	1.92492900	2.74210700	H	2.25853800	-4.58565000	3.72461100
C	2.70879300	-3.60853200	1.80007600	H	-1.38422700	-0.80318500	3.94218800
C	1.40403200	-0.64823000	-4.03062100	H	-0.37695800	-0.13983000	2.61956800
C	4.05159300	-1.89293600	-4.06732900	H	-1.65276200	-1.31013500	2.25094600
C	0.13780800	-2.19287100	3.22524800	H	6.86664300	0.03160200	-0.66455700
C	3.39954600	2.36636600	1.50607200	H	2.22473000	0.26416200	-2.30175700
C	2.58056900	-4.85605200	2.70361300	H	4.75406900	0.10375500	2.02896100
C	2.39370600	3.44715600	3.86321900	H	5.95502600	4.49786200	-0.79524600
C	-0.87809600	-1.05272100	2.98764500	H	6.32600200	3.79288500	0.80252800
C	6.97310000	-1.02380200	-0.96865600	H	4.74501100	4.54443200	0.51828500
C	2.57091600	0.06226300	-3.33164100	H	1.66998400	5.05691400	-1.54482500
C	3.80764500	0.63922600	-0.27993100	H	3.29577600	4.53101100	-1.06501200
C	5.02501500	0.85152500	2.79424300	H	2.39969900	5.70336200	-0.04766800
C	5.52909700	3.93153600	0.04857200	H	-1.02471500	-3.08314500	4.84767400
C	3.82925100	-0.78903900	-3.22462700	H	-1.40358100	-3.77740300	3.25134900
C	2.30186700	4.79872800	-0.67624600	H	0.10661000	-4.23164500	4.08282900
C	-0.58218200	-3.39377100	3.87930000	H	5.74816500	-0.75397200	4.06576200
C	5.07911200	0.11964100	4.14499600	H	5.47543700	0.76991100	4.94623400
C	1.17377000	-1.64106200	4.23031500	H	4.08424100	-0.24077000	4.45330300
C	0.14750300	3.92692000	0.28665100	H	0.64363200	-1.23299900	5.11432600
C	2.84527800	1.43038600	-3.98832200	H	1.86553600	-2.41978600	4.59227100
C	0.23102700	-3.51888800	-1.13169800	H	1.76907200	-0.81935500	3.79267200
C	1.85102400	3.82968400	2.63385600	H	-0.34571900	3.95187000	-0.69893500
C	-0.19319300	-5.20877800	0.68215900	H	-0.02800500	4.90466900	0.77333700
C	5.99023100	-1.28466600	-2.10594200	H	-0.34586100	3.13663300	0.87536600
C	8.44217700	-1.24575100	-1.36397600	H	3.59917100	2.00643800	-3.42708700
C	6.36183500	2.07927400	-2.57290300	H	3.19955800	1.31034000	-5.02880400
C	-1.63699400	-3.18804000	0.45530900	H	1.91542500	2.02582800	-4.00182400
C	6.57587800	-1.87976300	0.25037900	H	-0.45674600	-4.10085900	-1.77831500
C	5.37810100	1.83172800	-1.47728000	H	0.16038100	-2.45855000	-1.43168100
C	6.16825900	-2.37229200	-2.97975600	H	1.25718400	-3.86421200	-1.33604600
C	5.20971100	-2.67108100	-3.95279900	H	-0.94209600	-5.71914000	0.04320000
C	3.26992000	-4.02478300	0.41975900	H	0.78251700	-5.68475600	0.48660000
C	-0.18678300	-3.69946100	0.34878900	H	-0.46707700	-5.40620000	1.73335400
C	4.82214100	-0.50187500	-2.25676800	H	8.65234800	-2.30822500	-1.58126000
C	6.42622000	1.39337500	2.44098500	H	8.71525700	-0.65693200	-2.25753900
H	1.70802900	2.72957500	-0.52796000	H	9.10623000	-0.94385400	-0.53498000
H	4.74654100	-3.16174500	2.42431800	H	6.69939200	3.12706400	-2.54571700
H	3.82592700	-1.72123600	1.89128100	H	5.91113600	1.88234700	-3.56178500
H	3.52227100	-2.44925100	3.49606800	H	7.25240800	1.43010200	-2.48818100
H	0.48822800	-0.04871800	-3.89447100	H	-2.07452000	-3.35401200	1.45212200

H	-1.70909200	-2.10861500	0.21782900
H	-2.26780100	-3.72584900	-0.27486300
H	7.22592900	-1.65742600	1.11562200
H	6.66389900	-2.95601400	0.01735400
H	5.53118800	-1.68205500	0.53340800
H	4.27910900	-4.46463300	0.54973900
H	2.64268800	-4.78314900	-0.07763900
H	3.36961300	-3.15386600	-0.25232900
H	7.17817400	0.59322700	2.55942300
H	6.47772500	1.74448800	1.39896000
H	6.70677200	2.22775200	3.10878500
H	3.30458100	-2.14837400	-4.82372800
H	5.36076600	-3.52442800	-4.62302900
H	7.05987400	-3.00011800	-2.88655300
H	1.02511000	4.54600800	2.60974800
H	2.00413900	3.88100600	4.79078500
H	3.79829600	2.17055800	4.88988700
C	-4.18648500	0.38393300	-0.25529700
N	-5.16239200	-0.53860700	-0.52968500
N	-4.86128200	1.57661000	-0.22163200
C	-6.41117800	0.06809000	-0.67241300
C	-4.93704400	-1.95224700	-0.71559900
C	-6.21990400	1.40654100	-0.47598800
C	-4.21639800	2.85677800	-0.04430200
H	-7.30420200	-0.50894900	-0.90255600
C	-5.30279200	-2.82726600	0.33506900
C	-4.41359300	-2.39855100	-1.94762800
H	-6.91012800	2.24690000	-0.49417500
C	-4.05446400	3.35742300	1.26553500
C	-3.77665800	3.54735400	-1.19496900
C	-5.78840500	-2.27411100	1.67318800
C	-5.15250200	-4.20489200	0.10704100
C	-4.28274900	-3.78957600	-2.12217200
C	-4.02058900	-1.44245800	-3.06806200
C	-3.47035000	4.62887300	1.40138700
C	-4.40501300	2.51792400	2.48753900
C	-3.20756100	4.81854100	-1.00402200
C	-3.86522000	2.93806700	-2.58954400
H	-6.39302800	-1.37447900	1.45689700
C	-4.58894700	-1.81713900	2.53173600
C	-6.68473100	-3.24882200	2.45238000
H	-5.42139600	-4.91564300	0.89320600
C	-4.65527500	-4.68156000	-1.11307500
H	-3.87977400	-4.17412900	-3.06466300
H	-4.13611800	-0.41395600	-2.68809500
C	-4.96057100	-1.59954900	-4.28028900
C	-2.54017200	-1.60883400	-3.46092000
H	-3.32087200	5.04753200	2.40171000
C	-3.06651000	5.35885400	0.27824900
H	-5.03717500	1.67949500	2.14722500
C	-5.20348500	3.30068100	3.54456100
C	-3.11398900	1.91016000	3.07630500
H	-2.85488900	5.38437200	-1.87259600
H	-4.30222800	1.93044700	-2.48736500
C	-2.46018300	2.75979200	-3.20056700
C	-4.79415700	3.75499400	-3.50821200
H	-3.96471800	-2.68121600	2.81706200
H	-4.94095600	-1.32576400	3.45628200
H	-3.94974800	-1.10647500	1.98307200
H	-7.53060700	-3.60473500	1.83859400
H	-7.09354200	-2.74814800	3.34707000
H	-6.11897300	-4.13069000	2.80218600
H	-4.54484400	-5.75958800	-1.27035600
H	-4.88103500	-2.60955700	-4.72127000
H	-4.69920700	-0.86656900	-5.06422900
H	-6.01458600	-1.43767900	-3.99314500
H	-1.88342700	-1.42319300	-2.59379600
H	-2.27449900	-0.88048500	-4.24706400
H	-2.33325600	-2.62108500	-3.85164200
H	-2.61533300	6.34842300	0.40533600
H	-4.60907500	4.12581400	3.97577200
H	-5.48946200	2.63202600	4.37551300
H	-6.12429500	3.73292700	3.11511400
H	-2.56844000	1.33643100	2.30647100
H	-3.34942600	1.23356600	3.91663900
H	-2.44028000	2.70363600	3.44670800
H	-1.96744000	3.73499500	-3.36482400
H	-2.53316300	2.24837400	-4.17713400
H	-1.81701000	2.15456900	-2.53834000
H	-5.80885300	3.83720600	-3.08026300
H	-4.87498900	3.27392400	-4.49920000
H	-4.40747600	4.77870800	-3.66068500

6

TPSS-D3(BJ)/def2-SVP

E = -3657.461876 a.u.

Au	-2.47928300	0.05234400	-0.49867700
P	-0.33936600	-0.76863600	-0.06409900
Si	1.09122000	3.02719400	-0.72501700

Si	1.17476200	0.65286900	-0.42820800
N	3.74002600	-1.99449800	-0.68710900
C	-5.03126000	-2.47472800	0.68792500
C	-4.44899000	-1.66360400	3.32616000
N	2.77028500	0.15786600	-0.08402400
C	-3.01483700	1.05119500	3.02390800
N	4.07211900	-1.25404000	1.35468600
C	-5.05609800	-1.10503400	1.05536600
N	-5.39558500	-0.12806700	0.03611400
C	2.24839000	-3.04529500	-2.33196800
C	-6.83284500	0.31351600	-0.21550600
C	-4.50879400	0.79018800	2.73696800
C	-6.19281800	-4.08141600	-0.92450100
C	3.68201000	-1.72977300	-4.39551900
C	1.37455300	-3.64567600	-1.22905600
C	4.60151600	-2.91402500	-0.04410500
C	-4.71660300	-0.67424000	2.36214100
C	3.88767200	2.96927300	-1.45844100
C	4.00989900	-1.44609500	-3.05842400
C	2.90541600	3.75613900	-0.56258700
C	-7.30312200	1.23523000	0.92095400
C	0.40054500	-0.72198500	3.71100400
C	2.92947200	0.55085700	4.40353900
C	0.30996400	3.38179400	-2.48850300
C	-4.48564900	-3.02238300	3.00478100
C	-5.16857700	-2.94336300	-0.75812000
C	3.30802300	-2.14834500	-2.04866900
C	3.00086600	5.24695000	-0.96081600
C	2.69345600	-2.66998700	-4.70705500
C	-0.88263900	2.43221100	-2.74043800
C	-4.75280500	-3.41868800	1.69128200
C	-6.66272300	1.05242100	-1.56149700
C	6.44618300	0.31190500	1.85095100
C	1.67402600	-1.17494900	2.98479100
C	3.42450300	-0.93746900	0.16369000
C	5.05858500	-0.39361700	-2.69857700
C	5.21779700	-4.06919400	-0.76695500
C	2.90232200	-0.33018000	3.30771900
C	1.91387200	-4.95400600	-0.61425200
C	-0.16949700	4.84338800	-2.63852200
C	5.22228900	0.67667500	-3.78982900
C	1.34232600	3.08855800	-3.60072700
C	-0.07417800	-3.85690000	-1.70114800
C	1.91093200	-2.67988700	3.22952600
C	0.24053700	2.86904900	2.02862200

C	1.97434900	-3.30269600	-3.68876500
C	0.17163900	5.18501200	1.03386600
C	5.22863900	0.36610700	2.77123800
C	-4.62764000	1.15237200	-3.12154500
C	7.77964700	0.32462500	2.61824600
C	5.56987300	-3.03536900	2.37126400
C	-1.54611800	3.48428900	0.42748600
C	6.38529000	1.44544300	0.81008500
C	4.79604100	-2.45791000	1.23185200
C	-3.78556800	-3.35869100	-1.30338600
C	5.20353500	1.22673000	3.88375800
C	4.06418400	1.32074600	4.68817100
C	3.39002500	3.58506100	0.89641300
C	-0.05004900	3.68301100	0.74434800
C	-5.15229700	1.39984600	-1.69358500
C	-7.78425100	-0.88486300	-0.30941300
C	-4.47064900	0.45228800	-0.71831300
C	4.06803300	-0.39659600	2.50428700
C	-5.37105600	1.22689300	3.93703000
C	6.42980500	-1.00545100	-2.34420400
H	-2.84617300	2.12429500	3.21953100
H	-2.67769500	0.48462600	3.90965800
H	-2.38987100	0.74927100	2.16568200
H	-4.78299600	1.40619100	1.86582600
H	-6.31472500	-4.33001800	-1.99357700
H	-5.85912000	-4.99963900	-0.40959600
H	-7.18102200	-3.80794300	-0.51685000
H	1.32775700	-2.88696500	-0.42627400
H	4.92230100	3.31118600	-1.25516300
H	3.83296400	1.88966800	-1.24608500
H	3.69794900	3.12572900	-2.52967900
H	-8.33240300	1.57154600	0.70967200
H	-7.31011800	0.69534500	1.88127600
H	-6.66262900	2.12501900	1.02115000
H	-0.46322600	-1.26274200	3.28918000
H	0.44534900	-0.93131600	4.79658800
H	0.21466200	0.35476100	3.56952100
H	-5.50724600	-2.08581200	-1.36228400
H	4.03878900	5.60261200	-0.79925600
H	2.33316000	5.89574600	-0.37198700
H	2.76896500	5.39894300	-2.02939100
H	-1.30539200	2.62959200	-3.74678000
H	-0.57667900	1.37201800	-2.69950800
H	-1.69022300	2.55844700	-2.00364700
H	-7.30797700	1.94439500	-1.61799100

H	-6.95588600	0.37815100	-2.38465500
H	6.39818400	-0.63383100	1.28554100
H	1.46591000	-1.05023400	1.90542100
H	4.68100600	0.11830700	-1.79690100
H	5.64480500	-4.78274000	-0.04460600
H	6.02925300	-3.73517200	-1.43921100
H	4.48361600	-4.60207000	-1.39091800
H	1.16058200	-5.36719200	0.07994600
H	2.83572100	-4.79205300	-0.03530000
H	2.11169900	-5.71514600	-1.39155500
H	-0.57173200	5.00224300	-3.65969800
H	-0.97590300	5.08917700	-1.92725400
H	0.64697900	5.56935500	-2.48716000
H	5.85237900	1.49959100	-3.41194100
H	5.71370100	0.27045300	-4.69265800
H	4.24884500	1.10079800	-4.08745200
H	0.84316600	3.14431200	-4.58885300
H	2.16890900	3.81824300	-3.60796300
H	1.77310000	2.07527600	-3.50236800
H	-0.70869000	-4.08369300	-0.82823600
H	-0.15949600	-4.70179500	-2.41042300
H	-0.47458600	-2.94425500	-2.17185500
H	2.73371200	-3.07338600	2.61193600
H	2.14246300	-2.87860100	4.29224500
H	0.99940700	-3.24242700	2.96179700
H	-0.37796700	3.25863400	2.86255300
H	-0.02328800	1.80739600	1.87944700
H	1.29466500	2.91472100	2.34378700
H	-0.53145100	5.51143600	1.82676800
H	1.19146200	5.39759400	1.39412700
H	-0.01636900	5.81426000	0.14640000
H	-5.14498800	1.81734700	-3.83658300
H	-3.54435800	1.34797400	-3.17233400
H	-4.80005600	0.10610900	-3.42776200
H	7.94962000	1.29010200	3.12727700
H	7.81517100	-0.47114300	3.38315600
H	8.62018200	0.17044100	1.91884500
H	5.93084700	-4.04188700	2.10872500
H	4.93954600	-3.11564000	3.27457600
H	6.44483100	-2.41684900	2.64056800
H	-1.87849600	4.09979500	-0.42519100
H	-1.78278500	2.42478000	0.21105600
H	-2.15144500	3.78647800	1.30564200
H	7.23147900	1.36913300	0.10377900
H	6.43211600	2.43338700	1.30096500
H	5.44625500	1.39607400	0.23944600
H	-3.86004900	-3.63935900	-2.36934400
H	-3.06053500	-2.53156900	-1.20744800
H	-3.38870200	-4.22558200	-0.74609600
H	4.44589700	3.91143400	0.97174700
H	2.81008500	4.19506800	1.60802500
H	3.34012700	2.52946300	1.21612000
H	-8.81165500	-0.51240200	-0.46171600
H	-7.53052700	-1.53977000	-1.15628300
H	-7.76861100	-1.48067700	0.61878300
H	-6.44497000	1.03766300	3.76965700
H	-5.07543500	0.68741600	4.85435800
H	-5.23678100	2.30601500	4.12960600
H	7.16089200	-0.20160400	-2.14619900
H	6.37285200	-1.63202100	-1.44020500
H	6.81511700	-1.62215700	-3.17615000
C	-4.83347300	2.85270500	-1.27148200
H	-5.16566000	3.06407600	-0.24135300
H	-3.74836700	3.03170500	-1.31843500
H	-5.33884700	3.55899000	-1.95370300
H	2.04765900	0.63903200	5.04340100
H	4.05757100	2.00188800	5.54614700
H	6.08161800	1.83814500	4.11420100
H	1.16822800	-3.99381800	-3.95007200
H	2.46050200	-2.88807500	-5.75509600
H	4.20307900	-1.20842400	-5.20331000
H	-4.18230600	-1.35603400	4.34258300
H	-4.27334900	-3.77387800	3.77251100
H	-4.72421300	-4.48166700	1.43080200

3_{iso}

TPSS-D3(BJ)/def2-SVP

E = -5486.483472 a.u.

Si	0.53711200	0.48903400	-0.07178600
P	1.60703900	2.33967700	-0.02586000
Si	0.13859700	4.03265600	-0.08100400
C	-3.32909000	-1.16843400	0.18404400
C	-5.00592900	-2.73110400	0.26609500
H	-5.64662200	-3.51751400	-0.12665200
C	-4.95311900	-2.12820000	1.48803900
H	-5.53092000	-2.28432100	2.39633400
N	1.67508000	-0.77170100	0.21944300
C	4.91525400	-2.20392600	-0.04247900
C	3.09138600	-0.16692700	-2.39767200
C	1.05694900	-1.65326600	-2.88750900

H	1.10540500	-1.90145400	-1.81347000
N	-4.01143900	-2.13757000	-0.51226400
N	-3.93756600	-1.17663000	1.41633300
C	-3.64137000	-0.26076600	2.49571100
C	-2.42146800	-0.38940300	3.20128000
C	-2.21150900	0.49663300	4.27270200
H	-1.27562600	0.44236400	4.83353700
C	-3.17501900	1.44423200	4.63113100
H	-2.98709000	2.11816100	5.47323400
C	-4.36725000	1.55165700	3.90817800
H	-5.09926900	2.31426800	4.18668800
C	-4.62477400	0.70818800	2.81365100
C	-5.87636300	0.88233900	1.95208800
H	-6.20848900	-0.12106600	1.63362500
C	-5.54306700	1.67056900	0.66606500
H	-5.29026900	2.71655900	0.90749400
H	-6.40985900	1.67138300	-0.01889700
H	-4.68059500	1.23190400	0.13905000
C	-7.05445000	1.52643000	2.69902300
H	-7.28842800	0.98678800	3.63324200
H	-7.95381200	1.51497700	2.05919000
H	-6.84877600	2.58119900	2.95376200
C	-1.38692500	-1.45536800	2.84498300
H	-1.32744400	-1.48713900	1.73621700
C	-1.83513200	-2.84819800	3.33335400
H	-1.93541600	-2.85971900	4.43367800
H	-1.08478900	-3.60614000	3.04957400
H	-2.80424000	-3.14595800	2.89758000
C	0.02735500	-1.12686700	3.33837900
H	0.34148700	-0.12272000	3.00899200
H	0.74653700	-1.84358300	2.91323800
H	0.10616700	-1.18043500	4.43896900
C	-3.74753300	-2.55909900	-1.86803400
C	-3.07827700	-3.79082800	-2.05968300
C	-2.52073000	-4.55585700	-0.86664000
H	-3.23754100	-4.44279400	-0.03375300
C	-1.19272000	-3.92054800	-0.40850700
H	-0.40059000	-4.09217500	-1.15682400
H	-0.87268000	-4.36802600	0.54436400
H	-1.27927200	-2.82845900	-0.26210800
C	-2.34875000	-6.06133400	-1.11780000
H	-3.28607300	-6.52683500	-1.46959200
H	-2.04289100	-6.56303600	-0.18361000
H	-1.56244800	-6.26143800	-1.86740200
C	-2.88435400	-4.22260800	-3.38226300

H	-2.36758300	-5.16741500	-3.57154500
C	-3.32841800	-3.44903100	-4.46208600
H	-3.15782900	-3.79671600	-5.48631200
C	-3.98539700	-2.23548000	-4.24030700
H	-4.32932200	-1.64256100	-5.09316700
C	-4.22762800	-1.77035800	-2.93399500
C	-4.94464100	-0.45050700	-2.68429800
H	-5.32308300	-0.47255200	-1.64742700
C	-6.15309800	-0.23509400	-3.61163700
H	-5.84452000	-0.10617600	-4.66420400
H	-6.69358500	0.68062300	-3.31483600
H	-6.85670800	-1.08434300	-3.56253000
C	-3.93877500	0.71064100	-2.78215600
H	-3.11673500	0.56793700	-2.05890900
H	-4.42293500	1.67769500	-2.56238000
H	-3.49254300	0.76386000	-3.79053200
C	2.85318500	-1.15968200	-0.12226000
N	3.48084500	-1.10970400	-1.38291200
C	4.72395200	-1.78373600	-1.32968900
C	5.52161400	-2.06353100	-2.56290300
H	5.36380800	-1.28014400	-3.31996900
H	5.22508400	-3.02616300	-3.02213300
H	6.59899700	-2.11478900	-2.33620400
C	6.08070000	-2.89441800	0.58685900
H	6.92269600	-2.92675700	-0.12235400
H	5.85581400	-3.93190300	0.89569500
H	6.41001900	-2.35376300	1.49263800
N	3.77652800	-1.81500700	0.69871700
C	3.90103700	0.51530400	2.43354500
H	3.36359500	0.61423400	1.47498000
C	3.35786500	1.63322200	3.33474700
H	3.92571100	1.72456100	4.27948600
H	2.29429800	1.46894100	3.57542500
H	3.42621300	2.59367300	2.79761300
C	5.40799100	0.70966900	2.16322900
H	5.59642800	1.72456000	1.77512000
H	5.77949300	-0.01272400	1.41676000
H	5.99296600	0.57848100	3.09184800
C	3.27810900	-2.41454300	4.84563800
H	3.15930000	-2.57231900	5.92324700
C	3.47343800	-1.12147500	4.34777000
H	3.50239200	-0.27648400	5.04128600
C	3.63817300	-0.88627000	2.97093100
C	3.59120600	-2.00773600	2.10640600
C	3.36746200	-3.32079100	2.58489900

C	3.22299500	-3.50499300	3.97127600
H	3.05083700	-4.50962200	4.36930400
C	3.16700400	-4.47345700	1.60789900
H	3.76490200	-4.26326600	0.70568200
C	1.69426900	-4.48985700	1.15899800
H	1.03470700	-4.72406000	2.01454600
H	1.52981500	-5.25214000	0.37650600
H	1.39939800	-3.50422100	0.76243500
C	3.61034000	-5.83961400	2.15227700
H	4.65610800	-5.81459000	2.50609100
H	3.53193000	-6.60544700	1.36070300
H	2.97513800	-6.17247600	2.99277800
C	1.87488500	-0.38107300	-3.09492300
C	1.46868100	0.58854400	-4.02696500
H	0.52849000	0.45469300	-4.56759800
C	2.24402100	1.72561800	-4.27200000
H	1.90425300	2.47373600	-4.99541600
C	3.44208400	1.91246200	-3.58180600
H	4.03581600	2.81409100	-3.76533100
C	3.89122500	0.98289100	-2.62625100
C	5.19620100	1.27385500	-1.88469800
H	5.38518400	0.44396300	-1.18385600
C	6.39343900	1.36114900	-2.85632700
H	6.48937800	0.46243500	-3.48751900
H	7.33395700	1.48603300	-2.29037800
H	6.29080100	2.23235200	-3.52806600
C	5.10906500	2.56838000	-1.05436700
H	4.96532700	3.44760100	-1.70723900
H	6.04735700	2.71743200	-0.49037300
H	4.25883700	2.52969700	-0.35405400
C	1.67608600	-2.82836500	-3.67239100
H	1.65304800	-2.63105000	-4.75952500
H	1.11387000	-3.76071800	-3.47990300
H	2.72552400	-2.99337500	-3.37601800
C	-0.42564100	-1.47382100	-3.24044500
H	-0.86050200	-0.62659000	-2.67805100
H	-0.98999100	-2.38015300	-2.97580400
H	-0.58403200	-1.29568900	-4.31908200
C	-1.17508900	3.87262500	1.37724600
C	-1.86260000	5.21033500	1.73523800
H	-2.36284600	5.67288600	0.86753200
H	-1.14902800	5.94388400	2.14698400
H	-2.63637400	5.03513200	2.51107700
C	-2.27643900	2.86651300	0.99231500
H	-2.93730300	2.68676000	1.86001500
H	-1.84612400	1.88162900	0.70668400
H	-2.90218500	3.21679300	0.15585500
C	-0.50150300	3.30784900	2.64978400
H	0.31763200	3.94304000	3.01957800
H	-0.08302800	2.30667500	2.45438800
H	-1.25721900	3.21985100	3.45484000
C	-0.75579300	4.12949600	-1.83259800
C	-1.98941900	5.05900200	-1.84463800
H	-2.79151500	4.69395500	-1.18075200
H	-2.41156500	5.11031700	-2.86959200
H	-1.74077800	6.08940500	-1.53822200
C	-1.18505000	2.71034900	-2.25690900
H	-0.30168100	2.07390600	-2.43174900
H	-1.77223500	2.75670000	-3.19671100
H	-1.80048500	2.20305600	-1.49625500
C	0.23701800	4.61267800	-2.91272600
H	0.50492100	5.67627700	-2.79507800
H	-0.22189500	4.49709500	-3.91648900
H	1.16204500	4.01121400	-2.89338900
C	1.28214400	5.61672000	0.19869700
C	0.57296200	6.94807900	-0.13626400
H	0.30626300	7.01410300	-1.20502700
H	1.24917800	7.79928500	0.08478300
H	-0.34801700	7.09746800	0.45148100
C	2.56659300	5.52010900	-0.65907400
H	3.11666100	4.58892700	-0.44453900
H	3.23135600	6.37615000	-0.42182100
H	2.36171400	5.54716000	-1.74015700
C	1.75372900	5.66426700	1.67048600
H	0.92656100	5.85975000	2.37354400
H	2.49443400	6.47999000	1.79710300
H	2.24034300	4.71662300	1.96349000
Cu	-1.63943600	-0.23879900	-0.15647200

7

TPSS-D3(BJ)/def2-SVP

E = -3993.050651 a.u.

Ag	-1.56572500	-0.07617900	-0.20434700
Si	0.75779300	0.53791300	-0.10976900
P	1.99921800	2.27135100	-0.05363200
Si	0.72780700	4.12071900	-0.11302500
C	-3.50464100	-0.90675100	0.20487200
C	-5.33078300	-2.27758800	0.35175200
H	-6.06124600	-2.99831600	-0.00924900
C	-5.20471900	-1.63635900	1.55014000

H	-5.79689800	-1.68440900	2.46142900
N	1.72443800	-0.83746000	0.24642800
C	4.73213500	-2.71059900	0.05156000
C	3.11113600	-0.58642100	-2.39581500
C	0.98062000	-1.97363900	-2.73788000
H	0.95674900	-2.10050600	-1.64081800
N	-4.28135500	-1.82621200	-0.45011600
N	-4.09131200	-0.80466300	1.43820800
C	-3.69170700	0.13343300	2.46625200
C	-2.52205200	-0.11643400	3.22179100
C	-2.21156200	0.80764300	4.23556300
H	-1.30809200	0.66030000	4.83233500
C	-3.03314400	1.91016100	4.48960200
H	-2.76967200	2.61145600	5.28791100
C	-4.17426900	2.13738100	3.71353600
H	-4.79013400	3.02057600	3.90466300
C	-4.52318100	1.26030400	2.67226600
C	-5.69338000	1.56404800	1.73821100
H	-6.09213500	0.60289200	1.37005500
C	-5.19252000	2.34737200	0.50433100
H	-4.84381800	3.35159900	0.79852900
H	-6.00529600	2.46320600	-0.23494700
H	-4.34806700	1.83002700	0.01987300
C	-6.85459800	2.29897300	2.42661800
H	-7.19801500	1.76037400	3.32701100
H	-7.70697800	2.38921300	1.73101300
H	-6.56967300	3.32258200	2.72762600
C	-1.64675200	-1.34490500	2.98229200
H	-1.66446600	-1.54805900	1.89517800
C	-2.23365500	-2.58219900	3.69385200
H	-2.25198900	-2.43153000	4.78825900
H	-1.61534600	-3.47173400	3.47915600
H	-3.26496700	-2.79281900	3.36045700
C	-0.17563000	-1.12318900	3.36393800
H	0.22753900	-0.21599400	2.88462800
H	0.43686200	-1.97084400	3.01904800
H	-0.03980600	-1.03770200	4.45663900
C	-4.05679200	-2.29151900	-1.79846600
C	-3.59351900	-3.61777700	-1.97671100
C	-3.21320000	-4.47902700	-0.77710300
H	-3.91304600	-4.24282600	0.04351900
C	-1.80077800	-4.09991200	-0.28686700
H	-1.04126800	-4.38278700	-1.03559700
H	-1.57077500	-4.62469200	0.65453800
H	-1.70413400	-3.01473400	-0.11027900
C	-3.31702100	-5.98908600	-1.04045000
H	-4.31451000	-6.26896900	-1.42194700
H	-3.13645400	-6.54479700	-0.10417900
H	-2.56169900	-6.32858200	-1.77140700
C	-3.41696500	-4.06848200	-3.29500200
H	-3.05787400	-5.08572000	-3.47345700
C	-3.67294100	-3.22604400	-4.38448900
H	-3.51645000	-3.59294700	-5.40421000
C	-4.11998500	-1.91948700	-4.17545600
H	-4.31467700	-1.26901800	-5.03423000
C	-4.33818500	-1.42481800	-2.87527100
C	-4.84412300	-0.00351500	-2.66691400
H	-5.07300600	0.11692100	-1.59550800
C	-6.14391900	0.26900800	-3.44746400
H	-5.98309000	0.21713400	-4.53895800
H	-6.51841300	1.28138400	-3.21443700
H	-6.92974700	-0.46025300	-3.18408100
C	-3.74346100	1.01485200	-3.01667100
H	-2.83736700	0.83082600	-2.41325700
H	-4.08354600	2.04496600	-2.81438900
H	-3.45951200	0.94847300	-4.08206000
C	2.83157300	-1.40587000	-0.07592500
N	3.45938700	-1.48420000	-1.33159800
C	4.60913100	-2.30375200	-1.24874600
C	5.40945600	-2.67979100	-2.45804300
H	5.05898900	-2.11225100	-3.33423700
H	5.30520900	-3.75520100	-2.69167100
H	6.48407900	-2.46671900	-2.32405100
C	5.79343500	-3.52948200	0.70922000
H	6.60034000	-3.74089200	-0.01000400
H	5.41561000	-4.49487500	1.09338500
H	6.22835900	-2.98966900	1.57029400
N	3.64967200	-2.15572800	0.76966000
C	4.02315900	0.17416100	2.46192200
H	3.47357700	0.33121000	1.51783600
C	3.65270000	1.36101400	3.36213000
H	4.24307800	1.37986300	4.29735200
H	2.58042900	1.34417100	3.62049400
H	3.84288200	2.30010300	2.81655300
C	5.53215800	0.17735300	2.13908000
H	5.82463800	1.15281000	1.71431500
H	5.78872900	-0.60133000	1.40106800
H	6.13142400	0.00145000	3.05115000
C	2.97970000	-2.61134500	4.90685700
H	2.81822700	-2.73392500	5.98352400

C	3.36094400	-1.36449000	4.39745800
H	3.48771900	-0.52059500	5.08111600
C	3.58719500	-1.17564800	3.02214800
C	3.40937800	-2.29675500	2.17438800
C	2.98588200	-3.55590200	2.66072300
C	2.78783100	-3.69690100	4.04569700
H	2.46411700	-4.65992900	4.45225400
C	2.63572400	-4.67750500	1.68929600
H	3.27095800	-4.56383300	0.79492300
C	1.17972900	-4.49792100	1.21623500
H	0.48204800	-4.62155300	2.06439600
H	0.92668600	-5.25007500	0.44822800
H	1.02280800	-3.49154600	0.79252300
C	2.87437800	-6.08664200	2.25288300
H	3.90823000	-6.20495500	2.62245200
H	2.69977400	-6.84297000	1.46754000
H	2.18719500	-6.31693300	3.08686100
C	1.84984100	-0.75103300	-3.02825200
C	1.45294900	0.22158400	-3.96147000
H	0.47796500	0.13737500	-4.44751500
C	2.28450100	1.30132500	-4.27772200
H	1.94920100	2.05548100	-4.99695200
C	3.53410900	1.42344800	-3.66885900
H	4.17529700	2.27582100	-3.91662800
C	3.97440100	0.49255400	-2.71009900
C	5.34343400	0.68831200	-2.06133200
H	5.49649500	-0.12793700	-1.33596400
C	6.47050500	0.61308500	-3.11432700
H	6.42421600	-0.31393600	-3.70918200
H	7.45814600	0.65988400	-2.62134000
H	6.40735900	1.46423100	-3.81605400
C	5.43773300	2.01303500	-1.28184200
H	5.34505200	2.88030900	-1.95958900
H	6.41749000	2.08296100	-0.77557000
H	4.63082500	2.08671300	-0.53457500
C	1.60475400	-3.24585700	-3.34969400
H	1.64844500	-3.17041300	-4.45140100
H	0.99862300	-4.13308400	-3.08975500
H	2.62771900	-3.41186300	-2.97465600
C	-0.47122400	-1.80964000	-3.20446400
H	-0.92626400	-0.89994400	-2.77301800
H	-1.07334400	-2.67125600	-2.87762300
H	-0.55207000	-1.75203600	-4.30490600
C	-0.56691900	4.14660600	1.37374800
C	-1.05202000	5.56978500	1.73306100
H	-1.49712700	6.09265200	0.86931300
H	-0.23723600	6.19961400	2.12813100
H	-1.83069200	5.50900000	2.52138800
C	-1.80483700	3.30592100	1.00699500
H	-2.47632700	3.22803100	1.88252300
H	-1.52245300	2.27222700	0.72321100
H	-2.38344600	3.74346600	0.17734400
C	0.03913800	3.49323900	2.63817100
H	0.93761200	4.01501300	3.00197200
H	0.32161400	2.44625700	2.43793200
H	-0.71452100	3.50235700	3.45104500
C	-0.17636500	4.31861600	-1.85319000
C	-1.23453200	5.44398300	-1.87414700
H	-2.07720300	5.23194300	-1.19360800
H	-1.65705500	5.54368800	-2.89536200
H	-0.81278100	6.42393500	-1.59433100
C	-0.85485600	2.99136000	-2.25001300
H	-0.11388800	2.17600100	-2.31651100
H	-1.33752300	3.09926900	-3.24345900
H	-1.63149800	2.68119200	-1.53357500
C	0.87736800	4.60457400	-2.94642500
H	1.32516900	5.60761600	-2.84708500
H	0.39823900	4.55405000	-3.94597600
H	1.68593200	3.85336400	-2.91923200
C	2.07846900	5.54296000	0.13203700
C	1.55318600	6.95464700	-0.21158200
H	1.30535900	7.05060000	-1.28277500
H	2.33514800	7.70948300	0.01076500
H	0.65652600	7.22774700	0.36929800
C	3.33337500	5.26989800	-0.73149300
H	3.76882000	4.28449700	-0.49646700
H	4.09886900	6.04438500	-0.51887500
H	3.12442100	5.29517100	-1.81182600
C	2.56558000	5.53817700	1.59991000
H	1.77847100	5.84475700	2.30891300
H	3.40846800	6.24997200	1.71369000
H	2.92621000	4.53632400	1.89484500

8

TPSS-D3(BJ)/def2-SVP

E = -3981.879931 a.u.

Au	-1.42307000	-0.19339000	-0.17279600
Si	0.80829600	0.55504900	-0.05570600
P	1.82636600	2.39982700	0.15992900
Si	0.36355500	4.10311600	0.11762200

N	-4.04130600	-1.86522300	-0.78575200
N	-4.02019400	-1.10810700	1.23814300
N	1.87611100	-0.75345100	0.20372900
N	3.77996800	-1.09672000	-1.27414900
N	3.87000500	-1.93575300	0.76971400
C	2.26435400	-0.36900200	-3.05957400
C	1.89088100	0.61594700	-3.98880700
H	0.98072700	0.48240700	-4.57883300
C	-3.33089300	-1.05452900	0.05647400
C	-3.66739700	-2.19274100	-2.14351500
C	-3.81336700	-1.21571200	-3.15475300
C	-4.32004700	0.18874800	-2.83645700
H	-3.84015900	0.49616300	-1.88994500
C	-5.84804800	0.18451000	-2.61258900
H	-6.37425400	-0.12973800	-3.53172300
H	-6.19674400	1.19697700	-2.34376400
H	-6.14034100	-0.50125000	-1.79973500
C	-3.93086000	1.22723200	-3.89830100
H	-2.84087800	1.24124000	-4.06701400
H	-4.23484600	2.23228800	-3.56185900
H	-4.43432300	1.03706200	-4.86331600
C	-3.49540100	-1.60510200	-4.46824800
H	-3.58895300	-0.87968000	-5.28014300
C	-3.06515400	-2.90462500	-4.75567600
H	-2.83053200	-3.18356500	-5.78819900
C	-2.91116000	-3.84179200	-3.72960100
H	-2.54605600	-4.84562200	-3.96393900
C	-3.19777600	-3.50253900	-2.39570000
C	-2.90213500	-4.46589400	-1.25085100
H	-3.61649800	-4.25854500	-0.43554800
C	-3.06374300	-5.94650000	-1.62910200
H	-4.05939900	-6.14986000	-2.06071900
H	-2.94022500	-6.57734700	-0.73189000
H	-2.30079600	-6.26772700	-2.36030600
C	-1.48996600	-4.18126500	-0.69821700
H	-0.72288200	-4.42191800	-1.45454800
H	-1.29691600	-4.79442300	0.19781700
H	-1.36614900	-3.11824300	-0.42660500
C	-5.15473500	-2.41176200	-0.14374100
H	-5.84425300	-3.07704000	-0.65878000
C	-5.13610100	-1.93845400	1.13632300
H	-5.80397100	-2.10567100	1.97844900
C	-3.69044200	-0.36108800	2.43420400
C	-2.59132200	-0.76780500	3.22392100
C	-2.35992600	-0.05081900	4.41226300
H	-1.51472400	-0.33195700	5.04643800
C	-3.18601400	1.01024700	4.79186300
H	-2.98586300	1.55032300	5.72308800
C	-4.25215600	1.40339300	3.97539300
H	-4.87117800	2.25549300	4.26913800
C	-4.52378300	0.73244100	2.77151200
C	-5.60904500	1.22517100	1.81699600
H	-6.01157000	0.35084900	1.27570200
C	-6.79251200	1.90279900	2.52557900
H	-7.21952100	1.25620800	3.31201100
H	-7.58745700	2.12881600	1.79391300
H	-6.49673900	2.85949800	2.99127900
C	-4.97908600	2.16101900	0.76359200
H	-4.56055400	3.05978200	1.24787300
H	-5.73492100	2.48200800	0.02511400
H	-4.15832600	1.65852600	0.22666600
C	-1.70218800	-1.94819800	2.84537200
H	-1.75680300	-2.06358400	1.74931100
C	-2.22968400	-3.25263200	3.47796400
H	-2.20616700	-3.19286700	4.58111600
H	-1.60347100	-4.10788200	3.16677000
H	-3.27059300	-3.45794500	3.17055500
C	-0.22406300	-1.70357900	3.18922200
H	0.12827300	-0.75138500	2.76064900
H	0.40392700	-2.50314900	2.76641200
H	-0.04778500	-1.68818600	4.27885700
C	3.05604800	-1.18429900	-0.07557000
C	4.98533000	-1.82939600	-1.16790700
C	5.04714800	-2.33842100	0.10144600
C	6.11310100	-3.12754100	0.78786400
H	6.43646500	-2.62195100	1.71600700
H	6.98860200	-3.23164200	0.12839400
H	5.77666400	-4.14050600	1.07444400
C	3.52246700	-2.24965100	2.12292900
C	3.52688000	-1.21822300	3.09287700
C	3.87264900	0.21780600	2.71150100
H	3.41963800	0.41326400	1.72511600
C	5.39901300	0.39817500	2.57546000
H	5.90809100	0.19332200	3.53497900
H	5.63263800	1.43312700	2.27203000
H	5.81646900	-0.27934000	1.81129000
C	3.27360300	1.26551100	3.66086000
H	2.18709700	1.11547100	3.78121900
H	3.42123700	2.26998000	3.23037500
H	3.74684400	1.24676200	4.66038700

C	3.21955800	-1.57750200	4.41751500
H	3.21450000	-0.80705300	5.19363600
C	2.92340000	-2.90214700	4.75975000
H	2.69804600	-3.15845000	5.80081200
C	2.89194600	-3.89632100	3.77572800
H	2.62654100	-4.92168300	4.05073500
C	3.17487700	-3.58524900	2.43404200
C	2.98958300	-4.61100400	1.32054200
H	3.67924100	-4.35121800	0.49972500
C	1.56181300	-4.48364600	0.75055200
H	0.81712100	-4.76402400	1.51715800
H	1.42975800	-5.15247400	-0.11854500
H	1.35061700	-3.44759200	0.43615200
C	3.29762800	-6.05447800	1.74662900
H	4.30739900	-6.14073600	2.18532700
H	3.24246300	-6.72700100	0.87268100
H	2.57091400	-6.42609600	2.49132000
C	5.88447900	-2.04830600	-2.33936500
H	5.41228200	-2.71607100	-3.08454100
H	6.82818500	-2.51311600	-2.01397900
H	6.12333500	-1.10707700	-2.85856000
C	3.44241500	-0.14753900	-2.29850500
C	1.45018800	-1.65242900	-2.89936000
H	1.39227600	-1.86508700	-1.81817100
C	0.00622100	-1.52053900	-3.40205400
H	-0.50234400	-0.66111800	-2.93076200
H	-0.56536200	-2.42583400	-3.14307400
H	-0.04636300	-1.40285900	-4.49956000
C	2.15865300	-2.84399600	-3.57649000
H	2.25202200	-2.68046900	-4.66552500
H	1.58265700	-3.77371600	-3.41580400
H	3.16911800	-2.99536700	-3.16265400
C	2.65801500	1.77260200	-4.16418400
H	2.33986700	2.53444400	-4.88326200
C	3.81615100	1.96452900	-3.41036200
H	4.40033700	2.88125100	-3.54208200
C	4.23438700	1.01756500	-2.45727800
C	5.49772700	1.29918700	-1.64292200
H	5.69874900	0.41988200	-1.00782400
C	6.72143100	1.51755700	-2.55917400
H	6.86068800	0.69153000	-3.27819000
H	7.64074100	1.60593400	-1.95327700
H	6.61632700	2.44973900	-3.14239400
C	5.32874800	2.50886500	-0.70343700
H	5.14872200	3.43510800	-1.27832100

H	6.24754600	2.65184000	-0.10617600
H	4.46859500	2.37107300	-0.02918700
C	1.61600000	5.63060000	0.02875800
C	0.95318200	6.98998600	0.34462600
H	0.09539900	7.21018500	-0.31166600
H	1.69129500	7.80748100	0.21251500
H	0.60070700	7.03701700	1.38977800
C	2.80439700	5.44483000	1.00482300
H	2.49380000	5.43996900	2.06085200
H	3.51789000	6.28350500	0.86920500
H	3.34375200	4.50266300	0.80702700
C	2.23604800	5.68338400	-1.38810700
H	2.68734300	4.71105900	-1.65796600
H	3.03590500	6.45128100	-1.41601400
H	1.49905400	5.94838400	-2.16456400
C	-0.68991600	4.13977500	1.78039100
C	-1.82881300	5.18411200	1.75742800
H	-1.45621100	6.20710200	1.57915500
H	-2.35437200	5.18761300	2.73474200
H	-2.58283400	4.96148100	0.98337600
C	-1.29053500	2.74634500	2.06533600
H	-1.96690300	2.38992900	1.27294500
H	-1.86832400	2.77523900	3.00919900
H	-0.49298600	1.99233700	2.17810600
C	0.23884300	4.45498400	2.97475400
H	1.10297500	3.76750100	2.99675300
H	-0.32187900	4.32728400	3.92337400
H	0.61631400	5.49134300	2.95214800
C	-0.81407700	4.09335800	-1.46629600
C	-1.32920100	5.50195400	-1.84159100
H	-1.87494600	5.98329700	-1.01163200
H	-2.03109500	5.42309400	-2.69731000
H	-0.51414400	6.17733700	-2.15007100
C	-0.07391500	3.48664600	-2.67868100
H	0.81702300	4.06508500	-2.96835300
H	-0.75769800	3.45453800	-3.55277200
H	0.26450300	2.46050900	-2.46121000
C	-2.04848000	3.20384300	-1.22135700
H	-1.76093500	2.16485400	-0.96378500
H	-2.65657100	3.15842500	-2.14563000
H	-2.69619200	3.58967100	-0.41936900

9

TPSS-D3(BJ)/def2-SVP

E = -3657.481505 a.u.

Si	0.72257100	0.55565000	-0.13530400
P	1.82771300	2.17275300	0.67011400
Si	0.53132800	4.01052900	0.71626100
N	1.54287500	-0.93862100	0.01420300
N	3.70185400	-1.28221800	-1.06357000
N	2.94903700	-2.84287500	0.30319200
C	3.12258200	0.45501700	-2.69620700
C	3.32189900	1.75880000	-3.17745100
H	2.72388400	2.12442500	-4.01574700
C	2.62659200	-1.58690500	-0.21720600
C	4.64063000	-2.34008200	-1.07161500
C	4.17677000	-3.30344400	-0.21812100
C	4.75107000	-4.61675900	0.19684000
H	4.92034800	-4.64852700	1.28912100
H	5.71504100	-4.78760300	-0.30669700
H	4.07802500	-5.45911100	-0.04826800
C	2.17950900	-3.51119600	1.30687400
C	2.31112100	-3.10731300	2.65446900
C	3.17992900	-1.92236200	3.05451400
H	3.71077500	-1.58034600	2.15120800
C	4.24720200	-2.31654100	4.09301800
H	3.78933000	-2.63534000	5.04688600
H	4.90267800	-1.45423000	4.30829800
H	4.87724200	-3.14594000	3.72544600
C	2.30540700	-0.75063000	3.54500600
H	1.60999200	-0.42806400	2.75420300
H	2.93628100	0.11483700	3.81081500
H	1.72169600	-1.03905600	4.43856100
C	1.58405300	-3.82268300	3.62491900
H	1.66318600	-3.53057700	4.67747400
C	0.76620100	-4.89763200	3.26502600
H	0.21510800	-5.44674700	4.03612200
C	0.63287300	-5.26470000	1.91884200
H	-0.02713200	-6.09460300	1.64825900
C	1.32779300	-4.57158900	0.91368600
C	1.11612100	-4.86607300	-0.56597200
H	2.05092500	-4.60225000	-1.09128500
C	0.01465500	-3.93518900	-1.12083600
H	-0.96344000	-4.18901000	-0.67149600
H	-0.06675700	-4.05275500	-2.21634300
H	0.22926900	-2.87736700	-0.88993100
C	0.80972100	-6.34079700	-0.86780000
H	1.58185900	-7.00962100	-0.44840700
H	0.76891500	-6.50294800	-1.95913800
H	-0.16817000	-6.64840300	-0.45503500

C	5.85905800	-2.30636400	-1.93339500
H	5.59359600	-2.18384600	-2.99917100
H	6.42568500	-3.24408400	-1.82403500
H	6.52689400	-1.46795200	-1.67159900
C	3.91562300	0.03074500	-1.60087000
C	2.10030100	-0.48799300	-3.33082400
H	1.60726100	-1.02980800	-2.50386900
C	0.99589000	0.25057000	-4.10121600
H	0.48821700	0.99019300	-3.45860900
H	0.24122100	-0.47386500	-4.45209900
H	1.38962200	0.77069600	-4.99295900
C	2.78684200	-1.53651800	-4.23026200
H	3.32435900	-1.04858300	-5.06339100
H	2.03636900	-2.22469600	-4.66012600
H	3.50980900	-2.14061900	-3.65861700
C	4.25883600	2.61143600	-2.58285900
H	4.38214400	3.63164400	-2.96082400
C	5.01865000	2.17450100	-1.49620400
H	5.72933100	2.85981000	-1.02291400
C	4.86481200	0.87594900	-0.97865600
C	5.67431700	0.44211000	0.24196700
H	5.51469600	-0.64038800	0.38559700
C	7.18633200	0.66964100	0.04314400
H	7.55837700	0.18907000	-0.87893500
H	7.75015300	0.25750900	0.89888700
H	7.42384100	1.74631300	-0.02287300
C	5.18684100	1.14619200	1.52387800
H	5.36077400	2.23556900	1.46525000
H	5.73378700	0.75570400	2.40181600
H	4.10479500	1.00304100	1.67153400
C	1.85653900	5.35724000	1.29978400
C	1.21899300	6.68497200	1.76618000
H	0.58005700	7.14503600	0.99448400
H	2.01479800	7.41576800	2.01755000
H	0.60694400	6.54647500	2.67429000
C	2.73724100	4.81810400	2.45433700
H	2.16538800	4.62779900	3.37527300
H	3.51851600	5.56844200	2.69429900
H	3.24091900	3.87766700	2.16979600
C	2.82497000	5.64644900	0.12818300
H	3.27456000	4.71187800	-0.25409500
H	3.64754700	6.30215000	0.47992800
H	2.33101300	6.16192200	-0.71245600
C	-0.91496500	3.82013700	2.04234800
C	-1.94531800	4.97038900	1.97900400

H	-1.47872600	5.96088500	2.11425200
H	-2.69785800	4.84478300	2.78468800
H	-2.49354800	4.98375500	1.02152800
C	-1.65383200	2.47739900	1.85643400
H	-2.10851200	2.36390600	0.86026900
H	-2.46585500	2.38799500	2.60530200
H	-0.96500900	1.62822900	2.00149900
C	-0.31027300	3.77845200	3.46407400
H	0.47721100	3.00718700	3.53767900
H	-1.10435600	3.52505400	4.19597200
H	0.11986100	4.74771400	3.76773300
C	-0.17891100	4.48185800	-1.06458100
C	-0.51853000	5.98316600	-1.20548600
H	-1.24136100	6.32297300	-0.44394300
H	-0.97292100	6.16945900	-2.20023800
H	0.37786000	6.62130000	-1.13233400
C	0.83253700	4.08758400	-2.16324700
H	1.78350700	4.63696800	-2.08734800
H	0.39622700	4.29866300	-3.16164600
H	1.07275100	3.01365900	-2.10318900
C	-1.46334900	3.68128400	-1.35084900
H	-1.29410300	2.59004100	-1.25217700
H	-1.79988200	3.86829100	-2.39119400
H	-2.29004800	3.96192300	-0.68017500
Au	-1.48912300	0.07848600	-0.87271100
C	-3.29055100	-0.85837400	-1.35851200
N	-4.20313700	-1.23645400	-0.48149300
C	-3.61238300	-1.48754200	-2.70450600
C	-4.30320600	-0.66522700	0.85381500
C	-5.21289400	-2.28092800	-0.97238200
C	-4.62391400	-2.61509900	-2.36242000
C	-2.36002500	-2.02367500	-3.41490700
C	-4.24307700	-0.35950400	-3.56146400
C	-3.62428800	-1.26847900	1.94120700
C	-5.08297400	0.50983200	1.01401800
C	-6.63640700	-1.70574800	-1.03658700
C	-5.20886900	-3.49201400	-0.03306900
H	-5.41549300	-2.71667100	-3.12292100
H	-4.08749100	-3.57773900	-2.31127700
H	-2.63848100	-2.47072300	-4.38606100
H	-1.63961900	-1.20868000	-3.59261300
H	-1.85876000	-2.79110000	-2.80494900
H	-5.16123200	0.04086400	-3.09975600
H	-3.53029500	0.47372000	-3.68008100
H	-4.49868600	-0.75386000	-4.56076700
C	-2.60692600	-2.39438000	1.78780700
C	-3.82290600	-0.71305400	3.21933300
C	-5.23978400	1.02255700	2.31261000
C	-5.64884700	1.28554900	-0.17178700
H	-7.32984600	-2.51723000	-1.31499700
H	-6.94793900	-1.31221600	-0.05499500
H	-6.73162900	-0.90685400	-1.78768000
H	-5.91116600	-4.24839300	-0.42326700
H	-4.20899500	-3.94827700	0.02758400
H	-5.53567500	-3.20980800	0.98156200
C	-2.94171500	-3.63287800	2.64160300
C	-1.20031200	-1.87204200	2.14436900
H	-2.58462300	-2.69256300	0.72594500
C	-4.63507000	0.40579000	3.41159400
H	-3.30582400	-1.15886900	4.07496600
H	-5.83021900	1.93190900	2.46015600
C	-4.71087400	2.45560400	-0.52424600
C	-7.09362900	1.76619300	0.04723100
H	-5.65000600	0.61620200	-1.04336400
H	-2.19904500	-4.42658900	2.45218600
H	-2.89656800	-3.39118200	3.71831900
H	-3.94675900	-4.03313800	2.42872000
H	-0.43483000	-2.62534500	1.90532700
H	-0.96356500	-0.96283800	1.56852900
H	-1.12709500	-1.63146200	3.21926200
H	-4.77061900	0.81964700	4.41616200
H	-5.09314700	3.01044800	-1.39957800
H	-4.61793400	3.15912200	0.32111800
H	-3.70401700	2.07854000	-0.76475200
H	-7.48123600	2.23027500	-0.87666500
H	-7.76354100	0.93337100	0.32325900
H	-7.15305400	2.52716800	0.84503200

3’’

TPSS-D3(BJ)/def2-SVP

E = -4504.068016 a.u.

Cu	-2.06636600	0.58607100	-0.04357200
Si	0.42931100	0.84229800	0.70648500
P	-0.63313000	2.20782300	-0.48609800
Si	-0.14016300	0.36220300	2.93635100
N	3.92572900	-0.58642300	-0.77850900
N	3.67072800	1.54208600	-0.29184600
N	-3.25944100	-2.14995900	-0.26441200
N	1.89483100	0.00451800	0.35318800
N	-4.61672900	-0.59343200	-0.88515600

C	3.02608300	0.30560900	-0.19254200
C	-1.03307900	-4.05843700	2.02963800
H	-1.09056100	-4.59298400	2.98416000
C	3.65136500	-1.97134400	-0.99699300
C	3.14892200	-2.36746700	-2.25503900
C	-0.86980300	-2.66677200	-0.43063300
C	5.09107100	0.08261500	-1.21467700
C	-2.08876400	-2.79695300	0.26857400
C	0.19707900	-3.91404500	1.37522200
H	1.10500900	-4.34076700	1.81384900
C	2.81055800	-1.32655000	-3.29548100
H	2.07591300	-0.60562000	-2.89414400
C	4.93401000	1.40563600	-0.90359400
C	-2.20179800	-3.49277900	1.49278900
C	3.68537200	-4.25513500	-0.21977800
H	3.87761900	-4.98947800	0.57026300
C	3.09934000	2.76431900	0.18553000
C	-4.37645400	-2.78917200	-0.80067700
H	-4.44706400	-3.87270600	-0.86613000
C	0.27694000	-3.22203900	0.16342700
H	1.23790400	-3.10451900	-0.33873100
C	-5.23668100	-1.80278400	-1.19557000
H	-6.21227300	-1.84736400	-1.67510400
C	2.94143300	-3.74109900	-2.47704400
H	2.55001800	-4.07430300	-3.44453100
C	3.90193200	-2.89040000	0.04339700
C	-5.15676300	0.71975200	-1.13075100
C	4.33910200	-2.39271900	1.39804500
H	5.22318900	-1.73566700	1.32055100
C	3.10740500	3.00596400	1.57753500
C	3.21909500	-4.67732800	-1.47188100
H	3.05256600	-5.74319800	-1.65980600
C	-3.38600200	-0.78265100	-0.30680000
C	3.73002700	2.00389600	2.51935300
H	3.15651200	1.05903800	2.50973800
C	2.51686200	3.65647000	-0.73877400
C	5.85405900	2.57186300	-1.05144500
H	5.40444800	3.37144900	-1.66751500
H	6.79635900	2.25780500	-1.52676800
H	6.09196500	3.01941600	-0.06877800
C	6.23078400	-0.66271000	-1.82587500
H	6.62848200	-1.42963400	-1.13529200
H	7.04752100	0.03114800	-2.07840100
H	5.92966300	-1.18992000	-2.74999000
C	-4.63006500	1.47868000	-2.19716000
C	-6.15700300	1.20364600	-0.26181800
C	-0.77226800	-1.95814300	-1.75834800
H	-1.67411900	-2.12025100	-2.37255200
C	-6.62582900	0.37781100	0.91323000
H	-7.14170100	-0.54495700	0.59131800
C	2.50417600	4.18666900	2.04195800
H	2.48780900	4.39280100	3.11746800
C	-3.52465200	-3.59870800	2.21712600
H	-4.19723000	-4.33468000	1.73872800
C	1.93898600	4.83280800	-0.23074600
H	1.46214900	5.53270800	-0.92488700
C	-6.65941400	2.49360100	-0.50164900
H	-7.43213100	2.89717600	0.16169200
C	-5.16016400	2.76673700	-2.39382800
H	-4.76618300	3.38235700	-3.20929000
C	1.92914400	5.09373800	1.14371200
H	1.45662900	6.00679000	1.52082000
C	-6.16686800	3.26693200	-1.56072400
H	-6.56353700	4.27352800	-1.72995900
C	2.40031200	3.29998000	-2.19880400
H	1.50817300	2.65048500	-2.31507500
C	-3.50752700	0.95457300	-3.05732000
H	-3.65981300	-0.10369700	-3.33050400
C	-1.95347300	-0.18996800	3.06843200
C	0.96958300	-0.97387600	3.71322100
C	0.02797200	1.98541100	3.91627500
H	-0.66860100	-0.86314300	-1.60943400
H	0.10995500	-2.31519500	-2.31191200
H	-4.05426100	-2.63045300	2.22409700
H	-3.36841200	-3.91706800	3.26012500
H	-7.32151100	0.95539700	1.54215900
H	-5.76715900	0.06697800	1.53553100
H	-3.40813900	1.55186200	-3.97748600
H	-2.54755700	1.01886100	-2.50195300
H	2.38664100	-1.79507900	-4.19798500
H	3.69962500	-0.74182100	-3.59283400
H	4.57819900	-3.23051600	2.07242900
H	3.52806200	-1.79245700	1.84888000
H	3.74938100	2.38993900	3.55085600
H	4.76296700	1.75563400	2.21622300
H	2.25026000	4.20098500	-2.81571100
H	3.27779700	2.74801800	-2.57416100
H	2.02687200	-0.65295200	3.72673000
H	0.90748300	-1.90526400	3.12407200
H	0.66880500	-1.19868800	4.75458400

H	1.08137800	2.30743800	3.99022400
H	-0.53290200	2.78768600	3.40396800
H	-0.37168200	1.87350500	4.94264200
H	-2.27940000	-0.22449700	4.12575300
H	-2.08496300	-1.19185700	2.62756600
H	-2.61252700	0.50999800	2.51987700

TS$_{iso}$-3’

TPSS-D3BJ/def2-SVP

E = -4504.031097 a.u.

P	0.22752900	0.20487700	0.82188700
Si	-1.95358100	-0.45213100	3.82898900
Si	-1.66562300	-0.00149400	1.56214600
N	-3.22167500	-1.36956800	-1.21683000
N	-3.15074000	0.10253600	0.72413600
N	-4.42435900	0.47257400	-1.26156400
C	-0.98223100	-2.35858200	-1.40334000
C	-1.62123800	-4.09485100	0.73810000
C	-0.57101400	-1.34747100	-2.44289400
C	-3.90978600	-1.35097400	-2.44959200
C	-2.59782900	-3.19675600	0.27771000
C	-3.73869400	-0.10352400	4.38209900
C	-4.23360000	4.16919300	-0.95763100
C	-1.54426500	-2.28692000	4.12956900
C	-2.25517700	-2.33742600	-0.79278100
C	-0.35465100	-4.13906800	0.14258400
C	-6.92666600	0.92933400	0.05942800
C	-2.50641100	2.50633300	-1.82686000
C	-3.53460300	-0.23713200	-0.46477300
C	-3.96241400	-3.12109400	0.91758900
C	-3.82831000	-2.49549700	-3.40499400
C	-3.86104000	2.84582200	-1.25412700
C	-0.04034700	-3.28214000	-0.91581400
C	-6.02870500	2.07654100	-0.33735400
C	-5.55086700	0.37173600	-3.52542100
C	-4.64627100	-0.19606700	-2.48309400
C	-6.36338300	3.41352900	-0.05993100
C	-5.47579100	4.45075600	-0.37472600
C	-0.71508800	0.61065000	4.79701900
C	-4.78869500	1.82459200	-0.95621800
H	-1.42447300	-0.92235200	-2.99366600
H	-7.86204200	1.29767400	0.51070100
H	-1.87550600	2.00162300	-1.06841500
H	-4.06771800	-2.17491300	1.48082300
H	-4.46164000	-2.30242900	-4.28491100
H	-4.17027800	-3.43413800	-2.93071200
H	-2.79442300	-2.66864400	-3.75352800
H	-5.58009900	-0.28726400	-4.40701100
H	-5.20597900	1.37040100	-3.85126500
H	-6.58379000	0.49470600	-3.15056400
H	-3.53500800	4.98304900	-1.18140700
H	-5.74992900	5.48768200	-0.15178600
H	-7.32325400	3.63736000	0.41878600
H	0.96432100	-3.29760500	-1.35044200
H	0.39869600	-4.84036800	0.51568100
H	-1.86169700	-4.76385900	1.57146000
C	4.04298700	0.39409600	-0.25217000
N	4.93007700	1.43848500	-0.14571300
N	4.85284300	-0.71253700	-0.34179700
C	6.25183600	0.99375900	-0.16884800
C	4.50416900	2.80801100	-0.01265300
C	6.20326300	-0.36642200	-0.29301500
C	4.33788700	-2.05455400	-0.44258400
H	7.09333500	1.67697000	-0.07641200
C	3.89820200	3.21888800	1.19532200
C	4.68171000	3.67243000	-1.11468000
H	6.99294500	-1.11374800	-0.33169200
C	3.78707200	-2.66354700	0.70609800
C	4.39660800	-2.69747200	-1.69809400
C	3.67219400	2.26320600	2.33996100
C	3.47380600	4.55801500	1.27911100
C	4.24827200	5.00189200	-0.97895900
C	5.28025600	3.17104200	-2.40866500
C	3.31840000	-3.98351000	0.57185200
C	3.65134900	-1.92285700	2.01163100
C	3.91204700	-4.01363400	-1.78277800
C	4.92586900	-1.97195500	-2.91304200
H	4.53494600	1.59174000	2.48858300
H	2.99574700	4.90304700	2.20199000
C	3.65035200	5.44145800	0.20894700
H	4.36852100	5.69068900	-1.82226800
H	4.80243700	2.22602700	-2.72204500
H	2.88871400	-4.48064900	1.44769800
C	3.38663800	-4.65525800	-0.65331400
H	4.53587900	-1.29910900	2.22474300
H	3.94144100	-4.53260200	-2.74698600
H	4.41587000	-0.99986700	-3.03708700
H	3.31136800	6.47899300	0.29712800
H	3.01261600	-5.68125800	-0.73447800
H	2.77204200	-1.24540700	1.96564300

H	3.49921000	-2.62628800	2.84581500
H	6.00726900	-1.75994300	-2.82921400
H	4.76650200	-2.57032900	-3.82395500
H	2.78995000	1.61977100	2.13406200
H	3.48195300	2.81378300	3.27502800
H	6.36284600	2.96894900	-2.31291600
H	5.14671000	3.91406400	-3.21083800
H	-2.28215000	-2.94628700	3.63877700
H	-0.55008900	-2.52497700	3.71056300
H	-1.53689700	-2.52191000	5.21137000
H	0.28489100	0.50020700	4.33904300
H	-0.98798600	1.68030900	4.74760700
H	-0.65864200	0.31253300	5.86116800
H	-4.45026500	-0.71604300	3.79887500
H	-4.00007500	0.95674100	4.21332000
H	-3.88665500	-0.33087700	5.45517500
H	-4.12012000	-3.96407400	1.60937700
H	-4.76345800	-3.13132300	0.15736800
H	-0.06231300	-0.51583500	-1.91225000
H	0.14120200	-1.78716000	-3.16187300
H	-7.18450400	0.28980700	-0.80332600
H	-6.41129600	0.28529500	0.79495300
H	-1.98021500	3.41564000	-2.15912100
H	-2.59030300	1.81494800	-2.68422500
Cu	2.18238700	0.40403400	0.00458100

E-**PMP-3'**

TPSS-D3(BJ)/def2-SVP

E = -4504.065299 a.u.

P	-0.08943400	1.56355400	2.40374700
Si	3.37314600	0.17205800	3.03438700
Si	1.56271400	0.57203600	1.58947700
N	1.88802300	1.63855700	-1.65802300
N	1.84910600	-0.11983100	0.03341700
N	1.99193300	-0.45253300	-2.33496400
C	0.40374600	3.31765600	-0.68524800
C	2.58622100	4.32476600	0.81079100
C	-0.76827600	2.67977400	-1.37757200
C	2.00110100	1.66352500	-3.06247300
C	2.80512600	3.25894500	-0.07773700
C	4.86731700	-0.70688400	2.24698100
C	0.58309400	-3.83416600	-2.91871600
C	3.91369400	1.85945800	3.72865300
C	1.69843300	2.77355600	-0.80883500
C	1.30867400	4.87789200	0.95645600
C	4.23688400	-2.00814100	-1.47025700
C	-0.54678100	-1.54844300	-3.01436900
C	1.88819200	0.32285700	-1.17848300
C	4.16272900	2.61812600	-0.24196600
C	2.10018000	2.95457700	-3.80500100
C	0.65357300	-2.43674800	-2.78603700
C	0.22946700	4.38023800	0.21699400
C	2.98159300	-2.66612900	-1.98251100
C	2.28854900	-0.21915100	-4.84248500
C	2.06453400	0.36448400	-3.48656400
C	2.85513700	-4.06259300	-2.08729000
C	1.67520300	-4.64075700	-2.57305900
C	2.73667300	-0.89233500	4.47781300
C	1.87608500	-1.87612000	-2.35888800
H	-0.54437300	2.36961500	-2.41171700
H	5.02775900	-2.75034700	-1.27697300
H	-0.84372500	-1.07195600	-2.06011400
H	4.13846400	1.56684600	0.09845200
H	2.19019900	2.76964500	-4.88671800
H	2.97956000	3.53956700	-3.47720900
H	1.21184700	3.58935600	-3.63356400
H	2.35043700	0.58238400	-5.59498700
H	1.47700100	-0.90844400	-5.13937200
H	3.22904000	-0.80028100	-4.87652200
H	-0.34294800	-4.29078100	-3.28319900
H	1.60307000	-5.72912300	-2.67553300
H	3.69985900	-4.69796600	-1.79889500
H	-0.77303100	4.79707000	0.35908700
H	1.14830900	5.69355100	1.66909000
H	3.42350700	4.71007900	1.40241700
C	-2.73274700	-0.74245000	0.48896600
N	-2.98081600	-2.03128200	0.90118200
N	-3.97046400	-0.28603400	0.10458400
C	-4.32888400	-2.36102100	0.77951200
C	-1.92702600	-2.88731700	1.39055600
C	-4.95757600	-1.25598200	0.27505400
C	-4.18277400	1.06454900	-0.35223500
H	-4.71587800	-3.33442600	1.07322600
C	-1.37576000	-2.62744200	2.66304500
C	-1.45640600	-3.91384600	0.54417700
H	-6.00258700	-1.06734000	0.03778400
C	-4.25442000	2.09600700	0.60838700
C	-4.27881700	1.29774300	-1.73964500
C	-1.92466600	-1.56093800	3.57372100
C	-0.25919300	-3.39516900	3.04625200

C	-0.34283400	-4.65450700	0.97116600
C	-2.13384400	-4.20201700	-0.77361500
C	-4.43675800	3.40755800	0.13279200
C	-4.09863600	1.80427000	2.07888700
C	-4.46946800	2.62297500	-2.16704800
C	-4.14393300	0.15986400	-2.72309000
H	-3.01297700	-1.43413000	3.44924800
H	0.20261400	-3.20241200	4.02002100
C	0.26150500	-4.38431700	2.20590400
H	0.06021500	-5.43532900	0.31791100
H	-2.22403100	-3.28859300	-1.38507000
H	-4.48997000	4.22945600	0.85462400
C	-4.54872400	3.66865400	-1.23815600
H	-4.72755700	0.95324500	2.39352800
H	-4.54303600	2.83266000	-3.23968500
H	-3.20585000	-0.39442300	-2.54794600
H	1.14139900	-4.95660600	2.51818200
H	-4.69208400	4.69687000	-1.58684100
H	-3.04419300	1.53439500	2.29589600
H	-4.36074600	2.68574900	2.68453400
H	-4.97025200	-0.56720000	-2.62393400
H	-4.14128500	0.53566100	-3.75845600
H	-1.44271200	-0.58552700	3.34556000
H	-1.70986400	-1.80499600	4.62687100
H	-3.15683900	-4.59310000	-0.62440700
H	-1.56582100	-4.95150600	-1.34342900
H	4.39026800	2.47934800	2.94792500
H	3.02965300	2.40893600	4.09962100
H	4.63148200	1.74336700	4.56314300
H	1.81763800	-0.44077300	4.89302400
H	2.48605900	-1.91152300	4.13104500
H	3.48830100	-0.97593400	5.28610900
H	5.28060800	-0.13380400	1.39733800
H	4.57742700	-1.70429000	1.86988800
H	5.67841500	-0.84492900	2.98765200
H	4.92569400	3.15408000	0.34440400
H	4.47666300	2.60563600	-1.30115800
H	-1.05049800	1.75885800	-0.80920700
H	-1.64969400	3.33854800	-1.37522100
H	4.61868000	-1.26290400	-2.19106300
H	4.01175100	-1.46716200	-0.53417300
H	-1.40045700	-2.13026500	-3.39767100
H	-0.33873400	-0.72900200	-3.72429300
Cu	-1.22803100	0.34246200	0.93476300

TS1-3'

TPSS-D3(BJ)/def2-SVP

E = -4504.028768 a.u.

P	0.28487000	-0.08382800	2.59560900
Si	-1.96686200	1.38544200	3.30272100
Si	-1.73318200	-0.83710000	2.33044900
N	-2.10503800	-2.55051000	-0.90433500
N	-2.40175600	-0.81039300	0.75294800
N	-3.04052900	-0.64956900	-1.53903800
C	0.07650500	-3.35583300	-0.15844800
C	-1.21316100	-5.08389700	1.66549100
C	0.74755200	-2.34432600	-1.04685200
C	-2.33765900	-2.63883900	-2.29509600
C	-1.99762500	-4.27465800	0.82568400
C	-3.72233500	1.76198800	2.63832400
C	-2.74146100	3.04076500	-1.21462400
C	-1.97751800	1.24150000	5.19251700
C	-1.33031300	-3.43396800	-0.09066200
C	0.18549000	-5.04222500	1.59662900
C	-5.83678300	0.01333600	-1.41571900
C	-0.93165300	1.26473100	-1.31477200
C	-2.50104100	-1.29755000	-0.42123600
C	-3.50366400	-4.25190500	0.91653500
C	-2.01710900	-3.88450200	-3.05219400
C	-2.37504700	1.68988900	-1.34974700
C	0.82620500	-4.18285400	0.69646600
C	-4.77072600	1.08291800	-1.41430100
C	-3.35184500	-0.98030000	-4.03253900
C	-2.91281600	-1.45923500	-2.68899300
C	-5.09611900	2.44574500	-1.29290800
C	-4.09046900	3.41599400	-1.19569900
C	-0.98834900	2.95555700	2.81590900
C	-3.40584500	0.73159300	-1.46618900
H	0.23112900	-2.21015700	-2.01142300
H	-6.83591000	0.45564800	-1.27366200
H	-0.69611300	0.80183600	-0.32968500
H	-3.84308400	-3.27651000	1.31169100
H	-2.25754900	-3.75927900	-4.11957700
H	-2.59219100	-4.74784100	-2.66752800
H	-0.94688700	-4.14928700	-2.96622900
H	-3.10884200	-1.72802900	-4.80357100
H	-2.84921700	-0.03193900	-4.29897100
H	-4.44021600	-0.78835600	-4.07237500
H	-1.95491700	3.79572700	-1.11262800
H	-4.36148500	4.47168200	-1.08613900

H	-6.15021100	2.74117600	-1.24704200
H	1.91905600	-4.12505600	0.66695000
H	0.78158500	-5.67356500	2.26440600
H	-1.70818700	-5.74206700	2.38789600
C	2.82085000	0.95943900	-0.41030200
N	3.09806000	2.23801500	-0.83662700
N	3.98678500	0.27999900	-0.68261500
C	4.38934300	2.34909000	-1.35049000
C	2.17542600	3.33921200	-0.73145200
C	4.95195200	1.10963500	-1.25334100
C	4.20419500	-1.11347300	-0.38798100
H	4.78565000	3.29449100	-1.71475000
C	1.92162500	3.88835400	0.54336400
C	1.57095000	3.82198400	-1.91221400
H	5.94332000	0.74548100	-1.51420500
C	4.36935300	-1.50906200	0.95742700
C	4.26208500	-2.02091600	-1.46798000
C	2.58058000	3.33281400	1.78025900
C	1.01219500	4.95945300	0.61425600
C	0.68025700	4.90252700	-1.79235200
C	1.84163600	3.17111700	-3.24884000
C	4.58702000	-2.87757900	1.20345300
C	4.29414900	-0.51531000	2.08733900
C	4.49803700	-3.37462800	-1.17259200
C	4.03183700	-1.55710700	-2.88780900
H	3.66580900	3.19459600	1.63117400
H	0.78243500	5.39393800	1.59190300
C	0.40216800	5.46448500	-0.53945500
H	0.18783000	5.28961000	-2.69082700
H	1.76190100	2.07240200	-3.17389700
H	4.70779500	-3.21478300	2.23817700
C	4.65748600	-3.79947700	0.15268400
H	4.87263600	0.39759800	1.86258500
H	4.53728900	-4.10071600	-1.99166100
H	3.13920100	-0.91001700	-2.94927600
H	-0.30259300	6.29875400	-0.46092200
H	4.83191000	-4.85884000	0.36769000
H	3.24066300	-0.20078700	2.25085000
H	4.66994900	-0.95688000	3.02335800
H	4.88446800	-0.96904500	-3.27348800
H	3.88662400	-2.41906900	-3.55802000
H	2.15882900	2.33551700	2.02714200
H	2.41874800	3.99717800	2.64257500
H	2.85811400	3.39787100	-3.61924000
H	1.12092800	3.52317300	-4.00366900
H	-2.68701000	0.46328600	5.52382200
H	-0.97268800	0.97055400	5.56035700
H	-2.27161000	2.20576100	5.65123600
H	0.00424000	2.97444700	3.29791400
H	-0.84041600	2.99625100	1.72234300
H	-1.54731300	3.86407200	3.11836900
H	-4.44912100	1.01020000	2.99338600
H	-3.71920700	1.73413200	1.53420800
H	-4.05094600	2.76910200	2.96503100
H	-3.87206000	-5.04544000	1.58625600
H	-3.96903700	-4.37925100	-0.07675300
H	0.74548100	-1.35077800	-0.53868400
H	1.79755300	-2.61470600	-1.22550900
H	-5.84765400	-0.56248400	-2.35838400
H	-5.65267600	-0.70892900	-0.60074600
H	-0.26636800	2.12860600	-1.44971600
H	-0.70619900	0.50714400	-2.08466200
Cu	1.37308100	0.38119100	0.73219900

INT1-3’

TPSS-D3(BJ)/def2-SVP

E = -4504.064858 a.u.

P	-0.46384600	-0.41489500	2.40848500
Si	-1.33302400	-2.23138300	3.37844100
Si	1.73673200	-0.91493100	2.48996100
N	3.42588400	1.30574200	-0.52077100
N	2.32207500	0.09735900	1.23505400
N	3.34495900	-0.89620300	-0.71383500
C	1.78296900	3.06381200	-0.10365100
C	3.82326000	4.68850300	0.98256000
C	0.69859200	2.17147100	-0.64608800
C	3.99155400	0.96908100	-1.77328000
C	4.15213900	3.40257400	0.51821500
C	-1.20252500	-3.77912900	2.27647100
C	1.28854400	-3.98554000	-0.51718700
C	-0.41809800	-2.60121500	5.00067800
C	3.12200900	2.61721300	-0.04263800
C	2.50527600	5.15824700	0.91675600
C	5.20737000	-2.57008100	0.68075200
C	0.69567100	-1.69122400	-1.42204500
C	2.97106600	0.15581000	0.13837800
C	5.54810700	2.84475100	0.66759600
C	4.45422100	2.01781700	-2.72924400
C	1.66471900	-2.66296100	-0.81034600
C	1.49246900	4.34794800	0.38917400

C	3.87095600	-3.09774400	0.21941800
C	4.36591700	-1.30992900	-2.98929900
C	3.93831500	-0.39349800	-1.89189500
C	3.45952200	-4.41427100	0.48976600
C	2.18120600	-4.85434600	0.12179400
C	-3.16471800	-1.92749300	3.80990000
C	2.96480800	-2.24727000	-0.44907000
H	1.00258200	1.68083100	-1.58683000
H	5.83534600	-3.38090600	1.08357800
H	0.27587200	-1.03706900	-0.62237900
H	5.51612300	1.85171300	1.14896100
H	4.78703900	1.55385900	-3.67092600
H	5.29438800	2.61329600	-2.32686600
H	3.64058100	2.72985300	-2.96230000
H	4.79820900	-0.73668900	-3.82431000
H	3.51366100	-1.89953100	-3.37613300
H	5.12401100	-2.03616400	-2.64073000
H	0.28148900	-4.32311100	-0.78274800
H	1.87310300	-5.88058500	0.34917500
H	4.14420600	-5.09207400	1.01127200
H	0.45700200	4.70276100	0.36344800
H	2.26334000	6.15741400	1.29539200
H	4.60770800	5.31395700	1.42341100
C	-2.68767000	0.52267100	-0.89672600
N	-3.30463200	-0.25211100	-1.84995200
N	-3.39599600	1.70137600	-0.94308600
C	-4.36451200	0.41820800	-2.46044100
C	-2.91627900	-1.60419200	-2.15909300
C	-4.42112000	1.65547000	-1.88605100
C	-3.10033200	2.84198600	-0.11240400
H	-4.98047200	-0.05127300	-3.22447000
C	-3.21621200	-2.61972200	-1.22714000
C	-2.25078400	-1.85126400	-3.37781200
H	-5.09691900	2.49280000	-2.04522500
C	-3.36019000	2.76171000	1.27360900
C	-2.56054700	3.99310200	-0.72736900
C	-3.91296100	-2.29718400	0.06927300
C	-2.81667200	-3.92998200	-1.54657400
C	-1.89126800	-3.18004100	-3.66489000
C	-1.88150400	-0.71724700	-4.30473800
C	-3.02701800	3.88189000	2.05654800
C	-3.94246800	1.52288300	1.90378000
C	-2.26422800	5.09317800	0.09583300
C	-2.28908700	4.04176300	-2.21413100
H	-4.83424200	-1.71306500	-0.10264000
H	-3.02424900	-4.73574100	-0.83505600
C	-2.16849400	-4.20921900	-2.75627400
H	-1.36452100	-3.39955800	-4.59964400
H	-1.35209200	0.07817300	-3.75023500
H	-3.20037700	3.84048200	3.13674100
C	-2.48686100	5.03489600	1.47715500
H	-4.74075400	1.08364600	1.28144700
H	-1.83876900	5.99606200	-0.35550900
H	-1.87692700	3.08569800	-2.57908500
H	-1.86797100	-5.23602400	-2.98878100
H	-2.23496500	5.89445000	2.10675800
H	-3.15103400	0.75199400	2.01975100
H	-4.34700200	1.74561000	2.90346600
H	-3.21109700	4.24046500	-2.79151300
H	-1.57011000	4.84304500	-2.44790700
H	-3.25253700	-1.67587900	0.70750300
H	-4.16710000	-3.21318800	0.62302600
H	-2.77067100	-0.25222600	-4.76734300
H	-1.22570300	-1.07502600	-5.11389100
H	0.65896500	-2.76303200	4.81242400
H	-0.51078800	-1.75478600	5.70482900
H	-0.82704500	-3.50711300	5.48861800
H	-3.25367800	-1.06954600	4.50124400
H	-3.77527200	-1.70062200	2.91822800
H	-3.60615300	-2.81391700	4.30554500
H	-0.13724500	-4.05023300	2.16082400
H	-1.59719000	-3.58576400	1.26365700
H	-1.74053900	-4.64728100	2.70561100
H	6.17276600	3.51430800	1.28060800
H	6.05059100	2.71093500	-0.30753600
H	0.48191500	1.36098300	0.08400400
H	-0.22736700	2.73863200	-0.81434600
H	5.75580000	-2.06948500	-0.13617700
H	5.05795200	-1.81874800	1.47835000
H	-0.14512000	-2.21762500	-1.89312300
H	1.17648100	-1.03072600	-2.16299200
Cu	-1.42387300	0.02745000	0.46684200

INT2-3’

TPSS-D3(BJ)/def2-SVP

E = -4504.067098 a.u.

P	-0.15259000	-0.01208800	2.34812000
Si	-0.87698400	-1.67034200	3.68109200
Si	0.90716300	-1.29664600	0.73883500
N	2.00849700	2.29156000	-0.20433100

N	1.90238000	-0.14529300	-0.07492300	C	-3.44994800	-2.24532600	-2.58206600
N	3.84318200	1.13475800	-0.59587000	C	-1.27822600	-2.92232000	-1.55122600
C	-0.40609900	2.50054300	-0.52080900	C	-4.30835900	-1.19525600	-2.43421300
C	-0.64232800	3.50526700	2.10586500	C	-4.30817900	0.91936600	-1.11232500
C	-0.24974200	1.94774700	-1.91484300	H	-3.50328700	-3.14334600	-3.19375200
C	3.02842600	3.21284700	-0.52245200	C	-1.39570900	-3.88506300	-0.53039500
C	0.62727400	3.12693800	1.63448900	C	-0.15827100	-2.82620300	-2.39835300
C	-0.24217000	-3.41245000	3.24800000	H	-5.27335800	-0.98365600	-2.88858000
C	5.65545800	-1.89276100	-1.77420200	C	-4.66484400	1.12817200	0.23888600
C	-0.21165000	-1.27583200	5.42333200	C	-4.48838200	1.90763700	-2.10780000
C	0.72287000	2.65085900	0.30972700	C	-2.59182000	-3.87971700	0.38780700
C	-1.77369000	3.38295900	1.29542500	C	-0.33107200	-4.78533200	-0.36575200
C	5.49251400	0.69059900	1.70054200	C	0.88662900	-3.74217100	-2.19120300
C	3.73004700	-0.61856600	-2.85254600	C	-0.06675700	-1.74648900	-3.44762600
C	2.49187500	0.97580700	-0.25457800	C	-5.23129000	2.37083500	0.57577900
C	1.82268500	3.11349300	2.55301000	C	-4.41390900	0.09205400	1.30112400
C	2.74691400	4.67512900	-0.63135100	C	-5.06012400	3.13238300	-1.71953400
C	4.74080300	-0.82582300	-1.75360000	C	-4.05004700	1.69340800	-3.53949700
C	-1.66023000	2.86828300	-0.00282000	H	-2.59899900	-2.94290700	0.97577900
C	5.61106100	-0.17423700	0.46743600	H	-0.38454900	-5.53502000	0.43011300
C	5.52291000	2.91967600	-1.22609500	C	0.80023400	-4.71149600	-1.18607200
C	4.17013700	2.49316000	-0.75892300	H	1.78243700	-3.67825800	-2.81774900
C	6.51876400	-1.24597800	0.40256800	H	0.05464800	-0.75981300	-2.96710600
C	6.54642000	-2.09348300	-0.71222300	H	-5.51406100	2.55443600	1.61770000
C	-2.77562100	-1.77289300	3.85767500	C	-5.43150300	3.36296900	-0.38991300
C	4.75571000	0.03444300	-0.63599300	H	-4.63749400	-0.92747500	0.94615200
H	0.51233600	2.50503500	-2.48811000	H	-5.19565100	3.91728200	-2.47134200
H	6.18992400	0.35343300	2.48389500	H	-3.13525800	1.08103400	-3.59618900
H	2.71930200	-0.80567400	-2.44643900	H	1.63036900	-5.40777100	-1.03034200
H	2.75277000	3.42829500	2.05057000	H	-5.86936200	4.32471200	-0.10366600
H	3.65824400	5.21968200	-0.92407700	H	-3.34324000	0.09716200	1.60694900
H	2.38488400	5.09479000	0.32489700	H	-5.01390300	0.29779800	2.20123200
H	1.96341200	4.87596000	-1.38562700	H	-4.82600600	1.17667400	-4.13397400
H	5.56972000	4.01635400	-1.31266400	H	-3.85405200	2.66237700	-4.02615500
H	5.76120000	2.48208600	-2.21352700	H	-2.55704800	-4.72559900	1.09086300
H	6.31942500	2.59492000	-0.53232000	H	-3.54315400	-3.92349100	-0.17224000
H	5.66305400	-2.57304600	-2.63331300	H	-0.97718300	-1.69662900	-4.07081600
H	7.25614400	-2.92714000	-0.74556500	H	0.79937500	-1.91302000	-4.10661500
H	7.19336800	-1.42685800	1.24683700	H	0.89238400	-1.21510700	5.41112500
H	-2.54591300	2.78224700	-0.63549700	H	-0.59424100	-0.30192600	5.78060900
H	-2.75813100	3.67028900	1.67784100	H	-0.50523300	-2.05329700	6.15552800
H	-0.73942000	3.86582100	3.13530300	H	-3.20159200	-0.78451100	4.10934400
C	-2.52087300	-0.82405400	-1.04000800	H	-3.26070900	-2.12326600	2.92906900
N	-2.37357900	-1.99832900	-1.73416500	H	-3.05079400	-2.47760200	4.66646800
N	-3.72639700	-0.33730300	-1.50008900	H	0.85563000	-3.46001100	3.36736400

H	-0.45773500	-3.68465500	2.20185100
H	-0.69675600	-4.16921600	3.91702700
H	1.96576600	2.07531600	2.91113800
H	1.65396500	3.75653100	3.43183500
H	0.08561000	0.89656900	-1.86008700
H	-1.20442500	1.98269000	-2.46219200
H	5.70064300	1.75448900	1.48803100
H	4.46449000	0.64234000	2.10280100
H	3.91455800	-1.30056700	-3.69860800
H	3.74427700	0.42112700	-3.22500800
Cu	-1.41096400	-0.28493000	0.46464200

TS2-3’

TPSS-D3(BJ)/def2-SVP

E = -4504.052762 a.u.

P	0.31186900	-0.22344400	2.52133500
Si	-1.24147600	-1.77685400	3.01468600
Si	0.67348100	-0.76485900	0.45062500
N	3.22043600	1.69879400	0.06467300
N	2.22643500	-0.49389700	-0.18996700
N	4.52301500	0.05122600	-0.59909500
C	1.00210200	2.65454600	-0.29205000
C	0.85797300	3.28978600	2.45651800
C	1.08584900	2.29817600	-1.75718600
C	4.50533000	2.24304800	-0.14293600
C	2.01625200	2.66169600	1.96660900
C	-1.29447600	-3.29330100	1.87148600
C	5.21334200	-3.12909800	-2.39768100
C	-0.82135400	-2.40706100	4.76193000
C	2.07264100	2.37276500	0.58616900
C	-0.21183700	3.58256400	1.60601000
C	5.78846700	-1.38310900	1.52745600
C	3.92606900	-1.06315800	-3.16009700
C	3.21112500	0.33183400	-0.22008000
C	3.09321200	2.19227000	2.91307900
C	4.76117000	3.70606600	0.00706500
C	4.73154200	-1.83319100	-2.14329200
C	-0.14626500	3.25653300	0.24671500
C	5.65513000	-1.98146700	0.14690200
C	6.74379500	1.20263000	-0.98893100
C	5.31661800	1.21805900	-0.55149700
C	6.12880600	-3.27133200	-0.15056700
C	5.91650900	-3.83695300	-1.41447200
C	-3.00957900	-1.06667800	3.10429800
C	4.98730300	-1.27311100	-0.87472100
H	2.05416500	2.60362900	-2.19137200
H	6.32842600	-2.06697200	2.20164600
H	2.89014500	-0.94164000	-2.79287700
H	4.09218200	2.16882700	2.44854900
H	5.81287100	3.93641800	-0.22410100
H	4.54805200	4.05595300	1.03341100
H	4.11797600	4.29497800	-0.67312900
H	7.18332200	2.20762300	-0.89310600
H	6.83716100	0.88150200	-2.04323200
H	7.35116100	0.50245700	-0.38665000
H	5.02979900	-3.58503500	-3.37703800
H	6.28919400	-4.84423300	-1.62981200
H	6.65221500	-3.84105000	0.62533700
H	-0.98662400	3.48037700	-0.41356400
H	-1.11476400	4.05350300	2.00672600
H	0.78629800	3.51041800	3.52676700
C	-3.21781500	-0.03366100	-0.73487400
N	-3.99860200	-1.08538600	-1.16477800
N	-3.94206400	1.07039300	-1.12344700
C	-5.16442300	-0.64943200	-1.79031500
C	-3.63283400	-2.47344300	-1.03201800
C	-5.13012400	0.71349600	-1.76538200
C	-3.55769900	2.44803600	-0.95293900
H	-5.89686000	-1.34527700	-2.19340400
C	-4.35222000	-3.27318600	-0.11844500
C	-2.59851900	-2.98231600	-1.84909800
H	-5.83128800	1.46128000	-2.12965100
C	-3.67615100	3.04684900	0.31864600
C	-3.13968100	3.16075600	-2.09812500
C	-5.45326700	-2.69141100	0.73668500
C	-4.00096600	-4.63061000	-0.02460100
C	-2.27442900	-4.34290100	-1.70602700
C	-1.84524400	-2.10010700	-2.81112700
C	-3.43655400	4.43064300	0.40625800
C	-3.97892500	2.22221400	1.54205100
C	-2.89540500	4.53847700	-1.95821900
C	-2.92427700	2.45175700	-3.41505800
H	-5.16762600	-1.70554100	1.13764500
H	-4.53374400	-5.26957800	0.68774100
C	-2.96957800	-5.16035100	-0.80803500
H	-1.46461300	-4.75976400	-2.31388600
H	-1.15913700	-1.43706700	-2.24289200
H	-3.53849500	4.92471100	1.37832200
C	-3.06383900	5.17270700	-0.72055300
H	-4.80567900	1.51276300	1.36861600

H	-2.56341500	5.11256200	-2.82989000
H	-2.31499400	1.54236300	-3.26962000
H	-2.69845600	-6.21659100	-0.71052000
H	-2.87829500	6.24769600	-0.62872500
H	-3.08984600	1.61994500	1.80590100
H	-4.23298900	2.86458400	2.39977900
H	-3.87724200	2.13276000	-3.87487200
H	-2.40690600	3.11120600	-4.12962500
H	-5.67635000	-3.35828800	1.58447700
H	-6.38774800	-2.55603200	0.16069900
H	-2.52740400	-1.45211600	-3.38909600
H	-1.24812200	-2.70601900	-3.51089000
H	0.18248600	-2.86947300	4.77711400
H	-0.81544900	-1.57580100	5.49083900
H	-1.55621300	-3.16243200	5.10347000
H	-3.04708600	-0.17750500	3.75980000
H	-3.33489300	-0.75313300	2.09548700
H	-3.73114000	-1.81271000	3.49026600
H	-0.30167300	-3.77565300	1.82145700
H	-1.57376700	-3.01248500	0.84045300
H	-2.02875500	-4.03605200	2.23782600
H	2.84275100	1.16136500	3.23339800
H	3.13094700	2.82482200	3.81526800
H	0.99821500	1.20411200	-1.89091200
H	0.27282600	2.78304700	-2.32176000
H	6.32273800	-0.41639400	1.51253900
H	4.78849300	-1.18765300	1.95584900
H	3.90313200	-1.58713800	-4.12925400
H	4.33558700	-0.04911900	-3.31498200
Cu	-1.45509800	-0.25395200	-0.00544500

3''

TPSS-D3(BJ)/def2-SVP

E = -4504.075767 a.u.

P	0.60094500	-1.22317200	3.52772700
Si	-1.63264700	-1.44354000	3.73208500
Si	0.43916400	-0.47023200	1.54459600
N	2.33400700	2.22364800	0.62513800
N	1.97015100	-0.17273100	0.78753800
N	3.85588700	0.87656100	-0.25426100
C	0.02264600	2.75890400	0.04442200
C	-0.56967000	3.14339800	2.77342600
C	0.33089000	2.45573900	-1.40036400
C	3.34065800	3.03678300	0.06120000
C	0.74983000	2.81252500	2.40945200
C	-2.41270500	-2.57187200	2.40480000
C	4.81192800	-1.70544900	-2.76100300
C	-1.98710400	-2.21903600	5.43311600
C	1.02465900	2.63204200	1.03608100
C	-1.57165500	3.27948300	1.80821300
C	4.93922500	-1.10101600	1.53010800
C	3.66161800	0.52631000	-3.11692200
C	2.63481300	0.86402800	0.43629700
C	1.80364300	2.56914900	3.45781300
C	3.28381500	4.52252500	0.18821100
C	4.31534600	-0.50406400	-2.22456500
C	-1.28089000	3.08628000	0.45096100
C	4.91997500	-1.30691400	0.03757400
C	5.64803800	2.50416500	-1.01530900
C	4.28805100	2.20601200	-0.47442100
C	5.39269100	-2.49809100	-0.54215900
C	5.34729600	-2.69563800	-1.92771900
C	-2.58213700	0.20622200	3.63056700
C	4.39596500	-0.31571300	-0.82441900
H	1.30347300	2.86919200	-1.71736800
H	5.59975700	-1.83297800	2.02219000
H	2.75962500	0.94406400	-2.64097800
H	2.81790800	2.75077100	3.06826400
H	4.14216800	4.98792700	-0.32069600
H	3.29416300	4.83821000	1.24837500
H	2.35591400	4.92686900	-0.25757000
H	5.78460900	3.59141800	-1.12452400
H	5.82203300	2.03586900	-2.00004700
H	6.43553400	2.12421900	-0.33697500
H	4.75442300	-1.87071900	-3.84301900
H	5.72240500	-3.62957900	-2.36000400
H	5.80291300	-3.27774100	0.10925900
H	-2.07131500	3.17974600	-0.30002900
H	-2.59479400	3.52155500	2.11413000
H	-0.81006000	3.27291700	3.83368100
C	-2.33206600	-0.73706900	-1.45094900
N	-2.12665000	-1.93137200	-2.08929300
N	-3.30136300	-0.12390400	-2.19674200
C	-2.95047300	-2.06614100	-3.20221200
C	-1.18828300	-2.89327200	-1.55072700
C	-3.69720200	-0.92085800	-3.27329800
C	-3.80165500	1.18593200	-1.86893400
H	-2.93129000	-2.94969700	-3.83655900
C	-1.70639400	-4.00485500	-0.85052400
C	0.19254900	-2.61872100	-1.63813100

H	-4.46801700	-0.60892000	-3.97467000
C	-4.51929100	1.34973500	-0.66310000
C	-3.50772100	2.25903600	-2.73744300
C	-3.19530100	-4.23142600	-0.73556200
C	-0.78773300	-4.85563900	-0.21598500
C	1.07329300	-3.48873400	-0.96783200
C	0.74877800	-1.41649800	-2.35663000
C	-4.96487600	2.64592700	-0.34717500
C	-4.77699600	0.19052200	0.26843200
C	-3.97894600	3.53513100	-2.38001100
C	-2.68815100	2.05385800	-3.99065900
H	-3.70560200	-3.31510900	-0.39065600
H	-1.16375100	-5.71405600	0.35068500
C	0.58776400	-4.59232900	-0.26212400
H	2.14586600	-3.27019000	-0.98707300
H	1.08390900	-0.67804100	-1.60467500
H	-5.52228100	2.80124800	0.58254900
C	-4.70329900	3.72724500	-1.19717000
H	-5.14429300	-0.69516200	-0.27833400
H	-3.75748600	4.38637700	-3.03269300
H	-1.83348500	1.38251500	-3.80072500
H	1.28490300	-5.24716600	0.27020300
H	-5.05586500	4.72876000	-0.93027400
H	-3.84282800	-0.11384200	0.77597500
H	-5.51265000	0.46543400	1.04001000
H	-3.28554900	1.59884200	-4.80189200
H	-2.30264500	3.01681400	-4.36149700
H	-3.40845300	-5.03303400	-0.01165900
H	-3.64701500	-4.51623800	-1.70362100
H	0.01236000	-0.93958500	-3.02156000
H	1.63479400	-1.70287600	-2.94663700
H	-1.50307900	-3.20901100	5.52206800
H	-1.59154900	-1.58139200	6.24512300
H	-3.07413300	-2.35051100	5.59771700
H	-2.22961500	0.91869000	4.39818600
H	-2.39373500	0.66949400	2.64352100
H	-3.67398800	0.06553100	3.75623400
H	-1.91330000	-3.55605200	2.36878000
H	-2.27327900	-2.10342800	1.40730300
H	-3.49746400	-2.72318700	2.57085900
H	1.74655000	1.50857300	3.77895200
H	1.63356000	3.20172700	4.34477600
H	0.37541800	1.35830100	-1.53871400
H	-0.45931300	2.84757200	-2.05978400
H	5.27820800	-0.08210800	1.78591100
H	3.91524000	-1.20834900	1.93353800
H	3.37621300	0.07665400	-4.08188500
H	4.33286500	1.37749600	-3.33241100
Cu	-1.27735100	-0.31201800	0.10207500

4’

TPSS-D3(BJ)/def2-SVP

E = -3010.632419 a.u.

P	0.37896400	-0.15544000	-2.05090600
Si	0.43019900	-2.96046500	0.77918500
Si	1.26303000	-1.09879600	-0.39403300
N	3.10480400	1.40282900	1.05028500
N	2.87685900	-0.83295600	0.12347100
N	4.89622800	0.44989200	0.19598100
C	0.94836700	2.49150300	0.61624600
C	-0.14105700	1.11812300	2.84008600
C	1.47545900	3.09020400	-0.65921300
C	4.17516800	2.31062000	1.20828900
C	1.21403200	0.93042300	2.52221900
C	1.84350000	-3.89186700	1.64874300
C	6.34475200	-1.14931400	-2.82805800
C	-0.85865200	-2.42617100	2.07413900
C	1.73702200	1.62616300	1.40638400
C	-0.93972200	1.97669600	2.07490700
C	6.46037200	-1.57350200	1.48378400
C	4.61402500	0.71388300	-2.63009100
C	3.54117800	0.23633800	0.41529500
C	2.09183200	-0.00622300	3.31411000
C	3.99874900	3.60240300	1.93579600
C	5.56822400	-0.28988600	-2.03036400
C	-0.39991200	2.65722600	0.97768200
C	6.47761600	-1.40687200	-0.01689700
C	6.69445700	2.19837000	0.53137900
C	5.28659800	1.72206000	0.66591500
C	7.23926800	-2.24480000	-0.84993700
C	7.17844200	-2.11107000	-2.24328400
C	-0.41278200	-4.09962300	-0.48208900
C	5.67410600	-0.42508000	-0.62972400
H	1.38477700	2.31003200	-1.44789400
H	6.71792900	-0.63470800	2.00543300
H	3.56524700	0.40247400	-2.45777100
H	2.36861200	-0.87877200	2.69372500
H	4.94924000	4.15782700	1.96232400
H	3.66783400	3.43199600	2.97737600
H	3.23646400	4.24312000	1.45699300

H	6.79751900	3.21176700	0.94949300
H	7.00687000	2.22811300	-0.52885600
H	7.40440600	1.53300900	1.05671300
H	6.28458600	-1.06487700	-3.91876600
H	7.77713700	-2.77169700	-2.87981800
H	7.87435800	-3.01532700	-0.39867700
H	-1.03594100	3.30537700	0.36926200
H	-1.99417900	2.11072100	2.33076000
H	-0.56911000	0.58415400	3.69516600
C	-3.67993800	0.17312200	-0.29453300
N	-4.64885900	-0.78621000	-0.15932800
N	-4.40292500	1.33437400	-0.43688900
C	-5.93334800	-0.24545800	-0.21473300
C	-4.38228700	-2.19323300	0.01701400
C	-5.77737000	1.09900000	-0.39169900
C	-3.81885600	2.64633600	-0.56139500
H	-6.82334600	-0.86621100	-0.13566900
C	-4.40874000	-2.71286200	1.32737800
C	-4.13579500	-2.98560900	-1.12310300
H	-6.50384100	1.90125300	-0.50163400
C	-3.90927700	3.51587700	0.54862400
C	-3.19856200	3.01520600	-1.77483700
C	-4.61867800	-1.80306100	2.51449800
C	-4.20125200	-4.09395600	1.48245400
C	-3.93722800	-4.36252600	-0.91791800
C	-4.03680700	-2.37087800	-2.49690800
C	-3.34378500	4.79637100	0.42507400
C	-4.58295700	3.08537200	1.83185100
C	-2.63826300	4.30492500	-1.84539000
C	-3.11898700	2.07754900	-2.95348800
H	-5.61130300	-1.31761200	2.48854600
H	-4.20887000	-4.52456400	2.48947300
C	-3.97610200	-4.91197200	0.36865100
H	-3.73685400	-5.00412600	-1.78213800
H	-4.85688600	-1.65680200	-2.68674800
H	-3.38861200	5.48379500	1.27649400
C	-2.71023600	5.18684400	-0.76149200
H	-5.68318700	3.17195500	1.76114300
H	-2.14294500	4.61432200	-2.77170000
H	-2.25609800	1.38964300	-2.84517600
H	-3.81459600	-5.98617400	0.50586000
H	-2.26537100	6.18427900	-0.83934600
Ag	-1.63802400	-0.10900400	-0.77012800
H	-1.17956400	-3.52968400	-1.03568900
H	0.31958400	-4.47804100	-1.21779300
H	-0.90396600	-4.96224700	0.00543200
H	-1.39167700	-3.30216800	2.48888500
H	-1.60771900	-1.75741700	1.61068000
H	-0.38377900	-1.87728100	2.90573100
H	1.47951300	-4.81393600	2.14149900
H	2.62591400	-4.17261700	0.92087200
H	2.32132900	-3.25791900	2.41732600
H	4.77072400	0.80718400	-3.71664400
H	4.73135500	1.71101400	-2.16965000
H	7.16881300	-2.35494000	1.80284400
H	5.44689400	-1.86042000	1.81887500
H	1.57417100	-0.36486600	4.21815400
H	3.03421700	0.48464700	3.61522700
H	2.53578100	3.38358500	-0.59415900
H	0.87395600	3.96172200	-0.96516600
H	-4.02350700	1.45278500	-3.03935200
H	-2.98575500	2.64430300	-3.88875700
H	-4.35764800	2.03261400	2.07150200
H	-4.25268300	3.71676300	2.67200300
H	-4.53553100	-2.36674000	3.45675500
H	-3.86279500	-0.99736500	2.52399900
H	-4.05635900	-3.14830700	-3.27645700
H	-3.08586400	-1.80864600	-2.59004900

TS_{iso}-4'

TPSS-D3BJ/def2-SVP

E = -3010.595956 a.u.

P	0.09910100	0.57648100	0.87046100
Si	-2.39464900	1.09440100	3.69124000
Si	-1.85177600	0.67262800	1.46253300
N	-3.29879400	-1.64115000	-0.68876600
N	-3.28550000	0.46430600	0.54823700
N	-4.70140900	-0.06023000	-1.29807200
C	-0.98729900	-2.45074600	-0.59638400
C	-1.45545300	-3.36478500	2.03932500
C	-0.69248300	-1.81584800	-1.93074600
C	-4.06775200	-2.15425500	-1.75515800
C	-2.51213700	-2.77269700	1.32687000
C	-4.26828700	1.36624200	3.88673400
C	-5.32565100	3.43449900	-2.39560100
C	-1.85822000	-0.40754300	4.73024000
C	-2.25322000	-2.32362700	0.01191700
C	-0.19382200	-3.51219400	1.45140300
C	-6.94445400	0.51980400	0.37368200
C	-3.43870800	1.83257000	-3.01546400

C	-3.68818300	-0.33433600	-0.38367700
C	-3.87609600	-2.58309600	1.94537800
C	-3.91311800	-3.56591200	-2.21580700
C	-4.71459200	2.17323200	-2.28343500
C	0.03624600	-3.06133800	0.14772300
C	-6.42957900	1.53214500	-0.61997100
C	-5.99339500	-1.12879200	-3.19169700
C	-4.93468800	-1.16607000	-2.14035900
C	-7.00912400	2.80555400	-0.75771500
C	-6.46746000	3.74434000	-1.64539300
C	-1.43755600	2.61819600	4.29398800
C	-5.30234900	1.23380800	-1.41124800
H	-1.52693100	-1.90509600	-2.64571900
H	-7.87211500	0.87065500	0.85374200
H	-2.61233700	1.69463400	-2.29340400
H	-4.06440100	-1.50995600	2.13450600
H	-4.61698800	-3.77922800	-3.03545100
H	-4.11198500	-4.27744900	-1.39287400
H	-2.88889800	-3.77127400	-2.57623500
H	-6.02729600	-2.08573000	-3.73540900
H	-5.80844200	-0.32158000	-3.92445600
H	-6.99199500	-0.94196800	-2.75482900
H	-4.89067000	4.18213500	-3.06822600
H	-6.93125000	4.73195600	-1.74281700
H	-7.88762900	3.06205700	-0.15519900
H	1.03449400	-3.14551100	-0.29084300
H	0.62571000	-3.96421300	2.02024300
H	-1.62907500	-3.70675400	3.06547000
C	4.25063700	0.18850600	-0.38917200
N	5.18352900	1.15949700	-0.64200900
N	4.93873100	-0.98253300	-0.57772200
C	6.42033500	0.61130300	-0.97929500
C	4.88439500	2.56828000	-0.55962600
C	6.26587900	-0.74633000	-0.93843700
C	4.34240900	-2.28789000	-0.43481800
H	7.28325200	1.23286200	-1.20891900
C	5.01105900	3.20907000	0.68921400
C	4.44024300	3.22669600	-1.72396900
H	6.96581300	-1.55837300	-1.12383500
C	4.24860600	-2.85179900	0.85367500
C	3.86800800	-2.93536300	-1.59466000
C	5.41337000	2.43260400	1.91952000
C	4.70049900	4.57891400	0.74683500
C	4.14169000	4.59607700	-1.61895400
C	4.24642200	2.46608500	-3.01370700

C	3.68908100	-4.13832400	0.95735400
C	4.69052800	-2.08053900	2.07295300
C	3.32011500	-4.22089800	-1.44313500
C	3.90550600	-2.24008400	-2.93436100
H	6.33471400	1.84736000	1.75144800
H	4.78417900	5.10414800	1.70414800
C	4.27467000	5.26598600	-0.39640400
H	3.78967000	5.13455600	-2.50531900
H	3.44987000	1.70859800	-2.89332000
H	3.60350900	-4.60252500	1.94559600
C	3.23992600	-4.82054100	-0.17946600
H	5.68946400	-1.63222800	1.93273500
H	2.94047300	-4.74656300	-2.32572900
H	3.31346600	-1.30729200	-2.89608400
H	4.03255900	6.33183900	-0.33136400
H	2.80380100	-5.81949100	-0.07801500
H	3.98659600	-1.25048500	2.27096300
H	4.71640100	-2.73119900	2.96099800
H	4.93378100	-1.95912600	-3.22428500
H	3.48780300	-2.88615300	-3.72226600
H	4.61726000	1.71531100	2.19320100
H	5.57792300	3.10823300	2.77330300
H	5.16289800	1.92809000	-3.31500000
H	3.95732200	3.14612900	-3.83014800
H	-2.44149100	-1.30694500	4.46305100
H	-0.79254900	-0.62970100	4.54148100
H	-1.99313400	-0.21694900	5.81239900
H	-0.36476000	2.49074800	4.06195600
H	-1.78477800	3.53355400	3.78151200
H	-1.55128500	2.76497200	5.38510200
H	-4.82690000	0.45848900	3.59402800
H	-4.61093900	2.18947100	3.23422500
H	-4.53931000	1.61264900	4.93133800
H	-3.95480200	-3.12551000	2.90108900
H	-4.67680000	-2.93488600	1.27088800
H	-0.50232600	-0.73811100	-1.75229000
H	0.21640100	-2.24973900	-2.37993300
H	-7.14173800	-0.45636800	-0.10372200
H	-6.18403600	0.35007300	1.15768000
H	-3.15854800	2.63599300	-3.71554400
H	-3.53032200	0.88873800	-3.58204300
Ag	2.27570000	0.43035900	0.16748600

E-**PMP-4’**

TPSS-D3(BJ)/def2-SVP

E = -3010.628383 a.u.

P	0.42649500	1.18738700	2.75239100
Si	-3.22383600	2.14113600	2.51321700
Si	-1.38437600	0.89616100	1.73932400
N	-1.16111100	-2.50518500	0.43295800
N	-1.96882600	-0.18346200	0.53823300
N	-2.89212300	-1.92573300	-0.79930800
C	1.27276000	-2.40905200	0.44696000
C	1.25751800	-2.40621500	3.26748300
C	1.25596900	-2.35565000	-1.06309700
C	-1.60462300	-3.66197500	-0.24287700
C	0.02730900	-2.44656200	2.58547100
C	-4.17834400	1.15032400	3.83782600
C	-4.62957800	0.02494500	-3.44373200
C	-2.55797100	3.71313600	3.34725100
C	0.06104700	-2.44777400	1.17723800
C	2.46877900	-2.38499000	2.56943300
C	-5.18757600	-1.11395600	0.70013300
C	-2.39467200	-1.19406400	-3.51544800
C	-1.96812100	-1.39956500	0.11971500
C	-1.28114800	-2.43784100	3.33273000
C	-0.99091700	-4.99659100	0.02529000
C	-3.65727100	-0.76622100	-2.80683600
C	2.48041900	-2.38438600	1.16779700
C	-5.01746100	-0.71453600	-0.74301000
C	-3.63033200	-4.12667700	-1.81313500
C	-2.68144200	-3.30226200	-1.00647300
C	-5.96072700	0.08504600	-1.41217200
C	-5.77069700	0.45000600	-2.75108300
C	-4.43251000	2.62818600	1.12148600
C	-3.87460100	-1.12611900	-1.45973500
H	2.27632100	-2.21868400	-1.45142100
H	-6.15298300	-0.76550800	1.09961900
H	-1.50758000	-0.93107400	-2.91525800
H	-1.61051300	-1.39004900	3.47634500
H	-1.48921700	-5.77401800	-0.57459300
H	-1.08180400	-5.26764000	1.09358300
H	0.08741000	-5.01160100	-0.21570500
H	-3.30253100	-5.17767400	-1.82927400
H	-3.70620700	-3.77473100	-2.85767100
H	-4.65095000	-4.08851200	-1.38856500
H	-4.48005700	0.31931500	-4.48883000
H	-6.51563800	1.07295700	-3.25757300
H	-6.85164400	0.42343700	-0.87224000
H	3.42998000	-2.34509400	0.62436600
H	3.41562100	-2.34436100	3.11754500
H	1.25649600	-2.38351600	4.36194700
C	2.66913000	1.14473100	-0.96359400
N	2.22259900	2.15719600	-1.77281000
N	4.00506000	1.06090700	-1.25765300
C	3.24807700	2.68877500	-2.55072500
C	0.82474800	2.52795200	-1.79810200
C	4.38122800	1.99382500	-2.22564800
C	4.85748500	0.06705700	-0.65651400
H	3.08251900	3.50887600	-3.24638200
C	0.31672000	3.38161900	-0.79844600
C	0.01178400	1.92129600	-2.78063400
H	5.40659900	2.08283900	-2.57872000
C	5.27403500	0.24426300	0.68012100
C	5.19062800	-1.07266000	-1.42009200
C	1.18828800	4.00895000	0.25690300
C	-1.07948100	3.57612100	-0.77243500
C	-1.36905000	2.16522700	-2.72652500
C	0.61575600	1.00178500	-3.81641000
C	6.04763800	-0.78113100	1.25554000
C	4.88398200	1.46465200	1.47583400
C	5.96713200	-2.06864200	-0.80249500
C	4.71527900	-1.22189900	-2.84681000
H	2.24891400	4.03903900	-0.03874700
H	-1.50774700	4.20952000	0.01107500
C	-1.91227300	2.96993600	-1.71733400
H	-2.02849100	1.70318000	-3.46684200
H	1.04521200	0.10215600	-3.33745500
H	6.38075100	-0.67295200	2.29324800
C	6.38908800	-1.92548000	0.52568900
H	4.94897700	2.38356300	0.86904000
H	6.23083700	-2.96807900	-1.36936100
H	3.65623300	-0.92970900	-2.94834000
H	-2.99525800	3.11533500	-1.66684000
H	6.98681400	-2.71362200	0.99532600
H	3.83327100	1.38272400	1.82256400
H	5.52983200	1.57485200	2.36111400
H	5.29355600	-0.57782300	-3.53476500
H	4.82802700	-2.26338200	-3.18742300
H	1.10539800	3.41457100	1.19212000
H	0.84923000	5.03494800	0.47822000
H	1.43565700	1.48943700	-4.37279900
H	-0.14475400	0.67623200	-4.54213900
H	-1.79501900	3.44378200	4.09961800
H	-2.06534000	4.37168300	2.60865400

H	-3.36270200	4.28865700	3.84376100
H	-4.03150700	3.48035400	0.54339500
H	-4.58967500	1.79400000	0.41677200
H	-5.41465700	2.93400100	1.53007600
H	-3.50314700	0.87783700	4.66957000
H	-4.60487200	0.21622700	3.43098400
H	-5.01031800	1.75043100	4.25494300
H	-1.17228100	-2.89519200	4.32979800
H	-2.07204400	-2.96654400	2.77565200
H	0.83099100	-3.27364000	-1.50832300
H	0.63622300	-1.51041800	-1.41040600
H	-5.12883400	-2.21058500	0.82034700
H	-4.37226000	-0.67654100	1.30290600
H	-2.31411600	-0.70758900	-4.50085800
H	-2.35942900	-2.28667500	-3.67502400
Ag	1.58189500	0.57648400	0.75413500

TS1-4'

TPSS-D3(BJ)/def2-SVP

E = -3010.592170 a.u.

P	-0.15229400	0.27182000	2.75134500
Si	2.05727900	-1.31083200	3.37345100
Si	1.87624700	0.88496600	2.33468200
N	2.11744300	2.60382200	-0.86041700
N	2.47702200	0.80182100	0.72697700
N	3.18510900	0.79081400	-1.55029800
C	-0.18041100	3.15806500	-0.24701900
C	0.79526300	4.74079800	1.87707300
C	-0.68061400	2.25029600	-1.34221200
C	2.39462000	2.77331000	-2.23482600
C	1.71645900	4.12659900	1.00978000
C	3.76030000	-1.79394100	2.64196500
C	3.34014900	-2.91997000	-1.37441700
C	2.17128900	-1.13190600	5.25706500
C	1.20436900	3.34666400	-0.04922600
C	-0.58235100	4.57281400	1.69457600
C	6.03576400	0.47247700	-1.37045800
C	1.32344800	-1.37885500	-1.43415400
C	2.57894900	1.35208900	-0.41959600
C	3.20736900	4.25623000	1.20839100
C	2.05535200	4.04399400	-2.94101600
C	2.80965300	-1.62061900	-1.45915800
C	-1.06787200	3.78786900	0.64123700
C	5.11132700	-0.71942500	-1.44737600
C	3.56731100	1.26797100	-4.01032100
C	3.05563400	1.65174800	-2.66173500
C	5.60230200	-2.03522200	-1.37693200
C	4.72491200	-3.12658800	-1.34462800
C	0.97109400	-2.83397100	2.96451900
C	3.71384800	-0.53720100	-1.51692300
H	-1.76468600	2.09710900	-1.24365100
H	7.08018100	0.14990400	-1.23181000
H	1.03086000	-0.92385800	-0.46667500
H	3.63602700	3.29079900	1.53551800
H	2.32034300	3.97450900	-4.00771700
H	2.60219300	4.90180700	-2.50554800
H	0.97823900	4.28144200	-2.86695000
H	3.31474200	2.04387800	-4.74980800
H	3.12163400	0.31269300	-4.34496400
H	4.66430100	1.12953300	-4.02017100
H	2.65269000	-3.77127500	-1.32001900
H	5.12418000	-4.14431100	-1.27591000
H	6.68415300	-2.19914400	-1.31918400
H	-2.14426700	3.63237400	0.51783600
H	-1.28491400	5.04176000	2.39166600
H	1.16925200	5.34223800	2.71264600
C	-2.86800200	-1.03787200	-0.51152600
N	-3.06240200	-2.33356800	-0.91782200
N	-4.06547700	-0.43199100	-0.79914900
C	-4.34000600	-2.52841400	-1.44241200
C	-2.05870700	-3.36663700	-0.82924300
C	-4.97596200	-1.32233300	-1.36816400
C	-4.34067600	0.96366300	-0.55894000
H	-4.67390000	-3.49960600	-1.80170900
C	-1.80728500	-3.96739500	0.42178400
C	-1.37322600	-3.72724800	-2.00870900
H	-5.98215400	-1.01825800	-1.64894600
C	-4.63639600	1.38844300	0.75377900
C	-4.30478400	1.84360600	-1.66277600
C	-2.54959200	-3.54186800	1.66383400
C	-0.81502300	-4.96403200	0.47005200
C	-0.40329300	-4.74009100	-1.91399900
C	-1.63787000	-3.00886400	-3.31107900
C	-4.89813700	2.75850100	0.94281400
C	-4.64317300	0.42802900	1.91635800
C	-4.57715500	3.20132000	-1.42406900
C	-3.95493200	1.34223400	-3.04464600
H	-3.63142100	-3.43456700	1.47306700
H	-0.58531900	-5.43504600	1.43085900
C	-0.12427100	-5.34991800	-0.68410300

H	0.15078300	-5.03327000	-2.81215600
H	-1.53384800	-1.91678900	-3.18006300
H	-5.12412600	3.11888300	1.95183500
C	-4.87163700	3.65465000	-0.13179300
H	-5.09963500	-0.53887600	1.64488700
H	-4.54401200	3.90740300	-2.26058400
H	-3.04095100	0.72307300	-3.02042000
H	0.64332800	-6.12838400	-0.62370900
H	-5.07517800	4.71670800	0.03937600
H	-3.60418200	0.21870900	2.24343900
H	-5.19198500	0.85439600	2.77063400
H	-4.75989800	0.71351600	-3.46707400
H	-3.78744300	2.18590400	-3.73241700
H	-2.18053000	-2.55737400	2.01405900
H	-2.39995300	-4.26977600	2.47571600
H	-2.66080000	-3.19416700	-3.68552400
H	-0.92653900	-3.33523200	-4.08582100
H	2.92591800	-0.37518400	5.53427800
H	1.19850800	-0.81582700	5.67188200
H	2.45415100	-2.09754000	5.71997200
H	-0.00600400	-2.78024400	3.47563100
H	0.78553500	-2.89278100	1.87706900
H	1.48462700	-3.76628700	3.27518400
H	4.54107000	-1.08286600	2.96528900
H	3.72087000	-1.76932600	1.53833600
H	4.04424100	-2.81626200	2.96359400
H	3.43850800	5.01257200	1.97546800
H	3.71260500	4.53532900	0.26698900
H	-0.47747200	2.65944200	-2.34903400
H	-0.18398400	1.26580600	-1.28752100
H	5.98785600	1.09874900	-2.27898600
H	5.75194600	1.11903900	-0.52105100
H	0.76978300	-2.32084900	-1.54905000
H	1.01232000	-0.67870900	-2.22944600
Ag	-1.36963200	-0.29866500	0.80832000

INT1-4’

TPSS-D3(BJ)/def2-SVP

E = -3010.627957 a.u.

P	-0.08923700	-1.35944200	2.04323900
Si	1.07513100	-1.22143600	3.95320300
Si	-1.92746200	-0.20243400	2.70552400
N	-3.32735900	-0.86421700	-0.65288400
N	-2.58752000	0.42141000	1.25667300
N	-3.23456500	1.33322300	-0.86606900
C	-1.94139300	-2.87704600	-0.51511100
C	-3.67552300	-3.89334900	1.46936000
C	-0.97525600	-2.29601000	-1.51607800
C	-3.74579400	-0.55145700	-1.96337300
C	-3.96655400	-2.64798900	0.88758100
C	1.45622500	0.57082200	4.47444500
C	-1.55243500	4.57214800	-0.16283900
C	0.08533600	-2.03507700	5.35577500
C	-3.08908300	-2.16401800	-0.10861800
C	-2.55523800	-4.62976900	1.06403900
C	-5.54713700	2.89054400	-0.17910500
C	-0.55792600	2.29868000	-0.72620700
C	-2.99389700	0.30230400	0.05086500
C	-5.16643700	-1.83417300	1.30875400
C	-4.21189300	-1.61144600	-2.90543300
C	-1.72780000	3.21528900	-0.48597300
C	-1.69690300	-4.12547700	0.08186800
C	-4.16966100	3.50727500	-0.24057000
C	-3.94982700	1.69515400	-3.27003600
C	-3.68482100	0.80971700	-2.09779400
C	-3.95416500	4.86344200	0.06299500
C	-2.65756900	5.39296800	0.09577000
C	2.72275700	-2.16392700	3.76926900
C	-3.04411200	2.71157000	-0.54292000
H	0.02959400	-2.71748700	-1.36521100
H	-6.29820900	3.63682200	0.12586600
H	-0.52455800	1.51398600	0.05523700
H	-4.84548300	-0.91411900	1.83052300
H	-4.52393200	-1.16296800	-3.86170400
H	-5.07327500	-2.16380200	-2.48581000
H	-3.42418400	-2.35753100	-3.11425900
H	-4.27036500	1.09514500	-4.13603000
H	-3.04121100	2.25753800	-3.55676200
H	-4.73508100	2.44469100	-3.06128400
H	-0.53672600	4.97903800	-0.10644900
H	-2.50734100	6.44944900	0.34348300
H	-4.81339400	5.50127500	0.29887500
H	-0.79764700	-4.68226300	-0.20277000
H	-2.33755000	-5.59366900	1.53629000
H	-4.33635300	-4.28404600	2.25094500
C	2.74884800	0.22126700	-1.01255400
N	3.57413100	1.31634600	-1.00890200
N	3.27375000	-0.57288400	-1.99695800
C	4.58566100	1.20532900	-1.96197000
C	3.38044700	2.45260700	-0.14154200

C	4.39522000	0.00706600	-2.58974500
C	2.71625200	-1.84508200	-2.39005700
H	5.34262200	1.97560000	-2.09313700
C	3.69061100	2.31777400	1.22811700
C	2.87003000	3.64266900	-0.70208300
H	4.94947600	-0.48886500	-3.38380400
C	3.00153200	-2.98681000	-1.61319800
C	1.90836400	-1.88208700	-3.54654400
C	4.25210500	1.03238000	1.78240600
C	3.43874600	3.42389900	2.05930900
C	2.64535100	4.72457100	0.16753600
C	2.56093600	3.74851900	-2.17813900
C	2.46596000	-4.21274700	-2.05109800
C	3.80126000	-2.89455900	-0.33771500
C	1.40900500	-3.13094900	-3.95217100
C	1.55156200	-0.61109600	-4.28040100
H	5.06012400	0.63489400	1.14405600
H	3.65422500	3.34262700	3.12955100
C	2.91786700	4.61318300	1.53633900
H	2.23943600	5.65774900	-0.23793800
H	2.06890300	2.83467000	-2.55196800
H	2.66662400	-5.11679000	-1.46662200
C	1.69024200	-4.28728900	-3.21368300
H	4.66073100	-2.21062200	-0.43924800
H	0.77786300	-3.18849900	-4.84538300
H	1.04495800	0.09604700	-3.59819100
H	2.72239100	5.46048700	2.20142400
H	1.28792300	-5.25236000	-3.53849500
H	3.16239900	-2.50006600	0.47647300
H	4.16959100	-3.88645100	-0.03285000
H	2.44453500	-0.09481000	-4.67589600
H	0.87596400	-0.82431600	-5.12338600
H	3.46430900	0.25631700	1.82977900
H	4.64160300	1.18176400	2.80073400
H	3.48077000	3.89167500	-2.77502000
H	1.89781600	4.60599200	-2.37412700
H	-0.88963400	-1.52759400	5.47617600
H	-0.11604200	-3.09727200	5.12629100
H	0.62671700	-1.98183300	6.32011500
H	2.53477900	-3.21519000	3.48394900
H	3.36373100	-1.71601800	2.98795000
H	3.29398900	-2.16135200	4.71794600
H	0.50688100	1.09457900	4.69242100
H	1.95432300	1.12513500	3.65923600
H	2.09631300	0.61906000	5.37737900
H	-5.81433400	-2.41100000	1.98801600
H	-5.76123500	-1.52038400	0.43209100
H	-1.27741800	-2.51237500	-2.55824100
H	-0.90983300	-1.20026100	-1.41058000
H	-5.85863800	2.46382400	-1.14904100
H	-5.55882100	2.06186300	0.55146200
H	0.39070900	2.85141600	-0.68897700
H	-0.63669600	1.78101000	-1.69925100
Ag	1.28245600	-0.36968000	0.39245800

INT2-4’

TPSS-D3(BJ)/def2-SVP

E = -3010.639936 a.u.

P	0.68638700	-1.76306100	1.49762200
Si	0.30531900	-3.95239000	1.11042700
Si	1.25771300	-1.19006600	-0.65254000
N	2.43455100	2.65104900	0.25456700
N	1.55690100	0.51907500	-0.43232400
N	3.79581700	0.91477800	0.40339600
C	1.01495700	4.26225500	-0.91694700
C	-0.96634400	3.91190400	1.06363100
C	2.04768100	4.36196900	-2.01447700
C	3.65856800	3.11698200	0.79109000
C	0.22947900	3.17896400	1.16474100
C	0.61558600	-4.53610200	-0.67457600
C	4.86355800	-2.42262100	-0.83433000
C	1.48435100	-4.93867200	2.23343400
C	1.21347200	3.38453100	0.17064200
C	-1.17100900	4.81377600	0.01106600
C	3.84201500	-0.51609500	2.92090900
C	4.42055300	-0.26780700	-2.11879200
C	2.48885400	1.26916900	0.02469000
C	0.45696000	2.18702900	2.27597600
C	3.84403600	4.54272900	1.19128000
C	4.51229300	-1.06057300	-0.83713300
C	-0.19008100	4.98453100	-0.97399100
C	4.29200200	-1.15054700	1.62919300
C	5.94048800	1.95054200	1.26500300
C	4.50275000	2.04316700	0.87293900
C	4.66940400	-2.50364400	1.58565800
C	4.94689000	-3.13566200	0.36682000
C	-1.47861900	-4.45970700	1.55690500
C	4.23274600	-0.44439300	0.40606500
H	2.97623900	4.85055200	-1.66847000
H	4.24905700	-1.05878800	3.78969100

H	3.36550400	-0.01726300	-2.33817900
H	1.46145000	2.30309500	2.71871700
H	4.83953400	4.68463600	1.63994400
H	3.08471900	4.84888900	1.93487200
H	3.75253100	5.23697900	0.33532000
H	6.31161100	2.93303500	1.59616400
H	6.56335400	1.61441600	0.41454900
H	6.09997400	1.22638400	2.08426700
H	5.07035100	-2.92214600	-1.78690900
H	5.21861500	-4.19662700	0.35336700
H	4.71028200	-3.07373300	2.52003000
H	-0.36445800	5.67098200	-1.81017100
H	-2.10587600	5.38199100	-0.04739200
H	-1.73860200	3.77260900	1.82803900
C	-2.88870300	-0.13950800	-0.73869900
N	-2.87266900	-0.15549900	-2.10351500
N	-4.15006200	0.29076400	-0.42730700
C	-4.09279200	0.25071100	-2.63823300
C	-1.70944700	-0.53573700	-2.87568600
C	-4.90495600	0.53395800	-1.57529400
C	-4.59689200	0.51705900	0.92465300
H	-4.26435000	0.29760800	-3.71146700
C	-1.57225500	-1.88832200	-3.25120200
C	-0.75682100	0.45472800	-3.18446500
H	-5.93743100	0.87319200	-1.52696200
C	-4.85237000	-0.59554700	1.75277300
C	-4.73343600	1.85125600	1.36529000
C	-2.57046100	-2.92225400	-2.78956000
C	-0.44212400	-2.23697900	-4.00724500
C	0.35828400	0.05591200	-3.94351700
C	-0.87061600	1.85410600	-2.63928300
C	-5.25309700	-0.33718000	3.07653200
C	-4.67996100	-2.00913900	1.25388000
C	-5.13808000	2.05829800	2.69522300
C	-4.43913900	3.01183300	0.44422000
H	-3.60730200	-2.64427400	-3.05020700
H	-0.29896400	-3.28191200	-4.30098400
C	0.50933000	-1.27114100	-4.35677600
H	1.12474700	0.79886400	-4.18736400
H	-0.45612600	1.87113800	-1.61376200
H	-5.45527700	-1.18216300	3.74326700
C	-5.39323000	0.97411500	3.54427600
H	-5.04395700	-2.12387300	0.21886200
H	-5.24348200	3.08367900	3.06599500
H	-3.48362600	2.86713800	-0.08727600
H	1.39313700	-1.56379300	-4.93256200
H	-5.70237500	1.15336500	4.57929300
H	-3.61146300	-2.29416500	1.25387100
H	-5.21787500	-2.71944500	1.90127300
H	-5.22636700	3.13318800	-0.32216500
H	-4.38098700	3.95236700	1.01455200
H	-2.52711500	-3.02504500	-1.68897500
H	-2.34984200	-3.90548500	-3.23280800
H	-1.91337000	2.20937200	-2.59580200
H	-0.28300500	2.56055100	-3.24603200
H	2.53446900	-4.70412600	1.98065900
H	1.32932600	-4.67885100	3.29671800
H	1.33643100	-6.02991200	2.11819800
H	-1.77197900	-4.06715200	2.54761400
H	-2.18836200	-4.05643200	0.81022900
H	-1.59476000	-5.56091900	1.57294100
H	1.66086700	-4.34714700	-0.97859700
H	-0.03059600	-4.00560400	-1.39610700
H	0.41720700	-5.62318700	-0.75375700
H	0.40143600	1.15206700	1.88691600
H	-0.29892600	2.29965800	3.06951700
H	2.33119700	3.35456800	-2.36633500
H	1.65816000	4.94076400	-2.86771700
H	4.12304300	0.54669100	2.99735600
H	2.73590000	-0.57626500	2.96757700
H	4.82395500	-0.84447800	-2.96659600
H	4.97444400	0.68427800	-2.03706600
Ag	-1.26902200	-0.85292500	0.40926100

TS2-4’

TPSS-D3(BJ)/def2-SVP

E = -3010.619878 a.u.

P	0.57042600	-0.61204800	2.32276200
Si	-1.03126100	-2.11021800	2.83074400
Si	0.90395300	-1.06730900	0.20423100
N	3.29965600	1.66579600	0.13427700
N	2.43071700	-0.54491900	-0.35364800
N	4.71035100	0.14213400	-0.60477500
C	1.04083600	2.53972800	-0.16973100
C	0.83724500	2.92830600	2.62076300
C	1.16723000	2.32807400	-1.65939600
C	4.56471500	2.28271400	0.03232400
C	2.03105400	2.40375000	2.09427300
C	-1.31778800	-3.49790600	1.56711800
C	5.66660200	-2.86709600	-2.56905400

C	-0.51122700	-2.94432300	4.46246900
C	2.11463300	2.23502700	0.69521300
C	-0.23877500	3.23790100	1.78484000
C	5.91126500	-1.38106500	1.49505400
C	4.32912400	-0.81087700	-3.26968400
C	3.36823400	0.32676100	-0.26564900
C	3.12557500	1.91773100	3.01153700
C	4.74113500	3.74071600	0.30118000
C	5.10702000	-1.61550700	-2.25830700
C	-0.14286500	3.03440800	0.40277100
C	5.89327700	-1.88545100	0.07133000
C	6.88588800	1.42068400	-0.79090300
C	5.44211500	1.33422100	-0.42141800
C	6.44728100	-3.12836700	-0.28208600
C	6.34143800	-3.61129900	-1.59283300
C	-2.70310900	-1.25144200	3.16984000
C	5.25592300	-1.13643200	-0.94077600
H	2.08145800	2.80621000	-2.05476900
H	6.44976300	-2.08131100	2.15345200
H	3.27042500	-0.75187500	-2.95632400
H	4.13089400	2.01547900	2.57088200
H	5.79028800	4.03487000	0.14159200
H	4.46221700	4.00448600	1.33740100
H	4.10368400	4.34905700	-0.36754000
H	7.27208900	2.43214900	-0.59034700
H	7.04064200	1.19527500	-1.86268800
H	7.49890400	0.69865600	-0.22091400
H	5.56593100	-3.25994700	-3.58701000
H	6.77538000	-4.58319800	-1.85175400
H	6.94909200	-3.72805600	0.48550600
H	-0.98469200	3.27901500	-0.24771700
H	-1.16824100	3.62752400	2.21132600
H	0.74641000	3.05447400	3.70483100
C	-3.32737600	0.11404100	-0.72762100
N	-4.16187900	-0.89343500	-1.15044200
N	-4.02163600	1.25254800	-1.05355100
C	-5.33510800	-0.39585800	-1.71497100
C	-3.88377800	-2.30621900	-1.05002400
C	-5.24764300	0.96375000	-1.65533400
C	-3.57226700	2.60345300	-0.83600600
H	-6.10811100	-1.05388000	-2.10581700
C	-4.58380200	-3.05126800	-0.07631600
C	-2.97246300	-2.88978000	-1.95614900
H	-5.93046400	1.74882700	-1.97319200
C	-3.71702700	3.17125200	0.44615700
C	-3.05207100	3.31382000	-1.93874100
C	-5.54428600	-2.38378500	0.87983500
C	-4.34989700	-4.43534000	-0.02424200
C	-2.76626400	-4.27795700	-1.85682700
C	-2.21830800	-2.06743500	-2.97043700
C	-3.36320600	4.52493800	0.59552800
C	-4.18720000	2.34347200	1.61599800
C	-2.70521300	4.66177000	-1.73954700
C	-2.84094500	2.62789200	-3.26781800
H	-6.49338100	-2.11241600	0.38124300
H	-4.87036100	-5.03477100	0.73024300
C	-3.44990000	-5.04364300	-0.90697300
H	-2.05552000	-4.75635600	-2.53870200
H	-1.34615700	-1.58460800	-2.48608100
H	-3.47183500	4.99715200	1.57764300
C	-2.87181200	5.26519000	-0.48627800
H	-5.06560600	1.72851700	1.35514900
H	-2.29139100	5.23549400	-2.57557400
H	-2.24147900	1.70920900	-3.13799600
H	-3.27051500	-6.12185000	-0.84508000
H	-2.59806100	6.31591700	-0.34709200
H	-3.38947300	1.64690500	1.93296800
H	-4.44631100	2.98553200	2.47245400
H	-3.79772200	2.32654200	-3.73142800
H	-2.31513100	3.29339800	-3.97011200
H	-5.11435300	-1.45711600	1.29429600
H	-5.78482700	-3.05717500	1.71744300
H	-2.84516100	-1.26638000	-3.39784200
H	-1.84831700	-2.70518500	-3.78906900
H	0.44537600	-3.48375800	4.33591700
H	-0.36643200	-2.19356000	5.26097100
H	-1.27431800	-3.67107500	4.80410300
H	-2.57535300	-0.42160200	3.88905000
H	-3.10644300	-0.82255500	2.23391100
H	-3.45130100	-1.95575600	3.58279400
H	-0.38156500	-4.05774700	1.39182600
H	-1.65001600	-3.10475200	0.59053900
H	-2.08524700	-4.20548800	1.93419700
H	2.94786200	0.84343600	3.21719400
H	3.10122200	2.45365200	3.97464600
H	1.23674300	1.24965300	-1.89061900
H	0.29335900	2.74418200	-2.18641800
H	6.38825300	-0.38818800	1.57868600
H	4.87831200	-1.27088600	1.87288000
H	4.38119500	-1.27112600	-4.26959900

H	4.70587800	0.22531200	-3.33645200
Ag	-1.33982300	-0.17703000	-0.03110200

7'

TPSS-D3(BJ)/def2-SVP

E = -3010.641585 a.u.

Ag	-1.08292500	-0.30993700	0.42217000
Si	0.49251800	1.24903300	1.31316200
P	0.43859800	2.78632000	2.78292500
Si	-1.76588200	2.59503200	3.18284600
C	-2.29421600	-1.93902300	-0.23414500
C	-3.97638800	-3.31346100	-0.93920500
H	-4.94703100	-3.55789300	-1.36571900
C	-2.98686500	-4.10725700	-0.42592700
H	-2.91470200	-5.18714400	-0.31409900
N	2.07316200	0.98019600	0.67929400
C	4.33265900	1.07208000	-2.06033400
C	0.93575500	2.27686900	-1.74388600
C	0.50775700	0.00049700	-2.77608600
H	0.74992600	-0.60606800	-1.88224500
N	-3.53084000	-1.99752300	-0.81244200
N	-1.97441000	-3.24775200	-0.00483800
C	-0.71374900	-3.62535900	0.59417000
C	0.35354300	-3.97329300	-0.25916500
C	1.59173100	-4.26301000	0.33834200
H	2.44176100	-4.52866500	-0.29737700
C	1.75279400	-4.18928800	1.72725100
H	2.73281200	-4.39058900	2.17107000
C	0.67310600	-3.84245200	2.54692800
H	0.80623100	-3.77731100	3.63172900
C	-0.58848500	-3.55397600	1.99640300
C	-1.74665300	-3.12651800	2.86361200
H	-2.68761300	-3.61992300	2.56528900
C	0.17625900	-3.98129900	-1.75909400
H	-0.14710500	-2.98754400	-2.11734100
C	-4.23512400	-0.81736200	-1.25135900
C	-4.26892200	-0.53467200	-2.63294300
C	-3.59787400	-1.44786200	-3.63252500
H	-4.15101900	-2.39659400	-3.75787800
C	-4.91784700	0.64428500	-3.03878300
H	-4.95202100	0.89450800	-4.10457200
C	-5.49478000	1.50438100	-2.09563400
H	-5.98692300	2.42443400	-2.42755500
C	-5.44052000	1.19781100	-0.73126100
H	-5.88496400	1.87764100	0.00245700
C	-4.81185800	0.02403000	-0.27777900
C	-4.73126200	-0.30410200	1.19224300
H	-4.93554100	-1.37167700	1.38230900
C	2.69729700	1.16014700	-0.42233700
N	2.29398300	1.86330600	-1.57306600
C	3.29114000	1.79965600	-2.56951100
C	3.13688500	2.54024700	-3.85609100
H	3.04154300	3.62898600	-3.68465100
H	2.22752200	2.22142000	-4.39885200
H	4.00652800	2.36671800	-4.50884900
C	5.70474300	0.82103600	-2.59433300
H	5.78976800	1.20259900	-3.62384400
H	5.95826900	-0.25462700	-2.60322300
H	6.47059800	1.32401700	-1.97403000
N	3.97048500	0.67520700	-0.75207200
C	5.16239500	1.57430000	1.69818700
H	4.12185300	1.80678800	1.99192000
C	5.95521900	-2.17524700	1.63583400
H	6.47728000	-2.91485600	2.25272800
C	5.90825400	-0.83595300	2.04157900
H	6.38617000	-0.53023100	2.97886500
C	5.25468900	0.13480600	1.26107100
C	4.65003700	-0.28654500	0.05704800
C	4.64734200	-1.63977000	-0.35192200
C	5.32551000	-2.57442000	0.44954100
H	5.34181900	-3.62763600	0.14555500
C	3.86253000	-2.07621700	-1.56722100
H	4.11594200	-1.49522500	-2.47020300
C	0.03322200	1.36690100	-2.34489000
C	-1.31672100	1.73830700	-2.44262300
H	-2.03719400	1.04724900	-2.88983100
C	-1.75310900	2.97268700	-1.94178600
H	-2.81428300	3.23482800	-2.00111200
C	-0.84575400	3.85502800	-1.34929000
H	-1.19510100	4.81087200	-0.94536600
C	0.52056600	3.52920600	-1.24171700
C	1.48404600	4.45052400	-0.54181800
H	2.51843600	4.29584200	-0.88778300
C	-2.23961400	0.90008800	3.91993500
C	-2.88009200	2.89313400	1.66251600
C	-2.22575700	3.91946200	4.47027900
H	4.03605600	-3.14327300	-1.78206500
H	2.78207600	-1.92936100	-1.38910400
H	5.82995700	1.77428900	2.55162800
H	5.42311200	2.25847600	0.87160600

H	1.45142300	4.24218700	0.54774800
H	1.20128400	5.50571400	-0.69234300
H	-0.27601300	-0.52573900	-3.34443400
H	1.42307100	0.04698400	-3.39102900
H	1.12135800	-4.23849000	-2.26214200
H	-0.59327800	-4.70685400	-2.07887100
H	-1.90644700	-2.03560100	2.77605900
H	-1.54795900	-3.35316200	3.92250300
H	-3.71935500	-0.09197800	1.58210700
H	-5.44596300	0.30385700	1.76767800
H	-2.57739800	-1.71171500	-3.30358800
H	-3.53324400	-0.96310800	-4.61941700
H	-3.32091900	0.82759400	4.14880500
H	-1.97602000	0.09755500	3.20492900
H	-1.67140600	0.71079200	4.84871500
H	-3.95356900	2.81284500	1.92522500
H	-2.70137800	3.90211500	1.24862800
H	-2.65743100	2.16760700	0.85779600
H	-3.30300600	3.87771300	4.72333500
H	-2.00232500	4.93288600	4.08874500
H	-1.64798700	3.77967500	5.40232200

5’

TPSS-D3(BJ)/def2-SVP

E = -2999.456763 a.u.

Au	-1.73743000	-0.18565700	-0.82414000
P	0.41703600	-0.29078600	-1.76619000
Si	0.23313400	-1.91340200	1.88552100
Si	1.26884100	-0.79999000	0.09334400
N	3.71587600	1.59524000	0.48378900
N	2.95467900	-0.70960800	0.37880000
N	5.14567500	0.06808000	-0.19585700
C	1.66104100	2.89376600	0.12005200
C	1.00659000	2.51739000	2.85089200
C	1.95935700	3.01063600	-1.34991300
C	4.94066100	2.25307600	0.23806200
C	2.20843600	1.98400900	2.35930400
C	1.54885100	-2.55281700	3.10226600
C	5.82383400	-2.73860500	-2.53488900
C	-0.96516200	-0.75742700	2.80750600
C	2.51329900	2.18239200	0.99210100
C	0.13967500	3.21770500	2.00128700
C	6.58089700	-1.64565900	1.58986100
C	4.45069700	-0.61885800	-2.88242100
C	3.83124000	0.22784300	0.22148300
C	3.14648500	1.19200100	3.23600300
C	5.10646100	3.71233200	0.50567500
C	5.32519300	-1.51835900	-2.04413800
C	0.46298000	3.40319600	0.65397400
C	6.37572100	-2.01925400	0.14098600
C	7.24766700	1.40588700	-0.64400700
C	5.82723400	1.30435200	-0.19756600
C	6.85852800	-3.22794000	-0.38976400
C	6.59008500	-3.58039500	-1.71897100
C	-0.75095000	-3.38014300	1.19394000
C	5.63642700	-1.17454300	-0.71156000
H	1.60631300	2.08013900	-1.84609600
H	7.07387800	-0.66315700	1.69758300
H	3.40236400	-0.65754900	-2.52836600
H	3.19271700	0.14404600	2.88759500
H	6.13149800	4.03133500	0.26056400
H	4.91550200	3.94843200	1.56914800
H	4.40250600	4.32094800	-0.09028500
H	7.59360400	2.44950800	-0.58388900
H	7.36410000	1.06257100	-1.68864900
H	7.91894800	0.78273400	-0.02480400
H	5.59998400	-3.02957700	-3.56715300
H	6.97176100	-4.52617600	-2.11877700
H	7.43790000	-3.90146300	0.25164300
H	-0.23375800	3.92709500	-0.00716300
H	-0.80034500	3.61832700	2.39350500
H	0.75059700	2.37681900	3.90693900
C	-3.70811100	0.03793700	-0.26181100
N	-4.66486000	-0.91327200	-0.01201700
N	-4.40653700	1.21785600	-0.23860800
C	-5.92542000	-0.33932800	0.15572900
C	-4.40425800	-2.32830300	0.09434300
C	-5.76282900	1.00863700	0.01137800
C	-3.80615900	2.51743100	-0.41945600
H	-6.81016900	-0.94282700	0.34666200
C	-4.42659400	-2.90973400	1.37996800
C	-4.16007200	-3.06935800	-1.08084700
H	-6.47391600	1.83103400	0.05201300
C	-3.52027700	3.28088800	0.73116900
C	-3.54120100	2.96793800	-1.72853600
C	-4.64828200	-2.06754800	2.61442100
C	-4.20829000	-4.29452600	1.47142000
C	-3.93404700	-4.45017800	-0.93561400
C	-4.10951500	-2.40829500	-2.43530000
C	-2.98809000	4.56756200	0.54046500

C	-3.72400000	2.70477100	2.11204000
C	-2.99146100	4.25499700	-1.86923500
C	-3.79404300	2.08263400	-2.92306400
H	-5.69563400	-1.72302400	2.69548000
H	-4.21116400	-4.76983400	2.45817100
C	-3.96411500	-5.05825600	0.32407000
H	-3.73270900	-5.04985900	-1.82952400
H	-4.92345100	-1.67342700	-2.56055900
H	-2.76211000	5.18702700	1.41506000
C	-2.72935600	5.05160100	-0.74836900
H	-4.75341900	2.33455600	2.26139800
H	-2.77061400	4.63082400	-2.87388100
H	-4.78721400	1.60345200	-2.87348600
H	-3.78389900	-6.13443300	0.41391800
H	-2.31000400	6.05472900	-0.87880800
H	-3.04338700	1.84786700	2.26877600
H	-3.51270600	3.45971200	2.88577100
H	-3.72298900	2.65652300	-3.86013700
H	-3.04131400	1.27106000	-2.95028100
H	-4.00891800	-1.16901200	2.59795400
H	-4.41188600	-2.64436900	3.52245200
H	-4.18003400	-3.15813800	-3.23882600
H	-3.15474700	-1.85841400	-2.54888400
H	-0.42993400	0.09788500	3.25511700
H	-1.70958200	-0.35395900	2.09634800
H	-1.49991500	-1.30022400	3.61018600
H	-1.50198400	-3.02505700	0.46525100
H	-0.08605100	-4.09020300	0.67012800
H	-1.27880300	-3.92610200	1.99809000
H	2.05905700	-1.71692500	3.61304000
H	2.31998300	-3.13513000	2.56657500
H	1.09673900	-3.20174100	3.87673400
H	2.80967900	1.20084100	4.28503900
H	4.17502700	1.59271000	3.19160300
H	1.42014100	3.86254200	-1.79539500
H	3.03661700	3.11384000	-1.55849000
H	5.60497400	-1.57306700	2.10353200
H	7.19378700	-2.40018200	2.10881400
H	4.46615500	-0.92686200	-3.94013100
H	4.77200500	0.43551600	-2.81605000

TS$_{iso}$-5'

TPSS-D3BJ/def2-SVP

E = -2999.416850 a.u.

Au	2.12486900	0.36688900	0.14452300
P	-0.03554400	0.53899500	0.85713200
Si	-2.44995100	1.11092100	3.69123700
Si	-1.97889200	0.66622900	1.45403900
N	-3.42739700	-1.66169900	-0.66649800
N	-3.41475800	0.45769000	0.54828400
N	-4.84648500	-0.09654900	-1.27642000
C	-1.11065500	-2.45880100	-0.58241200
C	-1.55343300	-3.34066900	2.06865300
C	-0.82971600	-1.84391500	-1.92927400
C	-4.20703000	-2.19231500	-1.71620600
C	-2.61953300	-2.76561400	1.35611300
C	-4.31511400	1.39520800	3.93673300
C	-5.53269700	3.36793800	-2.43156600
C	-1.88891700	-0.38738600	4.71996800
C	-2.37285500	-2.33200600	0.03363800
C	-0.29438400	-3.48448500	1.47462900
C	-7.03085900	0.51021500	0.46247700
C	-3.65906800	1.76315900	-3.08274700
C	-3.82179300	-0.35505300	-0.37168800
C	-3.97994300	-2.57747800	1.98320700
C	-4.04961100	-3.60856100	-2.16148500
C	-4.90973800	2.11318700	-2.31228600
C	-0.07673700	-3.04976200	0.16340100
C	-6.55797300	1.50232100	-0.57143300
C	-6.16201000	-1.19775700	-3.13497900
C	-5.08486300	-1.21391600	-2.10196100
C	-7.14967200	2.76922800	-0.71716600
C	-6.64751800	3.68946000	-1.64647400
C	-1.46176800	2.63335600	4.24117600
C	-5.45883000	1.19171500	-1.39679400
H	-1.66994500	-1.94858500	-2.63534000
H	-7.94688200	0.86350100	0.96260800
H	-2.80878600	1.63259700	-2.38778100
H	-4.17125100	-1.50412000	2.16853300
H	-4.76360100	-3.83656300	-2.96825800
H	-4.23189600	-4.31086100	-1.32696400
H	-3.02929500	-3.81146400	-2.53398200
H	-6.19785200	-2.16170300	-3.66596100
H	-5.99722900	-0.39849100	-3.88102500
H	-7.15383700	-1.01348200	-2.68197400
H	-5.12766300	4.10174200	-3.13727700
H	-7.12078400	4.67193900	-1.74976000
H	-8.00695400	3.03512800	-0.08874700
H	0.91966600	-3.12959800	-0.27951900
H	0.53333600	-3.92065300	2.04374400

H	-1.71748600	-3.67087300	3.10019500
C	4.04159700	0.15478600	-0.41789700
N	4.95382200	1.14072300	-0.70005700
N	4.73790200	-1.01276600	-0.61683500
C	6.18672400	0.60181000	-1.06629600
C	4.64015000	2.54569600	-0.61939700
C	6.05132100	-0.75688600	-1.01370500
C	4.15969800	-2.32399800	-0.46298400
H	7.03513900	1.23284100	-1.32217000
C	4.80350700	3.19892000	0.61836000
C	4.14804900	3.18789000	-1.77309700
H	6.75666100	-1.56068100	-1.21339600
C	4.11388900	-2.89716300	0.82375600
C	3.65415900	-2.96809400	-1.61118600
C	5.24935500	2.43330200	1.84005300
C	4.48475500	4.56679700	0.67378400
C	3.84278700	4.55596500	-1.67118300
C	3.90978200	2.40932700	-3.04419000
C	3.57316200	-4.19083200	0.93652900
C	4.58487600	-2.12434000	2.03067100
C	3.12802200	-4.26177000	-1.45179900
C	3.63398200	-2.25788700	-2.94307500
H	6.18198600	1.87158800	1.65385300
H	4.59644800	5.10191200	1.62279300
C	4.01464500	5.23926800	-0.46091000
H	3.45419500	5.08257200	-2.54941600
H	3.12319900	1.64946100	-2.87980900
H	3.52432700	-4.66246400	1.92382000
C	3.09652100	-4.87107400	-0.19052600
H	5.58933700	-1.69410100	1.87263600
H	2.72538800	-4.78563000	-2.32527500
H	3.03034300	-1.33452600	-2.86976900
H	3.76707500	6.30406200	-0.39797000
H	2.67655300	-5.87629200	-0.08241400
H	3.89704900	-1.28075400	2.22816600
H	4.61296500	-2.76798700	2.92376900
H	4.64713500	-1.95839000	-3.26594800
H	3.19716300	-2.90067000	-3.72336800
H	4.47578300	1.69623300	2.12571200
H	5.41346100	3.11246500	2.69119600
H	4.81731300	1.87245500	-3.37344000
H	3.58452800	3.07623600	-3.85796700
H	-2.49013400	-1.28301400	4.48182300
H	-0.83328400	-0.61991300	4.49202400
H	-1.98121700	-0.18690600	5.80474200
H	-0.39995000	2.49510100	3.96853800
H	-1.82257500	3.54610100	3.73362800
H	-1.53226300	2.79066400	5.33438400
H	-4.88738200	0.48919100	3.66583200
H	-4.67161300	2.21752700	3.29058500
H	-4.55373000	1.64885800	4.98741200
H	-4.04956400	-3.11495200	2.94241300
H	-4.78450100	-2.93579500	1.31686000
H	-0.64177700	-0.76270600	-1.77328500
H	0.07705900	-2.28135900	-2.37860300
H	-7.23202900	-0.47928400	0.01507100
H	-6.24412000	0.36785600	1.22573300
H	-3.40235400	2.55857900	-3.80064700
H	-3.76899900	0.81342300	-3.63610000

***E*-PMP-5’**

TPSS-D3(BJ)/def2-SVP

E = -2999.449764 a.u.

Au	-1.22137400	0.44968500	0.96053500
P	0.43027900	1.21756700	2.44241400
Si	3.90173500	-0.11834400	3.05015600
Si	2.08465000	0.24338800	1.60041000
N	2.48357600	1.09142400	-1.71884200
N	2.34197500	-0.53008400	0.09547300
N	2.30753500	-1.04105100	-2.23808600
C	1.36153200	3.07402000	-0.81659300
C	3.73320900	3.71962400	0.58874800
C	0.07534800	2.67894200	-1.48784800
C	2.52015700	1.00169100	-3.12642600
C	3.72623800	2.59535900	-0.25347500
C	5.15476200	-1.35655200	2.32837200
C	0.36044000	-4.20484700	-2.21538300
C	4.78882700	1.51547800	3.45774600
C	2.52605300	2.28629600	-0.93428400
C	2.58386600	4.50533400	0.73689100
C	4.40381100	-2.83032000	-1.48326500
C	-0.43070400	-1.80642600	-2.54396200
C	2.36028400	-0.17902500	-1.14442000
C	4.94413800	1.72059800	-0.42789000
C	2.75394700	2.20862100	-3.97343800
C	0.65202600	-2.83261400	-2.31236500
C	1.41484200	4.18998500	0.03654600
C	3.01081600	-3.33588400	-1.76232300
C	2.43715300	-1.02701700	-4.76634100

C	2.40257300	-0.32322000	-3.45050100
C	2.67338500	-4.69606200	-1.65527700
C	1.36190900	-5.12832300	-1.89230300
C	3.19259400	-0.81779100	4.66907100
C	1.98826300	-2.42907700	-2.11003000
H	0.22711200	2.25227900	-2.49270500
H	5.10474700	-3.66393300	-1.31666900
H	-0.72600400	-1.34905600	-1.57892700
H	4.77207800	0.72459600	0.01945800
H	2.76159100	1.93245700	-5.03938800
H	3.72219500	2.68516400	-3.73116100
H	1.97273000	2.97477800	-3.81925700
H	2.54062600	-0.29923200	-5.58620800
H	1.51766100	-1.61561000	-4.94148500
H	3.28533000	-1.73474800	-4.82084300
H	-0.66631800	-4.54630300	-2.38134200
H	1.11687400	-6.19329200	-1.81529600
H	3.45021300	-5.42001200	-1.38531500
H	0.51425200	4.79919800	0.16735500
H	2.59881200	5.36859400	1.41045900
H	4.64709300	3.97391600	1.13571700
C	-3.03831000	-0.18266000	0.21289300
N	-3.71057200	-1.34804700	0.49760100
N	-4.00919000	0.63489800	-0.30642700
C	-5.06216000	-1.24995800	0.16880500
C	-3.06697000	-2.54279100	0.98985600
C	-5.25237000	0.00342100	-0.33986600
C	-3.75871900	1.97825600	-0.77008400
H	-5.75575900	-2.07106400	0.33593100
C	-2.62339100	-2.59467100	2.32819300
C	-2.89761300	-3.61459100	0.08385900
H	-6.14349600	0.50524800	-0.71100700
C	-3.80433800	3.03760100	0.15873500
C	-3.47649800	2.16460300	-2.13882100
C	-2.84180800	-1.46552400	3.30457200
C	-1.94202100	-3.75828800	2.73389900
C	-2.21388300	-4.75344900	0.53818100
C	-3.44881900	-3.54878500	-1.32182300
C	-3.58253300	4.33638200	-0.33410900
C	-4.03109600	2.77536200	1.62644000
C	-3.27368200	3.48062000	-2.58784000
C	-3.34310800	0.98002100	-3.06519900
H	-3.71953500	-0.85496800	3.03815800
H	-1.57309200	-3.81990200	3.76320000
C	-1.72875400	-4.81984400	1.85016500

H	-2.05297200	-5.58987100	-0.15010800
H	-3.29065100	-2.55683300	-1.77487200
H	-3.60798900	5.17998100	0.36380500
C	-3.33152200	4.55737600	-1.69377800
H	-4.89317600	2.10641400	1.79399400
H	-3.05326500	3.65534700	-3.64640300
H	-2.49226400	0.34808700	-2.75260500
H	-1.18433200	-5.70858900	2.18563800
H	-3.16768100	5.57696700	-2.05820300
H	-3.14055900	2.27628800	2.05610700
H	-4.20157600	3.71604600	2.17261600
H	-4.24356900	0.34054400	-3.05002600
H	-3.16863400	1.30987300	-4.10137900
H	-1.96332700	-0.79115600	3.31336800
H	-2.97998100	-1.86328500	4.32368000
H	-4.53753300	-3.74230900	-1.33549200
H	-2.97278800	-4.30796900	-1.96223900
H	5.32474200	1.91592500	2.57972300
H	4.05772500	2.27684400	3.78370700
H	5.52487200	1.36924300	4.27159200
H	2.40084800	-0.14641000	5.04766300
H	2.73367800	-1.80929800	4.50245100
H	3.97456500	-0.92088500	5.44587700
H	5.62289700	-0.97024800	1.40502200
H	4.65630400	-2.31001400	2.07652000
H	5.96307300	-1.57150600	3.05336700
H	5.82641400	2.17054500	0.05407900
H	5.16994900	1.55707400	-1.49658000
H	-0.42862100	1.90078500	-0.87002600
H	-0.61443800	3.53327200	-1.55843100
H	4.78083000	-2.21103500	-2.31651300
H	4.39023600	-2.19010400	-0.58299100
H	-1.32252100	-2.26871300	-2.99474700
H	-0.09291500	-0.98354400	-3.19565800

TS1-5’

TPSS-D3(BJ)/def2-SVP

E = -2999.418526 a.u.

Au	1.41295400	0.26130000	0.77377800
P	0.11238100	-0.15045100	2.66047900
Si	-1.98195900	1.61921900	3.20799900
Si	-1.96860700	-0.66347000	2.33163800
N	-2.47579700	-2.51847500	-0.75815500
N	-2.61023400	-0.61954400	0.74482900
N	-3.40757900	-0.65495000	-1.50102200

C	-0.23888200	-3.29092100	-0.15881500	C	4.91022600	0.87279000	-1.65210200
C	-1.33952000	-4.71583200	2.01646200	C	4.13798300	-1.32820200	-0.73240100
C	0.33654000	-2.44453000	-1.26415600	H	4.76999600	3.05528100	-2.13633800
C	-2.83329200	-2.73415100	-2.10731700	C	2.12673400	3.87396800	0.25742200
C	-2.20502000	-4.02224800	1.15183800	C	1.49596200	3.54632600	-2.11885800
C	-3.70759900	2.12253100	2.55108500	H	5.85531000	0.46795400	-2.00777200
C	-3.23525600	3.05799500	-1.49630600	C	4.50513400	-1.76606800	0.55718800
C	-1.97697200	1.60677700	5.10301500	C	3.92106800	-2.21085600	-1.81226900
C	-1.63138000	-3.32506900	0.06733400	C	2.91777200	3.44615800	1.46757400
C	0.04457000	-4.70373200	1.80532000	C	1.22432800	4.95149100	0.31708700
C	-6.21102900	-0.08007000	-1.22644800	C	0.61677200	4.63794800	-2.01398800
C	-1.36319200	1.34411400	-1.56589800	C	1.61613500	2.75099900	-3.39792200
C	-2.80377000	-1.21254500	-0.36919400	C	4.64599600	-3.15296200	0.74993700
C	-3.69640500	-3.97997200	1.38145200	C	4.70907800	-0.79480600	1.69224400
C	-2.62741500	-4.06197500	-2.75749500	C	4.08269400	-3.58632200	-1.57303700
C	-2.82255900	1.71453300	-1.54220500	C	3.49386000	-1.69143800	-3.16532600
C	0.59207300	-3.99745400	0.72700000	H	3.97406000	3.25004700	1.21381100
C	-5.19153100	1.02142500	-1.39549300	H	1.10393600	5.48861400	1.26303900
C	-3.93911600	-1.21546700	-3.91512100	C	0.48006000	5.33192300	-0.80500100
C	-3.40891100	-1.57909400	-2.56816500	H	0.02304400	4.92995700	-2.88693100
C	-5.56351200	2.37716500	-1.36681200	H	1.47815600	1.67295700	-3.20143400
C	-4.59427600	3.38707100	-1.42017900	H	4.92244600	-3.52340100	1.74266700
C	-0.84513200	3.02313500	2.58138500	C	4.44023300	-4.05324900	-0.30178500
C	-3.81919100	0.71442200	-1.51252200	H	5.26827700	0.09915600	1.36696800
H	-0.15590000	-2.62198500	-2.23565400	H	3.90986000	-4.29418000	-2.39069700
H	-7.21566500	0.33895900	-1.05565400	H	2.63836100	-1.00003200	-3.06788300
H	-1.05820600	0.92946700	-0.58536700	H	-0.21843300	6.17219200	-0.73500500
H	-4.00631700	-2.96623100	1.69657700	H	4.55436600	-5.12838600	-0.12893900
H	-2.94592600	-4.02849400	-3.81116900	H	3.72742100	-0.44484200	2.06904500
H	-3.20894100	-4.85231300	-2.24622900	H	5.25124700	-1.27228900	2.52322500
H	-1.56723000	-4.37412500	-2.72558400	H	4.30614200	-1.13310300	-3.66527400
H	-3.80293900	-2.05209400	-4.61804900	H	3.19776800	-2.52262700	-3.82435500
H	-3.41258700	-0.33214000	-4.32214500	H	2.50144600	2.50498000	1.87771300
H	-5.01494200	-0.96195800	-3.88809800	H	2.87627200	4.21538600	2.25385700
H	-2.47542800	3.84720800	-1.50844100	H	2.60991200	2.87143700	-3.86618400
H	-4.90066400	4.43807100	-1.38244400	H	0.85538900	3.07140800	-4.12692800
H	-6.62354100	2.63827700	-1.27336900	H	-2.73185700	0.90133500	5.49234300
H	1.67651800	-3.96446800	0.57908500	H	-0.98839100	1.29792600	5.48437800
H	0.70478900	-5.23676300	2.49786200	H	-2.20589100	2.61716300	5.49475400
H	-1.76010800	-5.25757600	2.87059300	H	0.15937300	2.95900800	3.03506000
C	2.87351500	0.81225400	-0.60713100	H	-0.72502700	2.96149500	1.48524200
N	3.14417400	2.07424600	-1.07543100	H	-1.28326900	4.01253300	2.82280700
N	3.97874400	0.08476800	-0.97575400	H	-4.49563200	1.47936800	2.98127600
C	4.38281400	2.13035700	-1.71416800	H	-3.74257400	2.01168800	1.45280200
C	2.23594200	3.19096800	-0.97124500	H	-3.92227600	3.17953900	2.80720300

H	-3.99487700	-4.69106500	2.16832500
H	-4.25110400	-4.21719400	0.45660100
H	0.20661900	-1.37267600	-1.01797800
H	1.41447300	-2.63226800	-1.36667100
H	-6.26153300	-0.74284800	-2.10867800
H	-5.94434300	-0.71602600	-0.36358700
H	-0.73921900	2.22583800	-1.76708900
H	-1.15185000	0.56881900	-2.32298700

INT1-5’

TPSS-D3(BJ)/def2-SVP

E = -2999.455646 a.u.

Au	-1.23694000	0.54449000	0.18191400
P	-0.18676300	1.51457900	2.03761500
Si	-1.79668600	1.19824500	3.58203000
Si	1.31175100	-0.01157300	2.81907300
N	3.53977400	0.74529100	0.04754500
N	2.10944200	-0.62558100	1.43047100
N	3.43044300	-1.41043500	-0.42311700
C	2.24500600	2.81429400	-0.09818700
C	3.30586900	3.61184900	2.39919800
C	1.59719400	2.32530600	-1.36776800
C	4.40777200	0.51101900	-1.03633000
C	3.72513000	2.39159200	1.84179500
C	-2.28816800	-0.62513900	3.77990300
C	1.90239900	-4.63156000	-1.47183100
C	-1.18492100	1.85592200	5.25516900
C	3.17955000	2.01592500	0.59472400
C	2.39081700	4.42840100	1.72442800
C	4.31602900	-3.05424600	1.76686800
C	1.66395900	-2.31131700	-2.48416300
C	2.92367400	-0.44580100	0.45957700
C	4.72955800	1.50082800	2.53548600
C	5.25588200	1.60550000	-1.59437300
C	2.22814900	-3.26442300	-1.45832300
C	1.86913000	4.03544200	0.48688500
C	3.48677000	-3.61716700	0.64105100
C	5.16106200	-1.65700000	-2.26355600
C	4.34458900	-0.82397700	-1.33082000
C	3.11644300	-4.97335000	0.60297300
C	2.34475800	-5.48128800	-0.44997200
C	-3.34735500	2.16497000	3.04735200
C	3.04455500	-2.78351900	-0.41030500
H	2.31054700	1.83309000	-2.04991300
H	4.72975000	-3.85818600	2.39643700
H	1.14206900	-1.47642600	-1.98347200
H	4.27585900	0.53020100	2.80526000
H	5.86959600	1.23054900	-2.42825500
H	5.93157400	2.02007100	-0.82303800
H	4.64165800	2.44577300	-1.96622600
H	5.83238900	-1.01496400	-2.85496400
H	4.53888000	-2.24084600	-2.96569400
H	5.78088500	-2.38522000	-1.70704400
H	1.27278200	-5.02339600	-2.27873300
H	2.07833000	-6.54361200	-0.46881900
H	3.45062600	-5.63800600	1.40738400
H	1.13114600	4.66350100	-0.02350700
H	2.06741600	5.37196800	2.17637800
H	3.70398500	3.91910200	3.37237600
C	-2.28272200	-0.26935100	-1.38147000
N	-3.09221500	-1.37141000	-1.31601700
N	-2.42001600	0.17368200	-2.67243500
C	-3.71502600	-1.61765000	-2.53902200
C	-3.35121500	-2.10645600	-0.09788800
C	-3.29074600	-0.64256100	-3.39675700
C	-1.78773300	1.36433900	-3.18716700
H	-4.39254300	-2.45702600	-2.68033700
C	-4.56972700	-1.85387100	0.56967000
C	-2.39051300	-3.02135800	0.37810700
H	-3.52638300	-0.44899200	-4.44086300
C	-2.24348400	2.62192900	-2.73181300
C	-0.75025600	1.22426600	-4.13299300
C	-5.51122600	-0.77530900	0.08751000
C	-4.84436200	-2.59999900	1.72752700
C	-2.70960200	-3.74082100	1.54471900
C	-1.04964600	-3.18610500	-0.28759900
C	-1.61249500	3.76517800	-3.25361300
C	-3.35103800	2.74761200	-1.71519300
C	-0.15321400	2.39718500	-4.62745100
C	-0.29605300	-0.13556500	-4.60689500
H	-5.91734000	-0.99146100	-0.91704000
H	-5.77928100	-2.41887500	2.26831300
C	-3.92675600	-3.54422900	2.20465100
H	-1.97969400	-4.45524700	1.93939000
H	-0.36460900	-2.38095900	0.03955800
H	-1.94425600	4.75246500	-2.91513900
C	-0.57941800	3.65670700	-4.19196700
H	-4.16974600	2.03541200	-1.91586800
H	0.66196300	2.31343500	-5.35424200
H	-0.36686100	-0.88376300	-3.80347500

H	-4.15184900	-4.11148000	3.11346000
H	-0.10041800	4.56042800	-4.58286900
H	-2.96559800	2.52252300	-0.70173600
H	-3.76201500	3.76948800	-1.71317500
H	-0.91661900	-0.49540500	-5.44914200
H	0.74779400	-0.09629300	-4.95737000
H	-4.98369800	0.19372400	0.02383600
H	-6.35833100	-0.66202800	0.78177700
H	-1.12468400	-3.12690900	-1.38588900
H	-0.58718900	-4.14652600	-0.01461200
H	-0.27189000	1.31342400	5.56345300
H	-0.93139200	2.92956400	5.18789900
H	-1.94702500	1.72660800	6.04760200
H	-3.11673500	3.23433100	2.89026500
H	-3.73600700	1.76059600	2.09382800
H	-4.15074000	2.09050600	3.80575100
H	-1.41852000	-1.22075500	4.11431700
H	-2.61724500	-1.03379000	2.80842900
H	-3.10800200	-0.76186500	4.51200200
H	5.10141600	1.97406000	3.45818600
H	5.59139800	1.28665200	1.87737900
H	0.81402700	1.57979500	-1.11441200
H	1.10014800	3.14819900	-1.90348100
H	5.14777500	-2.44037100	1.37825600
H	3.69244300	-2.38892700	2.39155100
H	0.95018900	-2.83142400	-3.14317600
H	2.44509700	-1.85753800	-3.11940900

INT2-5’

TPSS-D3(BJ)/def2-SVP

E = -2999.463034 a.u.

Au	-1.32158200	-0.60417800	0.28426600
P	0.48268200	-1.66543700	1.45533200
Si	-0.22845200	-3.78109000	1.11443700
Si	1.17651100	-1.20183000	-0.71219000
N	2.88409100	2.35862300	0.43276700
N	1.76153900	0.41666500	-0.44379900
N	4.01170100	0.45811300	0.44021700
C	1.66770900	4.21739000	-0.59276100
C	-0.31154500	3.98810500	1.40725700
C	2.69185200	4.26112900	-1.70181400
C	4.15158600	2.62062100	1.00496600
C	0.78445900	3.10683200	1.43448800
C	-1.00687600	-4.10883200	-0.59321900
C	4.81768100	-2.79845000	-1.16221700
C	1.25841100	-4.94923500	1.31392100
C	1.76627000	3.24775300	0.42768300
C	-0.41882800	4.97318100	0.41701600
C	3.89226200	-1.24418600	2.77671100
C	4.51375200	-0.49716900	-2.20639600
C	2.76770900	1.00579600	0.08697700
C	0.90921200	2.03141000	2.48111800
C	4.50974300	3.97534500	1.51850700
C	4.55510900	-1.42494600	-1.01696500
C	0.56342400	5.08758600	-0.57525400
C	4.30002600	-1.76956100	1.42369900
C	6.26451200	1.14150400	1.37741100
C	4.85322700	1.44622100	0.99702900
C	4.58611900	-3.13233600	1.23260600
C	4.84169800	-3.64269800	-0.04631300
C	-1.52865500	-4.22196800	2.43312000
C	4.30695300	-0.93029800	0.28546600
H	3.68941400	4.56082700	-1.33383100
H	4.19910400	-1.93695800	3.57711000
H	3.47188500	-0.18010800	-2.40158900
H	1.92905800	1.99721700	2.90227400
H	5.51617100	3.95875200	1.96488400
H	3.79494600	4.30921700	2.29361800
H	4.50038200	4.74268500	0.72221100
H	6.75535700	2.04195100	1.77859400
H	6.84525800	0.79124200	0.50338300
H	6.32467600	0.34535100	2.14132000
H	5.00345000	-3.20314100	-2.16303700
H	5.04790000	-4.71070700	-0.17442500
H	4.57697100	-3.80389800	2.09773100
H	0.46616400	5.84609300	-1.36009700
H	-1.27733400	5.65396700	0.41521600
H	-1.08199400	3.89898900	2.18086500
C	-2.80144000	0.24746700	-0.86496500
N	-2.75578600	0.39849200	-2.22150700
N	-4.04708700	0.68721600	-0.51428500
C	-3.94880700	0.92362900	-2.71196100
C	-1.59866500	0.03954200	-3.01176600
C	-4.76823800	1.10732400	-1.63202200
C	-4.50506500	0.71842800	0.85268000
H	-4.09830500	1.11698700	-3.77198900
C	-1.58176600	-1.23062600	-3.62503000
C	-0.53053100	0.95288900	-3.09134900
H	-5.78447000	1.48752200	-1.55308000
C	-4.91470000	-0.48894300	1.45446400

C	-4.47668300	1.95221800	1.53501600
C	-2.73619300	-2.18724700	-3.45091000
C	-0.44052000	-1.57381900	-4.36628900
C	0.59159100	0.56256500	-3.84542500
C	-0.54305500	2.25395000	-2.33498800
C	-5.32527100	-0.43155300	2.79844500
C	-4.87082900	-1.79517000	0.69928500
C	-4.89900700	1.96021300	2.87538400
C	-3.97094900	3.20107700	0.85276800
H	-3.66097900	-1.80422300	-3.92058200
H	-0.38997700	-2.55960600	-4.84024000
C	0.63456600	-0.68308400	-4.47792700
H	1.44551000	1.24432400	-3.91253600
H	-0.12769200	2.08425100	-1.32399100
H	-5.64548500	-1.35317400	3.29583100
C	-5.32047400	0.77958200	3.50027400
H	-5.28294700	-1.69203100	-0.31935300
H	-4.88306600	2.90242700	3.43380400
H	-2.96018300	3.03952200	0.43862400
H	1.52463000	-0.97473700	-5.04471200
H	-5.64070200	0.80261800	4.54713000
H	-3.82422100	-2.13858200	0.59209100
H	-5.43521800	-2.57550900	1.23326800
H	-4.62262900	3.50232100	0.01273200
H	-3.92395200	4.04075800	1.56403300
H	-2.95173400	-2.34293200	-2.37950200
H	-2.50241800	-3.16559600	-3.89826100
H	-1.55727200	2.67115500	-2.22739500
H	0.09378700	3.00091600	-2.83320700
H	2.01275800	-4.74008000	0.53385200
H	1.74588400	-4.80441000	2.29550100
H	0.95562900	-6.01097200	1.23173200
H	-1.11894500	-4.08897700	3.45101700
H	-2.41061700	-3.56106000	2.34167200
H	-1.87147400	-5.27013900	2.33262800
H	-0.38256700	-3.68469500	-1.40100000
H	-2.00284600	-3.63325000	-0.65460000
H	-1.13161900	-5.19435600	-0.77257800
H	0.71287300	1.03537900	2.04046100
H	0.18941700	2.18958700	3.29997400
H	2.80885000	3.26040300	-2.15409700
H	2.39064500	4.97320600	-2.48724200
H	4.30676700	-0.24517800	2.98946100
H	2.78660300	-1.15630400	2.80047200
H	4.90302900	-0.99580800	-3.10862500
H	5.10362000	0.41764500	-2.02001000

TS2-5’

TPSS-D3(BJ)/def2-SVP

E = -2999.435580 a.u.

Au	1.29220900	0.18660500	-0.01111300
P	-0.50494700	0.46555400	2.27034200
Si	0.96405400	2.07899900	2.84530000
Si	-0.96305500	1.06415000	0.20103100
N	-3.43762900	-1.63706600	0.17343300
N	-2.46985500	0.51898400	-0.37052000
N	-4.77868400	-0.06686900	-0.59001500
C	-1.22958200	-2.63264300	-0.14230000
C	-1.03051400	-3.02269700	2.64841100
C	-1.34272300	-2.40821200	-1.63068500
C	-4.72819400	-2.19957100	0.08204000
C	-2.19360100	-2.42826800	2.12805400
C	1.27243700	3.45779400	1.57920700
C	-5.60394800	2.91171800	-2.65735900
C	0.26274100	2.91045500	4.40917900
C	-2.27670000	-2.26175500	0.72875300
C	0.01838300	-3.40035800	1.80556500
C	-5.88423900	1.58782600	1.46057400
C	-4.38216100	0.76410900	-3.29327200
C	-3.44701800	-0.30797500	-0.25289400
C	-3.24538600	-1.86710100	3.05254400
C	-4.97014500	-3.64174100	0.38313600
C	-5.10494000	1.64556500	-2.30524300
C	-0.07530300	-3.19534100	0.42439600
C	-5.85048000	2.04146400	0.02018000
C	-7.01308100	-1.24412900	-0.74314300
C	-5.56390000	-1.22019700	-0.38583700
C	-6.34375900	3.29754000	-0.37416800
C	-6.22800500	3.72545600	-1.70306600
C	2.63165300	1.29351400	3.32936900
C	-5.26232600	1.22509300	-0.96894500
H	-2.30546200	-2.78277800	-2.02205800
H	-6.37604400	2.33958000	2.09832200
H	-3.32461900	0.65915600	-2.98840100
H	-4.25574900	-1.87826800	2.61285000
H	-6.02965400	-3.89367300	0.21983700
H	-4.71188100	-3.89252800	1.42780300
H	-4.35359000	-4.29191000	-0.26499800
H	-7.44337400	-2.23462800	-0.52847900
H	-7.16640000	-1.02315900	-1.81591400

H	-7.58734200	-0.48886800	-0.17585200
H	-5.49571100	3.26073800	-3.69035300
H	-6.61465400	4.70811700	-1.99417500
H	-6.80673000	3.94975100	0.37472300
H	0.74884900	-3.48194300	-0.23065900
H	0.92657100	-3.84273500	2.22670400
H	-0.93900200	-3.14927100	3.73244300
C	3.17062400	-0.15416100	-0.75446400
N	4.03488400	0.82959000	-1.18537800
N	3.79843900	-1.31965000	-1.12988300
C	5.16143400	0.28903900	-1.80260500
C	3.83094700	2.24896100	-1.03053200
C	5.01310300	-1.06550600	-1.77128900
C	3.33165100	-2.65970800	-0.88907800
H	5.95103500	0.92023300	-2.20428400
C	4.56496500	2.91553500	-0.02591100
C	2.95424000	2.91555700	-1.91281700
H	5.64982300	-1.87211000	-2.12819000
C	3.48523700	-3.21436000	0.39865300
C	2.81106800	-3.38547900	-1.98158500
C	5.48278500	2.15521800	0.90179900
C	4.40436200	4.30679300	0.08316200
C	2.82211800	4.30758300	-1.75838500
C	2.16152500	2.16987900	-2.95607100
C	3.15229400	-4.57190400	0.55954600
C	3.94497600	-2.37147200	1.56096100
C	2.48365400	-4.73657300	-1.77038400
C	2.58399600	-2.71282800	-3.31501200
H	6.40623400	1.83208900	0.38628900
H	4.95287800	4.84676000	0.86232000
C	3.54173600	4.99692300	-0.77661600
H	2.14010500	4.85036400	-2.42124100
H	1.28743400	1.68215900	-2.48138400
H	3.27306700	-5.03443600	1.54489500
C	2.66892200	-5.32904700	-0.51443600
H	4.81469700	-1.74668400	1.29418100
H	2.07152400	-5.32180500	-2.59933600
H	2.01680200	-1.77458500	-3.18273800
H	3.41978000	6.07991200	-0.67188800
H	2.41335400	-6.38314400	-0.36614800
H	3.13506300	-1.68299600	1.86669300
H	4.21237900	-3.00257500	2.42308800
H	3.53591900	-2.44911900	-3.81100700
H	2.01923100	-3.37301700	-3.99191700
H	4.99030600	1.24838700	1.29033000
H	5.77507000	2.78323900	1.75791000
H	2.76043300	1.37617700	-3.43479200
H	1.79637200	2.85909300	-3.73408800
H	-0.70826000	3.39273400	4.19269900
H	0.09620300	2.16893900	5.21179700
H	0.95368300	3.68706700	4.79205200
H	2.48345400	0.50454900	4.08934000
H	3.09985700	0.82392600	2.44613300
H	3.33324600	2.04409100	3.74179100
H	0.32794300	3.97304800	1.32771700
H	1.69230100	3.06460700	0.63759100
H	1.97920800	4.20416100	1.98929400
H	-2.98241400	-0.81264800	3.26993200
H	-3.26415300	-2.41375700	4.00981300
H	-1.29522300	-1.32806100	-1.86006200
H	-0.52123300	-2.91463900	-2.16268000
H	-6.41716300	0.62766500	1.58083300
H	-4.85639500	1.43044900	1.83546700
H	-4.42047900	1.19018200	-4.30876000
H	-4.81472600	-0.25196400	-3.31936000

8’

TPSS-D3(BJ)/def2-SVP

E = -2999.469023 a.u.

Au	-1.20729600	-0.24818300	-0.07302800
P	1.26215800	-1.10645500	3.04009800
Si	-0.87153400	-1.06675900	3.74750000
Si	0.85731000	-0.56644600	1.02453000
N	3.28382000	1.67442800	0.21967700
N	2.25315800	-0.51264300	0.02870900
N	4.46644600	0.05356700	-0.69235800
C	1.08587700	2.66928200	-0.12046800
C	0.92634700	3.24651900	2.63723700
C	1.16812400	2.31262500	-1.58357500
C	4.53507700	2.22299000	-0.13580000
C	2.08954900	2.62870400	2.14351900
C	-1.94431100	-2.40233100	2.91673300
C	5.26499200	-2.84961200	-2.87496600
C	-0.84506500	-1.42376600	5.61725000
C	2.15006600	2.35748400	0.75793600
C	-0.14086800	3.55572300	1.78693700
C	5.09276600	-1.86413800	1.34593600
C	4.37138500	-0.54520000	-3.48981800
C	3.22387200	0.31681200	-0.11943900
C	3.17370300	2.16355400	3.08025600

C	4.84018800	3.66720200	0.08513000
C	4.85773600	-1.56246400	-2.48395200
C	-0.06546100	3.26374400	0.42024400
C	5.19460000	-2.20898800	-0.11868200
C	6.68698100	1.17876300	-1.17340900
C	5.27433300	1.21398000	-0.69264700
C	5.58812300	-3.48794700	-0.55258200
C	5.63408800	-3.80241100	-1.91637100
C	-1.75459700	0.60625900	3.50438200
C	4.85838400	-1.25750400	-1.10612300
H	2.16053800	2.54855900	-2.00480600
H	5.59683200	-2.62170600	1.96724300
H	3.34633800	-0.21908700	-3.23751000
H	4.17225700	2.18243800	2.61525700
H	5.85305400	3.90444900	-0.27604300
H	4.78133200	3.94089400	1.15455900
H	4.12094800	4.31384600	-0.45160000
H	7.14964300	2.17253500	-1.06873300
H	6.75978100	0.87482800	-2.23374800
H	7.28774400	0.45511000	-0.59148800
H	5.27516100	-3.10819800	-3.93993000
H	5.94760700	-4.80262000	-2.23487200
H	5.85832400	-4.24317100	0.19381700
H	-0.90562400	3.49344900	-0.23844500
H	-1.04863700	4.01375700	2.19414800
H	0.85141100	3.45430600	3.71007900
C	-3.05558400	-0.14653900	-1.07733900
N	-3.78507100	-1.24915900	-1.42859400
N	-3.77660300	0.90113300	-1.57690000
C	-4.93570100	-0.90067600	-2.13121500
C	-3.40244300	-2.59438400	-1.06525600
C	-4.93212500	0.46272200	-2.22652800
C	-3.40807000	2.29026200	-1.45084500
H	-5.63730500	-1.64807300	-2.49559600
C	-4.03617800	-3.18402600	0.04731500
C	-2.39143500	-3.23487300	-1.81019500
H	-5.63098200	1.15794300	-2.68698000
C	-3.80681700	2.99037200	-0.29438100
C	-2.69160500	2.88739100	-2.50783500
C	-5.05529800	-2.41716100	0.85554100
C	-3.64038400	-4.48424700	0.40268400
C	-2.02677500	-4.53345300	-1.41376400
C	-1.69058500	-2.53337100	-2.94768500
C	-3.50408100	4.36320200	-0.23583100
C	-4.49214800	2.27960400	0.84657900
C	-2.40883700	4.26088200	-2.40409900
C	-2.22163200	2.06123600	-3.68081700
H	-5.95702900	-2.17705800	0.26333200
H	-4.10225700	-4.96188100	1.27280200
C	-2.64680800	-5.15277100	-0.32222300
H	-1.23819200	-5.05534900	-1.96590200
H	-0.98169300	-1.78357800	-2.54527100
H	-3.80291400	4.93644000	0.64808200
C	-2.82331300	4.99420400	-1.28438000
H	-5.31952500	1.64059700	0.49226200
H	-1.85231900	4.75347100	-3.20840400
H	-1.55696100	1.24877600	-3.33563900
H	-2.34215200	-6.16129200	-0.02445100
H	-2.59525500	6.06290500	-1.21944200
H	-3.77452900	1.62389600	1.37259200
H	-4.89239900	3.00209700	1.57479200
H	-3.06671600	1.58586400	-4.21039000
H	-1.66769000	2.68310400	-4.40106300
H	-4.62885400	-1.46069800	1.20716800
H	-5.36745900	-2.99812000	1.73684000
H	-2.40118400	-1.99578400	-3.59917400
H	-1.12164200	-3.25101000	-3.55926300
H	-0.38360800	-2.40676100	5.82438800
H	-0.25719200	-0.65868200	6.15712200
H	-1.86939000	-1.42934300	6.03788400
H	-1.22489100	1.41384600	4.04031100
H	-1.76301500	0.87544600	2.43067100
H	-2.80008000	0.56852200	3.87004400

16

TPSS-D3BJ/def2-SVP

E = -4370.230221 a.u.

Fe	-0.02874500	-3.02071800	0.94153900
Si	0.36149600	-0.79137900	0.43145400
P	1.86292200	0.66410200	0.09494700
Si	3.91557800	-0.02617100	-0.53831000
O	2.18965300	-3.61714500	2.73152200
O	-1.57831900	-2.16868700	3.25044400
N	-1.19126900	-0.13689100	0.23164900
N	-1.92598600	2.14114900	0.73037200
N	-3.21469100	0.85503000	-0.51791000
C	0.56242900	-4.13032900	-0.74780900
H	1.58908600	-4.25928800	-1.08941800
C	-0.13205800	-4.97921800	0.18490200
H	0.28379400	-5.85728600	0.68098800
C	-1.44541500	-4.45431900	0.35970000

H	-2.21600500	-4.85813000	1.01802500	H	4.53679900	-2.11154100	2.65898100
C	-1.57331100	-3.28021200	-0.46022600	C	-1.99082700	0.87033200	0.15550100
H	-2.44190100	-2.62728200	-0.53515200	C	-0.71923800	2.74054100	1.23901200
C	-0.33406000	-3.09063000	-1.15212400	C	-0.05050600	3.69473100	0.43310900
H	-0.12718800	-2.31595100	-1.88938100	C	1.10791400	4.29255700	0.95825700
C	1.35344100	-3.31241800	1.98341600	H	1.64989300	5.02996800	0.35764100
C	-0.95512600	-2.45607200	2.30889400	C	1.60032800	3.92933300	2.21432300
C	4.93640200	1.64805700	-0.32230300	H	2.51779200	4.39006200	2.59523700
C	5.19512100	1.88799500	1.18373400	C	0.94183200	2.95701800	2.97328900
H	5.65832700	2.88576400	1.32327000	H	1.35516700	2.66129900	3.94080000
H	5.88293700	1.14353600	1.61778600	C	-0.23343200	2.34488300	2.50646900
H	4.25169000	1.86958100	1.75880300	C	-0.96245400	1.28655800	3.32889100
C	4.13457300	2.87620600	-0.81531700	H	-1.34409700	0.53585300	2.61656200
H	4.71542800	3.79709200	-0.60230700	C	-0.03665300	0.54681800	4.30687000
H	3.16683900	2.95582700	-0.29174600	H	0.84279000	0.13526800	3.78073300
H	3.93433200	2.85252300	-1.89711000	H	-0.58146200	-0.28791800	4.77691000
C	6.29107200	1.62383300	-1.06537600	H	0.32248100	1.20911700	5.11518300
H	6.15728300	1.58117000	-2.16008600	C	-2.18029900	1.88606200	4.06148400
H	6.92475700	0.77193000	-0.76755200	H	-1.86390900	2.66330200	4.78038400
H	6.85531200	2.55192200	-0.84174300	H	-2.71885900	1.09782000	4.61726100
C	3.81786400	-0.55263900	-2.43744400	H	-2.88809700	2.34278000	3.34951000
C	2.65402800	-1.53998500	-2.67068800	C	-0.49017700	4.02408200	-0.99246000
H	2.54611200	-1.74296000	-3.75543600	H	-1.48422900	3.57474400	-1.15655600
H	1.70886000	-1.10430200	-2.30300200	C	-0.61507400	5.53946700	-1.24231400
H	2.80890800	-2.50450700	-2.16358300	H	0.36727000	6.03974300	-1.17448700
C	3.50400600	0.69019100	-3.30133900	H	-1.28868500	6.02462600	-0.51446400
H	4.33796200	1.41123100	-3.31711000	H	-1.01268700	5.72647000	-2.25561000
H	2.60005300	1.21103800	-2.93858800	C	0.47603500	3.38780700	-2.01267600
H	3.31942300	0.37571100	-4.34855500	H	1.47947300	3.84073800	-1.93426400
C	5.12742100	-1.19357200	-2.94950400	H	0.10861000	3.54982000	-3.04186500
H	5.34978000	-2.14578400	-2.43833000	H	0.59193100	2.30709100	-1.82565800
H	5.99754200	-0.52893400	-2.81876600	C	-3.11036600	2.86254800	0.45496200
H	5.03888500	-1.41568600	-4.03241800	C	-3.90543600	2.07000100	-0.32937100
C	4.65992500	-1.45250000	0.57920700	C	-3.65527800	-0.26492000	-1.29312300
C	6.19854000	-1.56801100	0.49837100	C	-3.01723500	-0.53102900	-2.53040700
H	6.70151800	-0.67277900	0.90140400	C	-1.90728500	0.38035700	-3.04915900
H	6.55614500	-1.72402500	-0.53380000	H	-1.27184200	0.63851200	-2.18483200
H	6.53797600	-2.43378200	1.10215800	C	-0.99379400	-0.28581000	-4.08952000
C	4.04843300	-2.79644900	0.12872400	H	-0.60202600	-1.25284000	-3.73191400
H	4.31975600	-3.59574800	0.84551300	H	-0.13016600	0.36847000	-4.29610400
H	4.41109900	-3.10340000	-0.86705900	H	-1.51670800	-0.46249800	-5.04689200
H	2.94702700	-2.73814400	0.09346400	C	-2.49824500	1.68880200	-3.61738900
C	4.25541500	-1.22720400	2.05523900	H	-1.69194100	2.33511400	-4.00373200
H	3.16765000	-1.07312200	2.15136200	H	-3.04216300	2.25532700	-2.84450800
H	4.75310600	-0.34808900	2.49197400	H	-3.19544400	1.47301300	-4.44671400

C	-3.46326000	-1.64505300	-3.26330300
H	-2.99269700	-1.88495900	-4.22071200
C	-4.50459800	-2.45206300	-2.78868600
H	-4.83897500	-3.31157300	-3.37940900
C	-5.11144400	-2.17427400	-1.55902000
H	-5.91144500	-2.82434000	-1.19287300
C	-4.69055900	-1.08151700	-0.77900300
C	-5.23837800	-0.84403000	0.62493200
H	-5.20006600	0.24086700	0.82221400
C	-4.29774100	-1.50752400	1.65087300
H	-4.30522400	-2.60639400	1.53282900
H	-4.60770800	-1.27091600	2.68387000
H	-3.26414400	-1.15694800	1.50849200
C	-6.69387800	-1.29842600	0.80892300
H	-7.35949800	-0.84772800	0.05170300
H	-7.05646700	-1.00122800	1.80812700
H	-6.79252200	-2.39661300	0.73996800
C	-5.23677800	2.32972600	-0.95479000
H	-5.54786700	3.36782000	-0.76178100
H	-6.02370000	1.65753300	-0.56721000
H	-5.19510900	2.17912400	-2.04873400
C	-3.35116500	4.22150500	1.02288600
H	-4.32450200	4.60755600	0.68307500
H	-2.56838000	4.93803300	0.72307400
H	-3.35490400	4.19455900	2.12772900

17

TPSS-D3BJ/def2-SVP

E = -7314.631928 a.u.

Ag	2.29022600	-0.71654800	0.20180800
P	0.17070200	-1.59783400	-0.55155500
Si	0.70715800	-2.26844000	-2.67073200
Si	-1.33875800	0.05313000	-0.47535100
Mn	-1.21138800	2.30923200	-0.74532600
Mn	-0.93069900	5.12604500	-1.12468900
O	-4.19521900	2.41966100	-0.57022000
O	-1.08847800	2.87329000	2.19112300
O	1.75794100	2.16739400	-0.52312900
O	-1.37353100	2.15166700	-3.73696700
O	-3.25048000	4.70518100	-2.98880200
O	1.29720300	5.02132200	0.88180400
O	-2.91054800	5.49282800	1.09896100
O	1.02696900	4.33957900	-3.26266600
O	-0.68487900	8.02631300	-1.65538900
N	-2.77577900	-0.64223800	0.02969000

N	-4.09918500	-0.72881500	2.05594100
N	-5.00641400	-1.51150100	0.19476400
N	4.41780200	-0.28440900	2.43517400
N	5.36228600	0.21395500	0.56517000
C	1.52987100	-4.01833100	-2.28302800
C	2.29439100	-4.58694100	-3.50064200
H	1.65031700	-4.68890900	-4.38997600
H	2.68417700	-5.59575800	-3.25560400
H	3.15923400	-3.96201200	-3.77955200
C	0.44616100	-5.03861000	-1.86707600
H	-0.15721800	-4.65429700	-1.02685300
H	0.93168400	-5.97994800	-1.53872400
H	-0.23365900	-5.29160000	-2.69759700
C	2.50455900	-3.91937300	-1.08717900
H	3.29802900	-3.17105500	-1.23773100
H	2.99244600	-4.90194500	-0.92353600
H	1.96206500	-3.65302500	-0.16358100
C	1.97009200	-1.09440700	-3.61210300
C	2.07939400	-1.41051800	-5.12187900
H	2.38795600	-2.45148000	-5.31641000
H	2.84131900	-0.74773400	-5.57901000
H	1.13056700	-1.22916100	-5.65318000
C	1.53762900	0.37579800	-3.44534600
H	0.61074900	0.61012100	-3.98529300
H	2.32886100	1.04889400	-3.83064800
H	1.38468200	0.60764700	-2.38070500
C	3.37836900	-1.20841000	-3.00438300
H	3.36765900	-1.01121200	-1.91356400
H	4.04420400	-0.45302900	-3.45987700
H	3.83604300	-2.19711800	-3.16657600
C	-0.93902500	-2.46413400	-3.70668400
C	-0.78449300	-3.40505300	-4.92387800
H	0.00610300	-3.08131300	-5.61997100
H	-1.73738100	-3.42892400	-5.48989300
H	-0.56461600	-4.44132900	-4.61501000
C	-2.07750900	-3.00769700	-2.82371400
H	-1.89008900	-4.03368400	-2.48027100
H	-3.01488100	-3.01625300	-3.40818000
H	-2.24525100	-2.37741900	-1.93510400
C	-1.40492800	-1.08057400	-4.19884600
H	-1.49603000	-0.37337900	-3.35596600
H	-2.40701400	-1.16687000	-4.66126400
H	-0.72974000	-0.63100600	-4.94410300
C	-3.03591800	2.38951700	-0.66341100
C	-1.15275900	2.64375900	1.05368900

C	0.60344000	2.22398300	-0.66813400
C	-1.28727600	2.19713000	-2.57899900
C	-3.84699800	-0.91438300	0.68922800
C	-5.38194000	-1.23317300	2.38640800
C	-5.91519100	-1.25145500	3.78296000
H	-6.50744500	-0.35221000	4.02594300
H	-5.08879300	-1.31156800	4.51057600
H	-6.56453600	-2.13029400	3.92648100
C	-5.94435500	-1.69756800	1.23356400
C	-7.29640600	-2.27202200	0.97414600
H	-7.85041300	-2.37707700	1.91960600
H	-7.23772800	-3.26138500	0.48860900
H	-7.88238400	-1.61814800	0.30203200
C	-5.12363600	-2.21033000	-1.05748800
C	-4.93972800	-3.61724200	-1.05114200
C	-5.19848000	-4.31504800	-2.24281900
H	-5.06533300	-5.40149000	-2.26896900
C	-5.60969500	-3.64125800	-3.39881800
H	-5.80612100	-4.20376600	-4.31779000
C	-5.74945200	-2.25037800	-3.38792900
H	-6.05537000	-1.72861200	-4.30089700
C	-5.51314800	-1.50608900	-2.21674000
C	-5.69027300	0.00500900	-2.21467500
H	-5.44295400	0.36668800	-1.20472400
C	-4.71177700	0.67344300	-3.19829500
H	-3.67287800	0.42076600	-2.93686500
H	-4.81885100	1.77061400	-3.16883900
H	-4.89072300	0.34059700	-4.23644600
C	-7.14977200	0.40421700	-2.50684800
H	-7.45964000	0.09165100	-3.52055700
H	-7.26459100	1.50035400	-2.43998200
H	-7.84334800	-0.05992300	-1.78356000
C	-4.45238000	-4.36412100	0.18840300
H	-4.52744100	-3.67636800	1.04649400
C	-2.96847800	-4.75882500	0.04599400
H	-2.82105900	-5.42272400	-0.82382800
H	-2.63085200	-5.30221100	0.94584300
H	-2.32621700	-3.87349400	-0.08688000
C	-5.31601500	-5.59747200	0.51405000
H	-6.38490900	-5.33608900	0.60013900
H	-4.99313000	-6.04190200	1.47199900
H	-5.22025200	-6.37786700	-0.26189700
C	-3.14976900	-0.20697100	2.99892600
C	-1.98171800	-0.96969600	3.27188500
C	-1.79801700	-2.37361900	2.70213700
H	-2.04483300	-2.34120800	1.62746200
C	-2.76503600	-3.35006800	3.41000400
H	-2.57267400	-3.35897000	4.49801600
H	-2.62036600	-4.37567500	3.02948500
H	-3.82055800	-3.07669300	3.24992200
C	-0.36141400	-2.90040100	2.80987200
H	0.36646600	-2.19570100	2.37594900
H	-0.27972900	-3.84545600	2.24683500
H	-0.07805900	-3.10275900	3.85805400
C	-1.04844100	-0.43666700	4.17467200
H	-0.12849900	-0.98603000	4.38203200
C	-1.28747900	0.77695900	4.82899900
H	-0.54964000	1.17055200	5.53581100
C	-2.46587600	1.48243900	4.58745000
H	-2.64533100	2.43044800	5.10428200
C	-3.41790200	1.01610200	3.66184300
C	-4.68062500	1.84133100	3.42669400
H	-5.33886000	1.27352500	2.74773400
C	-5.44002000	2.10021500	4.74587600
H	-5.61915100	1.17234400	5.31366000
H	-6.41449600	2.57624900	4.53728400
H	-4.87084800	2.78567000	5.39867700
C	-4.36123800	3.18075400	2.74046700
H	-3.68910000	3.79827600	3.36014500
H	-5.28845600	3.75380100	2.56440900
H	-3.87458600	3.02300700	1.77170300
C	4.17496500	-0.23904600	1.08661000
C	3.52541900	-0.76860200	3.46420700
C	2.96029300	0.18270700	4.34466300
C	3.04754600	1.67407400	4.03689400
H	4.04333200	1.87108200	3.59993500
C	1.99314800	2.03267700	2.96770300
H	2.11062600	1.40826600	2.06570000
H	2.08340100	3.08936100	2.66552200
H	0.97608400	1.86855300	3.35766500
C	2.90378600	2.57255800	5.27422100
H	1.88565900	2.51799100	5.69910800
H	3.08047200	3.62442100	4.99228800
H	3.62367800	2.30001500	6.06601900
C	2.25669300	-0.31062900	5.45653600
H	1.81705000	0.39024600	6.17088900
C	2.10539300	-1.68744200	5.65771100
H	1.55361800	-2.05164800	6.53059300
C	2.63325700	-2.60089900	4.73921200
H	2.48282200	-3.67185300	4.89799500

C	3.36351900	-2.16470900	3.61911400
C	3.98285000	-3.15478200	2.63476800
H	3.92993600	-2.68608500	1.63528100
C	3.21412500	-4.48206000	2.54840300
H	3.30599000	-5.07165000	3.47830700
H	3.62397200	-5.09407100	1.72754000
H	2.14451500	-4.31253300	2.34627900
C	5.47098900	-3.40733400	2.96054300
H	6.05864900	-2.47455700	2.94235400
H	5.91087000	-4.09945300	2.22077900
H	5.57736100	-3.86091100	3.96222600
C	5.70857200	0.14056200	2.74824300
H	6.06930900	0.16903500	3.77383600
C	6.30421400	0.45611900	1.56481500
H	7.30174900	0.81990400	1.33257200
C	5.72813900	0.31102200	-0.83286400
C	5.54969900	1.54037100	-1.50534900
C	6.02745800	1.62264900	-2.82635500
H	5.90084000	2.55482700	-3.38365800
C	6.66064800	0.53409000	-3.43532100
H	7.02745200	0.62339000	-4.46322400
C	6.81162400	-0.67344000	-2.74557800
H	7.29131100	-1.52286600	-3.24070600
C	6.34316200	-0.81610800	-1.42811000
C	6.52743800	-2.12750100	-0.66800000
H	5.76906900	-2.15202800	0.13351100
C	7.92144700	-2.19085800	-0.00585500
H	8.71446700	-2.15919500	-0.77422500
H	8.03287700	-3.12861200	0.56662600
H	8.08456700	-1.34944800	0.68664600
C	6.30710900	-3.37070700	-1.54812600
H	5.34518900	-3.32438800	-2.08134900
H	6.30670100	-4.27801800	-0.91942300
H	7.11144500	-3.48822100	-2.29569600
C	4.90775500	2.74186400	-0.81962500
H	4.19383300	2.35076100	-0.07633900
C	4.12059700	3.63448300	-1.79312100
H	4.79389400	4.19361900	-2.46775800
H	3.53295700	4.37367800	-1.22432300
H	3.42094300	3.04681600	-2.40659200
C	5.96648800	3.57937000	-0.06821000
H	6.49293400	2.98751100	0.69877700
H	5.48354800	4.43462100	0.43657300
H	6.72075300	3.97510600	-0.77188800
C	-2.34903800	4.83362100	-2.27169400
C	0.42889500	5.03578000	0.11151900
C	-2.14784500	5.31192500	0.24571300
C	0.26293200	4.61840900	-2.43694800
C	-0.78263900	6.88735900	-1.44636300

18

TPSS-D3BJ/def2-SVP

E = -4290.631164 a.u.

Mn	-1.53615400	-2.60081700	-0.76238800
Si	-0.35663600	-0.85665300	0.06650500
P	-2.34983000	-0.72263400	0.86510500
Si	-3.55007500	1.12320600	0.24126500
O	-1.23979700	-1.29296700	-3.43773800
O	0.10868500	-4.80236700	-1.85353000
O	-1.57547800	-4.02257800	1.88595900
O	-4.25629000	-3.62127400	-1.43511000
N	0.87571700	0.27715200	0.02895600
N	2.97274200	1.43196100	-0.09724200
N	3.02570000	-0.60272300	0.72080400
C	-1.37509600	-1.74355300	-2.37316300
C	-5.20212500	0.41217200	-0.53806900
C	-6.35701600	1.44029300	-0.51062500
H	-7.24708100	1.00829700	-1.01021600
H	-6.10532900	2.37674900	-1.03561000
H	-6.65430900	1.69668700	0.52063400
C	-5.66848700	-0.85878100	0.21245400
H	-6.00713000	-0.64291500	1.23675100
H	-4.86883200	-1.61434100	0.27406400
H	-6.51847300	-1.31225800	-0.33429900
C	-4.93827400	0.00128500	-2.00540700
H	-4.08297100	-0.69035400	-2.08648000
H	-4.74210800	0.87114900	-2.65438300
H	-5.82818700	-0.52045100	-2.40844200
C	-2.62271300	2.35381500	-0.96328600
C	-1.93986600	1.59367100	-2.11639200
H	-1.41540800	2.31516000	-2.77236200
H	-2.63564000	1.01332200	-2.73796800
H	-1.17275500	0.90381200	-1.72572800
C	-3.59010400	3.39667300	-1.57189000
H	-4.12618800	3.97781800	-0.80318700
H	-4.34002100	2.92995800	-2.23263000
H	-3.01131100	4.11401300	-2.18757700
C	-1.49280000	3.09829500	-0.21878400
H	-0.77398100	2.39253200	0.22885800
H	-1.87137300	3.77121400	0.56815700

H	-0.92955100	3.71864800	-0.94132800
C	-3.85349500	1.93229600	2.00860100
C	-4.44170200	3.35785300	1.90032600
H	-5.38788700	3.37997800	1.33343600
H	-3.74038500	4.06086000	1.41991200
H	-4.65382300	3.74863400	2.91586300
C	-4.82767200	1.06226100	2.83456200
H	-4.89773300	1.46924200	3.86309700
H	-4.47530400	0.01825100	2.90893300
H	-5.84619800	1.05756100	2.41248200
C	-2.53239000	1.99927800	2.81151400
H	-1.77374000	2.62865300	2.32583500
H	-2.09798800	0.99328900	2.95098000
H	-2.73480000	2.42546100	3.81488000
C	2.16981200	0.34502000	0.18440900
C	4.31270100	1.16118100	0.25152600
C	4.34664300	-0.10837800	0.76620500
C	5.47575100	-0.92952800	1.29449400
H	5.62127400	-1.85122400	0.70305700
H	5.29249300	-1.24122400	2.33800800
H	6.41077300	-0.34955300	1.26584500
C	2.59875200	-1.93698200	1.02688800
C	2.87177300	-2.95718800	0.08592100
C	2.42965600	-4.25421700	0.39687400
H	2.60461400	-5.06911200	-0.31015800
C	1.73478300	-4.50899300	1.58370800
H	1.38114600	-5.52248900	1.79810600
C	1.45972200	-3.47603700	2.48675900
H	0.88931300	-3.69238500	3.39315700
C	1.87765900	-2.16025800	2.22609600
C	1.58640000	-1.01695900	3.19619800
H	1.49514600	-0.09650400	2.59490400
C	0.25878200	-1.19232200	3.95178700
H	-0.57974000	-1.36487700	3.25528300
H	0.03737400	-0.27786100	4.52879000
H	0.29963900	-2.03352000	4.66646000
C	2.76117800	-0.81108200	4.17482100
H	2.92331000	-1.71522300	4.78834400
H	2.55059900	0.03398100	4.85424600
H	3.69796500	-0.58859200	3.63706800
C	3.55948800	-2.63736500	-1.23921800
H	4.18313700	-1.74009100	-1.08071500
C	4.48823500	-3.76327900	-1.72314300
H	5.22214600	-4.04479900	-0.94746900
H	5.04149300	-3.43597000	-2.62059600
H	3.91962200	-4.66801600	-2.00068000
C	2.52141600	-2.27149900	-2.32215200
H	1.91143500	-3.14880300	-2.58981500
H	3.03177700	-1.91142200	-3.23297600
H	1.83828300	-1.48070300	-1.97249300
C	5.38816600	2.16998300	0.02451800
H	6.36094100	1.76826600	0.34635200
H	5.19555300	3.10345200	0.58339800
H	5.45986300	2.44093100	-1.04427000
C	2.47841700	2.67984300	-0.60618900
C	2.30081800	2.82299600	-1.99870000
C	2.66798200	1.70791000	-2.97103500
H	3.12742500	0.89587500	-2.38206900
C	1.42209500	1.12452300	-3.66210700
H	0.71220000	0.73295700	-2.91975100
H	1.70526500	0.29858000	-4.33705000
H	0.90131500	1.89181300	-4.26232200
C	3.71545400	2.18232300	-3.99776700
H	3.30948300	2.97442700	-4.65190500
H	4.02489000	1.34128600	-4.64308100
H	4.61474500	2.58475800	-3.49908100
C	1.78925800	4.04890100	-2.46268300
H	1.62666400	4.19262400	-3.53601300
C	1.49333300	5.08592700	-1.57219400
H	1.09487000	6.03257100	-1.95217700
C	1.70543100	4.92413500	-0.19690000
H	1.47251700	5.74718100	0.48528100
C	2.20170800	3.71494300	0.31775100
C	2.40378100	3.50077000	1.81527100
H	3.15248300	2.69754100	1.93434800
C	1.10151700	3.00291300	2.47584000
H	1.27363100	2.76936600	3.54176200
H	0.72169500	2.09806300	1.97491000
H	0.31716800	3.77731300	2.41579600
C	2.95061600	4.74788700	2.53056100
H	2.21282500	5.56972400	2.54024400
H	3.87025100	5.12162500	2.04693000
H	3.18574800	4.50708800	3.58192600
C	-0.53121600	-3.92633100	-1.42402800
C	-1.53941800	-3.44722100	0.87945400
C	-3.19956000	-3.22401800	-1.16366900

19

TPSS-D3BJ/def2-SVP

E = -4409.091554 a.u.

Si	-0.46100800	-0.85222300	-0.14481300	C	2.10823600	0.31617200	0.15797100
P	-2.32480500	-1.10868200	0.88232900	C	4.20897100	1.22790200	0.26713100
Si	-3.58773400	0.75622100	0.38010700	C	4.26796100	-0.00340800	0.86440100
N	0.83898000	0.16616700	-0.08032100	C	5.40025900	-0.73727200	1.50308200
N	2.87610100	1.42057600	-0.15352600	H	5.65105700	-1.66357200	0.95611800
N	2.96877800	-0.55633900	0.79794900	H	5.15021100	-1.03010300	2.53811100
C	-5.28191700	0.03287700	-0.29355700	H	6.29649100	-0.09876700	1.52934600
C	-6.46009400	1.02632500	-0.16781800	C	2.60367500	-1.88060900	1.21393700
H	-7.36819500	0.57948700	-0.61985300	C	3.04453600	-2.96918200	0.42402600
H	-6.27054000	1.98165300	-0.68374400	C	2.74684900	-4.26229300	0.88345400
H	-6.69765900	1.24914000	0.88669900	H	3.06489900	-5.13040100	0.30027900
C	-5.67312700	-1.26161700	0.45969700	C	2.00058600	-4.45297700	2.05054900
H	-5.96225300	-1.06750000	1.50367300	H	1.75727000	-5.46882800	2.37770900
H	-4.85588400	-2.00007500	0.46784000	C	1.52933600	-3.35758800	2.78175000
H	-6.54081800	-1.72809200	-0.04689600	H	0.92099100	-3.52854400	3.67326700
C	-5.09565700	-0.33884800	-1.78397000	C	1.82828800	-2.04093500	2.38830500
H	-4.19849200	-0.96302500	-1.93558400	C	1.35987400	-0.83142300	3.19700400
H	-5.00413400	0.55380500	-2.42549800	H	1.03879800	-0.06381100	2.47072500
H	-5.97324300	-0.91412000	-2.13809800	C	0.14697300	-1.13371800	4.08945100
C	-2.80288300	2.05070500	-0.87321900	H	-0.68408800	-1.55722400	3.50024500
C	-2.17628300	1.36929400	-2.10627500	H	-0.20869200	-0.20007500	4.55674200
H	-1.78857100	2.14312000	-2.79761000	H	0.40196900	-1.83439700	4.90491600
H	-2.87011500	0.72378800	-2.66304200	C	2.51365300	-0.23052800	4.02814800
H	-1.30944500	0.74901600	-1.81715900	H	2.90511400	-0.97216800	4.74717000
C	-3.86459800	3.06079900	-1.37131900	H	2.15454700	0.64455400	4.59815900
H	-4.37417600	3.58244500	-0.54443900	H	3.34576900	0.10494400	3.38758900
H	-4.63356600	2.57828600	-1.99713100	C	3.72866300	-2.73616800	-0.92081700
H	-3.36849600	3.83088800	-1.99541500	H	4.23606700	-1.75636900	-0.87727600
C	-1.65914400	2.83904800	-0.19857500	C	4.79487600	-3.79267200	-1.25334100
H	-0.87820800	2.16443800	0.18918000	H	5.53914500	-3.88743000	-0.44293100
H	-2.01388500	3.48052100	0.62453900	H	5.32617100	-3.51398000	-2.17992000
H	-1.17547300	3.49608100	-0.94654800	H	4.34502100	-4.78706400	-1.42048600
C	-3.78431300	1.53544900	2.17532000	C	2.66854000	-2.64220400	-2.04088400
C	-4.43732100	2.93616400	2.14897500	H	2.14175300	-3.60353900	-2.16048600
H	-5.42605000	2.92974500	1.65996000	H	3.14656200	-2.38264500	-3.00228200
H	-3.80649200	3.67887100	1.63185500	H	1.91054000	-1.87483200	-1.81309700
H	-4.58260800	3.29632000	3.18745000	C	5.26041300	2.25231400	-0.00022400
C	-4.65455100	0.60963600	3.05609600	H	6.22883100	1.91611800	0.40016300
H	-4.65287400	0.98862100	4.09770000	H	5.01238100	3.22493500	0.46093800
H	-4.26278400	-0.42303200	3.07412100	H	5.37502500	2.42764600	-1.08538200
H	-5.70391800	0.57721600	2.71961100	C	2.34523700	2.60601800	-0.76419300
C	-2.40791700	1.64174700	2.87178900	C	2.13911500	2.61571100	-2.16019200
H	-1.72136800	2.32121800	2.34807900	C	2.53228500	1.43286500	-3.03714300
H	-1.92018100	0.65433400	2.93808400	H	3.02739700	0.69048700	-2.38804000
H	-2.54446900	2.02703600	3.90257600	C	1.29738500	0.75212400	-3.65352400

H	0.61272100	0.41097300	-2.86401100
H	1.59353800	-0.12606800	-4.25196700
H	0.74288100	1.44322800	-4.31290900
C	3.55062900	1.85150900	-4.11616700
H	3.11116000	2.57359800	-4.82715400
H	3.87740500	0.96919300	-4.69426400
H	4.44316300	2.32087200	-3.66607100
C	1.56984600	3.77349800	-2.72187100
H	1.38211400	3.81180500	-3.80000100
C	1.24897600	4.87552200	-1.92310200
H	0.80620200	5.76764500	-2.37850600
C	1.49434900	4.84895900	-0.54403000
H	1.24243400	5.72203300	0.06521200
C	2.04485200	3.71067400	0.06792000
C	2.27143800	3.64082000	1.57591900
H	3.06403300	2.89390700	1.75792600
C	1.00567500	3.12919200	2.29249900
H	1.19425000	3.00951200	3.37404200
H	0.68462600	2.15741400	1.88513100
H	0.17222900	3.84101200	2.16370700
C	2.75032500	4.97398700	2.17469000
H	1.96560900	5.74962100	2.12577000
H	3.64156500	5.35772100	1.64771100
H	3.00934700	4.83761600	3.23904000
Co	-1.53824700	-2.43118100	-1.11890500
C	-1.29813700	-1.88163100	-2.77814000
O	-1.11172400	-1.59842900	-3.89251600
C	-3.11874500	-3.25404800	-1.19071100
O	-4.13000700	-3.82118000	-1.23460800
C	-0.50757400	-3.80192600	-0.71336700
O	0.13426900	-4.76582400	-0.59049600

$Mn_2(CO)_{10}$

TPSS-D3BJ/def2-SVP

E = -3434.786648 a.u.

Mn	-1.43476700	-0.00015600	-0.00008500
C	-3.23897400	-0.00180400	0.00003900
O	-4.39696400	-0.00266300	0.00016300
C	-1.30284300	0.23263800	-1.83212000
O	-1.26183500	0.37789100	-2.97750200
C	-1.30233900	-0.23348800	1.83181300
O	-1.26078800	-0.37940100	2.97709800
C	-1.30610800	1.83213800	0.23301100
O	-1.26698300	2.97753500	0.37858600
C	-1.30235300	-1.83211800	-0.23302600
O	-1.26096600	-2.97747500	-0.37847500
Mn	1.43443900	0.00025700	0.00002500
C	3.23864900	0.00231900	0.00025600
C	1.30474100	-1.46020200	1.13082900
C	1.30257500	1.46028500	-1.13083900
C	1.30191500	1.13116900	1.45999800
C	1.30427500	-1.13095800	-1.45993200
O	4.39663800	0.00384000	0.00028900
O	1.26545300	-2.37321900	1.83754000
O	1.26189400	2.37317300	-1.83768900
O	1.26056600	1.83811200	2.37279800
O	1.26435600	-1.83809400	-2.37264100

E-**3**

TPSS-D3(BJ)/def2-SVP

E = -5486.470907 a.u.

P	-0.43259800	1.62162300	-0.59557100
Si	2.90915500	3.09116400	-0.33952100
Si	1.58318600	1.10525500	-0.48167800
N	1.55173200	-2.41468800	-1.17418300
N	2.47307100	-0.35230200	-0.22013300
N	3.34038300	-2.56486300	0.09555100
C	-0.87756400	-2.27128900	-1.08066300
C	-0.87067100	-0.84462300	-3.52377500
C	-0.84524600	-2.94650400	0.28822000
C	1.91190400	-3.78048400	-1.11648200
C	0.36259600	-1.21574400	-2.95582700
C	4.77500100	2.68806200	-0.83270300
C	5.44054500	-1.48027100	2.96515000
C	2.13613100	4.42300700	-1.58800400
C	0.32645000	-1.94934500	-1.75040400
C	-2.07784300	-1.19305000	-2.91611800
C	5.87371100	-2.44885200	-1.26015900
C	3.00580100	-2.24579500	2.95913900
C	2.43093800	-1.62896000	-0.41951500
C	1.69623100	-0.87088700	-3.61259100
C	1.16196400	-4.82700100	-1.87433300
C	4.32312100	-1.95014200	2.25153600
C	-2.08728600	-1.88284200	-1.69118700
C	5.73715800	-2.09147600	0.21831000
C	3.80574400	-5.05090800	0.14760100
C	3.02031300	-3.87033100	-0.31942900
C	6.82870600	-1.63892600	0.97774300
C	6.67536900	-1.31000800	2.33090500
C	2.78724100	3.59131900	1.57041100

C	4.48534900	-2.19642000	0.87098500
H	2.32956700	-2.68959400	2.20950500
H	2.38061100	-0.58105000	-2.79477400
H	1.64742800	-5.80668400	-1.74381800
H	1.13571200	-4.59322700	-2.95391900
H	0.11184800	-4.91426300	-1.54127800
H	3.40518300	-5.97528700	-0.29627800
H	3.75789200	-5.14635400	1.24804600
H	4.87423000	-4.97077500	-0.12173300
H	5.34326600	-1.25621700	4.03298600
H	7.53363600	-0.93644600	2.89967100
H	7.80954000	-1.53091500	0.50516500
H	-3.04058500	-2.16433600	-1.24165100
H	-3.02582000	-0.91295600	-3.38114200
H	-0.88657000	-0.27174900	-4.45430100
C	-3.79964200	0.01112400	0.60227100
N	-4.14300600	0.29861100	1.90656000
N	-5.01079900	0.00805800	-0.05053600
C	-5.51618700	0.48300600	2.05080200
C	-3.12637200	0.63269400	2.88104300
C	-6.06712000	0.29769200	0.81604000
C	-5.17588700	-0.16536700	-1.47386500
H	-5.97188200	0.74336300	3.00246400
C	-2.59049300	1.94238500	2.82989600
C	-2.66813300	-0.35559400	3.78079400
H	-7.09651000	0.35736600	0.47113800
C	-4.81753500	0.90085100	-2.33646200
C	-5.71819600	-1.38241200	-1.94554400
C	-3.24027900	3.02402800	1.97225000
C	-1.50239900	2.22490900	3.67235200
C	-1.58537100	-0.01477400	4.61110800
C	-3.35599000	-1.71492700	3.87328200
C	-5.08227800	0.73253500	-3.70733100
C	-4.17420900	2.17933800	-1.80890500
C	-5.95614800	-1.49881900	-3.32761900
C	-6.03935800	-2.53890300	-1.00285500
H	-1.03874000	3.21373100	3.63711000
C	-0.99439800	1.25202500	4.53928500
H	-1.18757800	-0.75484800	5.31113200
H	-3.67214800	-1.98819200	2.84992400
H	-4.82104700	1.53553300	-4.40189300
C	-5.65620500	-0.44642600	-4.19808200
H	-3.54640400	1.88625300	-0.94578200
H	-6.37679300	-2.42802400	-3.72488600
H	-5.53818300	-2.32887900	-0.04154300
H	-0.12978100	1.48489700	5.16957500
H	-5.85216800	-0.55225900	-5.27031000
H	-3.70127600	2.52228500	1.10512800
H	-0.04645600	-3.70896700	0.26757300
H	5.09442100	-3.19750400	-1.48607300
C	1.72056900	3.71730400	-2.90149300
H	0.89097500	3.01526400	-2.71020200
H	1.37081400	4.47728400	-3.63030400
H	2.54594200	3.15978400	-3.37181700
C	0.85886100	5.10961900	-1.05252800
H	0.12133600	4.36959600	-0.69920200
H	1.07832100	5.82389800	-0.24303800
H	0.39621900	5.68561500	-1.87998100
C	3.14871400	5.53886800	-1.93129300
H	4.00033200	5.16156700	-2.52154000
H	2.64502100	6.31304400	-2.54490700
H	3.54865400	6.03901900	-1.03066000
C	4.76636100	2.17024300	-2.28932300
H	4.53615700	2.97697000	-3.00565600
H	5.76441700	1.76944500	-2.55540300
H	4.02615300	1.36356100	-2.43642200
C	5.80157200	3.84341700	-0.76005500
H	6.79842600	3.45101500	-1.04911600
H	5.56684100	4.67276800	-1.44357800
H	5.89800700	4.25802300	0.25522800
C	5.30002400	1.56112800	0.08761100
H	5.47344300	1.92836700	1.11372000
H	4.61002600	0.70196200	0.13944800
H	6.27704400	1.19933100	-0.29017900
C	-2.45086800	-2.83631000	4.40842300
H	-2.21748400	-2.68959200	5.47810400
H	-2.96434600	-3.80892300	4.31542300
H	-1.50067400	-2.89396700	3.85542300
C	-4.62431800	-1.62119100	4.75188700
H	-4.35472800	-1.32958900	5.78250700
H	-5.33833000	-0.87671500	4.36653500
H	-5.13959800	-2.59759100	4.79264400
C	-5.49826500	-3.88407500	-1.52557000
H	-5.63279500	-4.66871400	-0.76064000
H	-6.03462300	-4.21085800	-2.43387500
H	-4.42534200	-3.81978400	-1.77000700
C	-7.55624700	-2.63461600	-0.73494500
H	-7.77403500	-3.45318500	-0.02610900
H	-7.95374600	-1.69828900	-0.30923900
H	-8.10422900	-2.83747900	-1.67254300

C	-3.21767900	2.83726700	-2.81379600
H	-2.46312800	2.11355200	-3.16455700
H	-3.75066800	3.26035700	-3.68529400
H	-2.67269100	3.65668500	-2.31680300
C	-5.24608800	3.17026900	-1.31011000
H	-5.87514600	2.72429600	-0.52066200
H	-4.76497400	4.07169400	-0.89262500
H	-5.90675700	3.48380500	-2.13878200
C	-4.36606600	3.70980300	2.78001700
H	-5.10334400	2.98310900	3.16086900
H	-3.94383000	4.24752100	3.64781600
H	-4.89977500	4.44099700	2.14712800
C	2.31498300	-2.09327700	-4.32305100
H	2.51303500	-2.91507700	-3.61585000
H	1.64276200	-2.46788800	-5.11608100
H	3.27564900	-1.81360800	-4.79077200
C	1.60106800	0.32577000	-4.56851000
H	2.61282400	0.63362300	-4.88390300
H	1.03054700	0.07634700	-5.48192000
H	1.11922400	1.18387900	-4.07428800
C	5.58605900	-1.23307800	-2.15893300
H	4.61808500	-0.77532600	-1.90365600
H	6.36474200	-0.46293800	-2.03166100
H	5.56653300	-1.53383700	-3.22166200
C	7.23193200	-3.08422500	-1.60064000
H	7.45281500	-3.94601500	-0.94626600
H	7.23163800	-3.43523000	-2.64749900
H	8.05904500	-2.35898000	-1.49969200
C	1.41771700	4.20696500	1.92476600
H	0.59490600	3.60465500	1.50369100
H	1.30610800	4.22212900	3.02886600
H	1.31368300	5.24365800	1.57144800
C	2.87850900	2.29740200	2.40983500
H	2.88468900	2.55086400	3.49038400
H	1.98770000	1.67101000	2.22464400
H	3.77006600	1.68790700	2.19872900
C	3.89771500	4.56644200	2.01192800
H	4.89649700	4.10022500	1.97129100
H	3.92119400	5.48160500	1.39327700
H	3.72485000	4.87900900	3.06218100
C	-2.26206900	4.06828400	1.42062100
H	-1.47038300	3.58023900	0.82601800
H	-2.80402500	4.77151300	0.76406000
H	-1.79602800	4.66422100	2.22516200
C	-2.15909300	-3.65576800	0.64501000
H	-2.97984400	-2.92509700	0.75857300
H	-2.45231900	-4.38783300	-0.12759200
H	-2.05430400	-4.19030800	1.60428900
C	-0.45622600	-1.92701100	1.37674500
H	-0.25759300	-2.44043500	2.33273400
H	0.43305900	-1.34337000	1.09890400
H	-1.27679600	-1.20056000	1.54145400
C	3.20475500	-3.28948500	4.07691700
H	3.67773300	-4.20927600	3.68992300
H	3.84533600	-2.89682700	4.88695800
H	2.23074800	-3.56193500	4.52203200
C	2.32681000	-0.97298500	3.48889200
H	2.10775900	-0.28758400	2.65867600
H	1.37674700	-1.22115000	3.99372300
H	2.97217300	-0.44413600	4.21323000
Cu	-2.02106100	0.14806600	-0.14585000

TS1-3

TPSS-D3(BJ)/def2-SVP

E = -5486.432679 a.u.

P	-0.33043500	0.59719800	-1.31857200
Si	0.66030300	2.93608400	-1.50565200
Si	1.81358600	0.59491800	-1.21515900
N	3.39281000	-2.28918800	-0.16621900
N	2.65990200	-0.01126300	0.17218000
N	4.46102800	-1.09414900	1.35354200
C	1.07446700	-2.92182400	-0.57525400
C	1.49203200	-2.62362600	-3.35196800
C	0.84495700	-3.10361200	0.91969700
C	4.35863800	-3.13336600	0.42229300
C	2.58086700	-2.43196300	-2.48226800
C	2.48468400	3.85688400	-1.45615100
C	4.99706600	1.72280600	3.72751900
C	-0.12527100	3.26475300	-3.30164500
C	2.35169300	-2.60428500	-1.09425300
C	0.22192700	-2.92798400	-2.85627500
C	6.57625900	0.26420600	-0.05759600
C	3.35849000	-0.23445500	3.87448800
C	3.42113900	-1.01577500	0.42325400
C	3.95616100	-2.06853800	-3.03076700
C	4.48619300	-4.56775400	0.03421100
C	4.48501200	0.52825400	3.19104200
C	0.01125400	-3.06866900	-1.48004500
C	6.04609500	0.76689700	1.28134700
C	6.15660200	-2.72755700	2.27182100

C	5.03211400	-2.38715200	1.35222500
C	6.52896800	1.95478600	1.85720500
C	6.00512500	2.43178700	3.06451800
C	-0.35783700	3.62918500	0.05509400
C	5.01584000	0.07724800	1.96273300
H	3.34921700	-1.24898400	3.43836300
H	4.58979000	-1.80448800	-2.16619000
H	5.26619200	-5.05909800	0.63623000
H	4.75384900	-4.67877200	-1.03214800
H	3.53668400	-5.11303400	0.18693200
H	6.44946400	-3.78179900	2.14830400
H	5.87400100	-2.56589600	3.32879000
H	7.04444100	-2.09757700	2.07684400
H	4.59763900	2.10596200	4.67223700
H	6.38709000	3.36492900	3.49269800
H	7.31725400	2.51992300	1.34945000
H	-0.99198300	-3.29051400	-1.10529000
H	-0.61736800	-3.04484500	-3.54711900
H	1.63615100	-2.49713700	-4.42978000
C	-3.84403900	-0.44364600	0.49859900
N	-4.75739100	0.31185000	1.19019900
N	-4.56598200	-1.54706600	0.11464300
C	-6.00693200	-0.30775500	1.24240500
C	-4.48126900	1.59988100	1.77830100
C	-5.88801100	-1.47960800	0.55447200
C	-4.01645100	-2.66398300	-0.61287000
H	-6.84813300	0.13595100	1.77013300
C	-5.13032700	2.73068700	1.22801400
C	-3.60353900	1.66864400	2.88572700
H	-6.60626400	-2.26813600	0.34380600
C	-3.70958400	-2.49989300	-1.98671900
C	-3.84883200	-3.88660300	0.07667400
C	-5.96140400	2.61811900	-0.05057900
C	-4.91523300	3.96610400	1.86361600
C	-3.41120300	2.93405300	3.46798000
C	-2.90375000	0.41912000	3.42064600
C	-3.25300700	-3.63529100	-2.67666100
C	-3.92385400	-1.16412500	-2.69379100
C	-3.36570900	-4.98401400	-0.66092300
C	-4.22086800	-4.04727700	1.54944100
H	-5.39693300	4.86589100	1.47231700
C	-4.07113400	4.06496300	2.97460100
H	-2.73499100	3.03697400	4.32005000
H	-2.52142300	-0.12597800	2.53569100
H	-3.02370900	-3.55486000	-3.74261100

C	-3.08093100	-4.86278900	-2.02429100
H	-3.71241100	-0.37360600	-1.94981900
H	-3.21826900	-5.94604600	-0.15911900
H	-4.47529600	-3.04971800	1.94375400
H	-3.90835100	5.03835300	3.44873700
H	-2.71042500	-5.72816200	-2.58325900
H	-6.53586200	1.67662300	0.00301300
H	1.80031300	-2.90270200	1.43219200
H	6.28835300	-0.79800200	-0.14912800
C	0.48952800	2.23353800	-4.28139000
H	0.26014800	1.20525100	-3.95278000
H	0.05737900	2.38607500	-5.29232200
H	1.58418500	2.32788500	-4.36246700
C	-1.65385800	3.04312400	-3.31554100
H	-1.93286700	2.10100700	-2.81195300
H	-2.19592600	3.87515400	-2.83650200
H	-2.00165700	2.98492100	-4.36707400
C	0.13152400	4.68268600	-3.85586900
H	1.20014100	4.86593200	-4.05275100
H	-0.40149300	4.80239000	-4.82102700
H	-0.23643000	5.47228200	-3.17573700
C	3.35024700	3.36604700	-2.63450700
H	2.98375700	3.75222900	-3.60134700
H	4.38905400	3.73106500	-2.50530400
H	3.37769700	2.26325600	-2.68647800
C	2.45483600	5.40492000	-1.55887900
H	3.50125400	5.76475900	-1.63849500
H	1.91796700	5.77331800	-2.44443000
H	2.02091700	5.88533200	-0.67196900
C	3.21620000	3.51344900	-0.13938100
H	2.72172100	3.97201800	0.73468000
H	3.28777600	2.42714000	0.04459400
H	4.25000500	3.91325500	-0.17489700
C	-1.69392300	0.73086500	4.31041500
H	-1.99325800	1.21509900	5.25764800
H	-1.17791400	-0.20867300	4.57042900
H	-0.97132800	1.38278600	3.79429300
C	-3.88940900	-0.51366500	4.15710800
H	-4.29269700	-0.01783900	5.05830200
H	-4.74030400	-0.80486300	3.51935600
H	-3.37229400	-1.43675600	4.47434200
C	-3.05655100	-4.57818600	2.40446400
H	-3.37071300	-4.65512400	3.46040600
H	-2.73075600	-5.58136300	2.07750300
H	-2.18978600	-3.90302300	2.35028800

C	-5.46799900	-4.94403600	1.69743600
H	-5.77658600	-5.00556800	2.75606400
H	-6.31919500	-4.55579200	1.11168600
H	-5.26034100	-5.96927500	1.34243500
C	-2.95132100	-0.91671500	-3.85530000
H	-1.90700900	-0.95028600	-3.50183500
H	-3.08563600	-1.64185900	-4.67863000
H	-3.12539800	0.09065600	-4.26949500
C	-5.39475200	-1.02409700	-3.13847300
H	-6.08747800	-1.12407900	-2.28482300
H	-5.56123900	-0.03504600	-3.59987500
H	-5.65414500	-1.80004700	-3.88127200
C	-6.97955500	3.75506600	-0.22566000
H	-7.63199100	3.85959400	0.65892100
H	-6.48077000	4.72452100	-0.40223400
H	-7.61777800	3.55235500	-1.10293000
C	4.59989700	-3.27746800	-3.74161300
H	4.66767300	-4.15325600	-3.07333400
H	4.00546600	-3.57529400	-4.62424900
H	5.61897200	-3.02633900	-4.08656800
C	3.90431000	-0.84736400	-3.96765800
H	4.92517300	-0.55425500	-4.27031700
H	3.33165200	-1.06745800	-4.88650700
H	3.42137100	0.00774700	-3.46090200
C	5.90824000	1.02127800	-1.22331300
H	4.81116500	0.92246000	-1.18413400
H	6.15083800	2.09751500	-1.18108000
H	6.25996200	0.62662400	-2.19314100
C	8.11120800	0.33200200	-0.14953400
H	8.58985500	-0.19723200	0.69338200
H	8.45525300	-0.13303900	-1.09028700
H	8.47530500	1.37498900	-0.14650900
C	-1.87278100	3.42164600	-0.10482000
H	-2.11467200	2.37852700	-0.39178300
H	-2.37537600	3.62406700	0.85829300
H	-2.30810200	4.09620600	-0.85847200
C	0.06865600	2.83656800	1.31633100
H	-0.40708800	3.28920900	2.21075600
H	-0.25768400	1.78498700	1.24080100
H	1.15891400	2.82991200	1.47448400
C	-0.15200700	5.13277800	0.34490700
H	0.86082800	5.34663800	0.72128100
H	-0.33419600	5.76524800	-0.54194300
H	-0.86477000	5.45012300	1.13328200
C	-5.04114900	2.51100400	-1.28687400
H	-4.29361800	1.70882600	-1.17024000
H	-5.63915300	2.30196000	-2.19115700
H	-4.49210100	3.45347500	-1.44390700
C	0.45272500	-4.56356500	1.22394500
H	-0.48968700	-4.83438300	0.71663100
H	1.23315500	-5.26305900	0.87680700
H	0.31322100	-4.71415800	2.30960600
C	-0.18458300	-2.09918600	1.46765000
H	-0.29700200	-2.21554500	2.56112700
H	0.12909700	-1.06536600	1.24797900
H	-1.17963300	-2.24676300	1.00557900
C	3.56955400	-0.37802400	5.39171600
H	4.54719700	-0.83732900	5.62200000
H	3.52420800	0.59942100	5.90494000
H	2.77765100	-1.01359200	5.82670600
C	1.99841700	0.41351800	3.54592600
H	1.85827300	0.48517100	2.45510800
H	1.17653200	-0.18731000	3.97199700
H	1.93731900	1.43159800	3.97214800
Cu	-2.06605500	0.01014900	-0.08344600

INT1-3

TPSS-D3(BJ)/def2-SVP

E = -5486.481856 a.u.

P	0.29263800	-1.27969500	0.26628900
Si	1.06571200	-2.99847600	-0.99347800
Si	-1.24561000	-0.28763500	-1.11134400
N	-4.22702200	1.70870500	0.41923800
N	-2.50066200	0.06051100	0.00049500
N	-4.81334900	-0.41336200	0.47226300
C	-3.17819100	3.67105100	1.41493700
C	-2.62323300	4.65315200	-1.17910600
C	-3.40817000	3.06057400	2.79582200
C	-5.60535200	1.66124900	0.74432800
C	-3.20063800	3.37610200	-1.05293900
C	-0.48477600	-4.09972000	-1.50743000
C	-4.41745600	-3.97041300	1.48098100
C	2.08698300	-2.49356100	-2.60567500
C	-3.49994700	2.92613500	0.25422800
C	-2.35269900	5.43574300	-0.04992500
C	-5.68889300	-1.65059400	-1.97585300
C	-3.80232700	-1.86298500	2.77506700
C	-3.70881700	0.41804000	0.25230000
C	-3.54262800	2.51789600	-2.26835300
C	-6.41217300	2.90285400	0.93847700

C	-4.34245000	-2.56728900	1.53699700
C	-2.60780500	4.94272400	1.23598700
C	-5.27700900	-2.47431400	-0.75917300
C	-7.26812600	-0.31581900	1.07795600
C	-5.96367300	0.34365100	0.77779400
C	-5.33443800	-3.87829500	-0.76606600
C	-4.91774300	-4.61832600	0.34730500
C	2.22106200	-3.96710100	0.27870000
C	-4.78863100	-1.84227600	0.40944000
H	-3.89478900	-0.77806600	2.60208300
H	-3.41667100	1.46602200	-1.96170000
H	-7.47137800	2.64203600	1.08842500
H	-6.34059800	3.56830700	0.05872000
H	-6.07638000	3.49092100	1.81221900
H	-8.03095500	0.44057100	1.31898700
H	-7.17801900	-1.00553600	1.93697600
H	-7.63038800	-0.91472400	0.22273200
H	-4.07621900	-4.56200800	2.33709200
H	-4.96968900	-5.71211200	0.32355600
H	-5.70659700	-4.40064100	-1.65296000
H	-2.35760600	5.55525900	2.10705500
H	-1.92675000	6.43727700	-0.17229100
H	-2.39950200	5.04696000	-2.17512300
C	3.63542200	1.10513300	0.21939500
N	4.49653900	1.15076900	1.28908900
N	3.88420200	2.27862200	-0.44512000
C	5.23725500	2.33280900	1.30105700
C	4.62741700	0.09671100	2.26873700
C	4.84599300	3.04905700	0.20535600
C	3.06191700	2.72026200	-1.54677800
H	5.95782600	2.55306100	2.08576000
C	5.80826700	-0.68290700	2.24778200
C	3.58036600	-0.10970300	3.19818500
H	5.14032800	4.03181100	-0.15529800
C	3.43224400	2.38613500	-2.86959700
C	1.88782700	3.43290200	-1.22748300
C	6.86752900	-0.48375700	1.16396500
C	5.93457000	-1.68965300	3.22045300
C	3.75842200	-1.13683600	4.14194200
C	2.31468100	0.74382100	3.18416600
C	2.56747600	2.79972400	-3.89710200
C	4.71875500	1.61628500	-3.15379400
C	1.05418400	3.81878000	-2.29233500
C	1.49416300	3.72583900	0.21400600
H	6.83037000	-2.31631300	3.23582000
C	4.92185700	-1.91178600	4.15924500
H	2.96774600	-1.33600500	4.86954100
H	2.05402100	0.90147800	2.11897900
H	2.80651800	2.55335700	-4.93503700
C	1.39114200	3.50540900	-3.61175000
H	4.83316900	0.87692100	-2.34048500
H	0.12288600	4.35072200	-2.07596000
H	2.33581500	3.43502000	0.86441700
H	5.03537700	-2.70635500	4.90424500
H	0.72438700	3.80096500	-4.42854300
H	6.93638500	0.59890500	0.95627100
H	-4.33532000	2.46372200	2.73971400
H	-5.92127700	-0.63220200	-1.61828500
C	1.38967300	-1.30523700	-3.30382500
H	1.37767100	-0.42175600	-2.63740000
H	1.94063600	-1.03201100	-4.22712100
H	0.34592100	-1.51367700	-3.58407600
C	3.50064300	-2.00621000	-2.23643000
H	3.45016100	-1.15323100	-1.52565500
H	4.12403400	-2.79196100	-1.78079900
H	4.02516300	-1.64704200	-3.14294400
C	2.24671300	-3.65999000	-3.60727400
H	1.28068500	-4.00177400	-4.01212100
H	2.86334600	-3.32911200	-4.46814300
H	2.75248100	-4.53022600	-3.15524100
C	-1.19308300	-3.51355500	-2.74756600
H	-0.58853000	-3.58559400	-3.66662400
H	-2.13947200	-4.06287100	-2.92576000
H	-1.45838100	-2.44735400	-2.59841800
C	-0.11025500	-5.56943100	-1.80863200
H	-1.01295100	-6.12107700	-2.14227600
H	0.64614800	-5.66108800	-2.60580000
H	0.27432300	-6.08765800	-0.91386000
C	-1.53794900	-4.09045400	-0.37718300
H	-1.14414700	-4.44303300	0.58822300
H	-1.95019900	-3.08017800	-0.21402900
H	-2.37918900	-4.75051000	-0.66177500
C	1.10202000	0.04668100	3.81409900
H	1.22137700	-0.09454900	4.90406400
H	0.20244800	0.66231800	3.65306300
H	0.91840800	-0.92928900	3.33466200
C	2.56810700	2.12463100	3.82340800
H	2.83219600	2.01862900	4.89124300
H	3.38936800	2.66371900	3.32080900
H	1.65974500	2.74873600	3.75368900

C	0.28968600	2.85850300	0.61870700
H	0.02044600	3.04307400	1.67045800
H	-0.59498100	3.07000500	-0.00321000
H	0.53373600	1.78574800	0.50639400
C	1.24447100	5.22683000	0.44587900
H	0.98117600	5.40943600	1.50277500
H	2.14248000	5.82202800	0.20289300
H	0.40971100	5.59623100	-0.17206800
C	4.68542600	0.84838100	-4.48423400
H	3.78234100	0.22301000	-4.56721500
H	4.71278200	1.53548800	-5.34913700
H	5.56807500	0.19020200	-4.55851400
C	5.94775800	2.55153300	-3.11670400
H	6.04992000	3.05789600	-2.14345500
H	6.87299700	1.97549300	-3.29687100
H	5.86625900	3.32686900	-3.89934100
C	8.27026700	-0.95234300	1.58090800
H	8.58405000	-0.49843000	2.53710600
H	8.31825700	-2.05041600	1.68986000
H	9.00438000	-0.66919100	0.80680100
C	-5.01473900	2.71118400	-2.68782600
H	-5.70249900	2.44735600	-1.86723500
H	-5.20883500	3.75913300	-2.98048000
H	-5.25305000	2.06258600	-3.54966900
C	-2.58910100	2.73554400	-3.45251600
H	-2.82585400	2.01322300	-4.25242500
H	-2.67917500	3.75029800	-3.88270900
H	-1.54312100	2.56392000	-3.14924800
C	-4.51070500	-1.52343900	-2.96316800
H	-3.61809100	-1.09859600	-2.47113300
H	-4.23072300	-2.51192800	-3.36769000
H	-4.78414400	-0.86731200	-3.80931200
C	-6.94682700	-2.19435600	-2.67471200
H	-7.78661100	-2.30880000	-1.96647800
H	-7.26275100	-1.50394300	-3.47652200
H	-6.76190300	-3.17768000	-3.14294400
C	3.16025400	-2.97447600	0.99740800
H	2.57796000	-2.23243100	1.57089700
H	3.81043600	-3.51711200	1.71281900
H	3.81635400	-2.42308600	0.30695900
C	1.35940600	-4.61496900	1.38738000
H	2.02166600	-5.03943500	2.16960200
H	0.70445400	-3.86436300	1.86486300
H	0.72895100	-5.43710300	1.00868300
C	3.08004200	-5.06696800	-0.38482500
H	2.46947600	-5.81727700	-0.91383600
H	3.80357900	-4.65120000	-1.10685600
H	3.66557600	-5.60221500	0.39065400
C	6.42523000	-1.16489000	-0.14882500
H	5.43942500	-0.79823700	-0.47492200
H	7.15434300	-0.96385100	-0.95395900
H	6.34934600	-2.25754100	-0.01246500
C	-3.59533900	4.10300000	3.90799500
H	-2.66837900	4.67620400	4.09064400
H	-4.39777300	4.82109000	3.66264800
H	-3.86022300	3.60075700	4.85460800
C	-2.27404000	2.07106600	3.14617100
H	-2.52238100	1.51589700	4.06833800
H	-2.10104500	1.34299700	2.33603300
H	-1.33027000	2.61638400	3.32224800
C	-4.63587500	-2.20416100	4.02607200
H	-5.70246400	-1.95717300	3.87946700
H	-4.56906000	-3.27869500	4.27489400
H	-4.26719300	-1.63516200	4.89839900
C	-2.30688400	-2.17000800	2.98347600
H	-1.70727900	-1.86708100	2.10789700
H	-1.92704700	-1.62213900	3.86492100
H	-2.14203800	-3.24900600	3.15932600
Cu	2.14490700	-0.08213300	-0.03227200

E-**4**

TPSS-D3(BJ)/def2-SVP

E = -3993.025045 a.u.

P	-0.28457300	1.20354800	-1.37962400
Si	2.78673700	3.01602000	-0.94011100
Si	1.69267700	0.89021400	-0.78488600
N	2.21544900	-2.68991600	-0.67521000
N	2.58186200	-0.35897000	-0.00836400
N	3.64623600	-2.28334500	0.94647500
C	-0.17528300	-3.00028100	-1.03254400
C	0.10234700	-2.01630500	-3.66541100
C	-0.32124200	-3.48113200	0.40891000
C	2.74419400	-3.93259500	-0.25720400
C	1.24289700	-2.07065500	-2.84646000
C	4.74302100	2.77801800	-0.88918300
C	4.83838500	-0.28714100	3.84558300
C	2.24924900	3.87529300	-2.64242900
C	1.07539200	-2.57554900	-1.53753100
C	-1.14265900	-2.44291700	-3.19452900
C	6.42093900	-2.07304600	0.19872700

C	2.56735600	-1.39212500	3.47988700
C	2.76269800	-1.63669000	0.06757800
C	2.61442700	-1.60814400	-3.33484000
C	2.34960700	-5.21630500	-0.91089100
C	3.98871500	-1.06549900	3.03872400
C	-1.28682500	-2.91612900	-1.88707000
C	5.88075100	-1.43460700	1.47705500
C	4.39425400	-4.59926700	1.64609800
C	3.62332400	-3.67750600	0.75932300
C	6.69610200	-0.67043800	2.32801600
C	6.17354100	-0.07921700	3.48585900
C	2.15251900	3.94728800	0.68644200
C	4.51891400	-1.58248900	1.83730400
H	2.15016500	-2.09195800	2.73652300
H	3.03493200	-0.97798600	-2.52749400
H	2.93716400	-6.04818300	-0.49225100
H	2.52991800	-5.17629900	-1.99996900
H	1.27790000	-5.44507400	-0.77250500
H	4.25164400	-5.64277600	1.32554200
H	4.05337100	-4.51007200	2.69436800
H	5.47658000	-4.37915300	1.64075200
H	4.45109200	0.14946800	4.77257100
H	6.82108100	0.53349200	4.12223500
H	7.75076300	-0.52260000	2.07788900
H	-2.27482200	-3.21998500	-1.53123500
H	-2.01704100	-2.39095600	-3.84872900
H	0.18418200	-1.62444200	-4.68199900
C	-4.06851700	0.22743400	0.52671800
N	-4.59930700	1.26838300	1.24095600
N	-5.17231300	-0.48044900	0.12606000
C	-5.99040600	1.21676200	1.27990400
C	-3.80219500	2.33583700	1.80019000
C	-6.35590100	0.10787000	0.56845400
C	-5.08347500	-1.63960900	-0.72587200
H	-6.57970300	1.96616500	1.80336300
C	-3.51893000	3.45518400	0.98538400
C	-3.33199800	2.20213800	3.12339100
H	-7.33280500	-0.30787000	0.33096000
C	-4.85570600	-1.43413200	-2.10455600
C	-5.23044200	-2.91899400	-0.13968700
C	-4.03631800	3.56221200	-0.44482600
C	-2.75457100	4.48581800	1.55806500
C	-2.56669700	3.25956700	3.64586000
C	-3.56666100	0.92838200	3.92594100
C	-4.74796500	-2.58175400	-2.91218200

C	-4.82036600	-0.03729700	-2.71794100
C	-5.09581000	-4.02999900	-0.99028400
C	-5.53936500	-3.06292500	1.35194300
H	-2.50479900	5.36526900	0.95745000
C	-2.28849900	4.39224800	2.87381600
H	-2.17472600	3.18907000	4.66560400
H	-4.34796000	0.34484400	3.40940300
H	-4.58235200	-2.46591300	-3.98788200
C	-4.85109100	-3.86215300	-2.36052500
H	-4.72052500	0.68694600	-1.89282200
H	-5.19144600	-5.03969400	-0.58206900
H	-6.25145000	-2.25603600	1.60512400
H	-1.68677000	5.20384300	3.29550100
H	-4.74886000	-4.74204100	-3.00415400
H	-4.35952000	2.55347800	-0.75482700
H	0.65602600	-3.87560500	0.73514500
H	5.83014300	-2.98725900	0.01667300
C	2.18379500	2.79961800	-3.75179600
H	1.37086300	2.08411500	-3.53992900
H	1.97043700	3.28559200	-4.72610800
H	3.12321400	2.23475500	-3.85917800
C	0.85332600	4.53621300	-2.60017000
H	0.09015200	3.83993500	-2.21566000
H	0.84772900	5.45597200	-1.99325900
H	0.56869300	4.82371200	-3.63293800
C	3.25577100	4.96650600	-3.07283700
H	4.24295400	4.54933500	-3.33223600
H	2.87110100	5.47926500	-3.97758500
H	3.40084400	5.73608500	-2.29325500
C	5.13305300	1.94622000	-2.13242500
H	5.01057500	2.52479500	-3.06348800
H	6.19806900	1.64795200	-2.06988200
H	4.52740400	1.02685900	-2.22149100
C	5.62957900	4.04623900	-0.88953800
H	6.69410200	3.73409700	-0.86277800
H	5.49455600	4.66817700	-1.78670600
H	5.45625000	4.68141300	-0.00761700
C	5.10951800	1.97261900	0.37919600
H	4.98677100	2.58135800	1.29150600
H	4.50133700	1.05899300	0.49090100
H	6.17598800	1.67473900	0.33622200
C	-2.27978600	0.07811900	3.92483800
H	-1.46723600	0.60396100	4.45627600
H	-2.44922100	-0.89293400	4.42346000
H	-1.93978000	-0.11142900	2.89321300

C	-4.06605900	1.20480500	5.35495600
H	-3.31027500	1.74397100	5.95324700
H	-4.98850600	1.81123200	5.34687500
H	-4.28121600	0.25385100	5.87372800
C	-4.28946200	-2.84433800	2.23055800
H	-4.57415700	-2.82458700	3.29745100
H	-3.56304400	-3.65974900	2.08382500
H	-3.78566800	-1.89548000	1.98912400
C	-6.22162200	-4.39435600	1.70331400
H	-6.53415800	-4.38617000	2.76178500
H	-7.11552700	-4.57269800	1.08074300
H	-5.53448100	-5.24931500	1.57212400
C	-3.61709100	0.18888600	-3.64859000
H	-2.66609100	0.03812200	-3.10908200
H	-3.64329700	-0.48287700	-4.52570100
H	-3.62880100	1.22690700	-4.02369100
C	-6.15898500	0.25321500	-3.42975900
H	-7.01297300	0.14234200	-2.73837600
H	-6.16600900	1.28430200	-3.82551000
H	-6.31646100	-0.43956200	-4.27617000
C	-5.27097800	4.48630800	-0.50320500
H	-6.07303900	4.13249100	0.16846400
H	-5.00504800	5.51480100	-0.19935500
H	-5.67449000	4.52644700	-1.53078300
C	3.58422800	-2.78852600	-3.55577200
H	3.76841400	-3.34750200	-2.62476500
H	3.18220200	-3.48706400	-4.31188300
H	4.55745000	-2.41362000	-3.91925000
C	2.53952300	-0.73984000	-4.59769700
H	3.53129700	-0.30636700	-4.81252000
H	2.23736700	-1.33336900	-5.48004900
H	1.82484000	0.08752200	-4.46857800
C	6.19654400	-1.16208900	-1.02087500
H	5.14343700	-0.84995300	-1.09267200
H	6.81175800	-0.25099500	-0.94300300
H	6.47204200	-1.68826800	-1.95215900
C	7.89456900	-2.49596900	0.31154000
H	8.06800400	-3.13510600	1.19534100
H	8.19344900	-3.06122700	-0.58853500
H	8.56598200	-1.62184900	0.38575500
C	0.70728900	4.47400300	0.55878900
H	0.02909800	3.70290200	0.15161900
H	0.34301800	4.74984300	1.56828600
H	0.64150300	5.37389400	-0.07177200
C	2.11823900	2.92338200	1.84411900
H	1.85831400	3.43742200	2.79260700
H	1.33214300	2.17057100	1.65484100
H	3.07027900	2.39125800	1.99558200
C	3.06506500	5.12728400	1.08266800
H	4.06578000	4.78853000	1.39919900
H	3.19275900	5.85378600	0.25986800
H	2.61767400	5.67089400	1.93995300
C	-2.94485200	4.00561000	-1.43406400
H	-2.07721000	3.32358800	-1.39710400
H	-3.34692700	3.99582900	-2.46273200
H	-2.59296500	5.03144100	-1.22525300
C	-1.34124900	-4.62274400	0.55027800
H	-2.36002000	-4.29673100	0.28045900
H	-1.08032200	-5.47549900	-0.10137000
H	-1.36949700	-4.98271900	1.59410300
C	-0.64540800	-2.30205400	1.34383300
H	-0.66910300	-2.63037100	2.39881800
H	0.09746000	-1.49548100	1.23673000
H	-1.63344500	-1.87227500	1.09454800
C	2.56663600	-2.11373900	4.84265900
H	3.20478200	-3.01481600	4.82099000
H	2.93917000	-1.45617900	5.64864400
H	1.53987900	-2.42253100	5.10966600
C	1.66078700	-0.15174300	3.49486500
H	1.60356100	0.28599700	2.48833300
H	0.64183100	-0.42679900	3.81552000
H	2.04288400	0.61918400	4.18817100
Ag	-2.09038700	0.21190500	-0.23817800

TS1-4

TPSS-D3(BJ)/def2-SVP

E = -3992.998767 a.u.

P	-0.16295300	0.82577600	-1.18560000
Si	0.86742300	3.14128100	-1.26474000
Si	1.96972900	0.76082300	-1.09650500
N	3.52230700	-2.26292000	-0.44475300
N	2.84158700	-0.02366000	0.17312500
N	4.59498800	-1.28727100	1.22209800
C	1.20282300	-2.88422100	-0.85606700
C	1.48871000	-2.12219900	-3.56128200
C	1.05269700	-3.27917800	0.60794000
C	4.44765200	-3.20103600	0.06235400
C	2.61762800	-2.06876700	-2.72291300
C	2.70660900	3.99969200	-1.00820300
C	5.01545200	1.21488500	3.94750200

C	0.26618600	3.54243200	-3.11453700
C	2.44958000	-2.45964200	-1.37138000
C	0.24150300	-2.50759700	-3.06234200
C	6.87069700	0.11522600	0.17185700
C	3.20192900	-0.55047000	3.66239800
C	3.57297700	-1.07247900	0.29709400
C	3.97732300	-1.63999600	-3.26326500
C	4.53432400	-4.58494400	-0.48728700
C	4.47645700	0.14686100	3.20689900
C	0.09683800	-2.88418500	-1.72174000
C	6.27996200	0.45697300	1.53610600
C	6.20621400	-3.07893600	1.98828700
C	5.12460400	-2.58971800	1.08441000
C	6.78750600	1.51053200	2.31465700
C	6.16265200	1.88464100	3.51130800
C	-0.29490300	3.84904900	0.19163800
C	5.13368000	-0.22376000	2.01421600
H	3.01166100	-1.37616700	2.95634900
H	4.62679700	-1.45931700	-2.38917600
H	5.28003100	-5.17294700	0.06987800
H	4.82533200	-4.58154600	-1.55361700
H	3.56152500	-5.10640300	-0.41995700
H	6.46484200	-4.12204600	1.74861400
H	5.89231500	-3.03167800	3.04768000
H	7.12252300	-2.46653300	1.89746000
H	4.52393500	1.52906900	4.87473300
H	6.56815300	2.71412800	4.10083200
H	7.67135100	2.05836200	1.97332900
H	-0.88891600	-3.17095700	-1.34417500
H	-0.63261600	-2.50304600	-3.71986900
H	1.58218800	-1.81939900	-4.60892800
C	-4.02822700	-0.60389000	0.53644200
N	-4.97306700	0.14196200	1.18693100
N	-4.70696200	-1.73415200	0.16309000
C	-6.20705900	-0.50642500	1.22496200
C	-4.73482900	1.46718200	1.70872600
C	-6.04054600	-1.69275100	0.56797000
C	-4.11097900	-2.80482300	-0.59916000
H	-7.07481300	-0.06388100	1.70930900
C	-5.24254100	2.56817400	0.97835800
C	-4.01824000	1.60282000	2.91669400
H	-6.73677600	-2.49867400	0.34595200
C	-3.88214300	-2.60045900	-1.97854200
C	-3.80403600	-4.01377200	0.07019500
C	-5.91772800	2.37721100	-0.37807500

C	-5.03053400	3.84733200	1.51841200
C	-3.81299600	2.90906300	3.39842800
C	-3.47667900	0.39866400	3.67840700
C	-3.31118000	-3.66714800	-2.69767200
C	-4.29596600	-1.31698900	-2.69103700
C	-3.23125200	-5.04280100	-0.69701100
C	-4.07144400	-4.17058700	1.56751400
H	-5.40202200	4.72608800	0.98378000
C	-4.32264800	4.01645200	2.71487000
H	-3.24901700	3.05644100	4.32495400
H	-3.87808600	-0.50819100	3.19638600
H	-3.12211300	-3.54915000	-3.76910000
C	-2.98517300	-4.87020900	-2.06560900
H	-4.52126800	-0.56455300	-1.91882800
H	-2.97453000	-5.99282500	-0.22125600
H	-5.02513800	-3.65538200	1.78218100
H	-4.15424900	5.02418300	3.10829000
H	-2.53199900	-5.68307300	-2.64211000
H	-6.36283700	1.36697600	-0.39233000
H	2.06650700	-3.36443500	1.03538800
H	6.61397700	-0.93615800	-0.04880300
C	0.95797600	2.54462900	-4.07614400
H	0.68438500	1.50710100	-3.81701300
H	0.62531700	2.74770100	-5.11553500
H	2.05601400	2.62242300	-4.05003500
C	-1.25511100	3.34237900	-3.29115300
H	-1.58658400	2.37632500	-2.87151700
H	-1.83775700	4.15212000	-2.82247700
H	-1.49602600	3.34662200	-4.37369900
C	0.59363800	4.98108200	-3.57045100
H	1.67855000	5.16575200	-3.62915500
H	0.17911300	5.15079700	-4.58512200
H	0.14922800	5.74111100	-2.90227800
C	3.65146900	3.57578000	-2.15200300
H	3.37561400	4.04498700	-3.11200200
H	4.68457200	3.89823500	-1.91149300
H	3.65878700	2.47972800	-2.28856700
C	2.72262100	5.55153900	-0.98905900
H	3.77793400	5.88941000	-1.03961300
H	2.19461900	6.00828000	-1.83932700
H	2.30262000	5.96793000	-0.06340500
C	3.33373100	3.52862700	0.32326400
H	2.78044500	3.91564500	1.19651300
H	3.38034400	2.42840300	0.41014000
H	4.37275900	3.90938300	0.39999800

C	-1.94028000	0.32715500	3.58112200
H	-1.46686500	1.21182600	4.04125200
H	-1.56428900	-0.57416800	4.09623600
H	-1.61258900	0.28280800	2.52641400
C	-3.95222800	0.38697300	5.14407600
H	-3.54863500	1.24605400	5.70881800
H	-5.05372100	0.42501000	5.20849300
H	-3.60693300	-0.53351800	5.64724800
C	-2.98308600	-3.46908700	2.40788300
H	-3.25665100	-3.48524100	3.47777500
H	-2.01455500	-3.98078400	2.29074000
H	-2.84741600	-2.42003600	2.09968500
C	-4.23877800	-5.63351100	2.00707600
H	-4.54957200	-5.67230700	3.06534300
H	-5.00104000	-6.15674000	1.40394200
H	-3.29026000	-6.19343100	1.92455700
C	-3.17408000	-0.73222800	-3.56713700
H	-2.26408000	-0.53961700	-2.97217600
H	-2.91567500	-1.40416600	-4.40535400
H	-3.50074900	0.22878600	-4.00067900
C	-5.59009000	-1.55300000	-3.49811700
H	-6.40508600	-1.92019300	-2.84960500
H	-5.92219300	-0.61209100	-3.97180600
H	-5.42966100	-2.29984900	-4.29653400
C	-7.05706400	3.37771600	-0.63446300
H	-7.79711900	3.36828800	0.18480600
H	-6.67782000	4.40931200	-0.74170100
H	-7.57678200	3.12244500	-1.57425600
C	4.60872300	-2.77604300	-4.09580000
H	4.69639200	-3.70722300	-3.50977400
H	3.99240800	-2.99693600	-4.98602900
H	5.61798800	-2.48876700	-4.44159100
C	3.91007400	-0.33543600	-4.07665500
H	4.92724900	-0.00821000	-4.35607300
H	3.33182500	-0.46579600	-5.00897000
H	3.42906900	0.46333500	-3.48408500
C	6.20116500	0.98463700	-0.91572300
H	5.10428100	0.87918000	-0.89186900
H	6.43630300	2.05188400	-0.75776800
H	6.56038200	0.69586700	-1.91995700
C	8.40260300	0.23578000	0.12158400
H	8.88209300	-0.36284200	0.91630700
H	8.77793400	-0.12076400	-0.85373400
H	8.73525000	1.28303200	0.23608000
C	-1.78941100	3.64253500	-0.11913700
H	-2.00387600	2.61387100	-0.45979100
H	-2.37988700	3.81600500	0.79920700
H	-2.15391200	4.34322800	-0.88731100
C	0.00281100	3.06292300	1.49176400
H	-0.61954900	3.46590700	2.31751300
H	-0.23752200	1.99423000	1.36785600
H	1.05896700	3.13283700	1.79857900
C	-0.11953800	5.35371300	0.49621100
H	0.83614000	5.56553200	1.00013100
H	-0.18113600	5.98319900	-0.40902100
H	-0.92614800	5.67998300	1.18442500
C	-4.86828300	2.42612200	-1.51029800
H	-4.05213500	1.70390200	-1.33518400
H	-5.33966000	2.19075100	-2.48109000
H	-4.41302800	3.42815700	-1.57862200
C	0.37579300	-4.65442400	0.75510300
H	-0.65289500	-4.64183600	0.35560000
H	0.93719900	-5.43578400	0.21281300
H	0.32269000	-4.94519200	1.81982000
C	0.31552600	-2.18131500	1.39642500
H	0.25637900	-2.44215500	2.46882500
H	0.82744000	-1.21308400	1.28601100
H	-0.71355300	-2.05162000	1.01450600
C	3.35200300	-1.16197300	5.06825300
H	4.19935700	-1.86923600	5.10912700
H	3.52532400	-0.38291900	5.83264400
H	2.43308100	-1.70748400	5.34931200
C	2.00119900	0.41235200	3.57837400
H	1.89172400	0.79261800	2.55085500
H	1.06970000	-0.10893400	3.85941200
H	2.13049400	1.27160000	4.26172900
Ag	-2.08099800	-0.00438700	-0.05694800

INT1-4 (7a)

TPSS-D3(BJ)/def2-SVP

E = -3993.034423 a.u.

P	0.08709500	1.25791300	-0.08785400
Si	-0.16413400	3.13226100	-1.31988900
Si	1.66507400	0.09643500	-1.30293900
N	4.01373800	-2.10118800	0.38095400
N	2.85164000	-0.02894200	-0.06889500
N	4.92846200	-0.23677700	1.11665600
C	2.04061000	-3.52943000	0.17872000
C	2.59908500	-3.86396500	-2.57214800
C	1.71993000	-3.29265400	1.64990900

C	5.18025800	-2.46258500	1.08755900
C	3.47245800	-3.08243800	-1.79582700
C	1.63490900	3.93136000	-1.46761400
C	4.59204500	3.09122600	2.74674000
C	-0.94300500	2.85005700	-3.11050400
C	3.17942400	-2.94296800	-0.41878200
C	1.46506700	-4.45107900	-2.00336100
C	6.69365400	1.25798600	-0.58799900
C	3.41031500	0.91004900	3.31673200
C	3.83047900	-0.71544000	0.40307300
C	4.68531400	-2.41173400	-2.43096100
C	5.56912000	-3.89234100	1.26176800
C	4.38525400	1.72605500	2.47762100
C	1.18342800	-4.28016200	-0.64335800
C	6.01712700	1.89788200	0.61929000
C	6.96661700	-1.05751000	2.37006200
C	5.74923200	-1.30281200	1.54291800
C	6.19755400	3.25530400	0.93305100
C	5.49246300	3.84602700	1.98908000
C	-1.33182300	4.27733900	-0.21932500
C	5.11700300	1.15120300	1.41498100
H	3.37821600	-0.10676600	2.89145300
H	5.10631200	-1.71678100	-1.68494700
H	6.52702300	-3.96694600	1.79949400
H	5.67735800	-4.40359800	0.28795800
H	4.80633700	-4.45156700	1.83539800
H	7.46276900	-2.01010600	2.61226200
H	6.71319400	-0.54630900	3.31692700
H	7.69302200	-0.41307400	1.84168800
H	4.03489300	3.56818800	3.56010000
H	5.63813000	4.90809000	2.21395000
H	6.88564600	3.86240200	0.33666000
H	0.27547300	-4.72154600	-0.22286700
H	0.78457100	-5.03686600	-2.62930800
H	2.79787800	-3.99265000	-3.64115500
C	-3.84135100	-0.88369200	0.25684100
N	-4.94090800	-0.33136700	0.86576200
N	-4.26721200	-2.13882600	-0.10018500
C	-6.01967600	-1.21575400	0.88402800
C	-5.00574300	0.98773400	1.45263100
C	-5.59943400	-2.35268500	0.26073800
C	-3.44615400	-3.18338300	-0.67043200
H	-6.97075500	-0.95315200	1.34153100
C	-5.86147500	1.94305300	0.84791400
C	-4.27491300	1.24884600	2.63292800
H	-6.11328600	-3.28451800	0.03566600
C	-2.99145000	-3.05836300	-2.00380800
C	-3.18662800	-4.32353400	0.13227800
C	-6.58007800	1.64339500	-0.46781000
C	-5.97942300	3.19192100	1.47869100
C	-4.42493800	2.52169000	3.21486100
C	-3.39526600	0.20501800	3.31114600
C	-2.27540600	-4.14368800	-2.54030800
C	-3.29669300	-1.81842900	-2.84021200
C	-2.47942200	-5.38238500	-0.46354700
C	-3.60793700	-4.38214900	1.60188600
H	-6.62582800	3.95741800	1.04165200
C	-5.27128600	3.47848100	2.65234700
H	-3.86913900	2.75812900	4.12768900
H	-3.32174900	-0.66140200	2.63620100
H	-1.90394600	-4.08379000	-3.56621600
C	-2.03970700	-5.29825700	-1.78862000
H	-3.18657700	-0.94588000	-2.16863900
H	-2.26706400	-6.28370600	0.11758700
H	-4.60015300	-3.90718100	1.68729100
H	-5.37442500	4.46149900	3.12342000
H	-1.49623300	-6.13796700	-2.23431000
H	-6.91477800	0.59152600	-0.43715500
H	2.65422700	-2.97015300	2.14215400
H	6.74889900	0.17116300	-0.39862100
C	-0.29254400	1.62225000	-3.78295200
H	-0.42670400	0.72047700	-3.16132500
H	-0.77359000	1.43877500	-4.76615700
H	0.78707100	1.74429400	-3.95694100
C	-2.44278800	2.52239700	-2.98199400
H	-2.59987800	1.68112400	-2.27960900
H	-3.03529900	3.38245400	-2.62870700
H	-2.85094700	2.21949700	-3.96816700
C	-0.78783400	4.07280100	-4.04274600
H	0.27018100	4.29411200	-4.26144700
H	-1.28588500	3.86886900	-5.01297000
H	-1.24475300	4.98418600	-3.62209900
C	2.43658300	3.27174400	-2.60924400
H	2.02098000	3.48927700	-3.60723400
H	3.48035300	3.64322600	-2.59165800
H	2.48520900	2.16753200	-2.49805400
C	1.58953600	5.45270700	-1.74387300
H	2.62242000	5.83530400	-1.87457000
H	1.02742000	5.70111100	-2.66024300
H	1.14014700	6.01285100	-0.90666600

C	2.44914400	3.69189200	-0.17241000
H	1.97004600	4.12002300	0.72118200
H	2.60855600	2.61750200	0.01936200
H	3.44606800	4.16506300	-0.27760500
C	-1.95971000	0.71264200	3.54218800
H	-1.93639000	1.58095100	4.22459500
H	-1.34919700	-0.08744400	3.99644200
H	-1.47836900	1.00341300	2.59157300
C	-4.04923600	-0.28345700	4.61994100
H	-4.14463200	0.53984500	5.35065300
H	-5.05904600	-0.69036700	4.43455900
H	-3.43526100	-1.07775900	5.08082000
C	-2.64289200	-3.55476500	2.47645400
H	-3.02491900	-3.48330900	3.51003700
H	-1.64722700	-4.02542600	2.50750600
H	-2.51731700	-2.53402300	2.08123700
C	-3.74886600	-5.81141500	2.14810200
H	-4.16555000	-5.77977500	3.16958800
H	-4.41934500	-6.42452100	1.52098300
H	-2.77225200	-6.32378500	2.21060600
C	-2.31049200	-1.60901100	-3.99765400
H	-1.26698200	-1.61971900	-3.64074600
H	-2.42442500	-2.37963700	-4.78184400
H	-2.49586700	-0.62861000	-4.46605900
C	-4.75488400	-1.83858700	-3.34536200
H	-5.47434800	-1.90799300	-2.51173100
H	-4.97449000	-0.91380600	-3.90708500
H	-4.92352600	-2.69994600	-4.01663800
C	-7.83148000	2.50481100	-0.69712700
H	-8.52833000	2.44903800	0.15739400
H	-7.57178300	3.56582100	-0.86044800
H	-8.36320700	2.15684100	-1.59951500
C	5.77681300	-3.44758400	-2.76990500
H	6.09278900	-4.00932700	-1.87396500
H	5.41043100	-4.17744100	-3.51453700
H	6.66628500	-2.94691500	-3.19273100
C	4.29641600	-1.58337800	-3.66984800
H	5.17333100	-1.02781900	-4.04624600
H	3.93404200	-2.23039600	-4.48927300
H	3.49715300	-0.86334300	-3.41936300
C	5.81230400	1.45614800	-1.83989100
H	4.79591100	1.06277100	-1.67524700
H	5.72201200	2.52974900	-2.08173800
H	6.25339100	0.94042600	-2.71150400
C	8.12709800	1.76073200	-0.82384600
H	8.75003400	1.64556800	0.08083500
H	8.59842700	1.19007100	-1.64334100
H	8.14459500	2.82612600	-1.11554500
C	-2.55536700	3.48147900	0.27974300
H	-2.24164800	2.62589700	0.90259300
H	-3.19229300	4.13598100	0.90537400
H	-3.17993300	3.09052800	-0.53664600
C	-0.57649700	4.73084400	1.05157900
H	-1.27891700	5.25793400	1.72966500
H	-0.16350700	3.86193600	1.59442900
H	0.25055700	5.42540100	0.82941300
C	-1.84700600	5.52650200	-0.96897100
H	-1.03010700	6.15385800	-1.36204900
H	-2.50362000	5.25631300	-1.81411600
H	-2.44656600	6.15498100	-0.27859900
C	-5.60119800	1.76751500	-1.65290900
H	-4.70973200	1.13725200	-1.50438100
H	-6.09036700	1.46047900	-2.59412900
H	-5.25996700	2.81018400	-1.76365200
C	1.24505300	-4.57478600	2.35601000
H	0.29604900	-4.94795900	1.93206100
H	1.99259300	-5.38265000	2.26516800
H	1.07424000	-4.37911500	3.42932500
C	0.70589200	-2.13738300	1.79555300
H	0.43169700	-1.98837900	2.85565600
H	1.12004200	-1.19226600	1.41043500
H	-0.21506300	-2.34979800	1.22659000
C	3.90482100	0.78886300	4.77280600
H	4.91461900	0.34393200	4.82062200
H	3.94901200	1.77805200	5.26357100
H	3.21923300	0.15141600	5.35976100
C	1.98276700	1.48261700	3.24561600
H	1.61079500	1.50785800	2.20629200
H	1.29439300	0.85284300	3.83751800
H	1.93912700	2.50691700	3.65971900
Ag	-1.97826900	0.07514200	-0.10625800

E-**5**

TPSS-D3(BJ)/def2-SVP

E = -3981.848814 a.u.

P	-0.25025800	1.19334000	-1.27766700
Si	2.70385200	3.09181800	-0.87099700
Si	1.75871900	0.89160200	-0.75688000
N	2.44573800	-2.64049200	-0.72569500
N	2.71701900	-0.31620000	-0.00627700

N	3.91409500	-2.20821200	0.85335600	C	-4.90009800	-1.91441300	-0.51975800
C	0.05498600	-3.01028900	-1.00916700	H	-6.53909500	1.66239900	1.96371300
C	0.22055100	-1.97723200	-3.63426700	C	-3.73836400	3.39276300	0.83030400
C	-0.02659700	-3.50775500	0.43118700	C	-3.24163900	2.29428400	3.00230800
C	3.04125700	-3.86801600	-0.35818300	H	-7.17810900	-0.74216500	0.65421200
C	1.38738200	-2.01213600	-2.85233700	C	-4.77573500	-1.75403200	-1.91715000
C	4.67006200	2.95238000	-0.91242800	C	-4.90737300	-3.17319800	0.12608300
C	5.14592600	-0.23115000	3.74936600	C	-4.33056700	3.34624900	-0.57338100
C	2.04808700	4.00424600	-2.50246000	C	-3.07259100	4.54108000	1.29240200
C	1.27559800	-2.54582300	-1.54915700	C	-2.58509800	3.46681100	3.41446300
C	-0.99593300	-2.44684700	-3.12983700	C	-3.22830100	1.03736900	3.86271500
C	6.63255000	-1.83301400	-0.02114000	C	-4.61676100	-2.92397500	-2.68311200
C	2.92512800	-1.45682000	3.46979800	C	-4.89950000	-0.38924000	-2.58790300
C	2.96863500	-1.58529100	0.02681100	C	-4.73579500	-4.30717400	-0.68668900
C	2.73007100	-1.50000800	-3.37011900	C	-5.11553200	-3.27217600	1.63910500
C	2.66465900	-5.15391100	-1.01790700	H	-2.97727100	5.40830400	0.63163200
C	4.29839000	-1.03302400	2.96343500	C	-2.51393600	4.58286800	2.57382100
C	-1.08533400	-2.94249500	-1.82575000	H	-2.11145300	3.50088000	4.40064600
C	6.12440100	-1.25474700	1.29832100	H	-3.99523100	0.35024800	3.46504900
C	4.79803800	-4.50215200	1.45997500	H	-4.52139600	-2.84398800	-3.77071500
C	3.94701800	-3.59759800	0.63090700	C	-4.58297700	-4.18224100	-2.07481000
C	6.94016500	-0.46891400	2.12889100	H	-4.85838700	0.37620400	-1.79604800
C	6.44704200	0.06110700	3.32858200	H	-4.72640200	-5.30199900	-0.23369600
C	2.09141200	3.90174300	0.82695800	H	-5.89503100	-2.53180700	1.89742300
C	4.79286800	-1.48643300	1.72191800	H	-1.99680300	5.48658800	2.91167100
H	2.49659000	-2.13781200	2.71558400	H	-4.44510400	-5.07944700	-2.68710800
H	3.17279100	-0.90382700	-2.54934600	H	-4.63172700	2.30362300	-0.77205600
H	3.29991100	-5.97087400	-0.64177300	H	0.96737000	-3.89594400	0.71286000
H	2.79217600	-5.08957600	-2.11315900	H	6.07705300	-2.76962000	-0.20075400
H	1.60904300	-5.42245600	-0.83383700	C	1.96942400	2.98446100	-3.66283300
H	4.68987700	-5.54340900	1.11935200	H	1.19670600	2.22491800	-3.45418000
H	4.50156300	-4.45396300	2.52419300	H	1.69133400	3.51144100	-4.59869100
H	5.86768300	-4.23063000	1.41176600	H	2.92503200	2.46639800	-3.84251700
H	4.78488100	0.15690700	4.70801400	C	0.63558600	4.61301000	-2.35482600
H	7.09260100	0.69155900	3.94944600	H	-0.08560100	3.87572800	-1.96663300
H	7.97102600	-0.25650400	1.83059700	H	0.63604700	5.50026400	-1.70099900
H	-2.05344300	-3.27255300	-1.44112500	H	0.28782400	4.94259000	-3.35508700
H	-1.89297700	-2.40822100	-3.75355100	C	2.99081200	5.15380100	-2.92628600
H	0.25847100	-1.56545800	-4.64577600	H	3.97685200	4.78817900	-3.25701000
C	-3.97801800	0.10056200	0.57148000	H	2.54000800	5.69413000	-3.78306100
N	-4.54810300	1.12358700	1.28321200	H	3.14885000	5.88986000	-2.11751300
N	-5.03317500	-0.72881800	0.28801900	C	5.04052900	2.17801600	-2.19829200
C	-5.92106800	0.94375200	1.43041100	H	4.84225500	2.77428100	-3.10492700
C	-3.82701900	2.29824400	1.71855500	H	6.12095700	1.93345900	-2.19643200
C	-6.23035100	-0.22783700	0.79786300	H	4.47796200	1.23134100	-2.28551600

C	5.49270500	4.26304600	-0.90871500
H	6.57134600	4.00313700	-0.91677600
H	5.30336800	4.89539800	-1.78835500
H	5.31244300	4.87081400	-0.00878800
C	5.13554200	2.12912600	0.31129400
H	5.02679900	2.70379200	1.24708900
H	4.58022100	1.18254800	0.42243300
H	6.21213000	1.88786300	0.20899300
C	-1.85961900	0.33961600	3.71330600
H	-1.05339100	0.97816100	4.11513100
H	-1.84777300	-0.62136600	4.25802100
H	-1.63424900	0.14553800	2.65020100
C	-3.57170900	1.30962000	5.33727400
H	-2.80923500	1.94425800	5.82284000
H	-4.54840200	1.81482400	5.43647800
H	-3.61459000	0.35947400	5.89847000
C	-3.85107500	-2.88153800	2.43376100
H	-4.08537000	-2.81994200	3.51125000
H	-3.05863700	-3.63418700	2.29733500
H	-3.44942400	-1.90878000	2.11010800
C	-5.63260800	-4.64696300	2.09056700
H	-5.88058300	-4.61806800	3.16562500
H	-6.53853800	-4.94649200	1.53548800
H	-4.86823000	-5.43243400	1.95227800
C	-3.74355900	-0.08189700	-3.55448300
H	-2.77530300	-0.11192000	-3.02615600
H	-3.71496100	-0.79299600	-4.39991200
H	-3.86966800	0.93098700	-3.97490400
C	-6.27419000	-0.26832200	-3.27912400
H	-7.09856300	-0.43900600	-2.56438800
H	-6.39638800	0.73966300	-3.71385900
H	-6.37554500	-1.00670000	-4.09496500
C	-5.59660400	4.22197800	-0.66599100
H	-6.35696800	3.90829900	0.07111000
H	-5.35758400	5.28352400	-0.47405800
H	-6.04280700	4.15005700	-1.67404000
C	3.70862800	-2.65087700	-3.68527100
H	3.93543500	-3.25000500	-2.78897800
H	3.28845100	-3.31989400	-4.45791900
H	4.66201700	-2.24342900	-4.06611300
C	2.58985800	-0.56871400	-4.58153600
H	3.56458000	-0.10528000	-4.81196500
H	2.26251800	-1.11985800	-5.48209700
H	1.86624600	0.23614800	-4.37953400
C	6.30995800	-0.90337000	-1.20419700
H	5.24182400	-0.63785800	-1.21891400
H	6.88692100	0.03282800	-1.13078600
H	6.56402800	-1.39331500	-2.16095000
C	8.12795200	-2.18812400	0.01304400
H	8.37151200	-2.84068700	0.87005000
H	8.41052400	-2.71532300	-0.91496200
H	8.76087200	-1.28527400	0.08110600
C	0.61332200	4.34646200	0.79692400
H	-0.04214800	3.56125800	0.37909600
H	0.28501800	4.54337600	1.83661300
H	0.46350100	5.27429300	0.22396900
C	2.17278400	2.82264100	1.93090000
H	1.92165700	3.27241400	2.91368400
H	1.42761900	2.03077200	1.73450200
H	3.16099600	2.34561000	2.01811900
C	2.95525800	5.11327400	1.23762400
H	3.99003900	4.82022300	1.48228700
H	2.99472100	5.88594100	0.44851200
H	2.52570500	5.58591500	2.14452500
C	-3.29049100	3.72280600	-1.64470900
H	-2.39979800	3.07500500	-1.57307900
H	-3.72799900	3.60290300	-2.65187800
H	-2.96383300	4.77309200	-1.54502600
C	-1.02611200	-4.66307000	0.60115300
H	-2.05876600	-4.34711300	0.37805800
H	-0.78228200	-5.50531300	-0.07081400
H	-1.00571200	-5.03536800	1.64088200
C	-0.32573400	-2.33704300	1.38572900
H	-0.33238600	-2.67882000	2.43673300
H	0.42347000	-1.53721400	1.27584800
H	-1.30705900	-1.88745400	1.15243200
C	3.05117100	-2.24849100	4.78752300
H	3.73176600	-3.11087000	4.67507200
H	3.44304000	-1.61454100	5.60319700
H	2.06167400	-2.62666000	5.10153700
C	1.95812800	-0.27149900	3.61297900
H	1.80653200	0.21340800	2.63851400
H	0.98005600	-0.61977700	3.98639600
H	2.34584000	0.48384000	4.32028800
Au	-2.07529400	0.21422400	-0.22504100

TS1-5

TPSS-D3(BJ)/def2-SVP

E = -3981.828675 a.u.

P	-0.20082000	1.08953500	-1.28819500

Si	1.10713900	3.37562500	-0.88621200
Si	1.92630900	0.94623000	-1.11489200
N	3.76670700	-2.11649800	-0.68394100
N	2.69218400	-0.06868600	0.03040900
N	4.19884200	-1.38118000	1.35211300
C	1.95746000	-3.03695400	-2.03953400
C	2.80854200	-1.44178100	-4.21191300
C	1.43342100	-3.78405200	-0.82011500
C	4.60165000	-3.07224100	-0.06190300
C	3.54108700	-1.39252800	-3.01294500
C	2.87803800	3.96172000	-0.09930500
C	3.37696600	0.67911000	4.33982800
C	0.90911800	4.07784100	-2.72776700
C	3.10257300	-2.21565200	-1.94602000
C	1.68522400	-2.26864200	-4.33617200
C	6.61949000	0.16972800	1.48793300
C	1.94913000	-1.07255200	3.16855700
C	3.47246400	-1.07178900	0.20026700
C	4.76426400	-0.49398300	-2.84367600
C	5.00291700	-4.33161000	-0.75573000
C	3.23880200	-0.27631900	3.31649600
C	1.26210000	-3.05680600	-3.26127900
C	5.52410300	0.29516300	2.54311000
C	5.65738300	-3.20832800	2.32019600
C	4.87579500	-2.61012600	1.19823600
C	5.62135800	1.22357500	3.59272300
C	4.55652400	1.41611600	4.48201900
C	-0.30351800	4.14555500	0.29293600
C	4.32761900	-0.45538800	2.43516700
H	2.08512600	-1.76598700	2.32241400
H	4.70100900	-0.06675600	-1.82723400
H	4.13290200	-4.98137200	-0.96290700
H	5.71435500	-4.89822900	-0.13510000
H	5.48334800	-4.12005000	-1.72800300
H	6.09348500	-4.17125700	2.01225200
H	5.01317900	-3.38444500	3.20187100
H	6.47886100	-2.54649900	2.65010700
H	2.54553900	0.84765900	5.03282600
H	4.64521000	2.15545400	5.28535900
H	6.53118800	1.82176400	3.70320400
H	0.36172500	-3.67173600	-3.35990500
H	1.12170700	-2.28048800	-5.27534300
H	3.10744800	-0.81204600	-5.05395500
C	-3.77746300	-0.73677500	0.46511700
N	-4.85088900	0.03303700	0.83452300
N	-4.30209100	-1.99942700	0.36030200
C	-6.01194500	-0.72818300	0.95392700
C	-4.84526200	1.47279300	0.95449200
C	-5.66654900	-2.01347800	0.65203700
C	-3.58703900	-3.17513000	-0.07197000
H	-6.96211100	-0.27952000	1.23461300
C	-5.13591400	2.23492200	-0.19806900
C	-4.61189000	2.04520000	2.22407300
H	-6.24966700	-2.93089600	0.61163600
C	-3.38513700	-3.37248100	-1.45467100
C	-3.19327800	-4.11169100	0.91250000
C	-5.32417200	1.59399500	-1.56869500
C	-5.23617800	3.62835600	-0.03735900
C	-4.72330300	3.44210100	2.32942400
C	-4.17687300	1.18540000	3.40449500
C	-2.79451300	-4.58834300	-1.84790100
C	-3.80426100	-2.34245000	-2.49718900
C	-2.60919900	-5.30770600	0.46502600
C	-3.35326100	-3.78913900	2.39579600
H	-5.45631500	4.25447700	-0.90806300
C	-5.04744100	4.22347700	1.21379600
H	-4.54678600	3.92561100	3.29486700
H	-4.50771400	0.15128100	3.20132500
H	-2.62900200	-4.78276500	-2.91269500
C	-2.42726500	-5.54971400	-0.90252900
H	-4.13737700	-1.43940500	-1.95997200
H	-2.29420600	-6.06154600	1.19187700
H	-4.28460500	-3.20467700	2.50707200
H	-5.13005100	5.31037700	1.31726800
H	-1.98208800	-6.49427300	-1.23054900
H	-5.23108400	0.50203100	-1.44355200
H	2.24288700	-3.81381900	-0.07144500
H	6.53163100	-0.83019600	1.02907500
C	1.66606700	3.18401900	-3.73530000
H	1.27263500	2.15285100	-3.72283300
H	1.53874900	3.59403400	-4.75878800
H	2.74517800	3.13812400	-3.51938000
C	-0.57360200	4.08790000	-3.16193400
H	-1.03038900	3.09078200	-3.03744900
H	-1.16995400	4.82248200	-2.59463500
H	-0.63715500	4.36903500	-4.23266100
C	1.44629900	5.52264500	-2.86516500
H	2.53055200	5.58819800	-2.67841200
H	1.27207500	5.87834600	-3.90098600
H	0.93957200	6.22787300	-2.18606800

C	3.99905800	3.73450100	-1.13515700
H	3.91879600	4.41178600	-2.00171000
H	4.98361400	3.91797400	-0.66074000
H	3.99063600	2.69536600	-1.51238900
C	2.92468100	5.44926500	0.32112400
H	3.95551600	5.69793300	0.64732800
H	2.66409700	6.13909200	-0.49816900
H	2.26060500	5.65932600	1.17564100
C	3.24152300	3.12156100	1.14807500
H	2.53447000	3.26207800	1.97907100
H	3.28626100	2.04188800	0.93044200
H	4.24232300	3.42732200	1.51407100
C	-2.63527800	1.16745900	3.48321300
H	-2.24332900	2.17785600	3.69348500
H	-2.29325900	0.48645400	4.28242700
H	-2.19640800	0.83078800	2.52785300
C	-4.80656600	1.61914900	4.73873000
H	-4.45039400	2.61630800	5.05277400
H	-5.90818600	1.65457700	4.67376500
H	-4.52909000	0.90737200	5.53580100
C	-2.19198800	-2.88627400	2.86721000
H	-2.35249700	-2.55950200	3.90995400
H	-1.23491700	-3.43432600	2.82122600
H	-2.09794700	-1.98999600	2.23177900
C	-3.48396300	-5.03373700	3.28683100
H	-3.70731300	-4.73038300	4.32422100
H	-4.29278700	-5.70021900	2.93980000
H	-2.54589500	-5.61651600	3.31240900
C	-2.61861600	-1.91777300	-3.38438600
H	-1.79656100	-1.50986600	-2.77187100
H	-2.23089000	-2.76420200	-3.97940400
H	-2.93752100	-1.12845500	-4.08710400
C	-4.99539300	-2.85526800	-3.33113400
H	-5.85356900	-3.11246100	-2.68554200
H	-5.32239000	-2.08102400	-4.04776900
H	-4.72205300	-3.75774100	-3.90720800
C	-6.72925900	1.87360700	-2.13620900
H	-7.51692400	1.52680500	-1.44425700
H	-6.88466400	2.95295100	-2.31276500
H	-6.86259500	1.35288700	-3.10105900
C	6.07423000	-1.30501800	-2.92100300
H	6.11518900	-2.07833500	-2.13716800
H	6.17243600	-1.79958700	-3.90418700
H	6.94391500	-0.63803900	-2.78266200
C	4.79132500	0.68657700	-3.82345700

H	5.61429600	1.37214200	-3.55772800
H	4.95814100	0.35511500	-4.86471900
H	3.84671500	1.25267200	-3.77845300
C	6.37826400	1.19901800	0.36378600
H	5.35895100	1.10647400	-0.04367500
H	6.48993000	2.22818900	0.74700000
H	7.10110700	1.05360000	-0.45902800
C	8.03999500	0.29025200	2.06333500
H	8.20630400	-0.43020900	2.88369100
H	8.78625400	0.09312500	1.27371800
H	8.23884300	1.30365500	2.45552500
C	-1.71259100	3.58335100	0.02577100
H	-1.79124600	2.51738700	0.30533900
H	-2.44106600	4.13581900	0.64808500
H	-2.02590000	3.67711100	-1.02401500
C	0.00390000	3.83208100	1.77461700
H	-0.85779300	4.14135100	2.40115500
H	0.16197100	2.75092000	1.93307500
H	0.88959700	4.37070600	2.14822800
C	-0.42068000	5.68189200	0.11992300
H	0.52967700	6.21636900	0.26016100
H	-0.81681600	5.94600000	-0.87534800
H	-1.13806300	6.07618900	0.86870900
C	-4.21208600	2.03070000	-2.54340900
H	-3.21742000	1.75543600	-2.15256900
H	-4.34805600	1.53418800	-3.52077600
H	-4.22820000	3.12220000	-2.71056100
C	1.04946200	-5.23812300	-1.13905200
H	0.23363600	-5.28861600	-1.87917000
H	1.91003800	-5.79964700	-1.54432800
H	0.69786800	-5.75070200	-0.22647400
C	0.26924500	-2.99366500	-0.18744500
H	-0.05820800	-3.47522300	0.74957800
H	0.57151500	-1.95689700	0.03378100
H	-0.59865100	-2.94970100	-0.86657700
C	1.66619300	-1.92074500	4.42439600
H	2.50872700	-2.59881100	4.64901300
H	1.49953300	-1.28398100	5.31216000
H	0.75874800	-2.53319000	4.27663700
C	0.76350500	-0.15328900	2.82181500
H	0.96417800	0.40154300	1.89247400
H	-0.14938100	-0.75104900	2.66429600
H	0.56276700	0.56762400	3.63501200
Au	-1.95756300	0.00329400	-0.18651900

INT1-5

TPSS-D3(BJ)/def2-SVP

E = -3981.859436 a.u.

P	-0.27730900	1.68948400	0.10519600
Si	-1.04152300	3.51507700	-1.02423500
Si	1.83290600	1.59779200	-0.62646300
N	4.53222700	-1.21446200	-0.14979600
N	2.48901900	0.06695200	-0.16169500
N	4.39268300	0.45322300	1.28764500
C	3.92575400	-3.50053400	-0.74225500
C	3.90665500	-2.77265500	-3.47355500
C	3.78789500	-3.81596000	0.74469600
C	5.73345100	-1.20159800	0.59355200
C	4.16749800	-1.77788300	-2.51148800
C	0.24655600	5.00163300	-0.79645200
C	2.43524000	2.77691600	3.44331300
C	-1.29526500	3.10712300	-2.93297400
C	4.19724300	-2.17256500	-1.15650000
C	3.70504300	-4.10361900	-3.09593300
C	5.67031300	2.98413500	0.54065800
C	2.16559300	0.25239400	3.11105900
C	3.68071500	-0.19356800	0.26877800
C	4.44092500	-0.34008600	-2.93596900
C	6.85883000	-2.13227000	0.28236000
C	2.85097700	1.58539300	2.82700200
C	3.71217900	-4.46328100	-1.74124000
C	4.59290500	2.88132400	1.61832900
C	6.58202000	0.27139200	2.56345200
C	5.64158100	-0.17412200	1.49344900
C	4.15317800	4.03845300	2.28527400
C	3.08987900	3.98775100	3.19081700
C	-2.74599200	3.96449700	-0.14245300
C	3.94270300	1.65938100	1.92260900
H	2.07951600	-0.26629500	2.14055400
H	4.43912900	0.28284300	-2.02775700
H	6.57080800	-3.19394400	0.38568100
H	7.70755400	-1.93768100	0.95608000
H	7.20581500	-1.99436100	-0.75869200
H	7.45114600	-0.40309900	2.60815100
H	6.08704100	0.26491500	3.55151800
H	6.95029900	1.29808300	2.39536800
H	1.58403800	2.75871800	4.12863000
H	2.74886000	4.90435600	3.68350800
H	4.63035000	4.99909200	2.06556700
H	3.53890900	-5.50532900	-1.46024300
H	3.53201200	-4.86841500	-3.86134100
H	3.88162100	-2.49991800	-4.53387900
C	-3.09465900	-1.70081000	0.14734400
N	-4.43232100	-1.62181000	0.45713200
N	-2.90293100	-3.02881100	-0.13954300
C	-5.05102300	-2.86846100	0.36774800
C	-5.16249000	-0.43647600	0.84283000
C	-4.09071100	-3.75304700	-0.01928900
C	-1.66280200	-3.71102600	-0.43127800
H	-6.10682400	-2.99889400	0.59375400
C	-6.12976700	0.06737400	-0.06256100
C	-4.95523300	0.10552900	2.12922800
H	-4.12545300	-4.82215000	-0.21605500
C	-1.05144600	-3.55847900	-1.69214500
C	-1.19435600	-4.62116900	0.55160000
C	-6.27709300	-0.52109100	-1.46554400
C	-6.91288600	1.14975500	0.37004600
C	-5.76746400	1.19054200	2.50850000
C	-3.93962700	-0.46996300	3.10905900
C	0.01725700	-4.42546600	-1.99446900
C	-1.50789700	-2.52461200	-2.71322400
C	-0.15540000	-5.48808800	0.18064600
C	-1.73445300	-4.57107500	1.98022500
H	-7.66732600	1.57121700	-0.29939000
C	-6.73865400	1.70212100	1.64521700
H	-5.63414700	1.63570800	3.49944900
H	-3.30379300	-1.18015900	2.55770400
H	0.52363600	-4.33424800	-2.96003600
C	0.42734500	-5.40651100	-1.09107000
H	-2.27243900	-1.89077300	-2.23465100
H	0.21984800	-6.22192600	0.89891200
H	-2.82611600	-4.41061000	1.92993300
H	-7.36067400	2.54588800	1.96168100
H	1.23134500	-6.09545400	-1.36459000
H	-6.15742100	-1.61598800	-1.38211700
H	4.58575500	-3.27215600	1.28111700
H	5.98357400	1.95911600	0.27732700
C	-0.05110200	2.39187200	-3.50416900
H	0.13509100	1.44669500	-2.96679200
H	-0.22368800	2.14873700	-4.57288300
H	0.86514700	2.99804900	-3.43901500
C	-2.47433600	2.12826900	-3.09790500
H	-2.35616000	1.25171300	-2.43397000
H	-3.44368200	2.60493800	-2.87592900
H	-2.51785200	1.76014800	-4.14328600

C	-1.58056700	4.36865200	-3.78018900
H	-0.73083200	5.07130500	-3.77919500
H	-1.76294300	4.07675200	-4.83460500
H	-2.47195500	4.91559600	-3.43045900
C	1.38853800	4.98065900	-1.84006400
H	1.02516800	5.14408100	-2.86824000
H	2.09843300	5.80065700	-1.60805500
H	1.95053800	4.03144100	-1.80747100
C	-0.44817200	6.37857100	-0.92619600
H	0.31614400	7.17738000	-0.84167000
H	-0.94825600	6.50509000	-1.90217800
H	-1.19325800	6.55659500	-0.13382300
C	0.92155900	4.91519100	0.59184800
H	0.20139700	4.88184200	1.42475600
H	1.55471900	4.01511800	0.67111200
H	1.57691300	5.79795500	0.73951900
C	-3.00611600	0.61635100	3.67229300
H	-3.56123500	1.37354600	4.25407100
H	-2.25794200	0.15948900	4.34215200
H	-2.46608500	1.12613700	2.85650300
C	-4.65296100	-1.25504900	4.22888000
H	-5.30472600	-0.59348000	4.82768700
H	-5.28130800	-2.06193600	3.81203200
H	-3.91187500	-1.71116500	4.90949200
C	-1.12972700	-3.34948600	2.71236500
H	-1.59558100	-3.22642700	3.70624200
H	-0.04368700	-3.48220100	2.85098400
H	-1.28033500	-2.41883700	2.13882300
C	-1.50183700	-5.86149900	2.77917900
H	-1.99750400	-5.78753900	3.76248200
H	-1.90701500	-6.74472000	2.25490600
H	-0.42731400	-6.03579600	2.96692700
C	-0.34149300	-1.59850000	-3.11152100
H	0.09061700	-1.10888100	-2.22239800
H	0.46336200	-2.15377400	-3.62504800
H	-0.70289000	-0.81118900	-3.79473100
C	-2.15718100	-3.19656100	-3.93895800
H	-3.01544500	-3.82527200	-3.64242300
H	-2.51886500	-2.43003400	-4.64717000
H	-1.43440400	-3.83929200	-4.47346600
C	-7.65342100	-0.26764300	-2.09927900
H	-8.47447700	-0.60350000	-1.44196900
H	-7.80793900	0.80285600	-2.32326000
H	-7.73040700	-0.81488000	-3.05463800
C	5.83461100	-0.21471400	-3.58381100
H	6.62540200	-0.56160500	-2.89553600
H	5.90169600	-0.81750800	-4.50785400
H	6.04401400	0.83794100	-3.84569900
C	3.33268200	0.20464000	-3.85471000
H	3.49692100	1.27777200	-4.05332200
H	3.30978800	-0.32164200	-4.82661900
H	2.34651600	0.09743400	-3.37519700
C	5.08740100	3.62455000	-0.73647600
H	4.19202300	3.07345400	-1.07668300
H	4.78237200	4.66958200	-0.54722700
H	5.84042500	3.62502300	-1.54563700
C	6.91752800	3.75259000	1.01711200
H	7.33575400	3.32462700	1.94543400
H	7.70211700	3.72494100	0.24032100
H	6.68508900	4.81426200	1.21422100
C	-3.64108000	2.71974300	0.02526000
H	-3.14766800	1.94431900	0.63625200
H	-4.57385100	3.00944200	0.54459100
H	-3.92218000	2.25842000	-0.93156000
C	-2.46058800	4.46875400	1.29233100
H	-3.41924200	4.59680700	1.83513600
H	-1.84987100	3.73578700	1.85020900
H	-1.93728100	5.43872300	1.31284300
C	-3.58263800	5.01800600	-0.90477400
H	-3.03880100	5.95752800	-1.08891300
H	-3.92833100	4.62842800	-1.87811200
H	-4.48834300	5.26623400	-0.31455100
C	-5.14628400	-0.01468200	-2.38389500
H	-4.15447200	-0.18367000	-1.93350400
H	-5.17842900	-0.53045100	-3.35995400
H	-5.25197600	1.06827800	-2.56220400
C	3.94044900	-5.30607700	1.08184700
H	3.11458600	-5.90547000	0.65854900
H	4.89229100	-5.71772800	0.70174200
H	3.91859300	-5.44702000	2.17652800
C	2.44346700	-3.26473100	1.26941500
H	2.38999900	-3.37890900	2.36671800
H	2.31051500	-2.20047800	1.01865600
H	1.60290600	-3.81379400	0.81901900
C	3.02126400	-0.62224700	4.05118500
H	4.01015900	-0.83927400	3.61407700
H	3.17351900	-0.12083200	5.02431000
H	2.51711100	-1.58722900	4.23839700
C	0.73537900	0.40510300	3.64664000
H	0.13476500	1.03788800	2.97158100

H	0.25455700	-0.58739200	3.69576200	Au	-1.74481300	-0.14787000	0.09193300
H	0.71283200	0.83568000	4.66516900				

5. References

(S1) Zhu, H.; Kostenko, A.; Franz, D.; Hanusch, F.; Inoue, S. Room Temperature Intermolecular Dearomatization of Arenes by an Acyclic Iminosilylene. *J. Am. Chem. Soc*. **2023**, *145*, 1011-1021.

(S2) Puschmann, F. F.; Stein, D.; Heift, D.; Hendriksen, C.; Gal, Z. A.; Grützmacher, H.-F.; Grützmacher, H. Phosphination of Carbon Monoxide: A Simple Synthesis of Sodium Phosphaethynolate (NaOCP). *Angew. Chem. Int. Ed*. **2011**, *50*, 8420-8423.

(S3) Marion, R.; Sguerra, F.; Meo, F. D.; Sauvageot, E.; Lohier, J.; Daniellou, R.; Renaud, J.; Linares, M.; Hamel, M.; Gaillard, S. NHC Copper(I) Complexes Bearing Dipyridylamine Ligands: Synthesis, Structural, and Photoluminescent Studies. *Inorg. Chem.* **2014**, *53*, 9181-9191.

(S4) Wang, H. M. J.; Lin, I. J. B. Facile Synthesis of Silver(I)−Carbene Complexes. Useful Carbene Transfer Agents. *Organometallics* **1998**, *17*, 972-975.

(S5) de Frémont, P.; Scott, N. M.; Stevens, E. D.; Nolan, S. P. Synthesis and Structural Characterization of N-Heterocyclic Carbene Gold(I) Complexes. *Organometallics* **2005**, *24*, 2411-2418.

(S6) Frey, G. D.; Dewhurst, R. D.; Kousar, S.; Donnadieu, B.; Bertrand, G. Cyclic (alkyl)(amino)carbene gold(I) complexes: A synthetic and structural investigation. *J. Organomet. Chem.* **2008**, 1674-1682.

(S7) *APEX suite of crystallographic software*, APEX 4 version 2021.10-0; Bruker AXS Inc.: Madison, Wisconsin, USA, 2021.

(S8) *CrysAlisPRO*, Oxford Diffraction /Agilent Technologies UK Ltd, Yarnton, England.

(S9) *SAINT, Version 7.56a and SADABS Version 2008/1*; Bruker AXS Inc.: Madison, Wisconsin, USA, 2008.

(S10) Sheldrick, G. M. *SHELXL-2014*, University of Göttingen, Göttingen, Germany, 2014.

(S11) Hübschle, C. B.; Sheldrick, G. M.; Dittrich, B. *J. Appl. Cryst.* **2011**, *44*, 1281-1284.

(S12) Sheldrick, G. M. *SHELXL-97*, University of Göttingen, Göttingen, Germany, 1998.

(S13) Wilson, A. J. C. International Tables for Crystallography, Vol. C, Tables 6.1.1.4 (pp. 500-502), 4.2.6.8 (pp. 219-222), and 4.2.4.2 (pp. 193-199); Kluwer Academic Publishers: Dordrecht, The Netherlands, 1992.

(S14) Macrae, C. F.; Bruno, I. J.; Chisholm, J. A.; Edgington, P. R.; McCabe, P.; Pidcock, E.; Rodriguez-Monge, L.; Taylor, R.; van de Streek, J.; Wood, P. A. *J. Appl. Cryst.* **2008**, *41*, 466-470.

(S15) Frisch, M. J.; Trucks, G. W.; Schlegel, H. B.; Scuseria, G. E.; Robb, M. A.; Cheeseman, J. R.; Scalmani, G.; Barone, V.; Mennucci, B.; Petersson, G. A.; Nakatsuji, H.; Caricato, M.; Li, X.; Hratchian, H. P.; Izmaylov, A. F.; Bloino, J.; Zheng, G.; Sonnenberg, J. L.; Hada, M.; Ehara, M.; Toyota, K.; Fukuda, R.; Hasegawa, J.; Ishida, M.; Nakajima, T.; Honda, Y.; Kitao, O.; Nakai, H.; Vreven, T.; Montgomery, J. A., Jr.; Peralta, J. E.; Ogliaro, F.; Bearpark, M.; Heyd, J. J.; Brothers, E.; Kudin, K. N.; Staroverov, V. N.; Kobayashi, R.; Normand, J.; Raghavachari, K.; Rendell, A.; Burant, J. C.; Iyengar, S. S.; Tomasi, J.; Cossi, M.; Rega, N.; Millam, J. M.; Klene, M.; Knox, J. E.; Cross, J. B.; Bakken, V.; Adamo, C.; Jaramillo, J.; Gomperts, R.; Stratmann, R. E.; Yazyev, O.; Austin, A. J.; Cammi, R.; Pomelli, C.; Ochterski, J. W.; Martin, R. L.; Morokuma, K.; Zakrzewski, V. G.; Voth, G. A.; Salvador, P.; Dannenberg, J. J.; Dapprich, S.; Daniels, A. D.; Farkas, O.; Foresman, J. B.; Ortiz, J. V.; Cioslowski, J.; Fox, D. J., Gaussian 16, Revision A.03; Gaussian, Inc., Wallingford CT, **2016**.

(S16) Tao, J. M., Perdew, J. P., Staroverov, V. N., Scuseria. G. E., Climbing the density functional ladder: Nonempirical meta-generalized gradient approximation designed for molecules and solids. *Phys. Rev. Lett.* **2003**, *91*, 146401.

(S17) (a) Grimme, S.; Antony, J.; Ehrlich, S.; Krieg, H. A consistent and accurate ab initio parameterization of density functional dispersion correction (DFT-D) for the 94 elements H-Pu. *J. Chem. Phys.* **2010**, *132*, 154104. (b) Grimme, S.; Ehrlich, S.; Goerigk, L. Effect of the damping function in dispersion corrected density functional theory. *J. Comput. Chem.* **2011**, *32*, 1456-1465.

(S18) Weigend, F., Ahlrichs. R., Balanced basis sets of split valence, triple zeta valence and quadruple zeta valence quality for H to Rn: Design and assessment of accuracy. *Phys. Chem. Chem. Phys.* **2005**, *7*, 3297-3305.

(S19) Marenich, A. V.; Cramer, C. J.; Truhlar, D. G. Universal Solvation Model Based on Solute Electron Density and a Continuum Model of the Solvent Defined by the Bulk Dielectric Constant and Atomic Surface Tensions. *J. Phys. Chem. B* **2009**, *113*, 6378-6396.

(S20) (a) Fukui, K. Formulation of the reaction coordinate. *J. Phys. Chem*. **1970**, *74*, 4161-4163. Fukui, K. (b) The path of chemical reactions-the IRC approach. *Acc. Chem. Res.* **1981**, *14*, 363-368.

(S21) Glendening, E. D.; Badenhoop, J. K.; Reed, A. E.; Carpenter, J. E.; Bohmann, J. A.; Morales, C. M.; Landis, C. R.; Weinhold, F. NBO 7.0; Theoretical Chemistry Institute, University of Wisconsin: Madison, WI, **2013**. http://nbo7.chem.wisc.edu/.

(S22) Lu, T.; Chen, F. Multiwfn: a multifunctional wavefunction analyzer. *J. Comput. Chem.* **2012**, *33*, 580-592.

(S23) C. Y. Legault, CYLview, 1.0b ed.; Université de Sherbrooke: Sherbrooke, Québec, Canada, **2009**; http://www.cylview.org.

(S24) Humphrey, W.; Dalke, A.; Schulten, K. VMD: Visual molecular dynamics. *J. Mol. Graphics* **1996**, *14*, 33-38.

(S25) Wilson, P. J.; Bradley, T. J.; Tozer, D. J. Hybrid exchange-correlation functional determined from thermochemical data and ab initio potentials. *J. Chem. Phys.* **2001**, *115*, 9233-9242.
